# Catalytic enantioselective addition of organometallics to unprotected carboxylic acids

**DOI:** 10.1038/s41467-019-11345-z

**Published:** 2019-07-30

**Authors:** Xingchen Yan, Syuzanna R. Harutyunyan

**Affiliations:** Stratingh Institute for Chemistry, University of Groningen, Nijenborgh 4, 9747 AG Groningen, The Netherlands

**Keywords:** Asymmetric catalysis, Synthetic chemistry methodology, Homogeneous catalysis

## Abstract

Conjugate addition of organometallics to carbonyl based Michael acceptors is a widely used method that allows the building of new carbon-carbon (C-C) bonds and the introduction of chirality in a single step. However, conjugate additions to the simplest Michael acceptors, namely unprotected, unsaturated carboxylic acids, are considered to be prohibited by the fact that acid-base reactions overpower any other type of reactivity, including nucleophilic addition. Here we describe a transient protecting group strategy that allows efficient catalytic asymmetric additions of organomagnesium reagents to unprotected *α*,*β*-unsaturated carboxylic acids. This unorthodox pathway is achieved by preventing the formation of unreactive carboxylate salts by means of a reactive intermediate, allowing modifications of the carbon chain to proceed unhindered, while the stereochemistry is controlled with a chiral copper catalyst. A wide variety of *β*-chiral carboxylic acids, obtained with excellent enantioselectivities and yields, can be further transformed into valuable molecules through for instance catalytic decarboxylative cross-coupling reactions.

## Abstract (web summary)

Unprotected carboxylic acids are classically deemed to be unsusceptible to conjugated additions due to acid-base neutralization. Here, the authors show that the use of an appropriate Lewis acid allows the catalytic addition of organometallics to unprotected carboxylic acids, also in an asymmetric fashion.

## Introduction

**Fig. 1:**
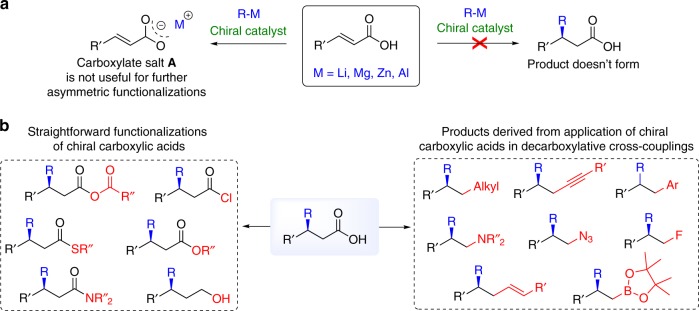
State of the art in conjugate additions to unsaturated carboxylic acids and potential value of addition products. **a** Fundamental problem that prevents the development of conjugate additions to unprotected unsaturated carboxylic acids: mixing of organometallics with carboxylic acids leads to an acid–base reaction, resulting in a carboxylate salt **A** nearly unreactive toward further reactions. **b** Overview of transformations of the carboxylic acid functional group leading to its chiral derivatives and new structural motives: carboxylic acids can undergo straightforward functionalization and decarboxylative cross-coupling reactions

Unprotected carboxylic acids are essential constituents of biologically active compounds and essential precursors in the synthesis of numerous useful derivatives^1–3^. As such, they are often produced industrially and used in the production of polymers, pharmaceuticals, solvents, and food additives. One of the simplest ways to generate target carboxylic acids would exist of taking simple, readily available variants, and modifying the carbon chain by, for example, introducing functional groups, forming additional carbon–carbon bonds, and introducing chirality. This implies (asymmetric) conjugate addition of organometallics, a highly important and widely used method, that allows the introduction of carbon–carbon (C–C) bonds and chirality in a single step^4–7^. However, applying this method to unprotected *α,β*-unsaturated carboxylic acids is inhibited by a fundamental problem, namely that upon mixing with common organometallics the acidity of the carboxylic acids and the basicity of the organometallics invariably leads to deprotonation and the formation of carboxylate salts, as the organometallic functions primarily as a base instead of as a nucleophile (Fig. 1a). Once the salt is formed, further organic reactions become unfeasible because of the inherent low reactivity and insolubility of salts in organic solvents. Consequently, even though the first effort to do this, in a non-enantioselective manner, dates back to 1953^8^, and despite many further attempts^9–13^, to our knowledge no examples of direct applications of unsaturated carboxylic acids in either catalytic or stoichiometric enantioselective reactions with hard organometallics, nor with organoboron or organosilicon reagents, are known.

This is unfortunate, given that carboxylic acids are not just the main precursors of various carbonyl compounds and common components of biologically active compounds but, perhaps even more importantly, because of their potential for application in decarboxylative coupling reactions that have witnessed tremendous progress in recent years and would allow access to a variety of *β*-chiral functionalized molecules in a simple manner (Fig. 1b)^14–19^. Therefore, developing a successful strategy for asymmetric conjugate additions to carboxylic acids would also allow direct access to *β*-chiral carboxylic acids and other chiral functional molecules, starting from simple substrates and without any derivatizations or protecting and deprotecting steps.

So far, chiral *β*-substituted carboxylic acids are mainly obtained by kinetic resolution or asymmetric hydrogenation reactions^20–23^. However, these methods are often limited to aryl substituents in the *β*-position of the substrate, require precious transition metal catalysts and make use of molecular frameworks, in which all the carbons have already been preinstalled. Thus, all C–C bonds must be formed in preceding reactions. Another common, indirect way is through asymmetric additions to premade *α*,*β*-unsaturated esters, thioesters, or amides using chiral catalysts or chiral auxiliaries, followed by a hydrolysis step^4–7,24–28^.

Our goal was to develop a general platform for direct catalytic synthesis of enantiopure *β*-substituted carboxylic acids from simple carboxylic acid-building blocks via C–C bond-forming reactions. Making this possible via additions of organometallics directly to unprotected *α*,*β*-unsaturated carboxylic acids would present a unique and important step forward in organic synthesis, but requires circumventing the fundamental issue of the acid–base reactions hindering the desired carbon–carbon bond-forming process.

Here, we describe a strategy that allows highly efficient direct catalytic asymmetric additions of organomagnesium reagents to unprotected *α*,*β*-unsaturated carboxylic acids by preventing the formation of unreactive carboxylate salts. This allows modifications of the carbon chain to proceed unhindered, while the stereochemistry is controlled with a chiral copper catalyst. The catalytic system is scalable, does not require cryogenic temperatures, does not rely on precious metals, and allows the catalyst to be reused.

## Results

### Racemic reaction development

**Fig. 2:**
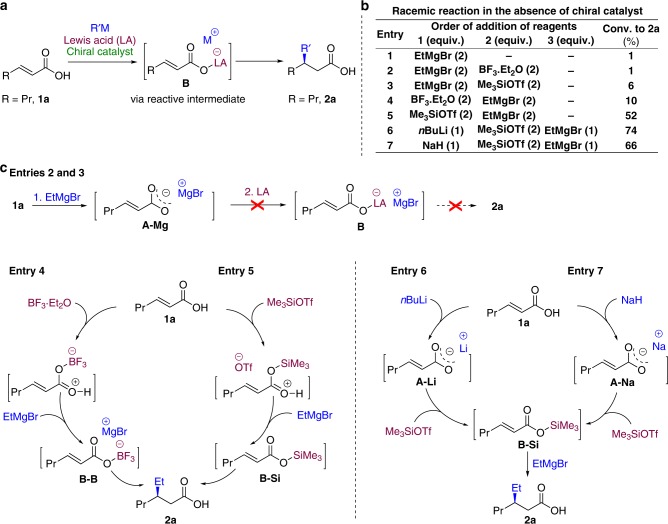
Reaction development. **a** Our approach, based on the use of Lewis acid to promote in situ formation of a transient intermediate **B** that can undergo conjugate addition of organometallic reagent. **b** Conjugate addition of EtMgBr to the substrate **1a** in the absence of chiral catalyst with varying conditions. **c** Rationalization of the experimental data in entries 1–7 obtained for conjugate addition of EtMgBr in various conditions

We realized that the fundamental issue of the acid–base reactions leading to the formation of unreactive carboxylate salts could be circumvented by in situ formation of a reactive intermediate **B** from or instead of the carboxylate salt **A** (Fig. 2a). This would require an intermediate that is reactive toward enantioselective conjugate additions, is easily formed under organometallic addition reaction conditions and would lead to the final carboxylic acid product without additional chemical reactions, simply upon reaction quenching or product isolation^29,30^. We set out to identify a compound that fulfils all of these stringent requirements, drawing on our past experience with combining Lewis acids (LA) and highly reactive organometallics in conjugate additions^28,31,32^. We speculated that using common LA such as trimethylsilyl triflate (Me_3_SiOTf, OTf = OSO_2_CF_3_) or boron trifluoride (BF_3_·Et_2_O) as electrophiles might lead to the formation of soluble and unstable silyl intermediates or boron intermediates, with anticipated high reactivity toward conjugate addition of Grignard reagents and can lead directly to the final unprotected carboxylic acid product. This particular choice of LA was based on our previous studies, where these LA were found to be compatible with Grignard reagents and successfully used to enhance reaction rates with various electrophiles^28,31,32^.

To address the feasibility of this strategy, we first investigated the noncatalyzed addition of EtMgBr to *trans*-2-hexenoic acid **1a** (Fig. 2b). At 0 °C, a complex mixture of products (including 20% of **2a**) was observed in the absence of any catalyst or additives, indicating that the system is dominated by various side reactions at such a relatively high temperature. As expected, no conversion of the substrate **1a** was seen at −55 °C (Fig. 2b, entry 1), because only the Mg-carboxylate **A–Mg** was formed and precipitated out of the reaction mixture (and hydrolyzed back to the substrate during quenching of the reaction). We proceeded by adding the reactive LA Me_3_SiOTf or BF_3_·Et_2_O to the mixture, hoping they would react with the Mg-carboxylate **A–Mg** to form a more reactive boron or silyl intermediate (**B–Si** or **B–B** depending on the Lewis acid used), but unfortunately this only had a minor effect (Fig. 2b, c, entries 2 and 3). Anticipating poor reactivity of the Mg-carboxylate **A–Mg** toward the silyl and boron electrophiles, we decided to add the latter first and EtMgBr second, and to our delight this yielded the addition product **2a** with 10% conversion with BF_3_·Et_2_O and 52% conversion with Me_3_SiOTf (Fig. 2b, entries 4 and 5, respectively). The much higher conversion toward the addition product **2a** obtained with Me_3_SiOTf can be rationalized by the higher electrophilicity of the latter in comparison with BF_3_·Et_2_O.

**Fig. 3:**
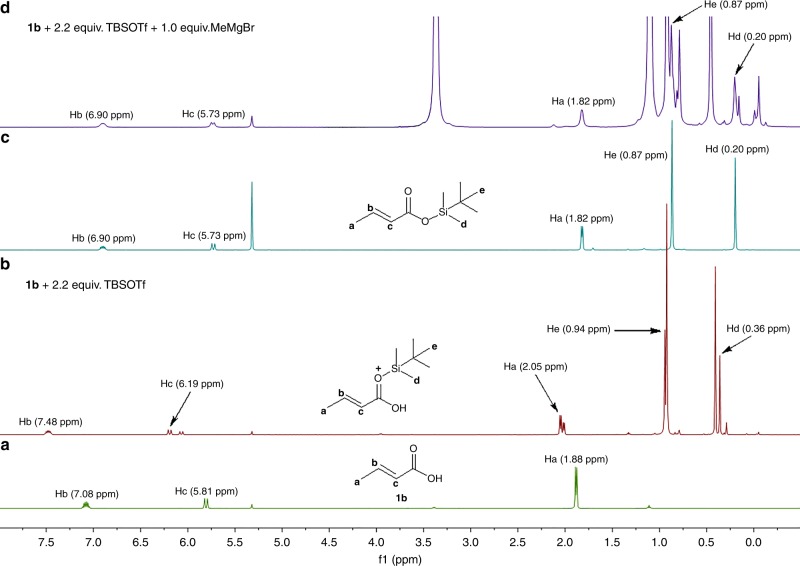
^1^H NMR experiments carried out in CD_2_Cl_2_ at −55 °C using substrate **1b**, *t*BuMe_2_SiOTf and MeMgBr. **a** Crotonic acid **1b**. **b** Mixture of **1b** with 2.2 equiv. of *t*BuMe_2_SiOTf. **c** Isolated pure *t*BuMe_2_Si-ester of **1b**. **d** Mixture of **1b** with 2.2 equiv. of *t*BuMe_2_SiOTf followed by addition of 1.0 equiv. of MeMgBr

We believe that the sequence of addition of the reagents is important, because the nucleophilicity of the Mg-carboxylate **A–Mg** is insufficient to react with the boron or silyl electrophile (presumably due to aggregates formation), and thus nearly no reactive intermediates are generated (Fig. 2b, c, entries 2 and 3). Conversely, when either Lewis acid is added first, it combines with the carboxylic acid **1a** to form an initial complex, after which addition of EtMgBr leads to abstraction of the proton and concomitant formation of intermediates **B–B** or **B–Si** (depending on the Lewis acid used), that are reactive toward conjugate addition (Fig. 2b, c, entries 4 and 5, respectively). Hence, the formation of the Mg-carboxylate **A–Mg** is avoided, and conjugate addition to form the product **2a** can proceed. To get further support for this rationalization, we attempted to observe both the silyl and the boron intermediates derived from Me_3_SiOTf and BF_3_·Et_2_O, respectively, using ^1^H NMR spectroscopy (Fig. 3). However, we failed to do so due to the apparent low stability of the Me_3_Si- and BF_3_ intermediates. To overcome this problem, we decided to switch to a more bulky *t*BuMe_2_Si intermediate, which is expected to be more stable and could derive from *t*BuMe_2_SiOTf. First, we carried out the conjugate addition reaction in the same conditions as those of entry 5 in Fig. 2b, using *t*BuMe_2_SiOTf instead of Me_3_SiOTf. Similar results were obtained in terms of conversion to the addition product, but the product was obtained as a mixture of protected and free carboxylic acid **2a**. We then carried out ^1^H NMR spectroscopic experiments using *t*BuMe_2_SiOTf and crotonic acid **1b** instead of **1a** in order to have simpler ^1^H NMR spectra (Fig. 3). NMR spectroscopic measurements were carried out for four samples derived from (a) crotonic acid **1b**; (b) mixing **1b** with 2.2 equiv. of *t*BuMe_2_SiOTf; (c) synthesized and isolated *t*BuMe_2_Si-ester of **1b**; and (d) mixing **1b** with 2.2 equiv. of *t*BuMe_2_SiOTf followed by addition of 1.0 equiv. of MeMgBr. Upon addition of *t*BuMe_2_SiOTf to **1b**, a new species appeared, corresponding to a complex between the two reagents (Fig. 3b). Addition of 1 equiv. of MeMgBr (just enough to deprotonate but not to be added) led to the formation of the *t*BuMe_2_Si-ester of **1b** with signals comparable with that of independently synthesized and isolated *t*BuMe_2_Si-ester of **1b** (compare Fig. 3c, d). These results confirm indeed that *t*BuMe_2_SiOTf allows in situ protection of **1a**, and that Me_3_SiOTf acts as a traceless protecting group for carboxylic acids and enables the conjugate addition reaction. We assume that BF_3_·Et_2_O works in a similar manner to Me_3_SiOTf. The importance of our strategy of in situ generation of transient intermediates is further highlighted by the difficulties to access pure isolated BF_3_- and TMS-protected unsaturated carboxylic acids. Our attempts to synthesize and isolate these compounds independently (even more bulky and stable silyl esters) resulted in a very low yields (below 20%) and tedious procedures for purification.

To investigate whether carboxylate salts are in general unreactive toward the formation of silyl or boron intermediate or whether this depends on the nature of the metal ion, Li and Na-carboxylates **A–Li** and **A–Na** were prepared from *n*BuLi and NaH, respectively, and subjected to the reaction with Me_3_SiOTf and EtMgBr (Fig. 2b, c, entries 6 and 7). Now the silyl intermediate **B–Si** was formed in both cases, and addition of 1 equiv. of EtMgBr resulted in the formation of the product **2a** with 74 and 66% conversions, respectively (Fig. 2b, c, entries 6 and 7). These results indicate that once the intermediate **B–Si** is formed, conjugate addition works well, but that when the metal carboxylate **A** is formed first, the success of the reaction depends on the counter ions (Fig. 1c). Since metal-carboxylates **A–Mg**, **A–Li**, and **A–Na** are all poorly soluble and precipitate in the reaction solvent, the differing reactivities could also originate from solubility differences. However, we found that the solubility of Li-carboxylate **A–Li** and Na-carboxylate **A–Na** is lower than that of Mg-carboxylate **A–Mg**. More specifically, the solubilities of **A–Mg** and **A–Na** in *t*BuOMe are 0.4318 mM and 0.1225 mM, respectively, while solubility of **A–Li** is under ^1^H NMR detection limit (see Supplementary Figs. 1–3). Thus the difference in reactivity has nothing to do with a better solubility, and can be attributed to a higher nucleophilicity of the Li- and Na-carboxylate most likely. Possible explanation for such a difference in reactivity might be higher aggregation state of the Mg-carboxylate that diminishes its nucleophilicity.

### Development of catalytic asymmetric reaction

Having established that the boron and silyl intermediates **B–B** and, especially, **B–Si** are indeed formed under the right conditions and lead to the racemic product **2a**, we shifted our attention to the question whether this reaction system would be susceptible to asymmetric catalysis in order to both accelerate the conjugate addition toward higher yields and achieve enantiocontrol. As copper is known to be an efficient catalyst for asymmetric conjugate addition reactions^5–7^, we started our investigation by selecting various chiral ligands that can bind to Cu(I). As expected, initial experiments in CH_2_Cl_2_ showed no addition of the highly reactive EtMgBr to **1a** when performing the reaction in the presence of 5 mol% of **L1**/Cu(I)-catalyst at −78 °C. Raising the temperature to 0 °C resulted in 79% conversion with only 20% toward noncatalyzed addition product **2a** and many byproducts (Table 1, entries 1 and 2). At this point, we decided to investigate catalytic reactions in the presence of Me_3_SiOTf (via the formation of the most reactive intermediate **B–Si**) and copper complexes with various chiral diphosphine ligands in CH_2_Cl_2_ at −78 °C. Several chiral catalytic systems result in both acceleration of the conjugate addition and in significant enantiodiscrimination (Table 1, entries 3–7). The superior yield and enantioselectivity obtained with diphosphine Tol-BINAP ligand (*R*)-**L4** (Table 1, entry 6) prompted us to select it as the optimal ligand for this reaction. Subsequently, a thorough optimization process was executed using the catalytic system derived from **L4**/CuBr·SMe_2_ involving the evaluation of various parameters and reaction conditions (for complete set of data see Supplementary Tables 1–5). In particular, the effect of different solvents was studied. With the exception of THF, all solvents tested were effectively tolerated, providing **2a** with good yields and *ee* (Table 1, entries 8–11). We were especially pleased to find exceptionally high yield (95%) and enantioselectivity (92% *ee*) in *t*BuOMe (Table 1, entry 11).

**Table 1:** Development of the catalytic system for direct asymmetric conjugate addition of EtMgBr to carboxylic acid 1a^a^


Entry	L/Cu(I)	LA	Solvent	T [°C]	Conv. [%]^b^	*ee* [%]^c^
1	**L1**/Cu(I)	–	CH_2_Cl_2_	−78	0	–
2	**L1**/Cu(I)	–	CH_2_Cl_2_	0	79^d^	Rac
3	**L1**/Cu(I)	Me_3_SiOTf	CH_2_Cl_2_	−78	74	47
4	**L2**/Cu(I)	Me_3_SiOTf	CH_2_Cl_2_	−78	70	9
5	**L3**/Cu(I)	Me_3_SiOTf	CH_2_Cl_2_	−78	72	47
6	**L4**/Cu(I)	Me_3_SiOTf	CH_2_Cl_2_	−78	87	56
7	**L5**/Cu(I)	Me_3_SiOTf	CH_2_Cl_2_	−78	75	47
8	**L4**/Cu(I)	Me_3_SiOTf	THF	−78	100	Rac
9	**L4**/Cu(I)	Me_3_SiOTf	Toluene	−78	62	80
10	**L4**/Cu(I)	Me_3_SiOTf	Ether	−78	91	88
11	**L4**/Cu(I)	Me_3_SiOTf	*t*BuOMe	−78	95	92
12^e^	**L4**/Cu(I)	*t*BuMe_2_SiOTf	*t*BuOMe	–78	95	95
13	**L4**/Cu(I)	BF_3_·Et_2_O	*t*BuOMe	−78	19	92
14^f^	**L4**/Cu(I)	BF_3_·Et_2_O	*t*BuOMe	−78	77	97
15^f^	**L4**/Cu(I)	Me_3_SiOTf	*t*BuOMe	−78	99	97
16^g^	**L4**/Cu(I)	Me_3_SiOTf	*t*BuOMe	−78	100	95
17	**L4**/Cu(I)	Me_3_SiOTf	*t*BuOMe	0	95	88
18	L4/Cu(I)	Me3SiOTf	tBuOMe	−20	97	97

^a^Reaction conditions: 0.1 M of **1a**, 5 mol% of CuBr·SMe_2_, 6 mol% of **L,** and 2–3 equiv. of LA followed by the addition of 2–3 equiv. of EtMgBr

^b^Conversion was determined by NMR of reaction crude

^c^Enantiomeric excess was determined by chiral HPLC after transforming **2a** to the corresponding *N,N*-dimethyl amide derivative

^d^Less than 20% of **2a** formed with many other byroducts

^e^The product was obtained as a mixture of silyl ester and free carboxylic acid in the ratio of 62:38, respectively

^f^The reaction was performed by first forming Li-carboxylate with *n*BuLi followed by addition of corresponding LA and EtMgBr

^g^The reaction was performed by first forming Na-carboxylate with NaH followed by the additions of Me_3_SiOTf and EtMgBr

In order to connect catalytic asymmetric addition with the results of our ^1^H NMR spectroscopic studies using *t*BuMe_2_SiOTf, we tested the addition of EtMgBr to substrate **1a** using this Lewis acid. As expected, we found that the reaction proceeds with excellent conversion toward the addition product, providing the final product as a mixture of *t*BuMe_2_Si-ester and free carboxylic acid **2a** in a ratio of 62:38, respectively, and high enantioselectivity (95% *ee*, Table 1, entry 12). This composition of the product mixture is not surprising, as under these reaction conditions *t*BuMe_2_Si-ester is expected to be relatively stable.

Catalytic reaction with **L4**/CuBr·SMe_2_ in *t*BuOMe was also investigated using BF_3_·Et_2_O as a Lewis acid. Although high level of enantiomeric purity (92% *ee*) could be obtained in this case, lower reactivity toward conjugate addition of Grignard reagent (only 19% of conversion to **2a**), similar to that observed earlier for racemic reaction was also found in this case (Table 1, entry 13). In contrast, high yield and enantioselectivitiy can be obtained when using BF_3_·Et_2_O in combination with Li-carboxylate (formed by *n*BuLi *in-situ*) followed by addition of EtMgBr (Table 1, entry 14). Similar excellent results were found when using Me_3_SiOTf in combination with Li- and Na-carboxylates (formed by *n*BuLi and NaH, respectively) followed by addition of EtMgBr (Table 1, entries 15 and 16). However, Me_3_SiOTf was selected as the Lewis acid of choice for further studies because of the convenience of a procedure using only one organometallic reagent and highest conversion obtained (Table 1, entry 11). Since a temperature of −78 °C is not practical, particularly for large-scale synthesis, we evaluated the temperature as well (Table 1, entries 17 and 18), finding that −20 °C is the optimal temperature for the reaction with the Grignard reagent, the chiral **L4**/Cu(I) catalyst system, and Me_3_SiOTf. Under these optimized conditions, the reaction is completed in 2 h providing the final product **2a** with 97% of conversion and an *ee* of 97% (Table 1, entry 17).

### Scope of the reaction

**Fig. 4:**
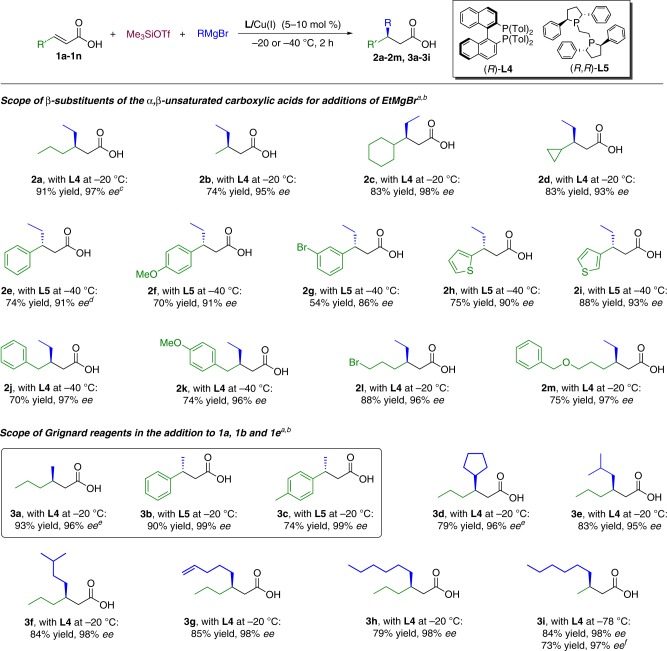
Scope of the substrate and Grignard reagent. ^a^For details see Supplementary Information. Isolated yields for all the products are shown. The absolute configuration of the products obtained with (*R*)-**L4** or (*R*,*R*)-**L5** as the ligands are opposite. ^b^Reaction conditions: 0.1 M of the substrate in *t*BuOMe with 5 mol% (*R*)-**L4**/CuBr·SMe_2_ or in *t*BuOMe/toluene = 1/1 with 10 mol% (*R*,*R*)-**L5**/CuBr·SMe_2_, 2–3 equiv. of Me_3_SiOTf and RMgBr. ^c^Using 5 mol% (*R,R*)-**L5**/CuBr·SMe_2_ as a catalyst in the same condition led to **1a** with 93% *ee*. ^d^Using 5 mol% (*R*)-**L4**/CuBr·SMe_2_ as a catalyst led to **2e** with 57% *ee*. ^e^The reaction performed using 10 mol% of (*R*)-**L4**/CuBr·SMe_2_. ^f^The reaction performed at −40 °C

With the optimized conditions in hand, initial efforts to explore the scope of this transformation focused on investigating the effect of varying the carboxylic acid substitution at the *β*-position (Fig. 4). A wide variety of substrates allow efficient transformation to the corresponding chiral *β*-substituted carboxylic acids. The substrates with linear and branched aliphatic chains (including cyclohexyl and cyclopropyl) gave the corresponding addition products **2a**–**2d** with high yields and excellent enantioselectivities. However, when we applied this condition to the aromatic substrate **1e**, only 57% *ee* was obtained for product **2e**.

Further optimization (see Supplementary Table 5) revealed a different catalytic system to be optimal for aromatic substrates, based on diphosphine ligand (*R*,*R*)-**L5** in combination with copper salt and lower temperatures. Using 10 mol% of **L5**/Cu(I) as the catalyst at −40 °C provided the product **2e** with 91% enantiopurity and 74% isolated yield (Fig. 4).

An aromatic ring with an electron-donating (methoxy, **2** **f**) or electron-withdrawing group (Br, **2** **g**), as well as a heteroaromatic ring (**2** **h**, **2i**), are well tolerated, but including a *m*-Br-substituent in the aromatic ring led to the addition product **2g** with lower yield (54%) and *ee* (86%). When the aromatic ring is at the *γ*-position, the substrates behave as aliphatic substrates, and the highest levels of *ee* and conversion are obtained with the catalyst **L4**/Cu(I) (products **2j** and **2k**). Finally, our catalytic system tolerates the presence of functional groups in the substrate, providing the corresponding products (**2l**, **2m**) with high yields and *ees* above 96%. Next, we examined the nucleophile scope, starting our investigation with the smallest and least reactive of all Grignard reagents, MeMgBr (Fig. 4). Methylations are highly relevant for building chiral polymethylated arrays commonly found in natural products, but they pose difficulties because of the low reactivity of methylating reagents in general^7,26,27^. Currently, asymmetric addition of MeMgBr to *α*,*β*-unsaturated esters is only successful with aliphatic substrates^26,27^.

To our delight, our catalytic system solves this long-standing problem, as it works with similar efficiency for both aliphatic and aromatic substrates, leading to final methylated carboxylic acids with excellent yields and *ees* (**3a**, **3b**, and **3c**). All alkyl Grignard reagents afforded addition products with excellent results, independent of the chain length and branching. The sterically demanding *α-*, *β*-, and *γ*-branched Grignard reagents are tolerated, providing products **3d**–**3f** with high yields and enantioselectivities (*ee*s exceeding 95%). Grignard reagents bearing olefinic substituents also function well, affording the corresponding product **3g** with excellent *ee* and yield. Products **3h** and **3i**, derived from additions of the linear Grignard reagent (*n*HexMgBr) to **1a** and crotonic acid, respectively, were obtained with high enantiopurities and yields as well.

A few important practical aspects of this chemistry deserve to be highlighted (see Supplementary Table 6). First is the possibility to recycle the catalyst, which can be recovered from the reaction mixture with 83% isolated yield in the form of chiral Cu complex and reused in the next reaction with similar performance. Furthermore, the catalyst loading can be decreased from 5 to 1 mol% for addition reactions to aliphatic substrates, and the reaction can be carried out with similar outcome in 1 -g scale of the substrate. Finally, an additional benefit of this catalytic system is that when substrate conversion exceeds 97% most products can be obtained by simple acid–base extraction rather than time consuming column chromatography, which is important for large-scale industrial application^7^.

### Application of the catalytic methodology

**Fig. 5:**
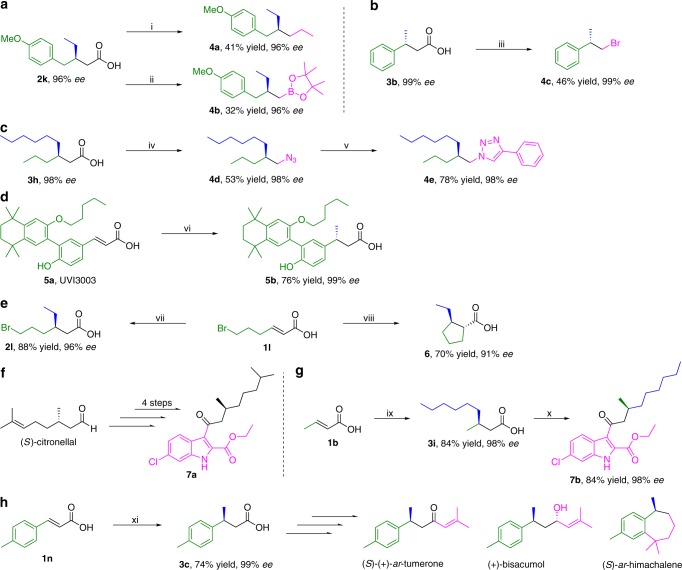
Synthetic utility of the process. **a** Ni-catalyzed decarboxylative alkylation and borylation of chiral acid **2k**. **b** Ag-catalyzed decarboxylative bromination of product **3b**. **c** Ag-catalyzed decarboxylative azidation of chiral acid **3h** followed by click reaction. **d** Late-stage functionalization of a RXR antagonist **5a**. **e** Effect of the different procedures on the structure of the final asymmetric conjugate addition of EtMgBr to the carboxylic acid substrate **1l**. **f** Reported synthetic route to a potent 15-lipoxygenase-1-inhibitor **7a**. **g** Synthesis of the derivative **7b** in two steps using current methodology. **h** Synthesis of chiral acid **3c**, which is a key intermediate of several natural products. i) HATU, NEt_3_, NiCl_2_·glyme, 4,4'-di-t-butyl-2,2'-dipyridyl, ZnEt_2_, in DMF at RT; ii) N-hydroxyphthalimide, DCC, in CH_2_Cl_2_ at RT, 2h, then MgBr_2_·OEt_2_, NiCl_2_·6H_2_O, 4,4'-dimethoxy-2,2'-bipyridyl, [B_2_pin_2_Me]Li, in DMF, THF, at 0 °C 1 h ~ RT 1 h; iii) Ag(Phen)_2_OTf, dibromoisocyanuric acid, in 1,2-dichloroethane at 60 °C; iv) AgF, K_2_S_2_O_8_, MesSO_2_N_3_ in CH_3_CN, H_2_O, at 55 °C; v) phenylacetylene, CuTc, in toluene at RT; vi) CuBr·SMe_2_, (*R,R*)-**L2**, Me_3_SiOTf, MeMgBr, in tBuOMe:Toluene = 1:1, at −20 °C; vii) CuBr·SMe_2_, (*R*)-**L1**, Me_3_SiOTf, EtMgBr, in tBuOMe at −20 °C; viii) CuBr·SMe_2_, (*R*)-**L1**, nBuLi, Me3SiOTf, EtMgBr, in tBuOMe at −78 °C 2 h ~ RT 16 h; ix) CuBr·SMe_2_, (*R*)-**L1**, Me_3_SiOTf, nHexMgBr, in *t*BuOMe at −78 °C; x) SOCl_2_, DMF (1 drop), in CH_2_Cl_2_ at RT, 1 h, then ethyl 6-chloro-1H-indole-2-carboxylate, SnCl_4_, in CH_2_Cl_2_, reflux; xi) CuBr·SMe_2_, (*S,S*)-**L2**, Me_3_SiOTf, MeMgBr, in *t*BuOMe:Toluene = 1:1 at −20 °C

To showcase the potential of our catalytic protocol for future applications, we demonstrate that *β*-chiral substituted carboxylic acids can easily be transformed into a variety of valuable molecules (Fig. 5). The first applications that illustrate utility pertain to the use of our chiral products in stereoselective decarboxylative cross-coupling reactions. Decarboxylative cross-coupling reactions are developing very rapidly, and various catalytic systems utilizing aliphatic carboxylic acids (mainly achiral) leading to diverse structural motives have been established over the past decade^14–19^. Here, we demonstrate how useful chiral analogues of those structural motives can be obtained by combining our methodology and decarboxylative couplings (Fig. 5a–c). Nickel-catalyzed decarboxylative alkylation and borylation of product **2k** afforded chiral alkane **4a** and chiral *β*-substituted boronate ester **4b**, maintaining the original enantiopurity of the starting material through the process (96% *ee*, Fig. 5a)^16,17^. Silver-catalyzed decarboxylative bromination of carboxylic acid **3b** lead to the *β*-chiral alkyl bromide **4c** with an *ee* of 99% (Fig. 5b)^18^, while Ag-catalyzed decarboxylative azidation of carboxylic acid **3h** provided chiral *β*-substituted azide **4d**, which was further transformed into chiral triazole **4e** via click reaction, once again without any racemization (98% *ee*) (Fig. 5c)^19^. Although some similar molecules can be obtained via other catalytic asymmetric methodologies, these are often limited to specific structures and feature varying levels of enantioselectivities^33,34^. For example, chiral *β*-substituted boronate esters can also be obtained through transition metal catalyzed hydroboration of 1,1-disubstituted alkenes. However, these methods are only effective when an aryl group or directing carbonyl groups are present in the substrate. Applying *β*-substituted carboxylic acids obtained by our methodology in decarboxylative borylation offers an attractive alternative for accessing a wide range of chiral aliphatic *β*-substituted boronate esters.

Our methodology is also sufficiently mild and robust to be applied in more complex molecules. For example, UVI3003 **5a**, a full antagonist of RXR (one of the retinoid receptors involved in the control of various physiological and pathological processes, including cancer and metabolic diseases) that demonstrates potent, nanomolar binding affinity^35^, can be functionalized successfully with our strategy without prior protection of the hydroxyl group and afford the product **5b** with 76% yield and 99% *ee* (Fig. 5d). Another synthetically useful transformation available with our methodology is the trapping of enolate intermediates formed upon conjugate addition (Fig. 5e). For instance, while conjugate addition product **2l** can be obtained with 88% yield and 96% *ee*, modifying the original procedure by using Li-carboxylate for asymmetric conjugate addition at −78 °C, followed by warming up to room temperature and stirring overnight, leads to intramolecular enolate trapping, affording the cyclic product **6** with contiguous stereocenters as a single diastereoisomer (70% yield, 91% *ee*). Recently, chiral indole derivative **7a**, synthesized in four steps from the commercially available (*S*)-citronellal, was reported (Fig. 5f) to exhibit in vitro and ex vivo anti-inflammatory properties as a potent 15-lipoxygenase-1 inhibitor^36^. However, as such chiral aldehydes or carboxylic acids are rarely commercially available, synthesis of similar chiral compounds with variations of the alkyl chain is difficult, thus limiting the number of molecules available for bioactivity screening. With our methodology, a library of this type of compounds with different substituents at the *β*-position of the acyl group can be straightforwardly accessed in just two steps, as exemplified by the synthesis of **7b** (Fig. 5g). Finally, our methodology allows us to effortlessly obtain the aromatic chiral *β*-substituted carboxylic acid **3c** (Fig. 5h), which is a key intermediate for the synthesis of several natural products, like (*S*)-( + )-ar-tumerone, ( + )-bisacumol, and (*S*)-ar-himachalene^37^.

## Discussion

We have shown that a wide range of *β*-chiral carboxylic acids are now synthetically accessible from their unsaturated analogues in one simple step and under mild conditions with high yields and enantioselectivities. Our strategy is based on activation of carboxylic acids via formation of transient silyl or boron intermediates, and it is crucial to overcoming the fundamental problem of carboxylate salt formation during the conjugate addition of organometallics to unsaturated carboxylic acid. Thus, this approach allows highly enantioselective catalytic C–C bond-forming reactions between organometallics and carboxylic acids without the use of separate protection/deprotection steps.

## Methods

### General procedure for the catalytic reaction

In a flame-dried Schlenk tube equipped with septum and magnetic stirring bar, the carboxylic acid substrate (0.2 mmol, 1.0 equiv.), CuBr·SMe_2_ (0.01 mmol, 5 mol%), and ligand **L** (0.012 mmol, 6 mol%) were dissolved in the solvent (2.0 mL) and stirred under nitrogen atmosphere for 20 min at RT. The mixture was cooled to −20 or −40 °C, and Me_3_SiOTf (0.44 mmol, 2.2 equiv.) was added. After 20 min, RMgBr (0.5 mmol, 2.5 equiv.) was added dropwise by hand in 10 min (syringe pump use is also an option), and the reaction mixture was allowed to stir for 2 h.

### General work-up procedure A

Upon reaction completion, the mixture was quenched with HCl aqueous solution (2.0 mL, 1.0 M) and warmed up to RT. The resulting mixture was extracted with CH_2_Cl_2_ (10.0 mL × 3). The combined organic phase was dried over MgSO_4_, filtered, and the solvent was evaporated on a rotary evaporator. Pentane (1.0 mL × 3) was added to the residue, and the mixture was filtered through a cotton in a small glass pipette in order to remove the catalyst. The crude was purified by flash chromatography on the silica gel to yield the final conjugate addition product after solvent removal.

### General work-up procedure B

Upon reaction completion, the mixture was quenched with saturated NaHCO_3_ aqueous solution (2.0 mL), warmed up to room temperature, and the organic phase was extracted. The organic phase was further extracted with saturated NaHCO_3_ aqueous solution (2.0 mL) for another two times. The combined aqueous phase was acidified with HCl aqueous solution (1.5 mL, 12.0 M), and extracted with CH_2_Cl_2_ (10.0 mL × 3). The combined organic phase was dried over MgSO_4_, filtered, and the solvent was evaporated on a rotary evaporator to yield the final conjugate addition product.

### Procedure for the preparative-scale reaction

In a flame-dried three-neck round-bottom flask equipped with the septum and mechanistic stirring bar, the substrate **1a** (1.14 g, 10.0 mmol, 1.0 equiv.), CuBr·SMe_2_ (102.8 mg, 0.5 mmol, 5 mol%), and ligand (*R*)-**L4** (407.3 mg, 0.6 mmol, 6 mol%) were dissolved in *t*BuOMe (50 mL), and the mixture was stirred under nitrogen atmosphere for 20 min at RT. The mixture was cooled to −20 °C and Me_3_SiOTf (3.98 mL, 22 mmol, 2.2 equiv.) was added. After 20 min, EtMgBr (8.33 mL, 25 mmol, 2.5 equiv.) was added with the syringe pump in 20 min, and the reaction mixture was allowed to stir for another 2 h at −20 °C. The reaction was quenched with water (10.0 mL) and warmed to RT. The aqueous phase was discarded, and the organic phase was extracted with saturated NaHCO_3_ aqueous solution (50.0 mL × 3). In this step, the chiral catalyst **L4**/Cu(I) remains in the organic phase, while the product **2a** is in the aqueous phase. The organic phase was washed with HCl aqueous solution (10.0 mL, 1.0 M), dried over MgSO_4_, filtered and evaporated on a rotary evaporator. The residue was rinsed with a little amount of pentane, and dried in vacuo overnight to afford the recovered chiral catalyst **L4**/Cu(I) as a light yellow powder [83% yield]. The combined aqueous phase was acidified with HCl aqueous solution (50.0 mL, 12.0 M), and extracted with CH_2_Cl_2_ (100.0 mL × 3). The combined organic phase was dried over MgSO_4_, filtered, and evaporated on a rotary evaporator to yield the product **2a** as a colorless oil [83% yield, 97% *ee*].

## Supplementary information

Supplementary Information

## Data Availability

The authors declare that the data supporting the findings of this study are available within the article and the Supplementary Information, as well as from the authors upon reasonable request. Supplementary Information and chemical compound information are available in the online version of the paper. For the optimization of reaction conditions, see Supplementary Tables 1–5. For practical aspects of the reaction, see Supplementary Table 6. For the experimental details and product characterization, see Supplementary Methods. For the formation, conjugate addition and NMR studies of unsaturated silyl ester, see Supplementary Methods. For the isolation, reactivity and measurement of the solubility of Mg-, Li-, and Na-carboxylates, see Supplementary Methods. For NMR analysis and HPLC traces of the compounds in this article, see Supplementary Figs. 1–75.
