# Supplementary material for: Catalytic enantioselective addition of organometallics to unprotected carboxylic acids
Source: Nat Commun. 2019 Jul 30;10:3402. doi: 10.1038/s41467-019-11345-z (PMC6667444; doi:10.1038/s41467-019-11345-z)
Supplement: Supplementary file 1 — Supplementary Information [file 41467_2019_11345_MOESM1_ESM.pdf]

Supplementary Information

Catalytic enantioselective addition of organometallics to unprotected carboxylic acids

Yan *et al.*

Table of contents

1. Supplementary Tables

2. Supplementary Methods

3. Supplementary Figures

4. Supplementary References

Supplementary Tables

Supplementary Table 1. Conjugate addition of EtMgBr to unsaturated carboxylic acids in the absence of chiral catalyst and in the presence of various bases and silyl electrophiles (investigating the reactivity of different metal carboxylates towards conjugate addition of EtMgBr by changing addition sequence of the reagents).[a]

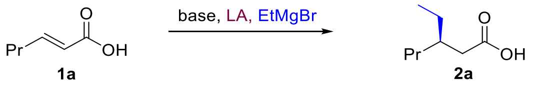

Entry	T [°C]	Addition sequence of reagents[b] 1	2	3	Conv. [%]
1	0	EtMgBr (2.5)	–	–	55[c]
2	−55	EtMgBr (2.5)	–	–	1
3	−55	EtMgBr (2.5)	Me_3SiOTf (2.2)	–	6
4	−55	Me_3SiOTf (2.2)	EtMgBr (2.5)	–	52
5	−55	EtMgBr (1.0)	Me_3SiOTf (2.2)	EtMgBr (1.5)	14
6	−55	*n*BuLi (1.0)	Me_3SiOTf (2.2)	EtMgBr (1.5)	74
7	−55	NaH (1.0)	Me_3SiOTf (2.2)	EtMgBr (1.5)	66
8	−78	EtMgBr (2.5)	–	–	1
9	−78	EtMgBr (2.5)	Me_3SiOTf (2.2)	–	3
10	−78	Me_3SiOTf (2.2)	EtMgBr (2.5)	–	10
11	−78	EtMgBr (1.0)	Me_3SiOTf (2.2)	EtMgBr (1.5)	1
12	−78	*n*BuLi (1.0)	Me_3SiOTf (2.2)	EtMgBr (1.5)	41
13	−78	NaH (1.0)	Me_3SiOTf (2.2)	EtMgBr (1.5)	42

[a]Reaction conditions: 0.1 M of **1a** in *t*BuOMe, overnight. The reaction was quenched with 1.0 M HCl aqueous solution and extracted with CH_2Cl_2, conversion was determined by NMR of reaction crude. [b]Value in brackets corresponds to the equivalents of the reagents used with respect to **1a**. [c]Product **2a** is formed (20%) with the complex mixture of side products.

Supplementary Table 2. Optimization data for the Cu-catalyzed asymmetric conjugate addition of EtMgBr to **1a**[a]

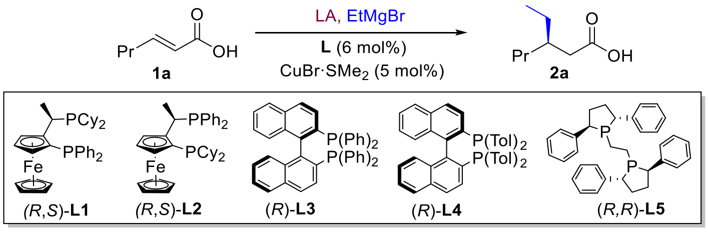

Entry	Cu-**L**	LA	T [°C]	Solvent	Conv. [%][b]	*ee* [%][c]
1	–	–	0	CH_2Cl_2	83[d]	–
2	**L1**	–	0	CH_2Cl_2	79[d]	Rac
3	–	–	−78	CH_2Cl_2	1	–
4	**L1**	–	−78	CH_2Cl_2	1	–
5	–	Me_3SiOTf	−78	CH_2Cl_2	55	–

6	**L1**	Me_3SiOTf	−78	CH_2Cl_2	74	47
7	**L2**	Me_3SiOTf	−78	CH_2Cl_2	70	9
8	**L3**	Me_3SiOTf	−78	CH_2Cl_2	72	47
9	**L4**	Me_3SiOTf	−78	CH_2Cl_2	87	56
10	**L5**	Me_3SiOTf	−78	CH_2Cl_2	75	47
11	**L4**	Me_3SiOTf	−78	THF	100	Rac
12	**L4**	Me_3SiOTf	−78	Toluene	62	80
13	**L4**	Me_3SiOTf	−78	Ether	91	88
14	**L4**	Me_3SiOTf	−78	*t*BuOMe	95	92
15	**L4**	*t*$BuMe_2SiOTf$	−78	*t*BuOMe	95[e]	95
16	**L4**	Me_3SiBr	−78	*t*BuOMe	12	89
17	**L4**	Me_3SiCl	−78	*t*BuOMe	1	–
18	**L4**	$BF_3·Et_2O$	−78	*t*BuOMe	19	92
19	**L4**	BCl_3	−78	*t*BuOMe	17	20
20	**L4**	BBr_3	−78	*t*BuOMe	16	13

[a]Reaction conditions: 0.1 M of **1a**, $CuBr·SMe_2$ (5 mol%), **L** (6 mol%), LA (3.0 equiv.), EtMgBr (3.0 equiv.). [b]The reaction was quenched with 1.0 M HCl aqueous solution and extracted with CH_2Cl_2. Conversion was determined by ^1H NMR of reaction crude. [c]Enantiomeric excess were determined by HPLC on a chiral stationary phase after transforming to the corresponding *N*,*N*-dimethyl amide. [d]Many byproducts formed. [e]Total conversion of the product and the *t*$BuMe_2Si$-protected product

Supplementary Table 3. The reactivity of different metal carboxylates (formed by deptrotonation of **1a** using different bases) towards various Me_3SiX and $BF_3·Et_2O$ in the presence of chiral copper catalyst Cu-**L4**[a]

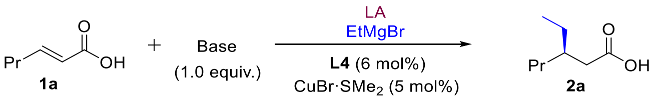

Entry	Base	LA	LA [equiv.]	EtMgBr [equiv.]	T [°C]	Conv. [%][b]	*ee* [%][c]
1	EtMgBr	Me_3SiOTf	2.2	1.5	−78	1	–
2	*n*BuLi	Me_3SiOTf	2.2	1.5	−78	99	97
3	*n*BuLi	Me_3SiOTf	1.2	1.5	−78	99	98
4	NaH	Me_3SiOTf	2.2	1.5	−78	100	95
5	*n*BuLi	Me_3SiBr	1.2	2.5	−78	60	98
6	*n*BuLi	Me_3SiBr	2.2	2.5	−78	83	99
7	*n*BuLi	Me_3SiBr	3.0	2.5	−78	90	99
8	*n*BuLi	$BF_3·Et_2O$	3.0	2.5	−78	77	97
9	*n*BuLi	Me_3SiCl	3.0	2.5	−78	10	–

[a]Reaction conditions: 0.1 M of **1a** in *t*BuOMe, $CuBr·SMe_2$ (5 mol%), **L4** (6 mol%), base (1.0 equiv.). [b]The reaction was quenched with 1.0 M HCl aqueous solution and extracted with CH_2Cl_2, conversion was determined by ^1H NMR of reaction crude. [c]Enantiomeric excess were determined by HPLC on a chiral stationary phase after transforming to the corresponding *N*,*N*-dimethyl amide.

Supplementary Table 4. Optimization of the equivalents of Me_3SiOTf and EtMgBr, and temperature[a]

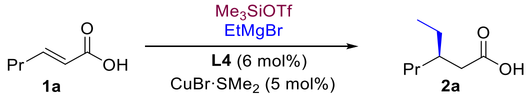

Entry	Me_3SiOTf [equiv.]	EtMgBr [equiv.]	T [°C]	Conv. [%][b]	*ee* [%][c]
1	4.0	4.0	−78	94	80
2	3.0	3.0	−78	95	92
3	3.0	2.2	−78	83	87
4	2.5	2.5	−78	92	97
5	2.2	3.0	−78	94	98
6	2.2	2.5	−78	94	98
7	2.2	2.2	−78	91	97
8	1.5	3.0	−78	88	98
9	1.5	2.2	−78	86	98
10	1.5	1.5	−78	40	–
11	2.2	2.5	0	95	88
12	2.2	2.5	−20	97	97

[a]Reaction conditions: 0.1 M of **1a** in *t*BuOMe, CuBr·SMe_2 (5 mol%), **L1** (6 mol%). [b]The reaction was quenched with 1.0 M HCl aqueous solution and extracted with CH_2Cl_2. Conversion was determined by ^1H NMR of reaction crude. [c]Enantiomeric excess were determined by HPLC on a chiral stationary phase after transforming to the corresponding *N*,*N*-dimethyl amide.

Supplementary Table 5. Optimization data for the Cu-catalyzed asymmetric conjugate addition of EtMgBr to **1e**[a]

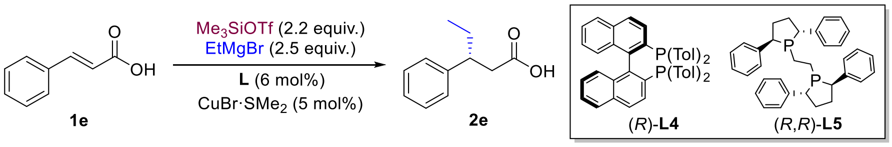

Entry	L	Cu-**L** [mol%]	T [°C]	Solvent [2 mL]	Conv. [%][b]	*ee* [%][c]
1	**L4**	5	−20	*t*BuOMe	94	57
2	**L4**	5	−78	*t*BuOMe	67	87
3	**L5**	5	−78	*t*BuOMe	54	89
4	**L5**	5	−78	CH_2Cl_2	41	72
5	**L5**	5	−78	Toluene	45	90
6	**L5**	5	−20	*t*BuOMe	96	64
7	**L5**	5	−20	*t*BuOMe	65	80[d]
8	**L5**	10	−20	*t*BuOMe	97	80
9	**L5**	10	−40	*t*BuOMe	95	86
10	**L5**	10	−40	Toluene	89	85
11	**L5**	10	−40	*t*BuOMe:Toluene = 1:1[e]	98	91

[a]Reaction conditions: 0.1 M of **1e** in the solvent. [b]The reaction was quenched with 1.0 M HCl aqueous solution and extracted with CH_2Cl_2, conversion was determined by ^1H NMR of reaction crude. [c]Enantiomeric excess were determined by HPLC on a chiral stationary phase after transforming to the corresponding *N*,*N*-dimethyl amide. [d]EtMgBr was diluted with *t*BuOMe to

1.0 mL and slowly added with syringe pump in 1 h. [e]Catalyst **L5**/Cu(I) is not fully soluble in *t*BuOMe, but completely dissolved in toluene. Although the reaction outcome in *t*BuOMe and toluene is similar, *t*BuOMe is a still better solvent.

Supplementary Table 6. Practical aspects of the Cu-catalyzed asymmetric conjugate addition of EtMgBr to **1a**[a]

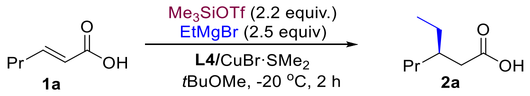

Entry	**1a** [mmol]	*t*BuOMe [mL]	**L4**/Cu(I) [mol%]	Yield [%][b]	*ee* [%][c]
1	0.2	2	1	84	94
2	10	50	5[d]	83	97
3	0.2	2	5[e]	86	95

[a]Reaction conditions: −20 °C, Me_3SiOTf (2.2 equiv.), EtMgBr (2.5 equiv.) for 2 h. [b]Isolated yields for **2a** are shown. Work-up was performed by acid base extraction. [c]Enantiomeric excess were determined by HPLC on a chiral stationary phase after transforming to the corresponding *N*,*N*-dimethyl amide. [d]83% catalyst can be recovered. [e]The reaction was performed with recovered catalyst.

Supplementary methods

General experimental information

All reactions using oxygen- and/or moisture-sensitive materials were carried out with anhydrous solvents under a nitrogen atmosphere using standard Schlenk techniques. Flash column chromatography was performed using Merck 60 Å 230-400 mesh silica gel. Thin layer chromatography was performed using 0.25 mm E. Merck silica plates (60F-254). Unless otherwise indicated, the saturated carboxylic acids were visualized by bromocresol green staining, and other products were visualized by UV and $KMnO_4$ staining. NMR data was collected on Varian VXR400 (^{1}H at 400.0 MHz; ^{13}C at 100.58 MHz) equipped with a 5 mm *z*-gradient broadband probe. Chemical shifts are reported in parts per million (ppm) relative to residual solvent peak ($CDCl_3$, ^{1}H: 7.26 ppm; ^{13}C: 77.16 ppm; DMSO-d_6, ^{1}H: 2.50 ppm; CD_2Cl_2, ^{1}H: 5.32 ppm;). Coupling constants are reported in Hertz. Multiplicity is reported with the usual abbreviations (s: singlet, d: doublet, dd: doublet of doublets, t: triplet, dt: doublet of triplets, q: quartet, dq: doublet of quartets, m: multiplet). Exact mass spectra were recorded on a LTQ Orbitrap XL apparatus with ESI ionization. Enantiomeric excess (*ee*) were determined by chiral HPLC analysis using a Shimadzu LC-10AD*VP* HPLC equipped with a Shimadzu SPD-M10A*VP* diode array detector.

General Chemicals

Unless otherwise indicated, reagents and substrates were purchased from commercial sources and used as received. Unsaturated carboxylic acids **1a-1i**, **1n** are commercially available, the rest were synthesized according to the literature procedures (see below). Solvents not required to be dry were purchased as technical grade and used as received. Dry DMF and THF used for decarboxylative cross-coupling reactions were purchased from Sigma-Aldrich, and the dry THF is inhibitor-free. Other dry solvents were freshly collected from a dry solvent purification system prior to use. Inert atmosphere experiments were performed with standard Schlenk techniques with dried (P_2O_5) nitrogen gas. Pent-4-en-1-ylMgBr (2.0 M in *t*BuOMe) was prepared from the corresponding alkyl bromides and Mg activated with I_2 in *t*BuOMe. Organolithium reagents and Grignard reagents were purchased from Sigma-Aldrich: *n*BuLi (2.5 M in hexane); EtMgBr, MeMgBr (3.0 M in Et_2O); *i*BuMgBr , *i*PentMgBr, *n*HexMgBr, cyclopentylMgBr (2.0 M in Et_2O). Chiral ligands (**L1-L5**) were purchased from Sigma-Aldrich and Solvias. All reported compounds were characterized by ^{1}H and ^{13}C NMR and compared with literature data. All new compounds were fully characterized by ^{1}H and ^{13}C NMR and HRMS techniques.

General determination of absolute configuration and enantiomeric excess of asymmetric conjugate addition products

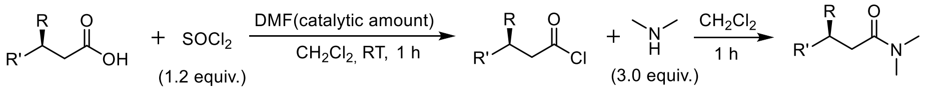

The absolute configuration of **2a-2c**, **2e-2i**, **2l**, **3a**, **3d-3h** was determined by comparing with reported data after transforming to the corresponding *N*,*N*-dimethyl amides.[1] The absolute configurations of other compounds were assigned by analogy. The *ee* of all the products was determined from the corresponding *N*,*N*-dimethyl amide derivatives.

General procedure for the synthesis of *α,β*-unsaturated carboxylic acids

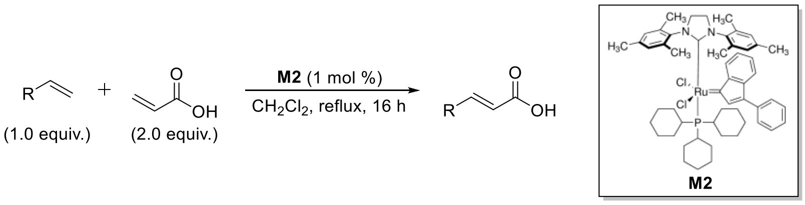

The reactions were performed according to the literature procedure.[2] The corresponding alkene (10.0 mmol, 1.0 equiv.) and acrylic acid (20.0 mmol, 2.0 equiv.) were added simultaneously to a stirred solution of 1 mol% of **M2** catalyst in CH_2Cl_2 (10.0 mL) at room temperature (RT). The reaction was refluxed under nitrogen for 16 h. The solvent and the remaining acrylic acid were removed under reduced pressure and the corresponding *α*,*β*-unsaturated carboxylic acids was purified by column chromatography and rinsed with pentane.

(*E*)-4-Phenylbut-2-enoic acid (1j)[3]

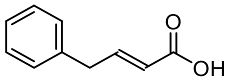

1j

The crude product was purified by column chromatography on silica gel (SiO_2, pentane:Et_2O = 4:1) and rinsed with pentane to afford product **1j** as a white solid [49% yield].

^1H NMR ($CDCl_3$, 400 MHz): δ 7.35-7.29 (m, 2H, CH_{Ar}), 7.28-7.15 (m, 4H, CH_{Ar}, CH_2CH=CH), 5.82 (dt, *J* = 15.5, 1.7 Hz, 1H, CH_2CH=C*H*), 3.55 (dd, *J* = 6.9, 1.6 Hz, 2H, CH_2).

^{13}C NMR ($CDCl_3$, 100 MHz): δ 172.2, 150.4, 137.4, 128.9, 128.8, 126.9, 121.8, 38.67.

(*E*)-4-(4-Methoxyphenyl)but-2-enoic acid (1k)[2]

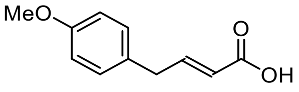

1k

The crude product was purified by column chromatography on silica gel (SiO_2, pentane:Et_2O = 4:1) and rinsed with pentane to afford product **1k** as a white solid [45% yield].

^{1}H NMR ($CDCl_3$, 400 MHz): δ 7.19 (dt, *J* = 15.5, 6.7 Hz, 1H, CH_2CH=CH), 7.11-7.06 (m, 2H, CH_{Ar}), 6.89-6.83 (m, 2H, CH_{Ar}), 5.79 (dt, *J* = 15.5, 1.6 Hz, 1H, CH_2CH=C*H*), 3.80 (s, 3H, CH_3O), 3.49 (dd, *J* = 6.8, 1.6 Hz, 2H, CH_2).

^{13}C NMR ($CDCl_3$, 100 MHz): δ 172.2, 158.6, 150.8, 129.9, 129.4, 121.5, 114.3, 55.4, 37.8.

(*E*)-6-Bromohex-2-enoic acid (1l)

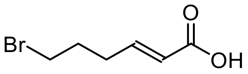

1l

The crude product was purified by column chromatography on silica gel (SiO_2, pentane:Et_2O = 4:1) and rinsed with pentane to afford product **1l** as a white solid [46% yield].

^{1}H NMR ($CDCl_3$, 400 MHz): δ 7.04 (dt, *J* = 15.7, 7.0 Hz, 1H, CH_2CH=CH), 5.89 (dt, *J* = 15.6, 1.6 Hz, 1H, CH_2CH=C*H*), 3.42 (t, *J* = 6.5 Hz, 2H, BrCH_2), 2.42 (m, *J* = 7.2, 1.6 Hz, 2H, CH_2CH=CH), 2.08-2.00 (m, 2H, BrCH_2CH_2).

^{13}C NMR ($CDCl_3$, 100 MHz): δ 172.1, 150.0, 122.0, 32.6, 30.7, 30.6.

HRMS (ESI, m/Z): calcd. for $C_6H_8BrO_2$ $[M-H]^-$: 190.97132, found: 190.97159.

(*E*)-6-(Benzyloxy)hex-2-enoic acid (1m)

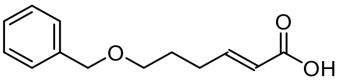

1m

The crude product was purified by column chromatography on silica gel (SiO_2, pentane:Et_2O = 3:1) and rinsed with pentane to afford product **1m** as a white solid [39% yield].

^{1}H NMR ($CDCl_3$, 400 MHz): δ 7.38-7.24 (m, 5H, CH_{Ar}), 7.09 (dt, *J* = 15.6, 7.0 Hz, 1H, CH_2CH=CH), 5.84 (dt, *J* = 15.6, 1.6 Hz, 1H, CH_2CH=C*H*), 4.50 (s, 2H, PhCH_2), 3.50 (t, *J* = 6.2 Hz, 2H, BnOCH_2), 2.38-2.30 (m, 2H, CH_2CH=CH), 1.83-1.74 (m, 2H, BnOCH_2CH_2).

^{13}C NMR ($CDCl_3$, 100 MHz): δ 172.0, 151.6, 138.4, 128.5, 127.8, 127.7, 121.2, 73.1, 69.3, 29.2, 28.1.

HRMS (ESI, m/Z): calcd. for $C_{13}H_{15}O_3$ $[M-H]^-$: 219.10267, found: 219.10319.

General procedure for Cu-catalyzed asymmetric conjugate addition of Grignard reagents to *α,β*-unsaturated carboxylic acids

In a flame-dried Schlenk tube equipped with septum and magnetic stirring bar, the substrate (0.2 mmol, 1.0 equiv.), $CuBr{\cdot}SMe_2$ (0.01 mmol, 5 mol%), and ligand **L** (0.012 mmol, 6 mol%) were dissolved in the solvent (2.0 mL) and stirred under nitrogen atmosphere for 20 min. at RT. The mixture was cooled to −20 or −40 °C and Me_3SiOTf (0.44 mmol, 2.2 equiv.) was added. After 20 min., RMgBr (0.5 mmol, 2.5 equiv.) was added dropwise by hand in 10 min. (syringe pump use is also an option), and the reaction mixture was allowed to stir for 2 h. (Note: Me_3SiOTf should be a new bottle, and dry solvents should be freshly collected from a dry solvent purification system and used immediately. Syringe pump can be used to add Grignards in the big scale reaction, but the Grignards cannot be diluted by the solvent. Otherwise the conversion will decrease.)

General Work-up A

The reaction was quenched with HCl aqueous solution (2.0 mL, 1.0 M) and warmed to RT. The mixture was extracted with CH_2Cl_2 (10.0 mL × 3). The combined organic phase was dried over $MgSO_4$, filtered and evaporated on rotary evaporator. Pentane (1.0 mL × 3) was added to the residue and the mixture was filtered with a small piece of cotton in glass pipette to remove most of the catalyst. The crude was purified by flash chromatography on silica gel.

General Work-up B

The reaction was quenched with saturated $NaHCO_3$ aqueous solution (2.0 mL), warmed to room temperature and the organic phase was extracted. The organic phase was further extracted with saturated $NaHCO_3$ aqueous solution (2.0 mL) for another two times. The combined aqueous phase was acidified with HCl aqueous solution (1.5 mL, 12.0 M), and extracted with CH_2Cl_2 (10.0 mL × 3). The combined organic phase was dried over $MgSO_4$, filtered and evaporated on rotary evaporator.

General Work-up C

The reaction was quenched with saturated $NaHCO_3$ aqueous solution (2.0 mL), warmed to room temperature and the organic phase was extracted. The organic phase was further extracted with saturated Na_2CO_3 aqueous solution (2.0 mL) for another three times. The combined aqueous phase was acidified with HCl aqueous solution (3.0 mL, 12.0 M), and extracted with CH_2Cl_2 (10.0 mL × 3). The combined organic phase was dried over $MgSO_4$, filtered and evaporated on rotary evaporator.

General procedure for the synthesis of racemic conjugate addition products

In a flame-dried Schlenk tube equipped with septum and magnetic stirring bar, the substrate (0.2 mmol, 1.0 equiv.), CuBr·SMe_2 (0.01 mmol, 5 mol%) and THF (2.0 mL) were added. The mixture was cooled to −20 °C and Me_3SiOTf (0.6 mmol, 3.0 equiv.) was added. After 20 min., RMgBr (0.6 mmol, 3.0 equiv.) was added dropwise, and the reaction mixture was allowed to stir for 2 h. The reaction was quenched with HCl aqueous solution (2.0 mL, 1.0 M) and warmed to RT. The mixture was extracted with CH_2Cl_2 (10.0 mL × 3). The combined organic phase was dried over $MgSO_4$, filtered and evaporated on rotary evaporator. The crude was purified by flash column chromatography on silica gel.

Procedure for the preparative scale (10 mmol) Cu-catalyzed asymmetric conjugate addition of EtMgBr to 1a and the recovery of the chiral catalyst L4/Cu

In a flame-dried three-neck round-bottom flask equipped with septum and mechanistic stirring bar, the substrate **1a** (1.14g, 10.0 mmol, 1.0 equiv.), CuBr·SMe_2 (102.8 mg, 0.5 mmol, 5 mol%) and ligand (*R*)-**L4** (407.3 mg, 0.6 mmol, 6 mol%) were dissolved in *t*BuOMe (50 mL) and stirred under nitrogen atmosphere for 20 min. at RT. The mixture was cooled to −20 °C and Me_3SiOTf (3.98 mL, 22 mmol, 2.2 equiv.) was added. After 20 min., EtMgBr (8.33 mL, 25 mmol, 2.5 equiv.) was added with syringe pump in 20 min., and the reaction mixture was allowed to stir for 2 h. The reaction was quenched with water (10.0 mL) and warmed to RT. The aqueous phase was discarded and the organic phase was extracted with saturated $NaHCO_3$ aqueous solution (50.0 mL × 3). In this step, the chiral catalyst **L4**/Cu(I) is in the organic phase while the ACA product **2a** is in the aqueous phase. The organic phase was washed with HCl aqueous solution (10.0 mL, 1.0 M), dried over $MgSO_4$, filtered and evaporated on rotary evaporator. The residue was rinsed with a little pentane and dried *in vacuo* for overnight to afford the recovered chiral catalyst **L4**/Cu(I) as a light yellow powder [83% yield]. The combined aqueous phase was acidified with HCl aqueous solution (50.0 mL, 12.0 M), and extracted with CH_2Cl_2 (100.0 mL × 3). The combined organic phase was dried over $MgSO_4$, filtered and evaporated on rotary evaporator to yield the product **2a** as a colorless oil [83% yield, 97% *ee*] (see Supplementary Table 4).

Troubleshooting and frequently asked questions (FAQs):

Question 1:

Why do I get sometimes lower conversion?

Answer:

This reaction is very sensitive to moisture. Therefore, it is recommended to use a new bottle of Me_3SiOTf, and to collect dry solvents freshly from a dry solvent purification system immediately before use. Also make sure that at every step the reaction is protected under nitrogen and not in contact with the air, otherwise the conversion will decrease.

Question 2:

Why were the Grignard reagents added dropwise by hand in 10 min; can I use a syringe pump instead?

Answer:

Because of the non-catalyzed racemic reaction at high temperature (–20 or –40 ^{o}C), addition of Grignard reagent should be very slow to make sure that the catalytic reaction can outcompete the non-catalyzed racemic reaction, otherwise the *ee* will decrease. While for the large scale reaction (> 1 mmol), syringe pump use is an option, for the small scale reaction the amount of Grignard reagent is so little that it has to be diluted with solvent before adding with a syringe pump. However, we found that if the Grignard is diluted with the solvent, the conversion decreases dramatically (see Supplementary Table 5, entry 7). When the reaction is performed at –78 ^{o}C, addition of Grignard can be faster (added dropwise by hand in 1 min).

Question 3:

Why is the mixture of solvents *t*BuOMe:Toluene = 1:1 required for the aromatic substrates?

Answer:

Catalyst Cu-**L1** is more suitable for aliphatic substrates (**2a**-**2d**, **2j**-**2m**, **3a**, **3d**-**3i**), while catalyst Cu-**L2** is more suitable for aromatic substrates (**2e**-**2i**, **3b**, **3c**). As catalyst Cu-**L1** is soluble in *t*BuOMe, good results can be obtained with *t*BuOMe. In contrast, catalyst Cu-**L2** is less soluble in *t*BuOMe, well soluble in toluene. Although the reaction outcome in *t*BuOMe and toluene is similar, *t*BuOMe is still a better solvent (see Supplementary Table 5, entries 9, 10). Therefore, adding toluene to *t*BuOMe can increase the solubility of Cu-**L2** and give a better reaction outcome (see Supplementary Table 5, entry 11).

Question 3:

Why are there three different methods for reaction work-up, and how to select the best one?

Answer:

The 1,4-addition products can be divided into two groups: the polar products (**2a**, **2b**, **2d**-**2m**, **3a**-**3c**), and the less polar products that have a longer aliphatic chain (**2c**, **3d**-**3i**). For the polar products, separation of the product from the chiral catalyst by column chromatography is difficult because of their similar polarity. However, we can use acid-base extraction to separate them (General Work-up B and C). After acid-base extraction, the products are almost pure, in some cases with small impurities or starting material present. If very pure products are needed, a further column chromatography can be used. To ensure sufficiently pure products, our rule of thumb is that when the conversion is lower than 97% or an obvious impurity is observed, we perform the column chromatography. Otherwise only acid-base extraction suffices to purify the polar products. In contrast, for less polar products the polarity of the product and chiral catalyst are more different. Therefore, column chromatography can be used directly to separate the product and chiral catalyst to obtain pure products (General Work-up A). In addition, because of the low polarity, acid-base extraction does not work well for less polar products.

Question 4:

Why is sometimes $NaHCO_3$ solution and sometimes Na_2CO_3 solution used for acid-base extraction? Can I use NaOH solution instead?

Answer:

Firstly the acid-base extraction only works for more polar products (**2a**, **2b**, **2d**-**2m**, **3a**-**3c**). Again they can be divided into two groups: aliphatic substrates (**2a**, **2b**, **2d**, **2j**-**2m**, **3a**) and aromatic substrates (**2e**-**2i**, **3b**, **3c**). For aliphatic substrates, acid-base extraction with saturated $NaHCO_3$ solution is enough to extract them. Using more alkaline Na_2CO_3 solution or NaOH solution sometimes will extract more impurities. Aromatic substrates are more difficult to extract due to their higher solubility in toluene. Therefore, more alkaline Na_2CO_3 should be used to make sure that all the product will go to the aqueous phase. Using 1.0 M NaOH solution is an option in both cases, but this can result in more impurities going the

aqueous phase. In addition, the NaOH solution will destroy part of the catalyst, so it can only be used when the catalyst does not need to be recovered.

Question 5:

Why was the reaction quenched with $NaHCO_3$ solution in General Work-up C, while the acid-base extraction was performed with Na_2CO_3 solution?

Answer:

We found that when the reaction is quenched with Na_2CO_3 solution, a lot of salt precipitates, making extraction more difficult. Therefore, less alkaline $NaHCO_3$ solution should be used to quench the reaction to avoid precipitation. For all the reactions, if the precipitate still appears when the reaction is quenched with $NaHCO_3$ solution (this is more likely to occur in big scale reactions), water can also be used to quench the reaction.

Question 6:

Why do I get high conversion but very low yield?

Answer:

There are two possibilities: (1) for some substrates, such as crotonic acid, a lot of byproducts will be formed at high temperature because of its high reactivity. In this case, decreasing the reaction temperature will be helpful; (2) the products can easily get stuck in the silica gel when purified by column chromatography. Therefore, a very short column and eluents with relatively high polarity are recommended.

Question 7:

How can I optimize the reaction if my reaction outcome is not good enough?

Answer:

Increasing the amount of catalyst will lead to an increase of both the conversion and *ee*. Increasing the temperature or the amount of Lewis acid will lead to an increase of the conversion but a decrease of the *ee* (and *vice versa*). Different combinations of these factors can be tried to optimize the reaction outcome.

Specific experimental details and product characterization

(*R*)-3-Ethylhexanoic acid (2a)[4]

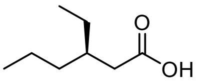

2a

The reaction was performed with **1a** (22.8 mg, 0.2 mmol, 1.0 equiv.), $CuBr{\cdot}SMe_2$ (2.06 mg, 0.01 mmol, 5 mol%), ligand (*R*)-**L4** (8.15 mg, 0.012 mmol, 6 mol%), Me_3SiOTf (80 μL, 0.44 mmol, 2.2 equiv), EtMgBr (0.5 mmol, 3.0 M in Et_2O, 2.5 equiv.), *t*BuOMe (2.0 mL) at −20 °C, and following General Work-up **B**. Product **2a** was obtained as a colorless oil without further purification [97% conversion, 91% yield, 97% *ee*].

^{1}H NMR ($CDCl_3$, 400 MHz): δ 2.28 (d, *J* = 6.9 Hz, 2H, CH_2CO_2H), 1.88-1.77 (m, 1H, C*H*), 1.45-1.22 (m, 6H, CH_2), 0.93-0.85 (m, 6*H*, CH_3).

^{13}C NMR ($CDCl_3$, 100 MHz): δ 180.4, 38.7, 36.2, 35.8, 26.4, 19.8, 14.4, 10.9.

The *ee* of this compound was determined from the corresponding *N*,*N*-dimethyl amide derivative. HPLC: Chiracel-OBH, *n*heptane/*i*PrOH 98:2, 0.5 mL/min., 40 °C, detection at 206 nm. Retention time (min): 14.7 (minor) and 16.7 (major).

(*S*)-3-Methylpentanoic acid (2b)[4]

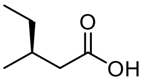

2b

The reaction was performed with **1b** (17.2 mg, 0.2 mmol, 1.0 equiv.), $CuBr{\cdot}SMe_2$ (2.06 mg, 0.01 mmol, 5 mol%), ligand (*R*)-**L4** (8.15 mg, 0.012 mmol, 6 mol%), Me_3SiOTf (80 μL, 0.44 mmol, 2.2 equiv), EtMgBr (0.5 mmol, 3.0 M in Et_2O, 2.5 equiv.), *t*BuOMe (2.0 mL) at −20 °C, and following General Work-up **B**. Product **2b** was obtained as a colorless oil after column chromatography (SiO_2, pentane:Et_2O = 5:1) [98% conversion, 74% yield, 95% *ee*].

^{1}H NMR ($CDCl_3$, 400 MHz): δ 2.36 (dd, *J* = 15.0, 6.0 Hz, 1H, C*H*HCO_2H), 2.15 (dd, *J* = 15.0, 8.1 Hz, 1H, CH*H*CO_2H), 1.96-1.82 (m, 1H, C*H*), 1.46-1.33 (m, 1H, CH_3C*H*H), 1.32-1.19 (m, 1H, CH_3CH*H*), 0.97 (d, *J* = 6.7 Hz, 3H, CH_3CH), 0.90 (t, *J* = 7.4 Hz, 3H, CH_3CH_2).

^{13}C NMR ($CDCl_3$, 100 MHz): δ 180.1, 41.4, 31.9, 29.4, 19.4, 11.4.

The *ee* of this compound was determined from the corresponding *N*,*N*-dimethyl amide derivate. HPLC: Chiracel-OBH, *n*heptane/*i*PrOH 90:10, 0.5 mL/min., 40 °C, detection at 206 nm. Retention time (min): 10.7 (major) and 13.1 (minor).

(*S*)-3-Cyclohexylpentanoic acid (2c)[5]

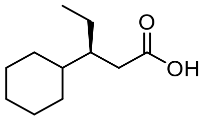

2c

The reaction was performed with **1c** (30.8 mg, 0.2 mmol, 1.0 equiv.), $CuBr{\cdot}SMe_2$ (2.06 mg, 0.01 mmol, 5 mol%), ligand (*R*)-**L4** (8.15 mg, 0.012 mmol, 6 mol%), Me_3SiOTf (80 µL, 0.44 mmol, 2.2 equiv), EtMgBr (0.5 mmol, 3.0 M in Et_2O, 2.5 equiv.), *t*BuOMe (2.0 mL) at −20 °C, and following General Work-up **A**. Product **2c** was obtained as a colorless oil after column chromatography (SiO_2, pentane:Et_2O = 15:1) [98% conversion, 83% yield, 98% *ee*].

^{1}H NMR ($CDCl_3$, 400 MHz): δ 2.36 (dd, *J* = 15.4, 6.0 Hz, 1H, C*H*HCO_2H), 2.19 (dd, *J* = 15.4, 7.6 Hz, 1H, CH*H*CO_2H), 1.79-1.56 (m, 6H, CH_2, C*H*), 1.49-0.93 (m, 8H, CH_2), 0.89 (t, *J* = 7.4 Hz, 3H, CH_3).

^{13}C NMR ($CDCl_3$, 100 MHz): δ 181.0, 42.0, 40.2, 36.1, 30.2, 29.3, 26.9, 26.9, 26.8, 24.0, 11.8.

The *ee* of this compound was determined from the corresponding *N*,*N*-dimethyl amide derivative. HPLC: Chiracel-OZH, *n*heptane/*i*PrOH 95:5, 0.5 mL/min., 40 °C, detection at 207 nm. Retention time (min): 17.0 (major) and 18.4 (minor).

(*S*)-3-Cyclopropylpentanoic acid (2d)

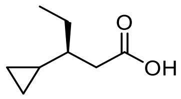

2d

The reaction was performed with **1d** (22.4 mg, 0.2 mmol, 1.0 equiv.), $CuBr{\cdot}SMe_2$ (2.06 mg, 0.01 mmol, 5 mol%), ligand (*R*)-**L4** (8.15 mg, 0.012 mmol, 6 mol%), Me_3SiOTf (80 µL, 0.44 mmol, 2.2 equiv), EtMgBr (0.5 mmol, 3.0 M in Et_2O, 2.5 equiv.), *t*BuOMe (2.0 mL) at −20 °C, and following General Work-up **B**. Product **2d** was obtained as a colorless oil without further purification [99% conversion, 83% yield, 93% *ee*].

^{1}H NMR ($CDCl_3$, 400 MHz): δ 2.42 (d, *J* = 7.0 Hz, 2H, CH_2CO_2H), 2.38 (dd, *J* = 14.5, 6.9 Hz, 1H, CH*H*CO_2H), 1.55-1.46 (m, 2H, CH_3CH_2), 1.11-1.00 (m, 1H, CH_3CH_2CH), 0.96 (t, *J* = 7.5 Hz, 3H, CH_3), 0.65-0.55 (m, 1H, CH_{Cy}), 0.53-0.45 (m, 1H, C*H*H_{Cy}), 0.45-0.38 (m, 1H, CHH_{Cy}), 0.20-0.08 (m, 2H, CH_{Cy2}).

^{13}C NMR ($CDCl_3$, 100 MHz): δ 180.3, 42.5, 39.8, 28.0, 15.9, 11.4, 4.7, 3.4.

HRMS (ESI, m/Z): calcd. for $C_8H_{15}O_2$ $[M+H]^+$: 143.10666, found: 143.10748.

The *ee* of this compound was determined from the corresponding *N,N*-dimethyl amide derivative. HPLC: Chiracel-OBH, *n*heptane/*i*PrOH 98:2, 0.5 mL/min., 40 °C, detection at 207 nm. Retention time (min): 16.9 (minor) and 18.8 (major).

(*R*)-3-Phenylpentanoic acid (2e)[6]

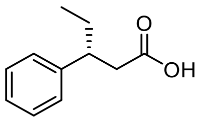

2e

The reaction was performed with **1e** (29.6 mg, 0.2 mmol, 1.0 equiv.), CuBr·SMe_2 (4.12 mg, 0.02 mmol, 10 mol%), ligand (*R*,*R*)-**L5** (12.16 mg, 0.024 mmol, 12 mol%), Me_3SiOTf (80 μL, 0.44 mmol, 2.2 equiv), EtMgBr (0.5 mmol, 3.0 M in Et_2O, 2.5 equiv.), *t*BuOMe (1.0 mL), Toluene (1.0 mL) at −40 °C, and following General Work-up **C**. Product **2e** was obtained as a colorless oil after column chromatography (SiO_2, pentane:Et_2O = 5:1) [98% conversion, 74% yield, 91% *ee*].

^1H NMR ($CDCl_3$, 400 MHz): δ 7.33-7.27 (m, 2H, CH_{Ar}), 7.24-7.16 (m, 3H, CH_{Ar}), 3.05-2.95 (m, 1H, C*H*), 2.68 (dd, *J* = 15.6, 7.1 Hz, 1H, C*H*HCO_2H), 2.61 (dd, *J* = 15.6, 7.9 Hz, 1H, CH*H*CO_2H), 1.80-1.68 (m, 1H, CH_3C*H*H), 1.68-1.55 (m, 1H, CH_3CH*H*), 0.80 (t, *J* = 7.4 Hz, 3H, CH_3).

^{13}C NMR ($CDCl_3$, 100 MHz): δ 178.8, 143.7, 128.6, 127.6, 126.6, 43.6, 41.2, 29.3, 12.0.

The *ee* of this compound was determined from the corresponding *N,N*-dimethyl amide derivative. HPLC: Chiracel-ODH, *n*heptane/*i*PrOH 90:10, 0.5 mL/min., 40 °C, detection at 208 nm. Retention time (min): 12.1 (minor) and 13.2 (major).

(*R*)-3-(4-Methoxyphenyl)pentanoic acid (2f)

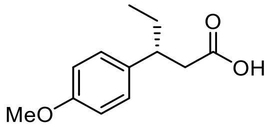

2f

The reaction was performed with **1f** (35.6 mg, 0.2 mmol, 1.0 equiv.), $CuBr·SMe_2$ (4.12 mg, 0.02 mmol, 10 mol%), ligand (*R*,*R*)-**L5** (12.16 mg, 0.024 mmol, 12 mol%), Me_3SiOTf (80 μL, 0.44 mmol, 2.2 equiv), EtMgBr (0.5 mmol, 3.0 M in Et_2O, 2.5 equiv.), *t*BuOMe (1.0 mL), Toluene (1.0 mL) at −40 °C, and following General Work-up **C**. Product **2f** was obtained as a white solid after column chromatography (SiO_2, pentane:Et_2O = 5:1) [95% conversion, 70% yield, 91% *ee*].

^{1}H NMR ($CDCl_3$, 400 MHz): δ 7.13-7.07 (m, 2H, CH_{Ar}), 6.87-6.81 (m, 2H, CH_{Ar}), 3.79 (s, 3H, CH_3O), 3.00-2.89 (m, 1H, C*H*), 2.64 (dd, *J* = 15.5, 7.0 Hz, 1H, C*H*HCO$_2$H), 2.57 (dd, *J* = 15.5, 8.1 Hz, 1H, CH*H*CO$_2$H), 1.77-1.65 (m, 1H, CH$_3$C*H*H), 1.64-1.50 (m, 1H, CH$_3$CH*H*), 0.78 (t, *J* = 7.3 Hz, 3H, CH_3).

^{13}C NMR ($CDCl_3$, 100 MHz): δ 178.9, 158.3, 135.8, 128.5, 113.9, 55.3, 42.9, 41.5, 29.4, 12.0.

HRMS (ESI, m/Z): calcd. for $C_{12}H_{17}O_3$ $[M+H]^+$: 209.11722, found: 209.11821.

The *ee* of this compound was determined from the corresponding *N*,*N*-dimethyl amide derivate. HPLC: Chiracel-OJH, *n*heptane/*i*PrOH 90:10, 0.5 mL/min., 40 °C, detection at 204 nm. Retention time (min): 14.1 (minor) and 16.9 (major).

(*R*)-3-(3-Bromophenyl)pentanoic acid (2g)

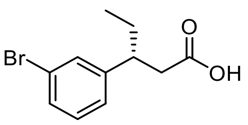

2g

The reaction was performed with **1g** (45.4 mg, 0.2 mmol, 1.0 equiv.), $CuBr·SMe_2$ (4.12 mg, 0.02 mmol, 10 mol%), ligand (*R*,*R*)-**L5** (12.16 mg, 0.024 mmol, 12 mol%), Me3SiOTf (80 μL, 0.44 mmol, 2.2 equiv), EtMgBr (0.5 mmol, 3.0 M in Et_2O, 2.5 equiv.), *t*BuOMe (1.0 mL), Toluene (1.0 mL) at −40 °C, and following General Work-up **C**. Product **2g** was obtained as a colorless oil after column chromatography (SiO_2, pentane:Et_2O = 5:1) [97% conversion, 54% yield, 86% *ee*].

^{1}H NMR ($CDCl_3$, 400 MHz): δ 7.37-7.31 (m, 2H, CH_{Ar}), 7.20-7.08 (m, 2H, CH_{Ar}), 2.96 (m, *J* = 7.4 Hz, 1H, C*H*), 2.66 (dd, *J* = 15.8, 6.9 Hz, 1H, C*H*HCO$_2$H), 2.58 (dd, *J* = 15.8, 8.0 Hz, 1H, CH*H*CO$_2$H), 1.79-1.66 (m, 1H, CH$_3$C*H*H), 1.66-1.52 (m, 1H, CH$_3$CH*H*), 0.79 (t, *J* = 7.3 Hz, 3H, CH_3).

^{13}C NMR ($CDCl_3$, 100 MHz): δ 178.4, 146.2, 130.7, 130.2, 129.9, 126.4, 122.7, 43.4, 41.0, 29.2, 12.0.

HRMS (ESI, m/Z): calcd. for $C_{11}H_{12}BrO_2$ $[M-H]^-$: 255.00262, found: 255.00297.

The *ee* of this compound was determined from the corresponding *N*,*N*-dimethyl amide derivative. HPLC: Chiracel-ODH, *n*heptane/*i*PrOH 97:3, 0.5 mL/min., 40 °C, detection at 205 nm. Retention time (min): 22.2 (minor) and 25.9 (major).

(*R*)-3-(Thiophen-2-yl)pentanoic acid (2h)

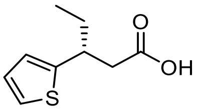

2h

The reaction was performed with **1h** (30.8 mg, 0.2 mmol, 1.0 equiv.), CuBr·SMe_2 (4.12 mg, 0.02 mmol, 10 mol%), ligand (*R*,*R*)-**L5** (12.16 mg, 0.024 mmol, 12 mol%), Me_3SiOTf (80 µL, 0.44 mmol, 2.2 equiv), EtMgBr (0.5 mmol, 3.0 M in Et_2O, 2.5 equiv.), *t*BuOMe (1.0 mL), Toluene (1.0 mL) at −40 °C, and following General Work-up **C**. Product **2h** was obtained as a colorless oil after column chromatography (SiO_2, pentane:Et_2O = 5:1) [95% conversion, 75% yield, 90% *ee*].

^1H NMR ($CDCl_3$, 400 MHz): δ 7.16 (dd, *J* = 5.2, 1.2 Hz, 1H, CH_{Ar}), 6.92 (dd, *J* = 5.1, 3.4 Hz, 1H, CH_{Ar}), 6.86-6.82 (m, 1H, CH_{Ar}), 3.40-3.31 (m, 1H, C*H*), 2.71 (dd, *J* = 15.7, 7.0 Hz, 1H, C*H*HCO_2H), 2.66 (dd, *J* = 15.7, 7.7 Hz, 1H, CH*H*CO_2H), 1.85-1.72 (m, 1H, CH_3C*H*H), 1.72-1.59 (m, 1H, CH_3CH*H*), 0.88 (t, *J* = 7.3 Hz, 3H, CH_3).

^{13}C NMR ($CDCl_3$, 100 MHz): δ 178.4, 147.5, 126.7, 124.2, 123.4, 42.1, 39.0, 30.3, 11.8.

HRMS (ESI, m/Z): calcd. for $C_9H_{13}O_2S$ $[M+H]^+$: 185.06308, found: 185.06390.

The *ee* of this compound was determined from the corresponding *N*,*N*-dimethyl amide derivative. HPLC: Chiracel-ADH, *n*heptane/*i*PrOH 90:10, 0.5 mL/min., 40 °C, detection at 240 nm. Retention time (min): 11.7 (minor) and 13.2 (major).

(*R*)-3-(Thiophen-3-yl)pentanoic acid (2i)

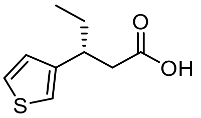

2i

The reaction was performed with **1i** (30.8 mg, 0.2 mmol, 1.0 equiv.), CuBr·SMe_2 (4.12 mg, 0.02 mmol, 10 mol%), ligand (*R*,*R*)-**L5** (12.16 mg, 0.024 mmol, 12 mol%), Me_3SiOTf (80 µL, 0.44 mmol, 2.2 equiv), EtMgBr (0.5 mmol, 3.0 M in Et_2O, 2.5 equiv.), *t*BuOMe (1.0 mL), Toluene (1.0 mL) at −40 °C, and following General Work-up **C**. Product **2i** was obtained as a colorless oil without further purification [97% conversion, 88% yield, 93% *ee*].

^1H NMR ($CDCl_3$, 400 MHz): δ 7.26 (dd, *J* = 5.1, 2.9 Hz, 1H, CH_{Ar}), 6.99 (dd, *J* = 3.0, 1.3 Hz, 1H, CH_{Ar}), 6.95 (dd, *J* = 5.0, 1.3 Hz, 1H, CH_{Ar}), 3.22-3.12 (m, 1H, C*H*), 2.66 (dd, *J* = 15.5, 7.0 Hz, 1H, C*H*HCO_2H), 2.59 (dd, *J* = 15.5, 7.9 Hz, 1H, CH*H*CO_2H), 1.72 (m, *J* = 15.1, 7.5, 5.6 Hz, 1H, CH_3C*H*H), 1.68-1.54 (m, 1H, CH_3CH*H*), 0.83 (t, *J* = 7.4 Hz, 3H, CH_3).

^{13}C NMR ($CDCl_3$, 100 MHz): δ 179.0, 144.5, 126.7, 125.7, 120.6, 41.0, 38.9, 29.0, 11.9.

HRMS (ESI, m/Z): calcd. for $C_9H_{13}O_2S$ $[M+H]^+$: 185.06308, found: 185.06383.

The *ee* of this compound was determined from the corresponding *N,N*-dimethyl amide derivative. HPLC: Chiracel-OBH, *n*heptane/*i*PrOH 90:10, 0.5 mL/min., 40 °C, detection at 206 nm. Retention time (min): 14.3 (minor) and 17.4 (major).

(*R*)-3-Benzylpentanoic acid (2j)[7]

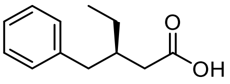

2j

The reaction was performed with **1j** (32.4 mg, 0.2 mmol, 1.0 equiv.), $CuBr·SMe_2$ (2.06 mg, 0.01 mmol, 5 mol%), ligand (*R*)-**L4** (8.15 mg, 0.012 mmol, 6 mol%), Me_3SiOTf (80 μL, 0.44 mmol, 2.2 equiv), EtMgBr (0.5 mmol, 3.0 M in Et_2O, 2.5 equiv.), *t*BuOMe (2.0 mL) at −40 °C, and following General Work-up **B**. Product **2j** was obtained as a colorless oil after column chromatography (SiO_2, pentane:Et_2O = 10:1) [99% conversion, 70% yield, 97% *ee*].

^1H NMR ($CDCl_3$, 400 MHz): δ 7.33-7.25 (m, 2H, CH_{Ar}), 7.23-7.15 (m, 3H, CH_{Ar}), 2.69 (dd, *J* = 13.6, 6.8 Hz, 1H, C*H*HCO$_2$H), 2.58 (dd, *J* = 13.6, 7.4 Hz, 1H, CH*H*CO$_2$H), 2.31 (dd, *J* = 15.7, 7.2 Hz, 1H, PhC*H*H), 2.27 (dd, *J* = 15.7, 6.4 Hz, 1H, PhCH*H*), 2.14 (m, *J* = 6.7 Hz, 1H, C*H*), 1.49-1.31 (m, 2H, CH_3CH_2), 0.95 (t, *J* = 7.4 Hz, 3H, CH_3).

^{13}C NMR ($CDCl_3$, 100 MHz): δ 179.9, 140.3, 129.4, 128.4, 126.2, 40.0, 38.5, 37.9, 26.3, 11.1.

The *ee* of this compound was determined from the corresponding *N,N*-dimethyl amide derivative. HPLC: Chiracel-ODH, *n*heptane/*i*PrOH 95:5, 0.5 mL/min., 40 °C, detection at 208 nm. Retention time (min): 16.6 (minor) and 22.2 (major).

(*R*)-3-(4-Methoxybenzyl)pentanoic acid (2k)

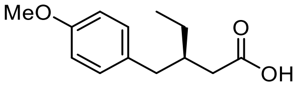

2k

The reaction was performed with **1k** (192.2 mg, 1.0 mmol, 1.0 equiv.), $CuBr{\cdot}SMe_2$ (10.3 mg, 0.05 mmol, 5 mol%), ligand (*R*)-**L4** (40.7 mg, 0.06 mmol, 6 mol%), Me_3SiOTf (398 μL, 2.2 mmol, 2.2 equiv), *t*BuOMe (10.0 mL) at −40 °C, and EtMgBr (2.5 mmol, 3.0 M in Et_2O, 2.5 equiv.) was added with syringe pump in 10 min. The reaction was quenched with saturated $NaHCO_3$ aqueous solution (10.0 mL), warmed to RT. and the organic phase was extracted. The organic phase was further extracted with saturated $NaHCO_3$ aqueous solution (10.0 mL × 3) for another two times. The combined aqueous phase was acidified with HCl aqueous solution (7.5 mL, 12.0 M), and extracted with CH_2Cl_2 (30.0 mL × 3). The combined organic phase was dried over $MgSO_4$, filtered and evaporated on rotary evaporator. Product **2k** was obtained as a colorless oil without further purification [98% conversion, 74% yield, 96% *ee*].

^1H NMR ($CDCl_3$, 400 MHz): δ 7.12-7.05 (m, 2H, CH_{Ar}), 6.86-6.79 (m, 2H, CH_{Ar}), 3.78 (s, 3H, CH_3O), 2.63 (dd, *J* = 13.7, 6.8 Hz, 1H, C*H*HCO_2H), 2.51 (dd, *J* = 13.8, 7.4 Hz, 1H, CH*H*CO_2H), 2.27 (d, *J* = 6.8 Hz, 2H, PhCH_2), 2.08 (m, *J* = 6.7 Hz, 1H, C*H*), 1.48-1.29 (m, 2H, CH_3CH_2), 0.94 (t, *J* = 7.4 Hz, 3H, CH_3CH_2).

^{13}C NMR ($CDCl_3$, 100 MHz): δ 180.2, 158.1, 132.3, 130.3, 113.8, 55.3, 39.0, 38.7, 37.9, 26.2, 11.1.

HRMS (ESI, m/Z): calcd. for $C_{13}H_{17}O_3$ $[M-H]^-$: 221.11832, found: 221.11842.

The *ee* of this compound was determined from the corresponding *N,N*-dimethyl amide derivative. HPLC: Chiracel-OZH, *n*heptane/*i*PrOH 95:5, 0.5 mL/min., 40 °C, detection at 204 nm. Retention time (min): 35.2 (minor) and 39.9 (major).

(*R*)-6-Bromo-3-ethylhexanoic acid (2l)

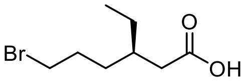

2l

The reaction was performed with **1l** (38.6 mg, 0.2 mmol, 1.0 equiv.), $CuBr{\cdot}SMe_2$ (2.06 mg, 0.01 mmol, 5 mol%), ligand (*R*)-**L4** (8.15 mg, 0.012 mmol, 6 mol%), Me_3SiOTf (80 μL, 0.44 mmol, 2.2 equiv), EtMgBr (0.5 mmol, 3.0 M in Et_2O, 2.5 equiv.), *t*BuOMe (2.0 mL) at −20 °C, and following General Work-up **B**. Product **2l** was obtained as a colorless without further purification [97% conversion, 88% yield, 96% *ee*].

^1H NMR ($CDCl_3$, 400 MHz): δ 3.40 (t, *J* = 6.8 Hz, 2H, BrCH_2), 2.33 (dd, *J* = 15.3, 6.8 Hz, 1H, C*H*HCO_2H), 2.27 (dd, *J* = 13.8, 6.9 Hz, 1H, CH*H*CO_2H), 1.92-1.79 (m, 3H, $BrCH_2CH_2$, C*H*), 1.56-1.30 (m, 4H, $BrCH_2CH_2CH_2$, CH_3CH_2), 0.90 (t, *J* = 7.4 Hz, 3H, CH_3).

^{13}C NMR ($CDCl_3$, 100 MHz): δ 179.9, 38.5. 35.8, 33.9, 32.0, 30.1, 26.3, 10.8.

HRMS was measured after transforming to the corresponding *N,N*-dimethyl amide (ESI, m/Z): calcd. for $C_{10}H_{21}NOBr$ $[M+H]^+$: 250.08010, found: 250.08049.

The *ee* of this compound was determined from the corresponding *N,N*-dimethyl amide derivate. HPLC: Chiracel-ODH, *n*heptane/*i*PrOH 95:5, 0.5 mL/min., 40 °C, detection at 208 nm. Retention time (min): 15.6 (major) and 17.2 (minor).

(*R*)-6-Benzyloxy-3-ethylhexanoic acid (2m)

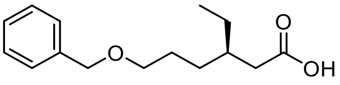

2m

The reaction was performed with **1m** (44.1 mg, 0.2 mmol, 1.0 equiv.), $CuBr{\cdot}SMe_2$ (2.06 mg, 0.01 mmol, 5 mol%), ligand (*R*)-**L4** (8.15 mg, 0.012 mmol, 6 mol%), Me_3SiOTf (80 µL, 0.44 mmol, 2.2 equiv), EtMgBr (0.5 mmol, 3.0 M in Et_2O, 2.5 equiv.), *t*BuOMe (2.0 mL) at −20 °C, and following General Work-up **B**. Product **2m** was obtained as a colorless oil without further purification [97% conversion, 75% yield, 97% *ee*].

^1H NMR ($CDCl_3$, 400 MHz): δ 7.38-7.25 (m, 5H, CH_{Ar}), 4.51 (s, 2H, PhCH_2), 3.47 (t, *J* = 6.6 Hz, 2H, BnOCH_2), 2.31 (dd, *J* = 15.5, 7.0 Hz, 1H, C*H*HCO$_2$H), 2.27 (dd, *J* = 15.5, 6.7 Hz, 1H, CH*H*CO$_2$H), 1.84 (m, *J* = 6.5 Hz, 1H, C*H*), 1.69-1.58 (m, 2H, BnOCH$_2$CH_2), 1.50-1.30 (m, 4H, BnOCH$_2$CH$_2$CH_2, CH$_3$CH_2), 0.89 (t, *J* = 7.4 Hz, 3H, CH_3).

^{13}C NMR ($CDCl_3$, 100 MHz): δ 179.8, 138.6, 128.5, 127.8, 127.6, 73.0, 70.6, 38.6, 36.2, 29.9, 26.9, 26.3, 10.9.

HRMS (ESI, m/Z): calcd. for $C_{15}H_{21}O_3$ $[M-H]^-$: 249.14962, found: 249.14996.

The *ee* of this compound was determined from the corresponding *N,N*-dimethyl amide derivative. HPLC: Chiracel-ASH, *n*heptane/*i*PrOH 98:2, 0.5 mL/min., 40 °C, detection at 207 nm. Retention time (min): 21.9 (minor) and 23.9 (major).

(*R*)-3-Methylhexanoic acid (3a)[8]

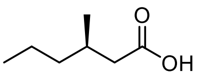

3a

The reaction was performed with **1a** (22.8 mg, 0.2 mmol, 1.0 equiv.), $CuBr{\cdot}SMe_2$ (4.12 mg, 0.02 mmol, 10 mol%), ligand (*R*)-**L4** (16.30 mg, 0.024 mmol, 12 mol%), Me_3SiOTf (109 µL, 0.6 mmol, 3.0 equiv), MeMgBr (0.6 mmol, 3.0 M in Et_2O, 3.0 equiv.), *t*BuOMe (2.0 mL) at −20 °C, and following

General Work-up **B**. Product **3a** was obtained as a colorless oil without further purification [97% conversion, 93% yield, 96% *ee*].

^{1}H NMR ($CDCl_3$, 400 MHz): δ 2.35 (dd, *J* = 14.9, 5.9 Hz, 1H, C*H*HCO_2H), 2.14 (dd, *J* = 14.9, 8.2 Hz, 1H, CH*H*CO_2H), 2.04-1.91 (m, 1H, C*H*), 1.42-1.14 (m, 4H, $CH_3CH_2CH_2$, $CH_3CH_2CH_2$), 0.96 (d, *J* = 6.6 Hz, 3H, CH_3CH), 0.90 (t, *J* = 7.1 Hz, 3H, CH_3CH_2).

^{13}C NMR ($CDCl_3$, 100 MHz): δ 180.0, 41.8, 39.1, 30.0, 20.1, 19.8, 14.3.

The *ee* of this compound was determined from the corresponding *N,N*-dimethyl amide derivative. HPLC: Chiracel-OBH, *n*heptane/*i*PrOH 90:10, 0.5 mL/min., 40 °C, detection at 209 nm. Retention time (min): 9.0 (minor) and 10.1 (major).

(*R*)-3-Phenylbutanoic acid (3b)[9]

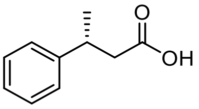

3b

The reaction was performed with **1e** (148.2 mg, 1.0 mmol, 1.0 equiv.), CuBr·SMe_2 (20.56 mg, 0.1 mmol, 10 mol%), ligand (*R*,*R*)-**L5** (60.79 mg, 0.12 mmol, 12 mol%), Me_3SiOTf (544 μL, 0.6 mmol, 3.0 equiv), *t*BuOMe (5.0 mL), Toluene (5.0 mL) at −20 °C, and MeMgBr (3.0 mmol, 3.0 M in Et_2O, 3.0 equiv.) was added with syringe pump in 10 min. The reaction was quenched with saturated $NaHCO_3$ aqueous solution (10.0 mL), warmed to room temperature and the organic phase was extracted. The organic phase was further extracted with saturated Na_2CO_3 aqueous solution (10.0 mL) for another three times. The combined aqueous phase was acidified with HCl aqueous solution (15.0 mL, 12.0 M), and extracted with CH_2Cl_2 (30.0 mL × 3). The combined organic phase was dried over $MgSO_4$, filtered and evaporated on rotary evaporator. Product **3b** was obtained as a colorless oil without further purification [97% conversion, 90% yield, 99% *ee*].

^{1}H NMR ($CDCl_3$, 400 MHz): δ 7.35-7.29 (m, 2H, CH_{Ar}), 7.26-7.18 (m, 3H, CH_{Ar}), 3.29 (m, *J* = 7.1 Hz, 1H, C*H*), 2.69 (dd, *J* = 15.5, 6.8 Hz, 1H, C*H*HCO_2H), 2.59 (dd, *J* = 15.5, 8.3 Hz, 1H, CH*H*CO_2H), 1.33 (d, *J* = 6.9 Hz, 3H, CH_3).

^{13}C NMR ($CDCl_3$, 100 MHz): δ 179.2, 145.6, 128.7, 126.8, 126.6, 42.7, 36.2, 22.0.

The *ee* of this compound was determined from the corresponding *N,N*-dimethyl amide derivative. HPLC: Chiracel-ODH, *n*heptane/*i*PrOH 90:10, 0.5 mL/min., 40 °C, detection at 208 nm. Retention time (min): 19.7 (minor) and 22.1 (major).

(*R*)-3-(4-Methylphenyl)butanoic acid (3c)[10]

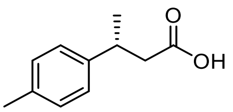

3c

The reaction was performed with **1n** (32.4 mg, 0.2 mmol, 1.0 equiv.), $CuBr\cdot SMe_2$ (4.12 mg, 0.02 mmol, 10 mol%), ligand (*R,R*)-**L5** (12.16 mg, 0.024 mmol, 12 mol%), Me_3SiOTf (109 µL, 0.6 mmol, 3.0 equiv), MeMgBr (0.6 mmol, 3.0 M in Et_2O, 3.0 equiv.), *t*BuOMe (1.0 mL), Toluene (1.0 mL) at −20 °C, and following General Work-up **C**. Product **3c** was obtained as a colorless oil after column chromatography (SiO_2, pentane:Et_2O = 5:1) [91% conversion, 74% yield, 99% *ee*].

^{1}H NMR ($CDCl_3$, 400 MHz): δ 7.13 (s, 4H, CH_{Ar}), 3.26 (m, *J* = 7.1 Hz, 1H, C*H*), 2.67 (dd, *J* = 15.5, 6.8 Hz, 1H, C*H*HCO_2H), 2.57 (dd, *J* = 15.5, 8.2 Hz, 1H, CH*H*CO_2H), 2.34 (s, 3H, ArCH_3), 1.32 (d, *J* = 7.0 Hz, 3H, CHCH_3).

^{13}C NMR ($CDCl_3$, 100 MHz): δ 178.9, 142.6, 163.1, 129.4, 126.7, 42.8, 35.9, 22.1, 21.1.

The *ee* of this compound was determined from the corresponding *N,N*-dimethyl amide derivative. HPLC: Chiracel-ODH, *n*heptane/*i*PrOH 95:5, 0.5 mL/min., 40 °C, detection at 207 nm. Retention time (min): 15.3 (minor) and 17.7 (major).

(*R*)-3-Cyclopentylhexanoic acid (3d)

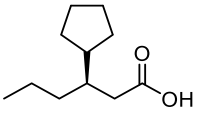

3d

The reaction was performed with **1a** (22.8 mg, 0.2 mmol, 1.0 equiv.), $CuBr\cdot SMe_2$ (4.12 mg, 0.02 mmol, 10 mol%), ligand (*R*)-**L4** (16.30 mg, 0.024 mmol, 12 mol%), Me_3SiOTf (80 µL, 0.44 mmol, 2.2 equiv), cyclopentylMgBr (0.5 mmol, 2.0 M in Et_2O, 2.5 equiv.), *t*BuOMe (2.0 mL) at −20 °C, and following General Work-up **A**. Product **3d** was obtained as a colorless oil after column chromatography (SiO_2, pentane:Et_2O = 15:1) [91% conversion, 79% yield, 96% *ee*].

^{1}H NMR ($CDCl_3$, 400 MHz): δ 2.37 (dd, *J* = 15.2, 4.6 Hz, 1H, C*H*HCO_2H), 2.28 (dd, *J* = 15.3, 6.5 Hz, 1H, CH*H*CO_2H), 1.86-1.68 (m, 4H, C*H*, CH_2), 1.67-1.45 (m, 4H, CH_2), 1.45-1.22 (m, 4H, CH_2), 1.21-1.06 (m, 2H, CH_2), 0.89 (t, *J* = 6.4 Hz, 3H, CH_3).

^{13}C NMR ($CDCl_3$, 100 MHz): δ 180.7, 44.1, 39.8, 37.6, 35.1, 30.5, 30.3, 25.5, 25.4, 19.7, 14.6.

HRMS (ESI, m/Z): calcd. for $C_{11}H_{19}O_2$ $[M-H]^-$: 183.13905, found: 183.13919.

The *ee* of this compound was determined from the corresponding *N*,*N*-dimethyl amide derivative. HPLC: Chiracel-OZH, *n*heptane/*i*PrOH 98:2, 0.5 mL/min., 40 °C, detection at 206 nm. Retention time (min): 30.1 (minor) and 32.3 (major).

(*S*)-5-Methyl-3-propylhexanoic acid (3e)

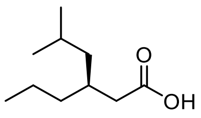

3e

The reaction was performed with **1a** (22.8 mg, 0.2 mmol, 1.0 equiv.), $CuBr·SMe_2$ (2.06 mg, 0.01 mmol, 5 mol%), ligand (*R*)-**L4** (8.15 mg, 0.012 mmol, 6 mol%), Me_3SiOTf (80 μL, 0.44 mmol, 2.2 equiv), *i*BuMgBr (0.5 mmol, 2.0 M in Et_2O, 2.5 equiv.), *t*BuOMe (2.0 mL) at −20 °C, and following General Work-up **A**. Product **3e** was obtained as a colorless oil after column chromatography (SiO_2, pentane:Et_2O = 15:1) [95% conversion, 83% yield, 95% *ee*].

^1H NMR ($CDCl_3$, 400 MHz): δ 2.26 (d, *J* = 6.7 Hz, 2H, CH_2CO_2H), 2.00-1.88 (m, 1H, $CH_3CH_2CH_2CH$), 1.71-1.56 (m, 1H, CH_3CH), 1.37-1.23 (m, 4H, CH_3CH_2, $CH_3CH_2CH_2$), 1.24-1.07 (m, 2H, CH_3CHCH_2), 0.93-0.83 (m, 9H, CH_3).

^{13}C NMR ($CDCl_3$, 100 MHz): δ 180.4, 43.8, 39.4, 36.6, 32.6, 25.4, 22.9, 22.8, 19.6, 14.4.

HRMS (ESI, m/Z): calcd. for $C_{10}H_{19}O_2$ $[M-H]^-$: 171.13905, found: 171.13925.

The *ee* of this compound was determined from the corresponding *N*,*N*-dimethyl amide derivative. HPLC: Chiracel-ADH, *n*heptane/*i*PrOH 95.5:0.5, 0.5 mL/min., 40 °C, detection at 215 nm. Retention time (min): 44.2 (major) and 47.9 (minor).

(*S*)-6-Methyl-3-propylheptanoic acid (3f)

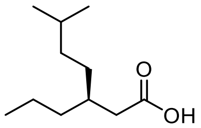

3f

The reaction was performed with **1a** (22.8 mg, 0.2 mmol, 1.0 equiv.), $CuBr·SMe_2$ (2.06 mg, 0.01 mmol, 5 mol%), ligand (*R*)-**L4** (8.15 mg, 0.012 mmol, 6 mol%), Me_3SiOTf (80 μL, 0.44 mmol, 2.2 equiv), *i*PentMgBr (0.5 mmol, 2.0 M in Et_2O, 2.5 equiv.), *t*BuOMe (2.0 mL) at −20 °C, and following

General Work-up **A**. Product **3f** was obtained as a colorless oil after column chromatography (SiO_2, pentane:Et_2O = 15:1) [98% conversion, 84% yield, 98% *ee*].

^{1}H NMR ($CDCl_3$, 400 MHz): δ 2.28 (d, *J* = 6.8 Hz, 2H, C$H_2$$CO_2$H), 1.86 (m, *J* = 6.3 Hz, 1H, $CH_3CH_2CH_2C$*H*), 1.56-1.44 (m, 1H, CH_3C*H*), 1.40-1.21 (m, 6H, CH_2), 1.21-1.12 (m, 2H, $CH_3CHC$$H_2$), 0.94-0.82 (m, 9H, C$H_3$).

^{13}C NMR ($CDCl_3$, 100 MHz): δ 180.4, 39.2, 36.3, 35.8, 35.0, 31.6, 28.4, 22.8, 22.7, 19.8, 14.4.

HRMS (ESI, m/Z): calcd. for $C_{11}H_{21}O_2$ $[M-H]^-$: 185.15470, found: 185.15487.

The *ee* of this compound was determined from the corresponding *N,N*-dimethyl amide derivative. HPLC: Chiracel-ODH, *n*heptane/*i*PrOH 98:2, 0.5 mL/min., 40 °C, detection at 208 nm. Retention time (min): 12.9 (major) and 13.7 (minor).

(*S*)-3-Propyl-oct-7-enoic acid (3g)

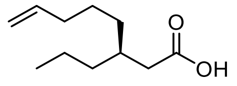

3g

The reaction was performed with **1a** (22.8 mg, 0.2 mmol, 1.0 equiv.), CuBr·SMe_2 (2.06 mg, 0.01 mmol, 5 mol%), ligand (*R*)-**L4** (8.15 mg, 0.012 mmol, 6 mol%), Me_3SiOTf (80 μL, 0.44 mmol, 2.2 equiv), pent-4-en-1-ylMgBr (0.5 mmol, 2.0 M in *t*BuOMe, 2.5 equiv.), *t*BuOMe (2.0 mL) at −20 °C, and following General Work-up **A**. Product **3g** was obtained as a colorless oil after column chromatography (SiO_2, pentane:Et_2O = 15:1) [94% conversion, 85% yield, 98% *ee*].

^{1}H NMR ($CDCl_3$, 400 MHz): δ 5.86-5.74 (m, 1H, CH_2=C*H*), 5.00 (m, *J* = 17.1, 1.7 Hz, 1H, C*H*H=CH), 4.96-4.92 (m, 1H, CH*H*=CH), 2.28 (d, *J* = 6.8 Hz, 2H, C$H_2$$CO_2$H), 2.08-2.00 (m, 2H, CH_2=CHCH_2), 1.94-1.83 (m, 1H, $CH_3CH_2CH_2C$*H*), 1.45-1.20 (m, 8H, CH_2), 0.95-0.82 (m, 3H, CH_3).

^{13}C NMR ($CDCl_3$, 100 MHz): δ 180.3, 138.9, 114.6, 39.1, 36.2, 34.7, 34.1, 33.4, 26.0, 19.8, 14.4.

HRMS (ESI, m/Z): calcd. for $C_{11}H_{19}O_2$ $[M-H]^-$: 183.13905, found: 183.13927.

The *ee* of this compound was determined from the corresponding *N,N*-dimethyl amide derivative. HPLC: Chiracel-OBH, *n*heptane/*i*PrOH 99.7:0.3, 0.5 mL/min., 40 °C, detection at 215 nm. Retention time (min): 39.5 (major) and 44.1 (minor).

(*S*)-3-Propylnonanoic acid (3h)

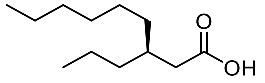

3h

The reaction was performed with **1a** (144.1 mg, 1.0 mmol, 1.0 equiv.), CuBr·SMe_2 (10.3 mg, 0.05 mmol, 5 mol%), ligand (*R*)-**L4** (40.7 mg, 0.06 mmol, 6 mol%), Me_3SiOTf (398 μL, 2.2 mmol, 2.2 equiv), *t*BuOMe (10.0 mL) at −20 °C, and *n*HexMgBr (2.5 mmol, 2.0 M in Et_2O, 2.5 equiv.) was added with syringe pump in 10 min. The reaction was quenched with HCl aqueous solution (5.0 mL, 1.0 M) and warmed to RT. The mixture was extracted with CH_2Cl_2 (30.0 mL × 3). The combined organic phase was dried over $MgSO_4$, filtered and evaporated on rotary evaporator. Pentane (3.0 mL × 3) was added to the residue and the mixture was filtered with a small piece of cotton in glass pipette to remove most of the catalyst. product **3h** was obtained as a colorless oil after column chromatography (SiO_2, pentane:Et_2O = 10:1) [99% conversion, 79% yield, 98% *ee*].

^{1}H NMR ($CDCl_3$, 400 MHz): δ 2.27 (d, *J* = 6.8 Hz, 2H, CH_2CO_2H), 1.93-1.80 (m, 1H, C*H*), 1.39-1.19 (m, 14H, CH_2), 0.94-0.83 (m, 6H, CH_3).

^{13}C NMR ($CDCl_3$, 100 MHz): δ 180.4, 39.2, 36.3, 34.8, 34.0, 32.0, 29.7, 26.6, 22.8, 19.8, 14.4, 14.2.

HRMS (ESI, m/Z): calcd. for $C_{12}H_{23}O_2$ $[M-H]^-$: 199.17035, found: 199.17054.

The *ee* of this compound was determined from the corresponding *N,N*-dimethyl amide derivative. HPLC: Chiracel-ODH, *n*heptane/*i*PrOH 98:2, 0.5 mL/min., 40 °C, detection at 207 nm. Retention time (min): 12.8 (major) and 13.6 (minor).

(*S*)-3-Methylnonanoic acid (3i)

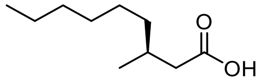

3i

The reaction was performed with **1b** (17.2 mg, 0.2 mmol, 1.0 equiv.), CuBr·SMe_2 (2.06 mg, 0.01 mmol, 5 mol%), ligand (*R*)-**L4** (8.15 mg, 0.012 mmol, 6 mol%), Me_3SiOTf (80 μL, 0.44 mmol, 2.2 equiv), *n*HexMgBr (0.5 mmol, 2.0 M in Et_2O, 2.5 equiv.), *t*BuOMe (2.0 mL) at −78 °C for 16 h, and following General Work-up **A**. Product **3i** was obtained as a colorless oil after column chromatography (SiO_2, pentane:Et_2O = 15:1) [97% conversion, 84% yield, 98% *ee*].

^{1}H NMR ($CDCl_3$, 400 MHz): δ 2.35 (dd, *J* = 14.9, 5.9 Hz, 1H, C*H*HCO$_2$H), 2.14 (dd, *J* = 14.9, 8.1 Hz, 1H, CH*H*CO$_2$H), 2.03-1.88 (m, 1H, C*H*), 1.39-1.14 (m, 10H, CH_2), 0.96 (d, *J* = 6.6 Hz, 1H, CH_3CH), 0.87 (t, *J* = 6.6 Hz, 1H, CH_3CH_2).

^{13}C NMR ($CDCl_3$, 100 MHz): δ 180.2, 41.8, 36.8, 32.0, 30.3, 29.5, 27.0, 22.8, 19.8, 14.2.

HRMS (ESI, m/Z): calcd. for $C_{10}H_{19}O_2$ $[M-H]^-$: 171.13905, found: 171.13929.

The *ee* of this compound was determined from the corresponding *N,N*-dimethyl amide derivative. HPLC: Chiracel-ASH, *n*heptane/*i*PrOH 99:1, 0.5 mL/min., 40 °C, detection at 206 nm. Retention time (min): 14.4 (major) and 16.0 (minor).

Decarboxylative cross-coupling reactions

(*S*)-1-(2-Ethylpentyl)-4-methoxy-benzene (4a)

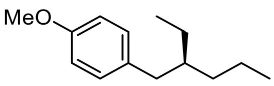

4a

Procedure for *in situ* Ni-catalyzed decarboxylative alkylation of 2k. The reaction was performed according to the literatre.[11] In a flame-dried Schlenk tube equipped with septum and magnetic stirring bar, **2k** (44.5 mg, 0.2 mmol, 1.0 equiv.) and HATU (76.05 mg, 0.2 mmol, 1.0 equiv.) were dissolved in DMF (1.0 mL). Et_3N (28 μL, 0.2 mmol, 1.0 equiv) was added and the mixture was stirred under nitrogen atmosphere for 30 min. at RT. A solution of $NiCl_2$·glyme (8.78 mg, 0.04 mmol, 20 mol%), and 4,4'-di-*t*-butyl-2,2'-dipyridyl (21.5 mg, 0.08 mmol, 40 mol%) in DMF (1.0 mL) was added, and the mixture was stirred for 5 min. Diethylzinc (0.4 mmol, 1.0 M in hexane, 2.0 equiv) was then added with syringe pump in 30 min. The resulting mixture was allowed to stir for 16 h at RT. The reaction mixture was quenched with HCl aqueous solution (3.0 mL, 1.0 M) and extracted with Et_2O (10.0 mL × 3). The organic layer was washed with water and brine and dried over $MgSO_4$. The organic layer was concentrated under vacuum by rotary evaporator in a water bath at 40 °C. The crude product was purified by silica gel flash column chromatography (SiO_2, pentane:Et_2O = 400:1) to yield pure product **4a** as a colorless oil. [41% yield, 96% *ee*].

^{1}H NMR ($CDCl_3$, 400 MHz): δ 7.10-7.04 (m, 2H, H_{Ar}), 6.86-6.79 (m, 2H, H_{Ar}), 3.79 (s, 3H, CH_3O), 2.49 (dd, J = 14.0, 7.0 Hz, 1H, PhC*H*H), 2.45 (dd, J = 14.0, 7.0 Hz, 1H, PhCH*H*), 1.58-1.48 (m, 1H, C*H*), 1.39-1.17 (m, 6H, CH_2), 0.94-0.82 (m, 6H, CH_3CH_2, $CH_2CH_2CH_3$).

^{13}C NMR ($CDCl_3$, 100 MHz): δ 157.7, 134.0, 130.2, 113.6, 55.4, 41.2, 39.3, 35.1, 25.5, 19.9, 14.6, 10.9.

HRMS was measured after transforming to **4f**, see compound **4f**.

The *ee* of this compound was determined after transforming it to **4f**, see compound **4f**.

(*R*)-4,4,5,5-Tetramethyl-2-(2-(4-methoxybenzyl)butyl)-1,3,2-dioxaborolane (4b)

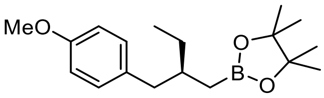

4b

Preparation of $NiCl_2\cdot 6H_2O$/4,4′-dimethoxy-2,2′-bipyridyl suspension (0.05 M in DMF). In a flame-dried Schlenk tube equipped with septum and magnetic stirring bar, $NiCl_2\cdot 6H_2O$ (9.5 mg, 0.04 mmol) and 4,4′-dimethoxy-2,2′-bipyridyl (11.2 mg, 0.052 mmol) were dissolved in DMF (0.8 mL), and the mixture was stirred under nitrogen atmosphere for 16 h to afford a pale green suspension.

Preparation of [B_2pin_2Me]Li complex (0.6 M in THF and Et_2O). In a flame-dried Schlenk tube equipped with septum and magnetic stirring bar, MeLi (0.75 mL, 1.6 M in Et_2O, 1.2 mmol) was added to a solution of B_2pin_2 (335.2 mg, 1.32 mmol) in THF (1.25 mL) at 0 °C under nitrogen atmosphere. The reaction mixture was warmed to RT and stirred for 1 h to afford a suspension (sometimes a clear solution was observed).

Procedure for *in situ* Ni-catalyzed decarboxylative borylation of 2k. The reaction was performed according to the literatre.[12] In a flame-dried Schlenk tube equipped with septum and magnetic stirring bar, **2k** (44.5 mg, 0.2 mmol, 1.0 equiv.), N-hydroxyphthalimide (32.6 mg, 0.2 mmol, 1.0 equiv.) and *N,N′*-dicyclohexylcarbodiimide (31 μL, 0.2 mmol, 1.0 equiv.) were dissolved in CH_2Cl_2 (2.0 mL). The resulting mixture was stirred under nitrogen atmosphere for 2 h at RT before the volatiles were removed *in vacuo*. $MgBr_2\cdot OEt_2$ (77 mg, 0.3 mmol, 1.5 equiv.) was added, and the tube was evacuated and backfilled with nitrogen for three times. $NiCl_2\cdot 6H_2O$/4,4'-dimethoxy-2,2'-bipyridyl suspension (0.4 mL, 0.02 mmol, 10 mol%) was added, and the mixture was stirred vigorously for 10 min. at RT. The mixture was subsequently cooled to 0 °C and [B_2pin_2Me]Li complex (1.0 mL, 0.6 mmol, 3.0 equiv.) was added in one portion (note: do not add it dropwise!). After stirring at 0 °C for 1 h, the reaction was warmed to RT and stirred for another 1 h. The reaction mixture was then quenched with HCl aqueous solution (5.0 mL, 0.1 M) and extracted with Et_2O (10.0 mL × 3). The combined organic layers were dried over $MgSO_4$, concentrated *in vacuo* and purified by column chromatography (SiO_2, pentane:Et_2O = 40:1) to afford pure product **4b** as a colorless oil. [32% yield, 96% *ee*].

^1H NMR ($CDCl_3$, 400 MHz): δ 7.11-7.06 (m, 2H, H_{Ar}), 6.84-6.78 (m, 2H, H_{Ar}), 3.78 (s, 3H, CH_3O), 2.52 (dd, J = 13.5, 7.0 Hz, 1H, PhC*H*H), 2.47 (dd, J = 13.5, 6.9 Hz, 1H, PhCH*H*), 1.86-1.74 (m, 1H, C*H*), 1.42-1.29 (m, 1H, CH_3C*H*H), 1.29-1.15 (m, 1H, CH_3CH*H*), 1.24 (s, 12H, CCH_3), 0.88 (t, J = 7.4 Hz, 3H, CH_3CH_2), 0.78 (dd, J = 15.6, 6.9 Hz, 1H, C*H*HB), 0.73 (dd, J = 15.6, 7.1 Hz, 1H, CH*H*B).

^{13}C NMR ($CDCl_3$, 100 MHz): δ 157.7, 133.9, 130.4, 113.6, 83.0, 55.4, 41.9, 38.2, 28.4, 25.0, 25.0, 11.3.

HRMS was measured after transforming to **4g**, see compound **4g**.

The *ee* of this compound was determined after transforming to **4g**, see compound **4g**.

(*R*)-(2-Bromo-1-methylethyl)benzene (4c)[13]

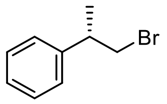

4c

Preparation of Ag(Phen)$_2$OTf.[14] The reaction was performed according to the literatre.[14] To a stirred mixture of AgOTf (256.9 mg, 1.0 mmol, 1.0 equiv.) in MeOH (5.0 mL) was added 1,10-phenanthroline anhydrate (360.4 mg, 2.0 mmol, 2.0 equiv.) in MeOH (10.0 mL). The colorless solution turned into yellow suspension on adding a 1,10-phenanthroline solution. After stirring for three hours the solid was collected on a Kiriyama filter and washed with MeOH, then dried under vacuum for overnight to give Ag(phen)$_2$OTf as yellow solid [71% yield].

^{1}H NMR (DMSO-d_6, 400 MHz): 9.16 (dd, J = 4.5, 1.6 Hz, 4H, H_{Ar}), 8.79 (dd, J = 8.1, 1.6 Hz, 4H, H_{Ar}), 8.23 (s, 4H, H_{Ar}), 8.01 (dd, J = 8.1, 4.5 Hz, 4H, H_{Ar}).

Procedure for Ag-catalyzed decarboxylative bromonation of 3b. The reaction was performed according to the literatre.[15] In a Schlenk tube equipped with septum and magnetic stirring bar, **3b** (32.8 mg, 0.2 mmol, 1.0 equiv.), Ag(Phen)$_2$OTf (12.0 mg, 0.0.2 mmol, 10 mol%), dibromoisocyanuric acid (172.1 mg, 0.6 mmol, 3.0 equiv.) and 1,2-dichloroethane (8 mL) were added. The mixture was heated at 60 °C under nitrogen atmosphere for 16 h. The reaction was cooled to RT and pentane (10 mL) was added. The reaction mixture was filtered and washed with pentane. The combined organic phase was concentrated under reduced pressure. Product **4c** was obtained as a colorless oil after column chromatography (SiO$_2$, pentane), and the components were visualized by PMA [46% yield, 99% *ee*].

^{1}H NMR (CDCl$_3$, 400 MHz): δ 7.37-7.29 (m, 2H, H_{Ar}), 7.28-7.19 (m, 2H, H_{Ar}), 3.58 (dd, J = 9.9, 6.0 Hz, 1H, C*H*HBr), 3.48 (dd, J = 9.9, 8.0 Hz, 1H, CH*H*Br), 3.19-3.08 (m, 1H, C*H*), 1.42 (d, J = 6.9, 3H, CH_3).

^{13}C NMR (CDCl$_3$, 100 MHz): δ 143.9, 128.7, 127.2, 127.1, 42.4, 40.1, 20.1.

HPLC: Chiracel-OJH, *n*heptane/*i*PrOH 98:2, 0.5 mL/min., 40 °C, detection at 208 nm. Retention time (min): 9.1 (major) and 10.0 (minor).

(*S*)-1-Azido-2-propyloctane (4d)

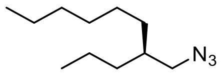

4d

Preparation of $MesSO_2N_3$.[16] The reaction was performed according to the literatre.[17] A warmed (45 °C) solution of $MesSO_2Cl$ (1093.5 mg, 5.0 mmol, 1.0 equiv.) in ethanol (5.0 mL) was added to sodium azide (487.6 mg, 7.5 mmol, 1.5 equiv.) in water (1.0 mL) and ethanol (2.0 mL). The mixture was stirred for 3 h at room temperature and then concentrated by rotary evaporation, gradually warming the bath from 10 °C to 35 °C to prevent foaming. Water was added to the residue (5.0 mL) and the aqueous phase was extracted with Et_2O (10.0 mL × 3). The combined organic layer was dried over $MgSO_4$, filtered and evaporated on rotary evaporator. $MesSO_2N_3$ was obtained as a colorless oil after column chromatography (SiO_2, pentane:Et_2O = 100:1), and the components were visualized by PMA staining [81% yield].

^{1}H NMR ($CDCl_3$, 400 MHz): δ 7.02 (s, 2H, H_{Ar}), 2.66 (s, 6H, CH_3), 2.34 (s, 3H, CH_3).

^{13}C NMR ($CDCl_3$, 100 MHz): δ 144.7, 140.1, 133.4, 132.3, 22.9, 21.3.

Procedure for Ag-catalyzed decarboxylative azidation of 3h. The reaction was performed according to the literatre.[18] In a Schlenk tube equipped with septum and magnetic stirring bar, **3h** (120.2 mg, 0.6 mmol, 1.0 equiv.), AgF (22.8 mg, 0.18 mmol, 30 mol%) and $K_2S_2O_8$ (162.2 mg, 0.6 mmol, 1.0 equiv.) were added, followed by addition of CH_3CN (6.0 mL), H_2O (6.0 mL) and $MesSO_2N_3$ (270.3 mg, 1.2 mmol, 2.0 equiv.). The mixture was heated at 55 °C under nitrogen atmosphere for 48 h. The reaction was cooled to RT and the mixture was extracted with Et_2O (10.0 mL × 3). The combined organic layer was dried over $MgSO_4$, filtered and evaporated on rotary evaporator. Product **4d** was obtained as a colorless oil after column chromatography (SiO_2, pentane), and the components were visualized by bromocresol green staining. [53% yield, 98% *ee*].

^{1}H NMR ($CDCl_3$, 400 MHz): δ 3.23 (d, J = 5.9 Hz, 2H, CH_2N_3), 1.61-1.45 (m, 1H, C*H*), 1.37-1.22 (m, 14H, CH_2), 0.97-0.84 (m, 6H, CH_3).

^{13}C NMR ($CDCl_3$, 100 MHz): δ 55.4, 38.1, 34.2, 32.0, 31.9, 29.7, 26.7, 22.8, 19.9, 14.5, 14.2.

HRMS was measured after transforming to **4e**, see compound **4e**.

The *ee* of this compound was determined after transforming to **4e**, see compound **4e**.

(*S*)-1-(2-Propyl)octyl-4-phenyl-1*H*-1,2,3-triazole (4e)

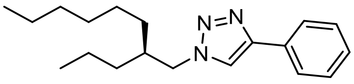

4e

Procedure for Cu-catalyzed click reaction of 4d. The reaction was performed according to the literatre.[19] In a flame-dried Schlenk tube equipped with septum and magnetic stirring bar, CuTc (7.6 mg, 0.04 mmol, 20 mol%) was added to a solution of **4d** (39.5 mg, 0.2 mmol, 1.0 equiv.) and

phenylacetylene (24.5 mg, 0.24 mmol, 1.2 equiv.) in Toluene (2.0 mL). The reaction mixture was stired under nitrogen atmosphere for 2 h at RT before the volatiles were removed *in vacuo*. Product **4e** was obtained as a white solid after column chromatography (SiO_2, pentane:Et_2O = 10:1) [78% yield, 98% *ee*].

^1H NMR ($CDCl_3$, 400 MHz): δ 7.88-7.82 (m, 2H, H_{Ar}), 7.71 (s, 1H, H_{Ar}), 7.46-7.39 (m, 2H, H_{Ar}), 7.36-7.30 (m, 1H, H_{Ar}), 4.29 (d, *J* = 6.8 Hz, 2H, CH_2N), 1.99 (m, *J* = 6.1 Hz, 1H, C*H*), 1.44-1.17 (m, 14H, CH_2), 0.94-0.82 (m, 6H, CH_3).

^{13}C NMR ($CDCl_3$, 100 MHz): δ 147.7, 130.9, 128.9, 128.1, 125.8, 120.0, 54.1, 39.0, 33.7, 31.9, 31.4, 29.6, 26.4, 22.7, 19.6, 14.4, 14.2.

HRMS (ESI, m/Z): calcd. for $C_{19}H_{30}N_3$ $[M+H]^+$: 300.24342, found: 300.24378.

HPLC: Chiracel-OZH, *n*heptane/*i*PrOH 90:10, 0.5 mL/min., 40 °C, detection at 245 nm. Retention time (min): 32.3 (minor) and 34.7 (major).

(*S*)-4-(2-Ethylpentyl)phenol (4f)

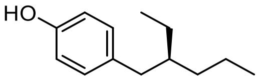

4f

The reaction was performed according to the literatre.[20] **4a** (16.5 mg, 0.08 mmol), Aliquat-336 (50 mg) and HBr aqueous solution (47%, 1 mL) was added to a Schlenk tube equipped with septum and magnetic stirring bar. The resulting reaction mixture was heated at 105 °C under nitrogen atmosphere for 16 h. The reaction was cooled to RT and water was added (2.0 mL). The mixture was extracted with Et_2O (10.0 mL × 3), and the combined organic layer was dried over $MgSO_4$, filtered and evaporated on rotary evaporator. The crude product was purified by silica gel flash column chromatography (SiO_2, pentane:Et_2O = 10:1) to yield pure product **4f** as a colorless oil. [96% *ee*].

^1H NMR ($CDCl_3$, 400 MHz): δ 7.04-6.99 (m, 2H, H_{Ar}), 6.77-6.72 (m, 2H, H_{Ar}), 4.62 (s, 1H, O*H*), 2.47 (dd, *J* = 14.0, 7.0 Hz, 1H, PhC*H*H), 2.45 (dd, *J* = 14.1, 7.1 Hz, 1H, PhCH*H*), 1.51 (m, *J* = 6.4 Hz, 1H, C*H*), 1.39-1.16 (m, 6H, CH_2), 0.92-0.81 (m, 6H, CH_3).

^{13}C NMR ($CDCl_3$, 100 MHz): δ 153.5, 134.2, 130.4, 115.0, 41.2, 39.3, 35.1, 25.5, 19.9, 14.6, 10.9.

HRMS (ESI, m/Z): calcd. for $C_{13}H_{19}O$ $[M-H]^-$: 191.14414, found: 191.14423.

HPLC: Chiracel-OZH, *n*heptane/*i*PrOH 99:1, 0.5 mL/min., 40 °C, detection at 224 nm. Retention time (min): 63.3 (major) and 69.8 (minor).

(*R*)-2-(4-Methoxybenzyl)butan-1-ol (4g)[21]

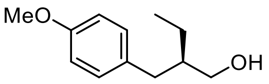

4g

The reaction was performed according to the literatre.[22] In a Schlenk tube equipped with septum and magnetic stirring bar, **4b** (18.3 mg, 0.06 mmol, 1.0 equiv.) was dissolved in 1 mL THF and 1 mL H_2O. $NaBO_3·4H_2O$ (27.6 mg, 0.18 mmol, 3.0 equiv.) was added and the reaction mixture was stirred under nitrogen atmosphere for 16 h at RT. Water (2.0 mL) was added and the mixture was extracted with Et_2O (10.0 mL × 3). The combined organic phase was dried over $MgSO_4$, filtered and evaporated on rotary evaporator. Product **4g** was obtained as a colorless oil after column chromatography (SiO_2, pentane:Et_2O = 4:1). [96% *ee*].

^{1}H NMR ($CDCl_3$, 400 MHz): δ 7.13-7.08 (m, 2H, H_{Ar}), 6.86-6.81 (m, 2H, H_{Ar}), 3.79 (s, 3H, CH_3O), 3.54 (d, *J* = 5.3 Hz, 2H, CH_2OH), 2.58 (d, *J* = 7.1 Hz, 2H, PhCH_2), 1.75-1.63 (m, 1H, C*H*), 1.45-1.31 (m, 2H, CH_3CH_2), 0.94 (t, *J* = 7.5 Hz, 3H, C$H_3$$CH_2$).

^{13}C NMR ($CDCl_3$, 100 MHz): δ 158.0, 132.9, 130.2, 113.9, 64.7, 55.4, 44.4, 36.5, 23.4, 11.5.

HRMS (ESI, m/Z): calcd. for $C_{12}H_{17}O_2$ $[M-H]^-$: 193.12340, found: 193.12357.

HPLC: Chiracel-ODH, *n*heptane/*i*PrOH 95:5, 0.5 mL/min., 40 °C, detection at 224 nm. Retention time (min): 20.6 (major) and 22.7 (minor).

Functionalization of 5a

(*R*)-3-(4-hydroxy-3-(5,6,7,8-tetrahydro-5,5,8,8-tetramethyl-3-pentyloxy-2-naphthalenyl)phenyl) butanoic acid (5b)

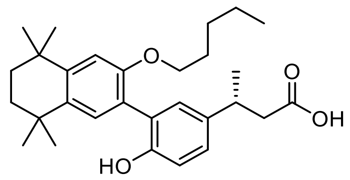

5b

The reaction was performed with **5a** (21.8 mg, 0.05 mmol, 1.0 equiv.), CuBr·SMe_2 (1.03 mg, 0.005 mmol, 10 mol%), ligand (*R*,*R*)-**L5** (3.04 mg, 0.006 mmol, 12 mol%), Me_3SiOTf (36 µL, 0.2 mmol, 4.0 equiv), MeMgBr (0.2 mmol, 3.0 M in Et_2O, 4.0 equiv.), *t*BuOMe (0.5 mL), Toluene (0.5 mL) at −20 °C. The reaction was quenched with HCl aqueous solution (2.0 mL, 1.0 M) and warmed to RT. The mixture was extracted with CH_2Cl_2 (10.0 mL × 3). The combined organic phase was dried over

$MgSO_4$, filtered and evaporated on rotary evaporator. Product **5b** was obtained as a colorless oil after column chromatography (SiO_2, pentane:Et_2O = 5:1) [76% yield, 99% *ee*].

^1H NMR ($CDCl_3$, 400 MHz): δ 7.23 (s, 1H, H_{Ar}), 7.16-7.10 (m, 2H, H_{Ar}), 6.95 (d, *J* = 8.1 Hz, 1H, H_{Ar}), 6.93 (s, 1H, H_{Ar}), 4.01 (t, *J* = 6.6 Hz, 2H, CH_2O), 3.35-3.23 (m, 1H, C*H*), 2.70 (dd, *J* = 15.4, 6.4 Hz, 1H, COCH*H*), 2.57 (dd, *J* = 15.5, 8.6 Hz, 1H, COCH*H*), 1.78-1.67 (m, 6H, CH_2), 1.37-1.25 (m, 19H, CH_2, CH_3, CH_3CH), 0.85 (t, *J* = 7.1 Hz, 3H, CH_3CH_2).

^{13}C NMR ($CDCl_3$, 100 MHz): δ 178.1, 152.8, 152.6, 146.3, 139.2, 137.8, 130.7, 129.7, 127.1, 127.0, 125.7, 117.9, 111.4, 70.2, 42.9, 35.6, 35.3, 35.2, 34.7, 34.0, 32.1, 32.0, 29.0, 28.1, 22.4, 22.1, 14.1.

HRMS (ESI, m/Z): calcd. for $C_{29}H_{41}O_4$ $[M+H]^+$: 453.29994, found: 453.29935.

HPLC: Chiracel-ADH, *n*heptane/*i*PrOH 95:5, 0.5 mL/min., 40 °C, detection at 209 nm. Retention time (min): 30.3 (major) and 34.7 (minor).

Catalytic asymmetric conjugate addition of EtMgBr to 1l followed by intramolecular trapping

(1*R*,2*R*)-2-Ethylcyclopentane-1-carboxylic acid (6)

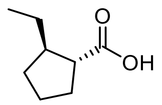

6

In a flame-dried Schlenk tube equipped with septum and magnetic stirring bar, **1l** (38.6 mg, 0.2 mmol, 1.0 equiv.), CuBr·SMe_2 (2.06 mg, 0.01 mmol, 5 mol%) and ligand (*R*)-**L4** (8.15 mg, 0.012 mmol, 6 mol%) were dissolved in *t*BuOMe (2.0 mL) and stirred under nitrogen atmosphere for 20 min. at RT. The mixture was cooled to −78 °C and *n*BuLi (0.2 mmol, 2.5 M in hexane, 1.0 equiv.) was added. After 5 min., Me_3SiOTf (80 μL, 0.44 mmol, 2.2 equiv) was added, and the mixture was allowed to stir for 5 min before EtMgBr (0.3 mmol, 3.0 M in Et_2O, 1.5 equiv.) was added dropwise. The reaction mixture was stirred under nitrogen atmosphere for 2 h, and warmed to RT. After stirring for 16 h, the reaction mixture was quenched HCl aqueous solution (2.0 mL, 1.0 M) and extracted with CH_2Cl_2 (10.0 mL × 3). The combined organic phase was dried over $MgSO_4$, filtered and evaporated on rotary evaporator. Pentane (1.0 mL × 3) was added to the residue and the mixture was filtered with a small piece of cotton in glass pipette to remove most of the catalyst. Product **6** was obtained as a colorless oil after column chromatography (SiO_2, pentane:Et_2O = 10:1) [70% yield, 91% *ee*]. Relative configuration was determined by NOE experiments (see Supplementary Fig. 4).

^{1}H NMR ($CDCl_3$, 400 MHz): δ 2.36 (q, *J* = 8.2 Hz, 1H, C*H*CO_2H), 2.13-2.01 (m, 1H, C*H*CHCO_2H), 2.00-1.82 (m, 3H, CH_2), 1.76-1.52 (m, 3H, CH_2), 1.37-1.17 (m, 3H, CH_2), 0.92 (t, *J* = 7.4 Hz, 3H, CH_3).

^{13}C NMR ($CDCl_3$, 100 MHz): δ 183.6, 50.1, 46.4, 32.4, 30.5, 28.2, 25.0, 12.7.

HRMS (ESI, m/Z): calcd. for $C_8H_{15}O_2$ $[M+H]^+$: 143.10666, found: 143.10720.

The *ee* of this compound was determined from the corresponding *N,N*-dimethyl amide derivative. HPLC: Chiracel-OZH, *n*heptane/*i*PrOH 95:5, 0.5 mL/min., 40 °C, detection at 208 nm. Retention time (min): 16.2 (major) and 18.4 (minor).

Synthesis of chiral indole derivative

(*S*)-Ethyl 6-chloro-3-(3-methylnonanoyl)-1*H*-indole-2-carboxylate (7b)

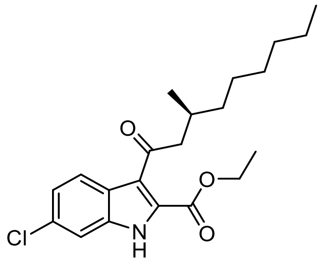

7b

The reaction was performed according to the literatre.[23] In a flame-dried Schlenk tube equipped with septum and magnetic stirring bar, **3i** (17.2 mg, 0.1 mmol, 1.0 equiv.) was dissolved in CH_2Cl_2 (1.0 mL). $SOCl_2$ (15 µL, 0.2 mmol, 2.0 equiv.) and DMF (1 drop) weres added, and the reaction mixture was stirred under nitrogen atmosphere for 1 h at RT. The solvent and the remaining $SOCl_2$ were removed under reduced pressure and the tube was evacuated and backfilled with nitrogen for three times. CH_2Cl_2 (1.0 mL) was added to afford a solution of acyl chloride of **3i** (0.1 M). In another flame-dried Schlenk tube equipped with septum and magnetic stirring bar, ethyl 6-chloro-1*H*-indole-2-carboxylate (22.4 mg, 0.1 mmol, 1.0 equiv.) was dissolved in CH_2Cl_2 (1.0 mL). $SnCl_4$ (0.3 mmol, 1.0 M in CH_2Cl_2, 3.0 equiv.) was added in a single portion *via* syringe and the mixture was stirred under nitrogen atmosphere for 30 min. at RT. The acyl chloride of **3i** (0.1 mmol, 0.1 M in CH_2Cl_2, 1.0 equiv.) was transferred to the stirring solution *via* syringe, and the reaction mixture was reflux under nitrogen atmosphere for 16 h. The reaction was cooled to RT and quenched with water (3.0 mL). The

mixture was extracted with CH_2Cl_2 (10.0 mL × 3), and the combined organic phase was dried over $MgSO_4$, filtered and evaporated on rotary evaporator. Product **7b** was obtained as a white solid after column chromatography (SiO_2, pentane:Et_2O = 8:1), and the components were visualized by 2,4-DNP staining [84% yield, 98% *ee*].

^{1}H NMR ($CDCl_3$, 400 MHz): δ 9.47 (s, 1H, N*H*), 7.83 (d, *J* = 8.7 Hz, 1H, H_{Ar}), 7.38 (d, *J* = 1.8 Hz, 1H, H_{Ar}), 7.18 (dd, *J* = 8.7, 1.8 Hz, 1H, H_{Ar}), 4.45 (q, *J* = 7.1 Hz, 2H, CO_2CH_2), 3.09 (dd, *J* = 15.8, 5.7 Hz, 1H, COC*H*H), 2.88 (dd, *J* = 15.8, 8.1 Hz, 1H, COCH*H*), 2.18-2.04 (m, 1H, C*H*), 1.42 (t, *J* = 7.1 Hz, 3H, $CO_2CH_2CH_3$), 1.38-1.14 (m, 10H, CH_2), 0.93 (d, *J* = 6.6 Hz, 3H, CH_3CH), 0.85 (t, *J* = 6.8 Hz, 3H, $CH_2CH_2CH_3$).

^{13}C NMR ($CDCl_3$, 100 MHz): δ 201.6, 160.8, 135.5, 132.2, 126.1, 125.2, 123.6, 123.5, 122.5, 111.7, 62.2, 51.8, 37.2, 31.9, 30.5, 29.6, 27.1, 22.7, 20.1, 14.3, 14.2.

HRMS (ESI, m/Z): calcd. for $C_{21}H_{29}ClNO_3$ $[M+H]^+$: 378.18305, found: 378.18322.

HPLC: Chiracel-ASH, *n*heptane/*i*PrOH 98:2, 0.5 mL/min., 40 °C, detection at 223 nm. Retention time (min): 29.9 (major) and 33.7 (minor).

Synthesis of *t*BuMe$_2$Si-esters as the references

t-Butyldimethylsilyl hex-2-enoate

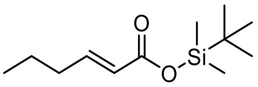

In a flame-dried Schlenk tube equipped with septum and magnetic stirring bar, **1a** (22.8 mg, 0.2 mmol, 1.0 equiv.) was dissolved in *t*BuOMe (2.0 mL) and cooled down to −20 °C. *t*BuMe$_2$SiOTf (101 μL, 0.44 mmol, 2.2 equiv.) was added. After 20 min., EtMgBr (0.2 mmol, 3.0 M in Et_2O, 1.0 equiv.) was added dropwise, and the reaction mixture was allowed to stir for 5 min under nitrogen atmosphere. The reaction was quenched with saturated $NaHCO_3$ aqueous solution (3.0 mL) and warmed to RT. The mixture was extracted with Et_2O (10.0 mL × 3). The combined organic phase was dried over $MgSO_4$, filtered and evaporated on rotary evaporator. The product was obtained as a colorless oil after column chromatography (SiO_2, pentane:Et_2O = 100:1) [19% yield].

^{1}H NMR ($CDCl_3$, 400 MHz): δ 6.91 (dt, *J* = 15.5, 6.9 Hz, 1H, CH_2C*H*=CH), 5.78 (dt, *J* = 15.5, 1.6 Hz, 1H, CH_2CH=C*H*), 2.17 (m, *J* = 7.2, 1.6 Hz, 2H, CH_2CH=CH), 1.49 (m, *J* = 7.4 Hz, 2H, CH_3CH_2), 0.95 (s, 9H, $SiCCH_3$), 0.94 (t, *J* = 7.3, 3H, CH_3CH_2), 0.29 (s, 6H, $SiCH_3$).

^{13}C NMR ($CDCl_3$, 100 MHz): δ 166.8, 149.8, 123.4, 34.2, 25.8, 21.5, 17.9, 13.8, −4.6.

HRMS (ESI, m/Z): calcd. for $C_{12}H_{25}O_2Si$ $[M+H]^+$: 299.16183, found: 299.16199.

t-Butyldimethylsilyl but-2-enoate[24]

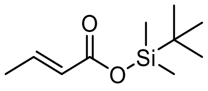

In a flame-dried Schlenk tube equipped with septum and magnetic stirring bar, **1b** (17.2 mg, 0.2 mmol, 1.0 equiv.) was dissolved in *t*BuOMe (2.0 mL) and cooled to −20 °C. *t*$BuMe_2SiOTf$ (101 μL, 0.44 mmol, 2.2 equiv.) was added. After 20 min., EtMgBr (0.2 mmol, 3.0 M in Et_2O, 1.0 equiv.) was added dropwise, and the reaction mixture was allowed to stir for 5 min under nitrogen atmosphere. The reaction was quenched with saturated $NaHCO_3$ aqueous solution (3.0 mL) and warmed to RT. The mixture was extracted with Et_2O (10.0 mL × 3). The combined organic phase was dried over $MgSO_4$, filtered and evaporated on rotary evaporator. The product was obtained as a colorless oil after column chromatography (SiO_2, pentane:Et_2O = 100:1) [13% yield].

^{1}H NMR ($CDCl_3$, 400 MHz): δ 6.92 (dq, J = 15.4, 6.9 Hz, 1H, CH_3CH=CH), 5.81 (dt, J = 15.4, 1.7 Hz, 1H, CH_3CH=C*H*), 1.87 (dd, J = 6.9, 1.7 Hz, 3H, CH_3CH=CH), 0.95 (s, 9H, $SiCCH_3$), 0.28 (s, 6H, $SiCH_3$).

^{13}C NMR ($CDCl_3$, 100 MHz): δ 166.6, 145.1, 124.8, 25.8, 18.0, 17.9, −4.6.

Racemic t-Butyldimethylsilyl 3-ethylhexanoate

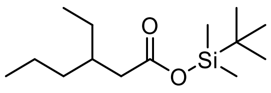

In a flame-dried Schlenk tube equipped with septum and magnetic stirring bar, **1a** (22.8 mg, 0.2 mmol, 1.0 equiv.), CuBr·SMe_2 (2.06 mg, 0.01 mmol, 5 mol%) and THF (2.0 mL) were added. The mixture was cooled to −20 °C and *t*$BuMe_2SiOTf$ (138 μL, 0.6 mmol, 3.0 equiv.) was added. After 20 min., EtMgBr (0.6 mmol, 3.0 M in Et_2O, 3.0 equiv.) was added dropwise, and the reaction mixture was allowed to stir for 2 h. The reaction was quenched with saturated $NaHCO_3$ aqueous solution (3.0 mL) and warmed to RT. The mixture was extracted with Et_2O (10.0 mL × 3). The combined organic phase was dried over $MgSO_4$, filtered and evaporated on rotary evaporator. The product was obtained as a colorless oil after column chromatography (SiO_2, pentane:Et_2O = 100:1) [39% yield].

^{1}H NMR ($CDCl_3$, 400 MHz): δ 2.23 (d, J = 6.8 Hz, 2H, CH_2CO_2Si), 1.84-1.72 (m, 1H, C*H*), 1.43-1.18 (m, 6H, CH_2), 0.93 (s, 9H, $SiCCH_3$), 0.91-0.85 (m, 6H, CH_3CH_2), 0.26 (s, 6H, $SiCH_3$).

^{13}C NMR ($CDCl_3$, 100 MHz): δ 174.4, 40.8, 36.6, 35.9, 26.5, 25.7, 19.9, 17.7, 14.5, 11.0, −4.7.

HRMS (ESI, m/Z): calcd. for $C_{14}H_{31}O_2Si$ $[M+H]^+$: 259.20878, found: 259.20940.

Isolation of the *t*BuMe$_2$Si ester of 1a and testing of its reactivity in Cu-catalyzed asymmetric conjugate addition

In a flame-dried Schlenk tube equipped with septum and magnetic stirring bar, **1a** (22.8 mg, 0.2 mmol, 1.0 equiv.) was dissolved in *t*BuOMe (2.0 mL) and cooled down to −78 °C. *t*BuMe$_2$SiOTf (101 μL, 0.44 mmol, 2.2 equiv.) was added. After 20 min., EtMgBr (0.2 mmol, 3.0 M in Et_2O, 1.0 equiv.) was added dropwise, and the reaction mixture was allowed to stir for 5 min under nitrogen atmosphere. The reaction was quenched with HCl aqueous solution (2.0 mL, 1.0 M) and warmed to RT. The mixture was extracted with Et_2O (10.0 mL × 3). The combined organic phase was dried over $MgSO_4$, filtered and evaporated on rotary evaporator. The product was obtained as a colorless oil after column chromatography (SiO_2, pentane:Et_2O = 100:1) [22% conversion, 10% yield].

In a flame-dried Schlenk tube equipped with septum and magnetic stirring bar, the *t*BuMe$_2$Si ester of **1a** (22.8 mg, 0.1 mmol, 1.0 equiv.), CuBr·SMe$_2$ (1.03 mg, 0.005 mmol, 5 mol%) and ligand (*R*)-**L4** (4.07 mg, 0.006 mmol, 6 mol%) were dissolved in *t*BuOMe (1.0 mL) and stirred under nitrogen atmosphere for 20 min. at RT. The mixture was cooled to −78 °C and EtMgBr (0.15 mmol, 3.0 M in Et_2O, 1.5 equiv.) was added dropwise. The reaction mixture was allowed to stir for 16 h. The reaction was quenched with HCl aqueous solution (1.0 mL, 1.0 M) and warmed to RT. The mixture was extracted with CH_2Cl_2 (5.0 mL × 3). The combined organic phase was dried over $MgSO_4$, filtered and evaporated on rotary evaporator. [**2a**:*t*BuMe$_2$Si ester of **1a** = 12:88, 99% total conversion, 98% *ee*].

General Procedure for ^{1}H NMR spectroscopy based mechanistic studies

Formation of carboxylic acid 1b-*t*BuMe$_2$SiOTf complex

1b (4.3 mg, 0.05 mmol, 1.0 equiv.) was dissolved in CD_2Cl_2 (0.5 mL) in a dry NMR tube under nitrogen atmosphere and cooled down to −78 °C. *t*BuMe$_2$SiOTf (25 μL, 0.11 mmol, 2.2 equiv.) was added and the resulting mixture was measured by ^{1}H NMR spectroscopy at −55 °C (see Fig. 3 in the main text).

***In situ* formation of *t*BuMe$_2$Si ester of 1b**

1b (4.3 mg, 0.05 mmol, 1.0 equiv.) was dissolved in CD_2Cl_2 (0.5 mL) in in a dry NMR tube under nitrogen atmosphere. The solution was cooled down to −78 °C and *t*BuMe$_2$SiOTf (25 μL, 0.11 mmol, 2.2 equiv.) was added. After 20 min., MeMgBr (0.05 mmol, 3.0 M in Et_2O, 1.0 equiv.) was added dropwise and the resulting mixture was measured by ^{1}H NMR spectroscopy at −55 °C (see Fig. 3 in the main text).

General procedure for Cu-catalyzed asymmetric conjugate addition of EtMgBr to carboxylate salts formed first by deprotonation of 1a by base.

In a flame-dried Schlenk tube equipped with septum and magnetic stirring bar, **1a** (22.8 mg, 0.2 mmol, 1.0 equiv.), CuBr·SMe_2 (2.06 mg, 0.01 mmol, 5 mol%) and ligand (*R*)-**L4** (8.15 mg, 0.012 mmol, 6 mol%) were dissolved in *t*BuOMe (2.0 mL) and stirred under nitrogen atmosphere for 20 min. at RT. The mixture was cooled to −78 °C and the base (0.2 mmol, 1.0 equiv.) was added (when NaH was used as the base, it should be added and stirred for 1 h at RT before the mixture is cooled to −78 °C because of its low solubility in *t*BuOMe). After 5 min., Me_3SiOTf was added, then the mixture was allowed to stir for 5 min before EtMgBr was added dropwise. The reaction mixture was allowed to stir for 16 h. The reaction was quenched with HCl aqueous solution (2.0 mL, 1.0 M) and warmed to RT. The mixture was extracted with CH_2Cl_2 (10.0 mL × 3). The combined organic phase was dried over $MgSO_4$, filtered and evaporated on rotary evaporator (see Supplementary Table 3).

General procedure for Cu-catalyzed asymmetric conjugate addition of EtMgBr to Li carboxylate A-Li with different electrophiles

In a flame-dried Schlenk tube equipped with septum and magnetic stirring bar, **1a** (22.8 mg, 0.2 mmol, 1.0 equiv.), CuBr·SMe_2 (2.06 mg, 0.01 mmol, 5 mol%) and ligand (*R*)-**L4** (8.15 mg, 0.012 mmol, 6 mol%) were dissolved in *t*BuOMe (2.0 mL) and stirred under nitrogen atmosphere for 20 min. at RT. The mixture was cooled to −78 °C and *n*BuLi (0.2 mmol, 2.5 M in hexane, 1.0 equiv.) was added. After 5 min., the electrophile was added, and the mixture was allowed to stir for 5 min before EtMgBr (0.5 mmol, 3.0 M in Et_2O, 2.5 equiv.) was added dropwise. The reaction mixture was allowed to stir for 16 h. The reaction was quenched with HCl aqueous solution (2.0 mL, 1.0 M) and warmed to RT. The mixture was extracted with CH_2Cl_2 (10.0 mL × 3). The combined organic phase was dried over $MgSO_4$, filtered and evaporated on rotary evaporator (see Supplementary Table 3)

General procedure for the isolation and measurement of the solubility of Mg-, Li- and Na- carboxylates A-Mg, A-Li and A-Na in *t*BuOMe.

In a flame-dried Schlenk tube equipped with septum and magnetic stirring bar, **1a** (144.1 mg, 1.0 mmol, 1.0 equiv.) was dissolved in *t*BuOMe (10.0 mL) at RT. EtMgBr (1.0 mmol, 3.0 M in Et_2O, 1.0 equiv.) or *n*BuLi (1.0 mmol, 2.5 M in hexane, 1.0 equiv.) or NaH (1.0 mmol, 60% in mineral oil, 1.0 equiv.) were added. The reaction mixture was allowed to stir for overnight at RT and the metal carboxylate has precipitated. The precipitate was centrifuged and washed with *t*BuOMe (10.0 mL × 3). The precipitate was dried *in vacuo* during overnight to give the metal carboxylates **A-Mg** or **A-Li** or **A-Na** as the white solid respectively.

The metal carboxylates **A-Mg** or **A-Li** or **A-Na** (0.1 mmol) was added to 50 mL *t*BuOMe, and the mixture was refluxed for 3 h, followed by addition of 1,3,5-trimethoxybenzene (8.4 mg, 0.05 mmol) as the internal standard. The mixture was filtered and the filtrate was evaporated on rotary evaporator. DMSO-d_6 was added and the corresponding ^{1}H NMR spectra of the samples were recorded (see Supplementary Figures 1-3). The solubility of **A-Mg**: 0.4318 mM, **A-Na**: 0.1225 mM. No peaks of **A-Li** were observed because the solubility is under NMR detection limit.

Magnesium bromide hex-2-enoate (A-Mg)

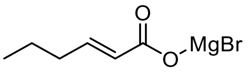

A-Mg

^{1}H NMR (DMSO-d_6, 400 MHz): δ 6.60 (dt, J = 15.5, 7.0 Hz, 1H, CH_2CH=CH), 5.76-5.69 (m, 1H, CH_2CH=CH), 2.13-2.04 (m, 2H, CH_2CH=CH), 1.46-1.34 (m, 2H, CH_3CH_2), 0.88 (t, J = 7.4 Hz, 3H, CH_3).

Lithium hex-2-enoate (A-Li)

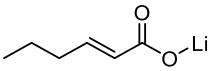

A-Li

^{1}H NMR (DMSO-d_6, 400 MHz): δ 6.20 (dt, J = 14.6, 6.9 Hz, 1H, CH_2CH=CH), 5.58 (dt, J = 15.3, 1.5 Hz, 1H, CH_2CH=CH), 2.02-1.94 (m, 2H, CH_2CH=CH), 1.42-1.30 (m, 2H, CH_3CH_2), 0.86 (t, J = 7.4 Hz, 3H, CH_3).

Sodium hex-2-enoate (A-Na)

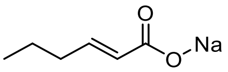

A-Na

^{1}H NMR (DMSO-d_6, 400 MHz): δ 6.24-6.13 (m, 1H, CH_2CH=CH), 5.58 (dt, J = 15.4, 1.4 Hz, 1H, CH_2CH=CH), 2.02-1.93 (m, 2H, CH_2CH=CH), 1.42-1.30 (m, 2H, CH_3CH_2), 0.86 (t, J = 7.3 Hz, 3H, CH_3).

Supplementary Figures

Supplementary Figures 1-3 are for determining solubilities of Mg, Li- and Na- carboxylates (**A-Mg**, **A-Li** and **A-Na** respectively) in *t*BuOMe. Solubilities are: **A-Mg**: 0.4318 mM, **A-Na**: 0.1225 mM. No peaks for **A-Li** were observed in ^{1}H NMR (because the solubility is under NMR detection limit).

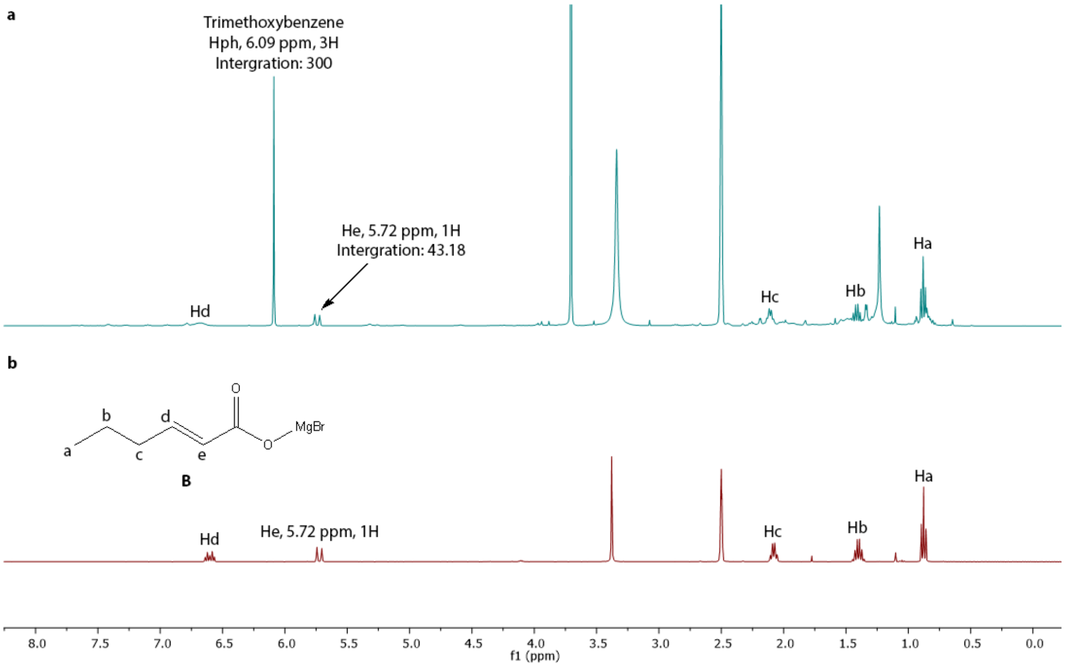

Supplementary Figure 1. a, Measurement of the solubility of Mg carboxylate **A-Mg** in *t*BuOMe. ^{1}H NMR spectra were obtained after evaporating *t*BuOMe and dissolving the residue in DMSO-d_6 using 1,3,5-trimethoxybenzene as the internal standard. **b**, ^{1}H NMR spectrum of Mg carboxylate **A-Mg** in DMSO-d_6 as the reference.

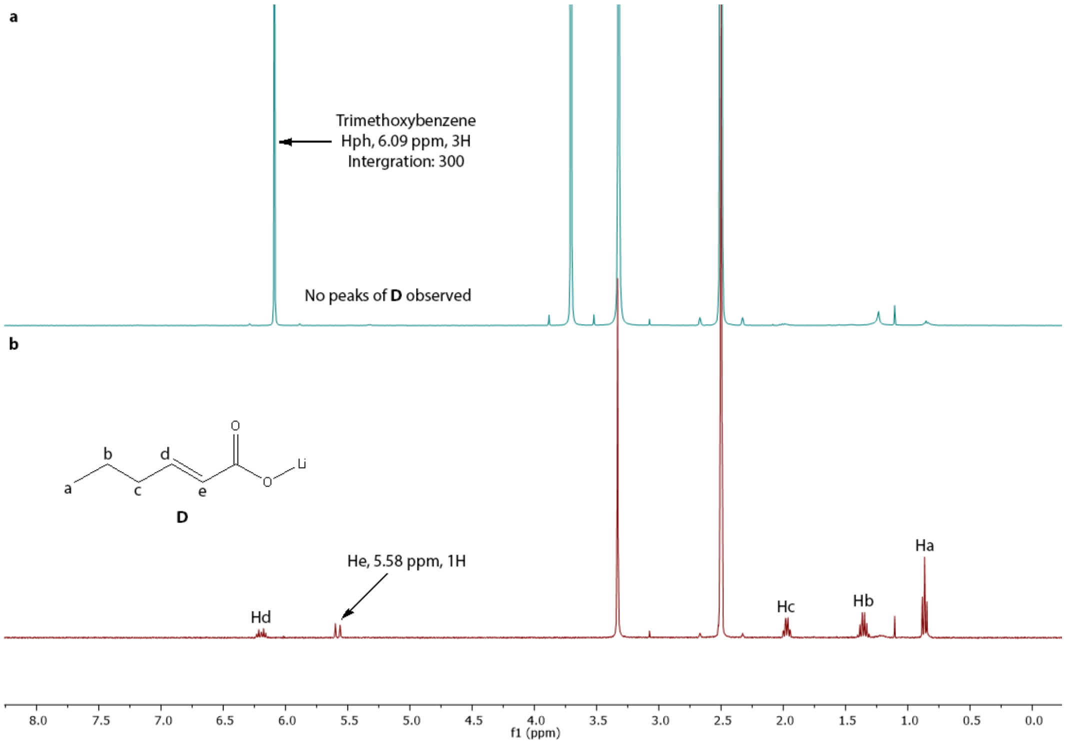

Supplementary Figure 2. **a,** Measurement of the solubility of Li carboxylate **A-Li** in *t*BuOMe. ^{1}H NMR spectra were obtained after evaporating *t*BuOMe and dissolving the residue in DMSO-d_6 using 1,3,5-trimethoxybenzene as the internal standard. **b**, ^{1}H NMR spectrum of Li carboxylate **A-Li** in DMSO-d_6 as the reference.

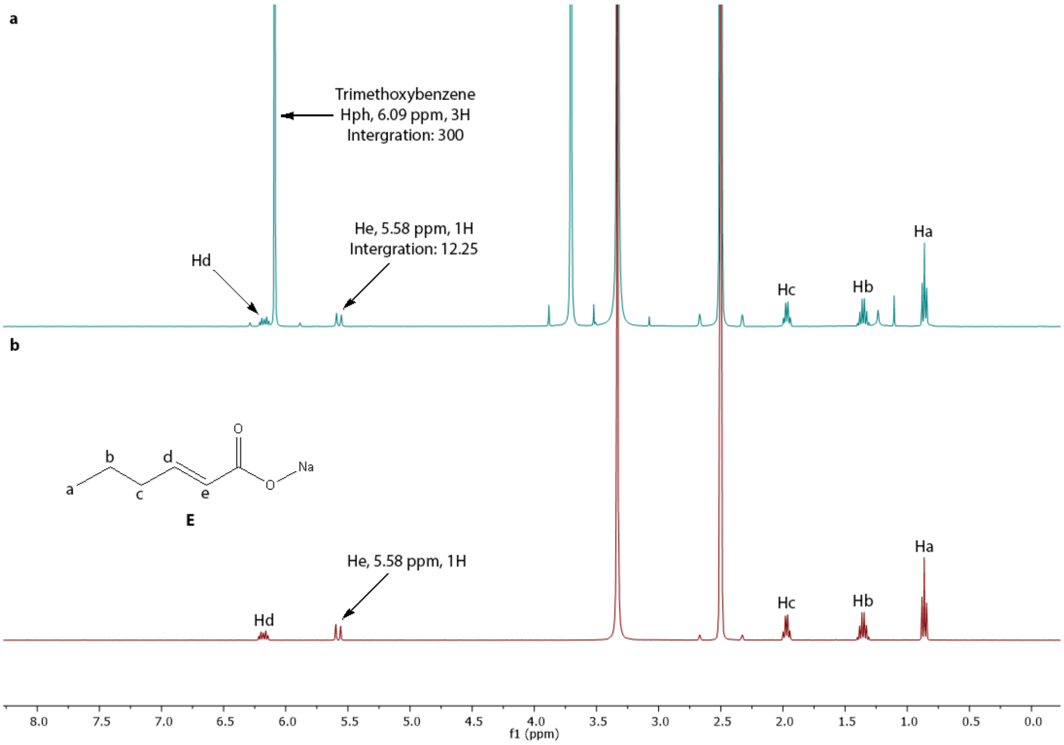

Supplementary Figure 3. **a**, Measurement of the solubility of Na carboxylate **A-Na** in *t*BuOMe. ^{1}H NMR spectra were obtained after evaporating *t*BuOMe and dissolving the residue in DMSO-d_6 using 1,3,5-trimethoxybenzene as the internal standard. **b**, ^{1}H NMR spectrum of Na carboxylate **A-Na** in DMSO-d_6 as the reference.

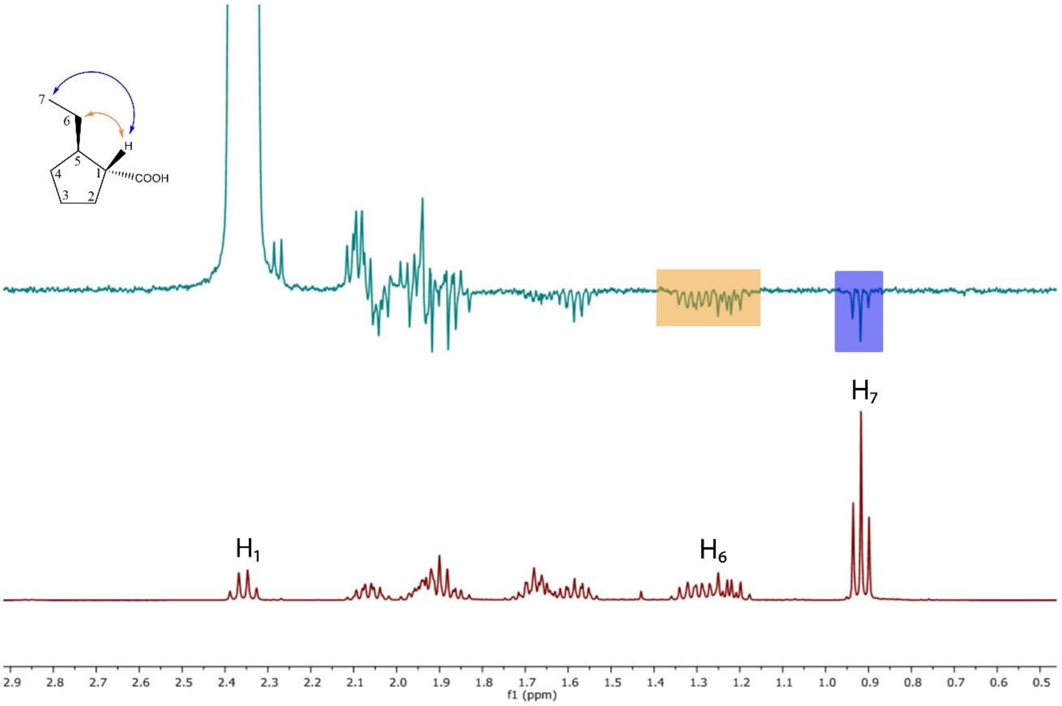

Supplementary Figure 4. ^{1}H NMR and 1D NOE experiment of **6.** Selective irradiation on H_1 showed NOE with ethyl moiety (H_6 and H_7, highlighted) which are positioned on the same side of the ring.

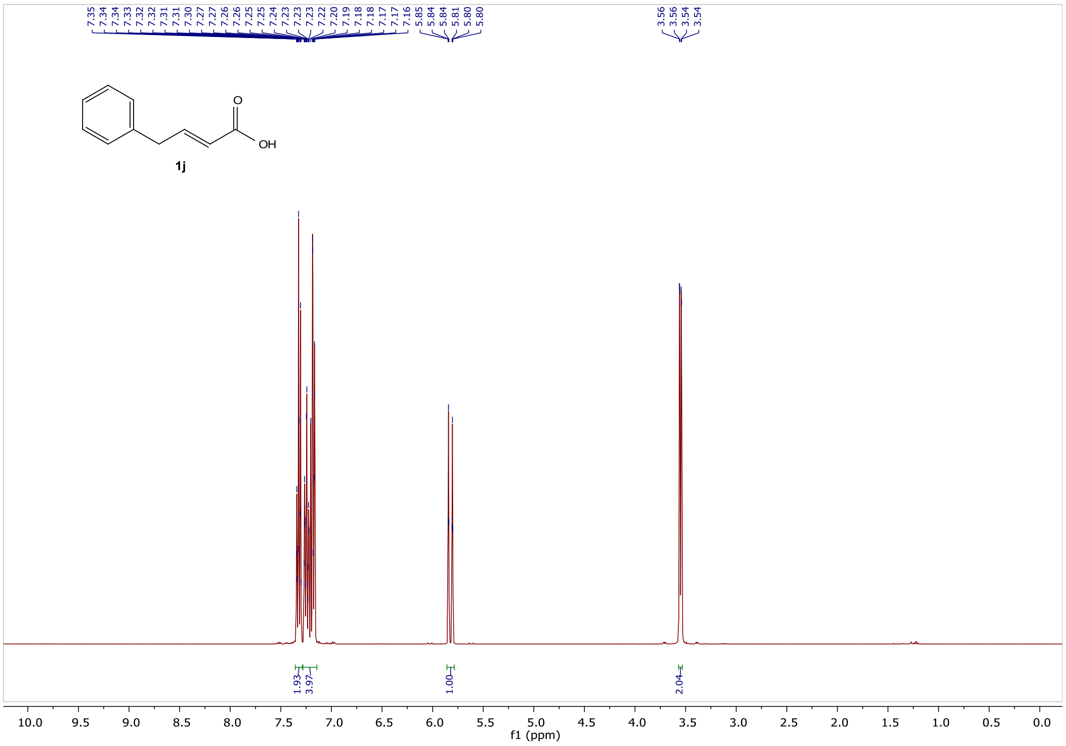

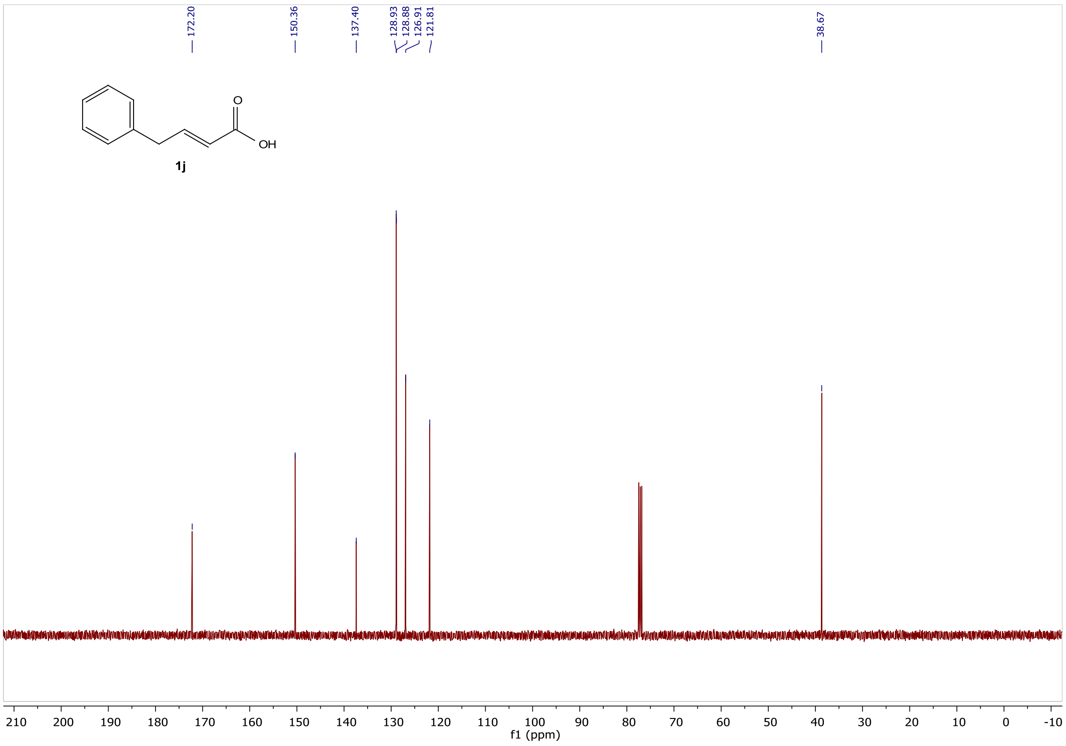

Supplementary Figure 5. NMR spectra of (*E*)-4-phenylbut-2-enoic acid (1j)

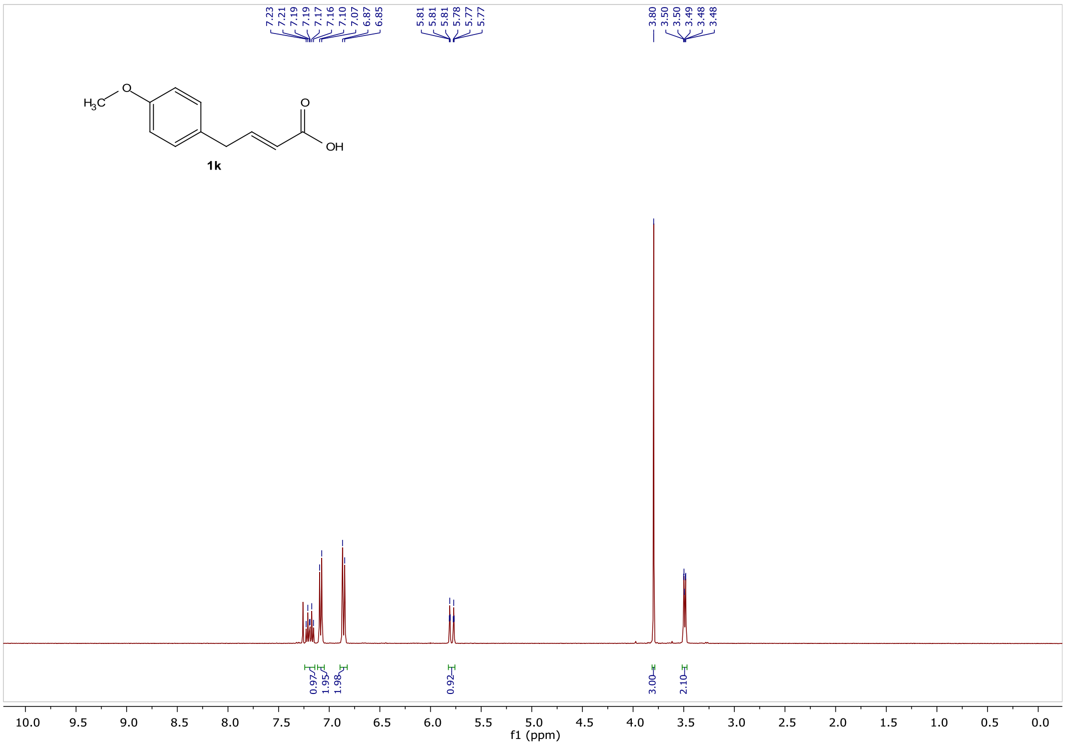

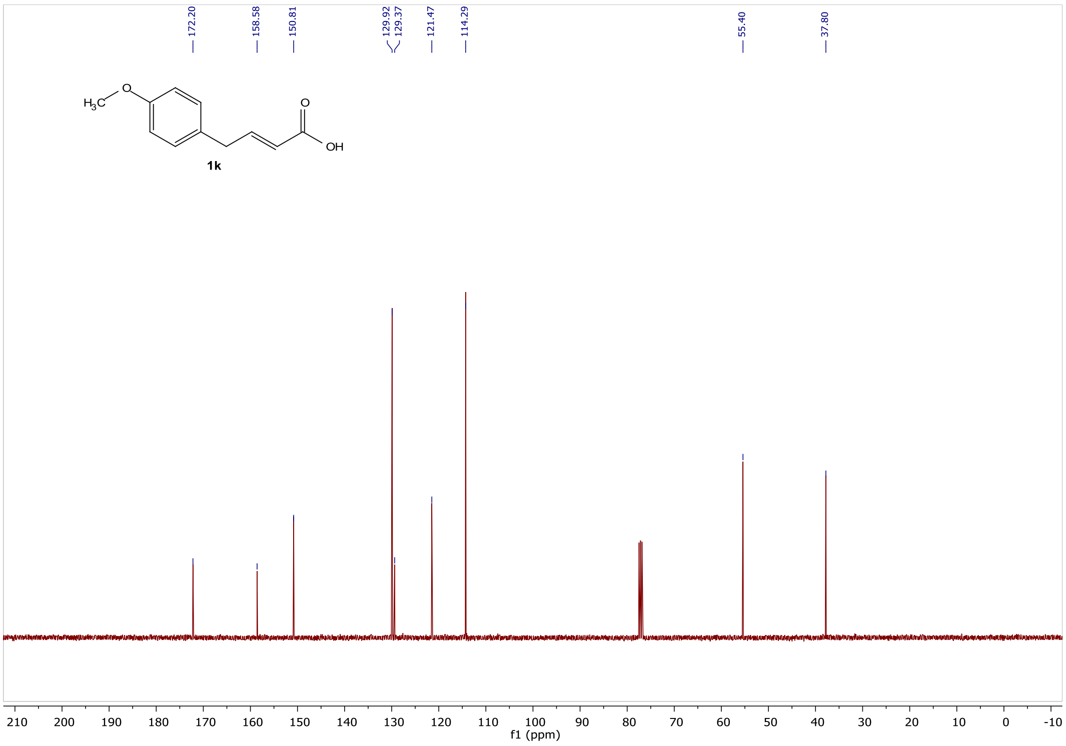

Supplementary Figure 6. NMR spectra of (*E*)-4-(4-methoxyphenyl)but-2-enoic acid (1k)

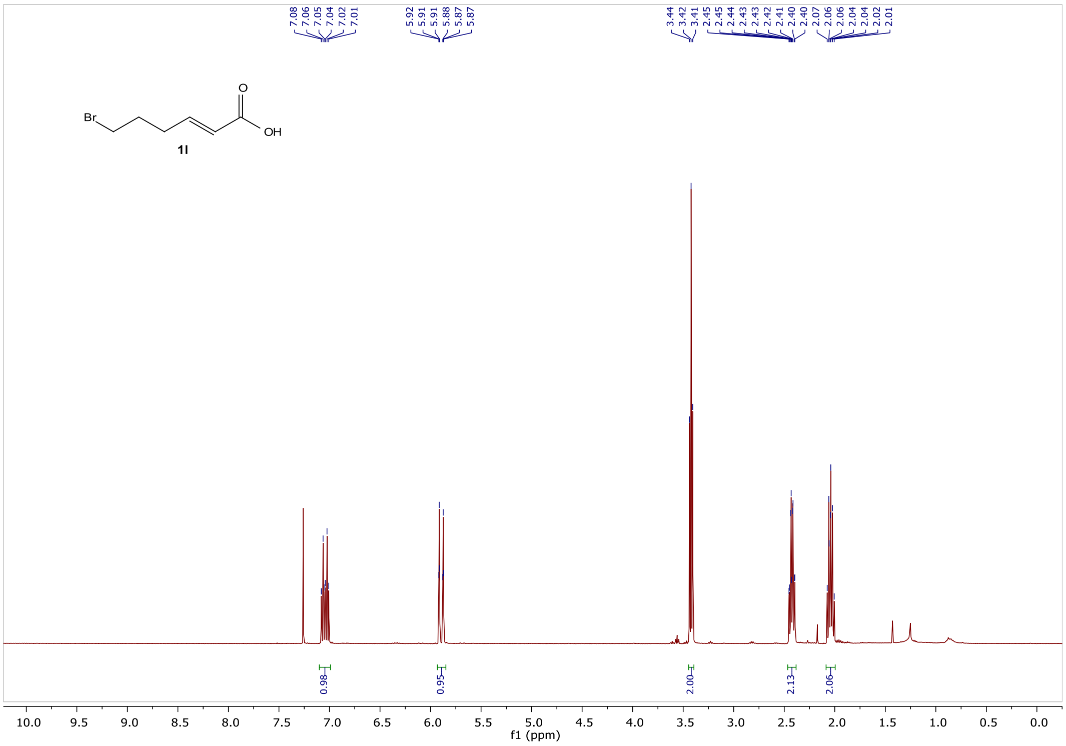

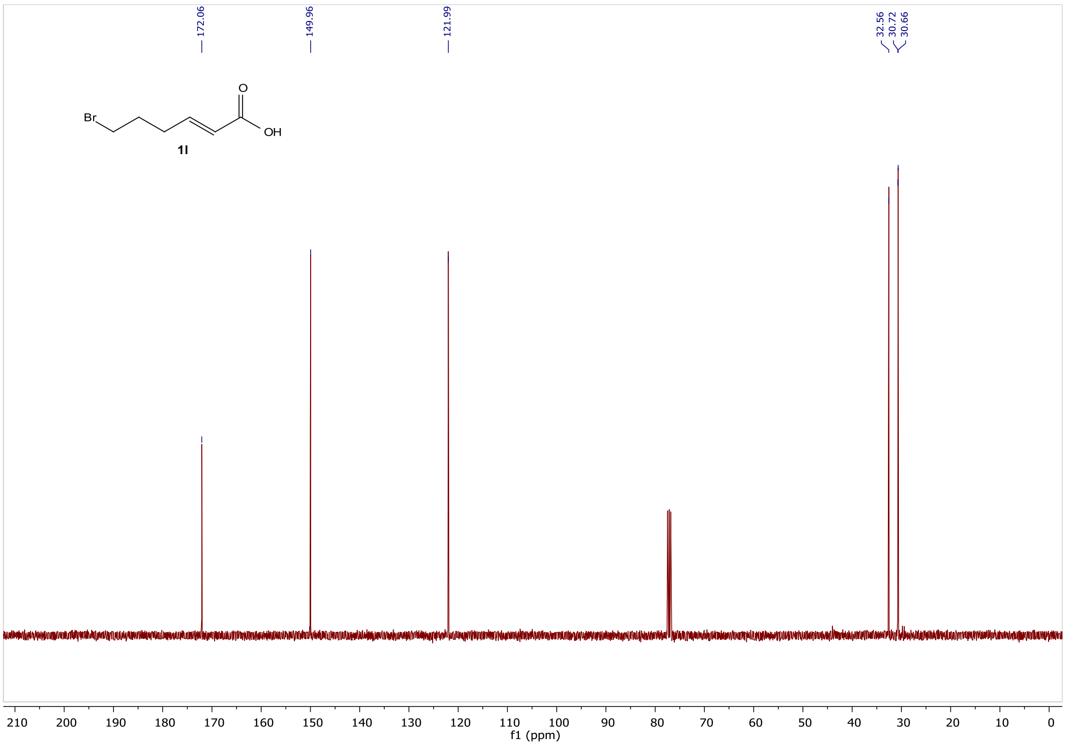

Supplementary Figure 7. NMR spectra of (*E*)-6-bromo-hex-2-enoic acid (1l)

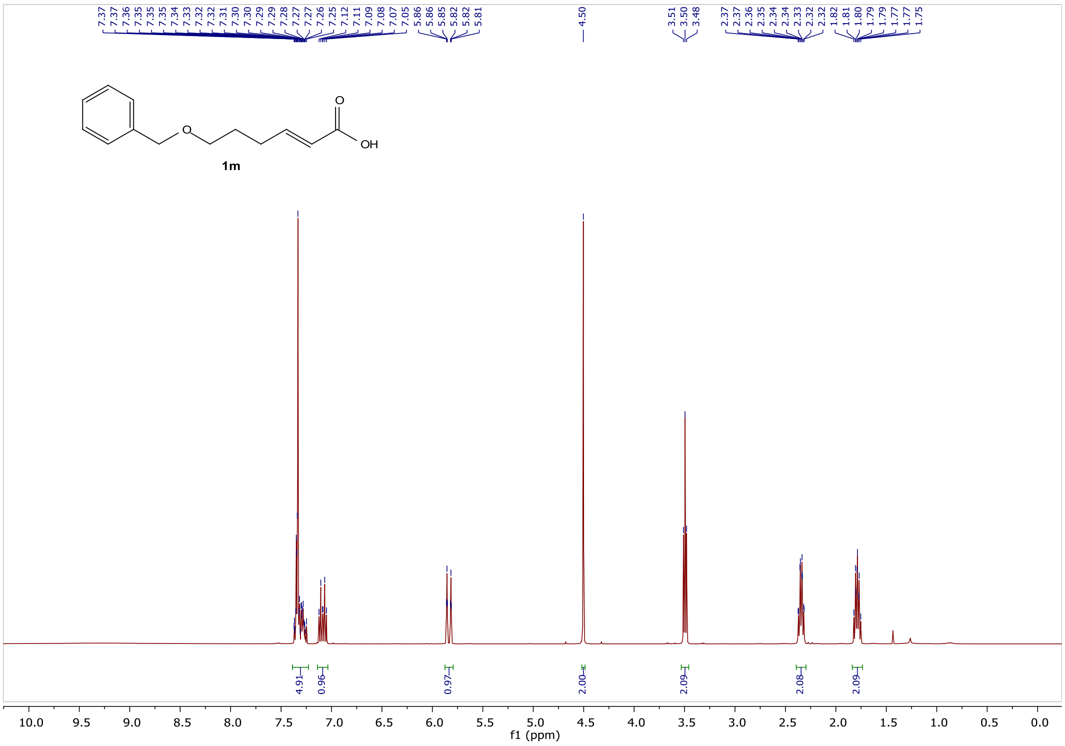

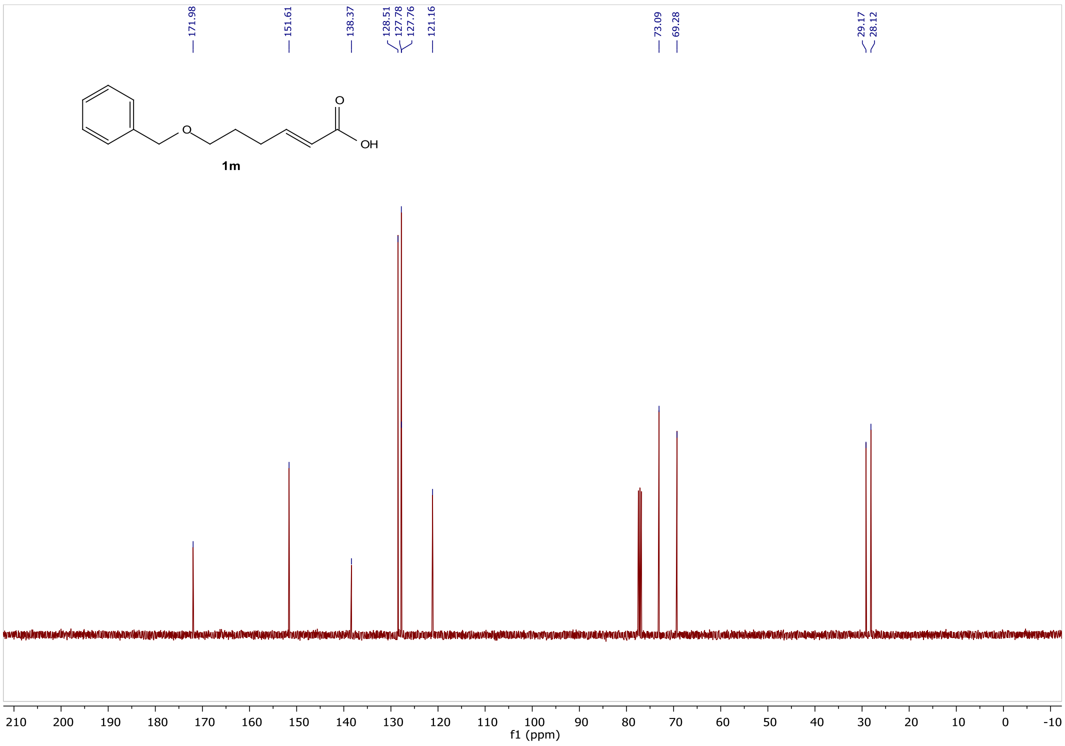

Supplementary Figure 8. NMR spectra of (*E*)-6-(benzyloxy)hex-2-enoic acid (1m)

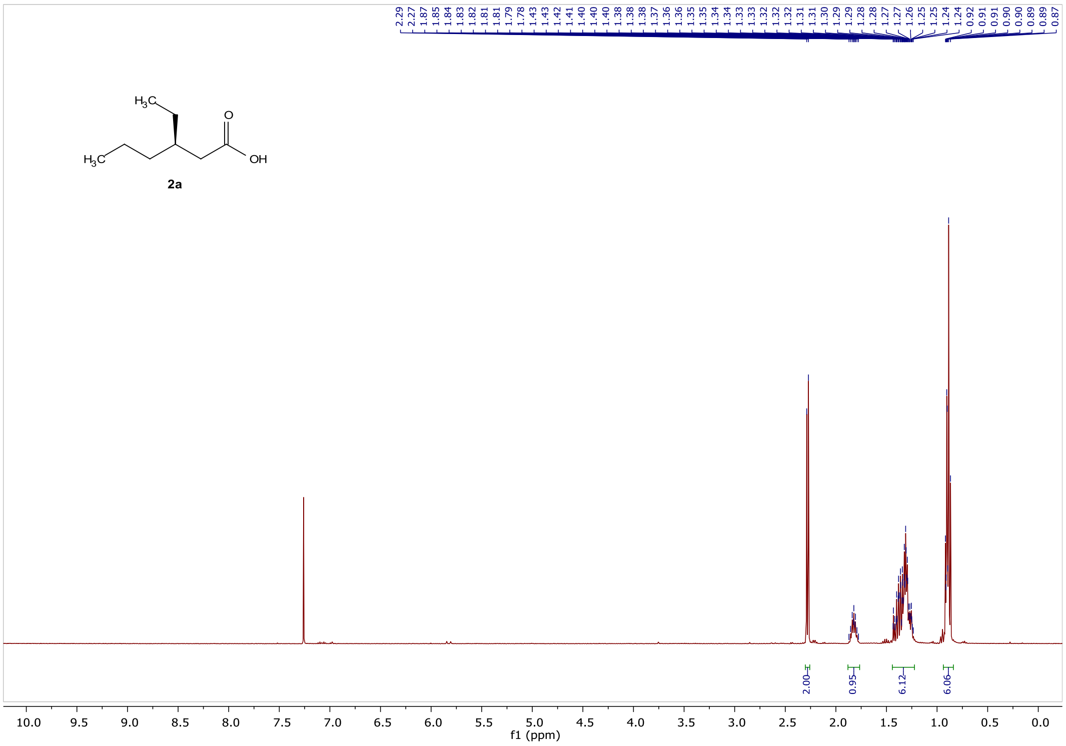

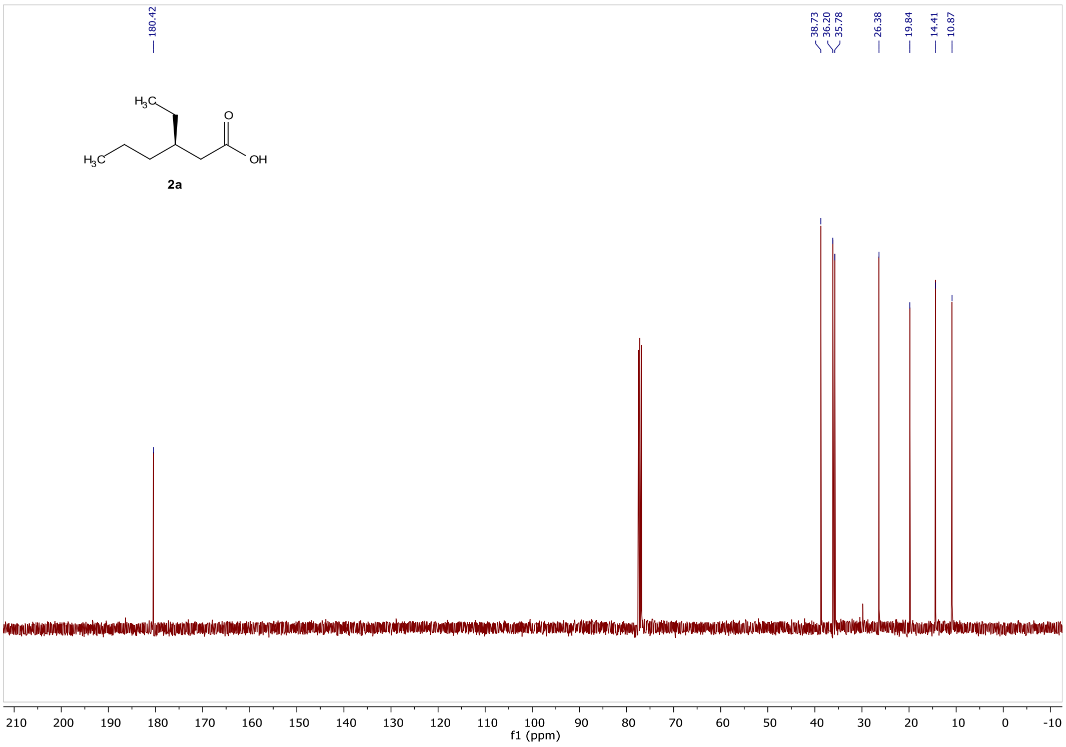

Supplementary Figure 9. NMR spectra of (*R*)-3-ethylhexanoic acid (2a)

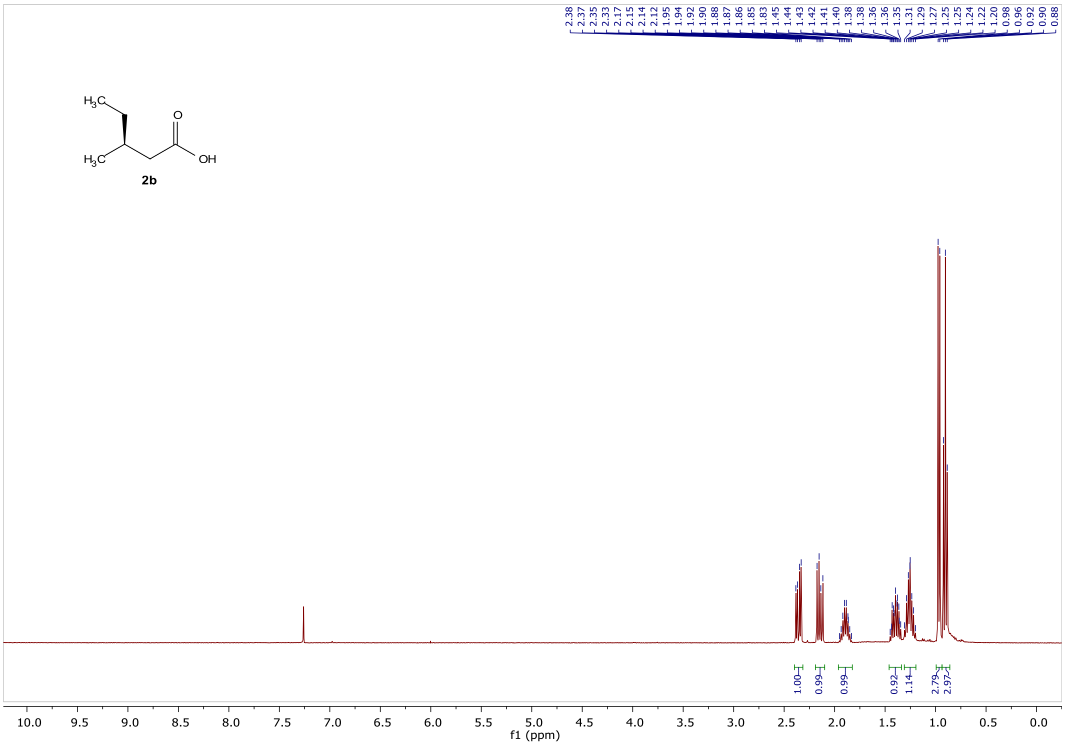

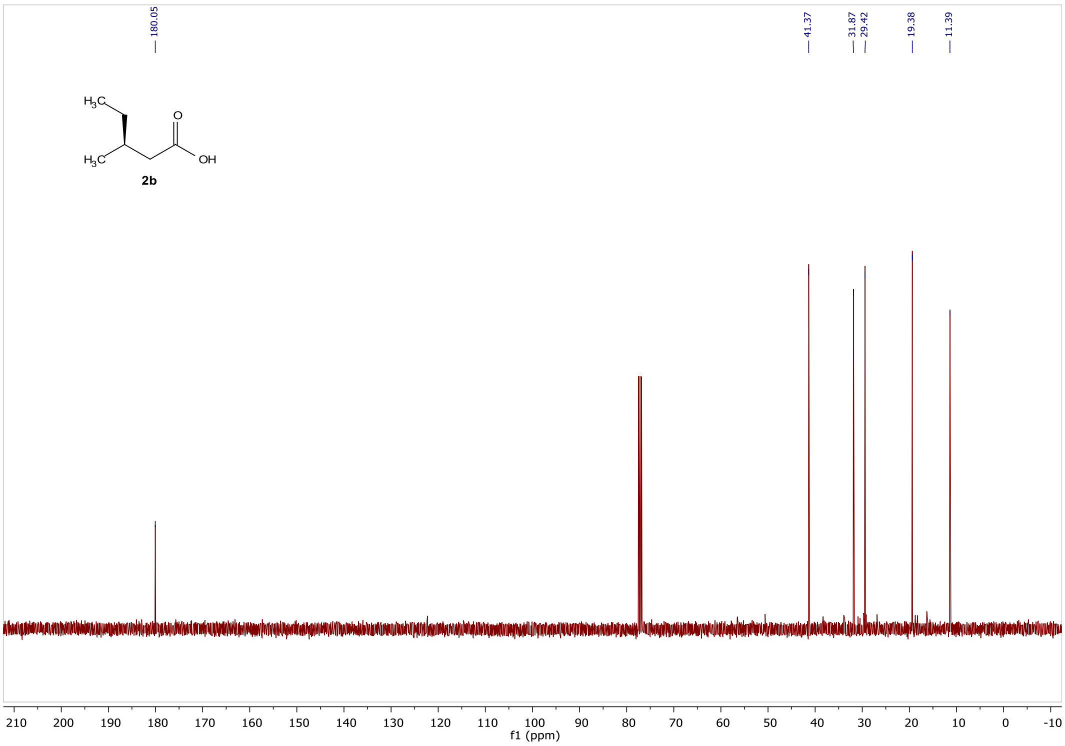

Supplementary Figure 10. NMR spectra of (*S*)-3-methylpentanoic acid (2b)

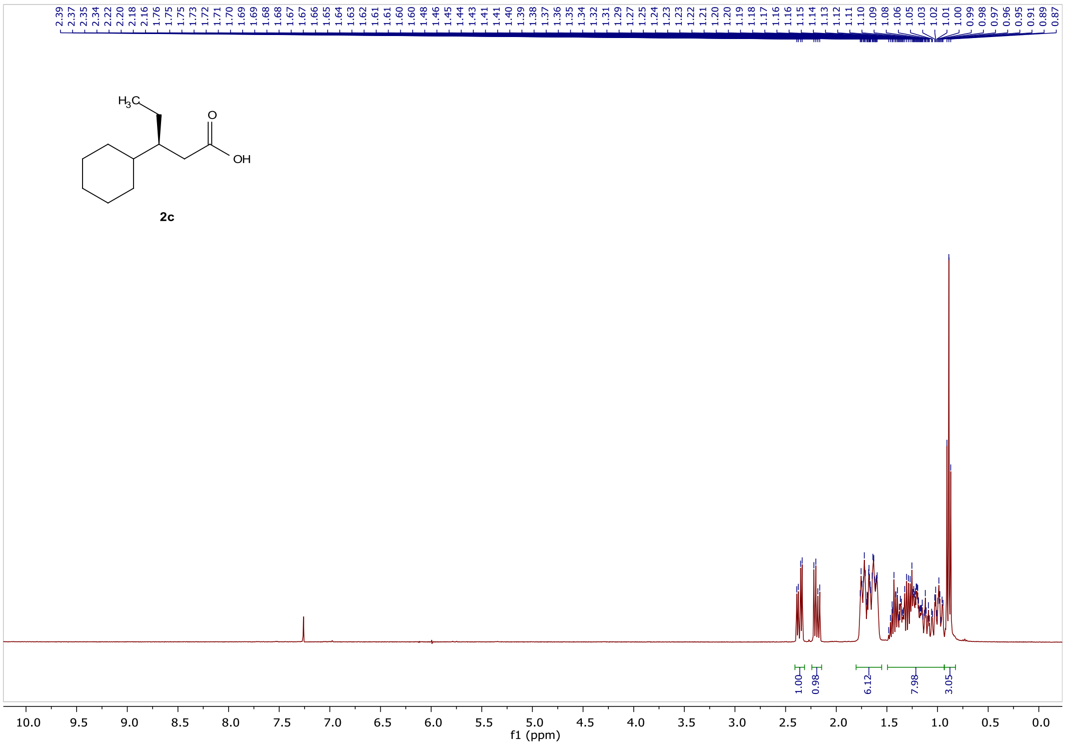

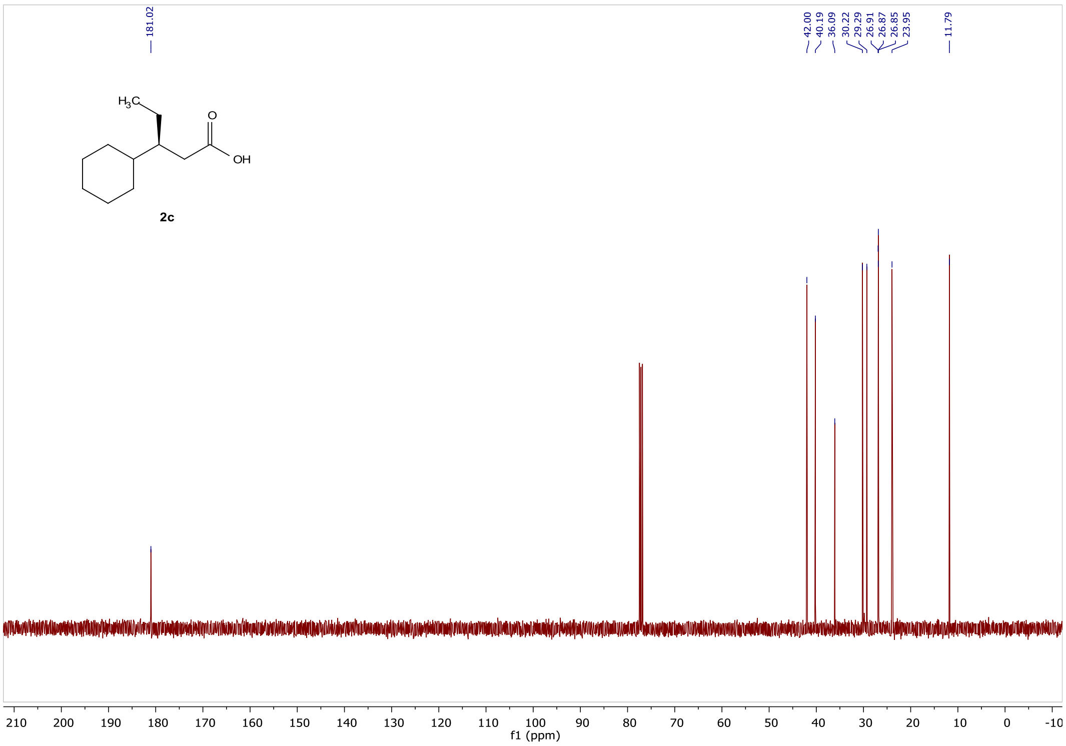

Supplementary Figure 11. NMR spectra of (*S*)-3-cyclohexylpentanoic acid (2c)

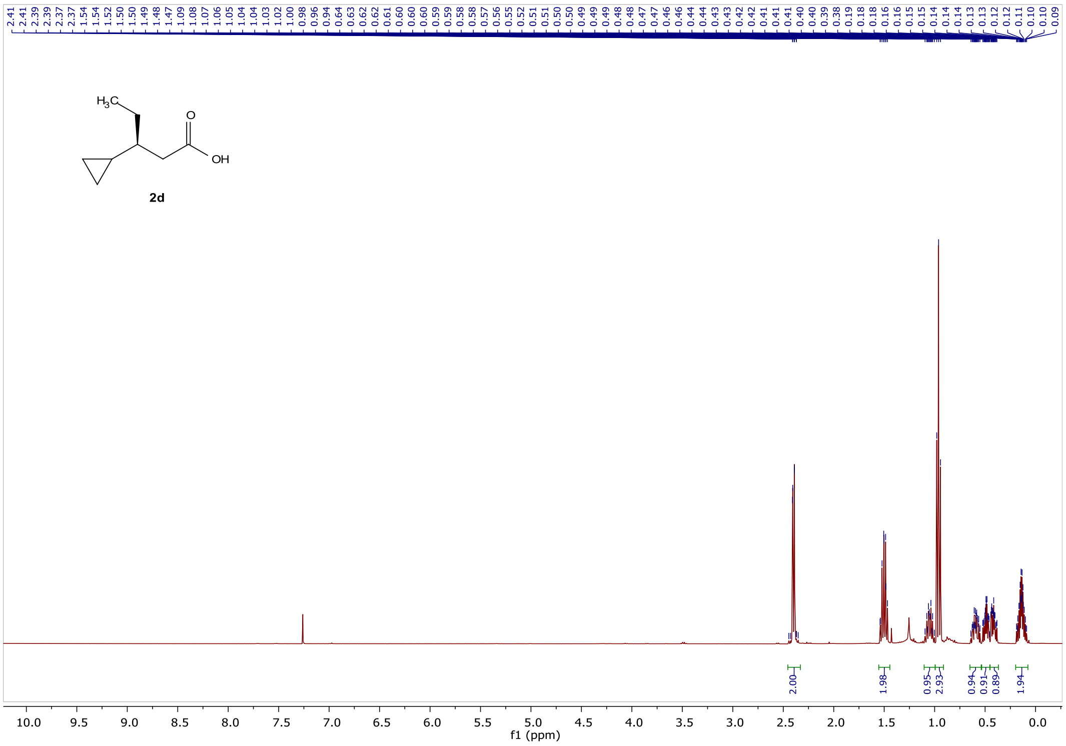

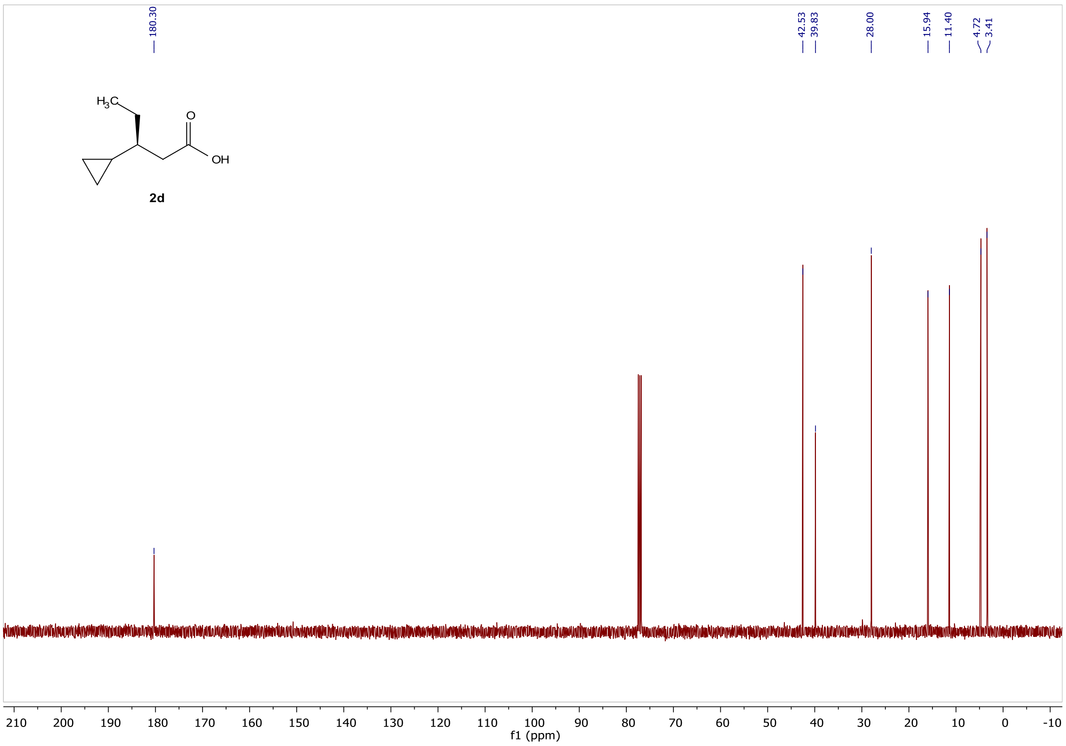

Supplementary Figure 12. NMR spectra of (*S*)-3-cyclopropylpentanoic acid (2d)

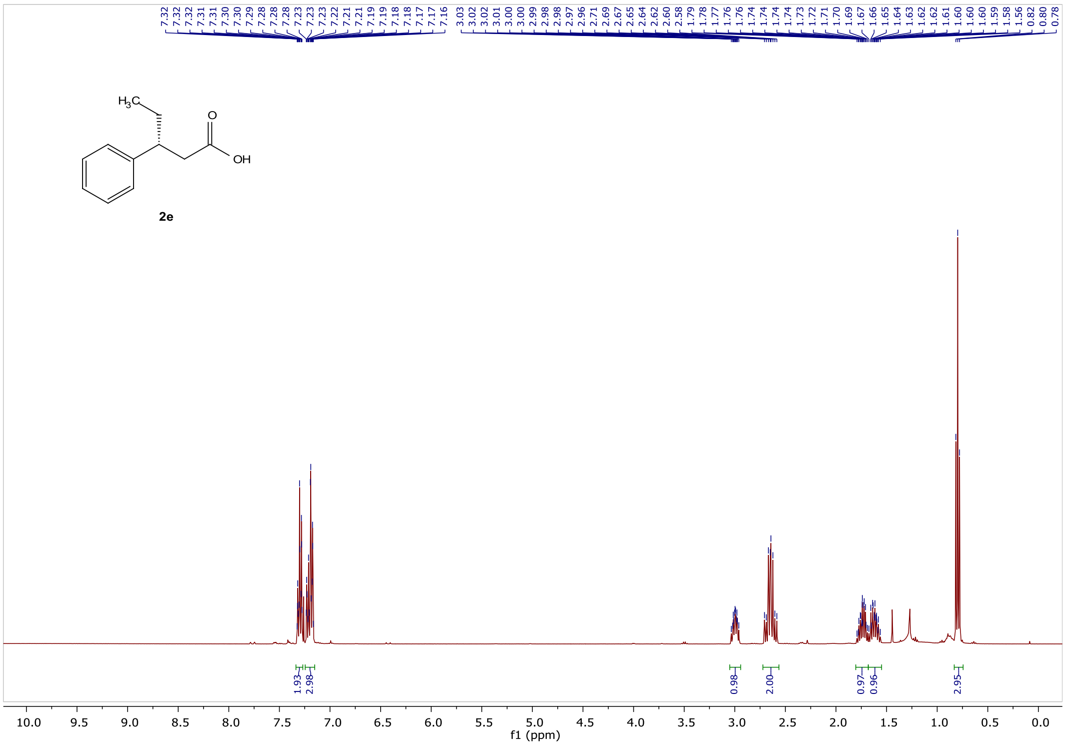

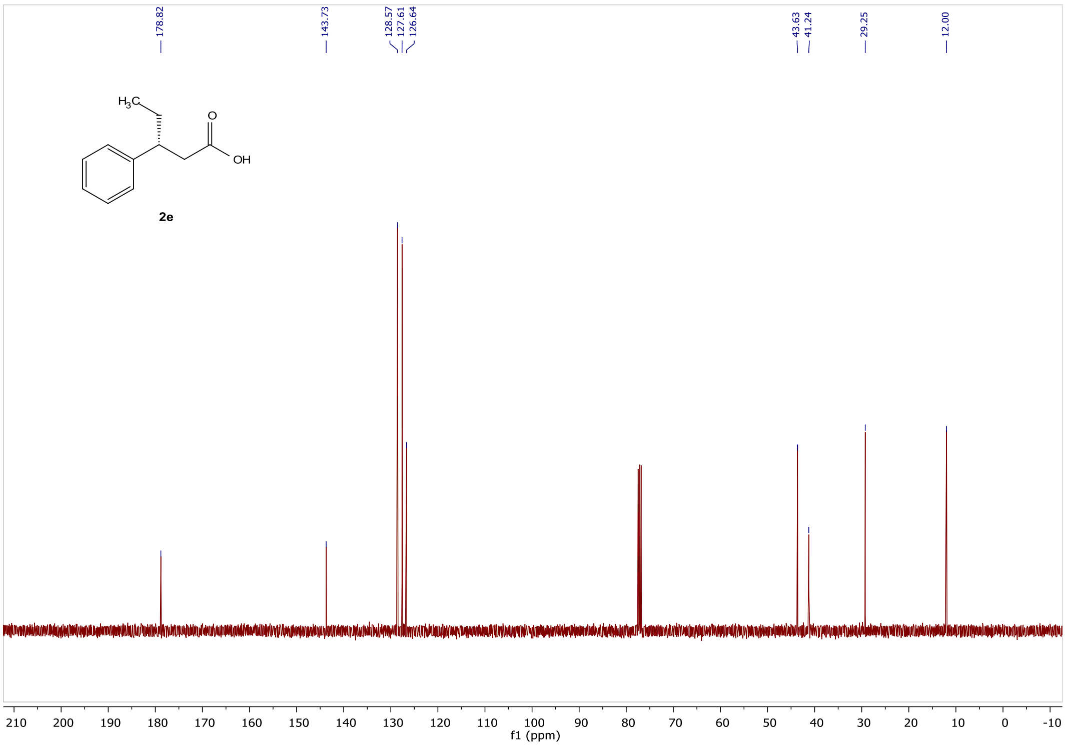

Supplementary Figure 13. NMR spectra of (*R*)-3-phenylpentanoic acid (2e)

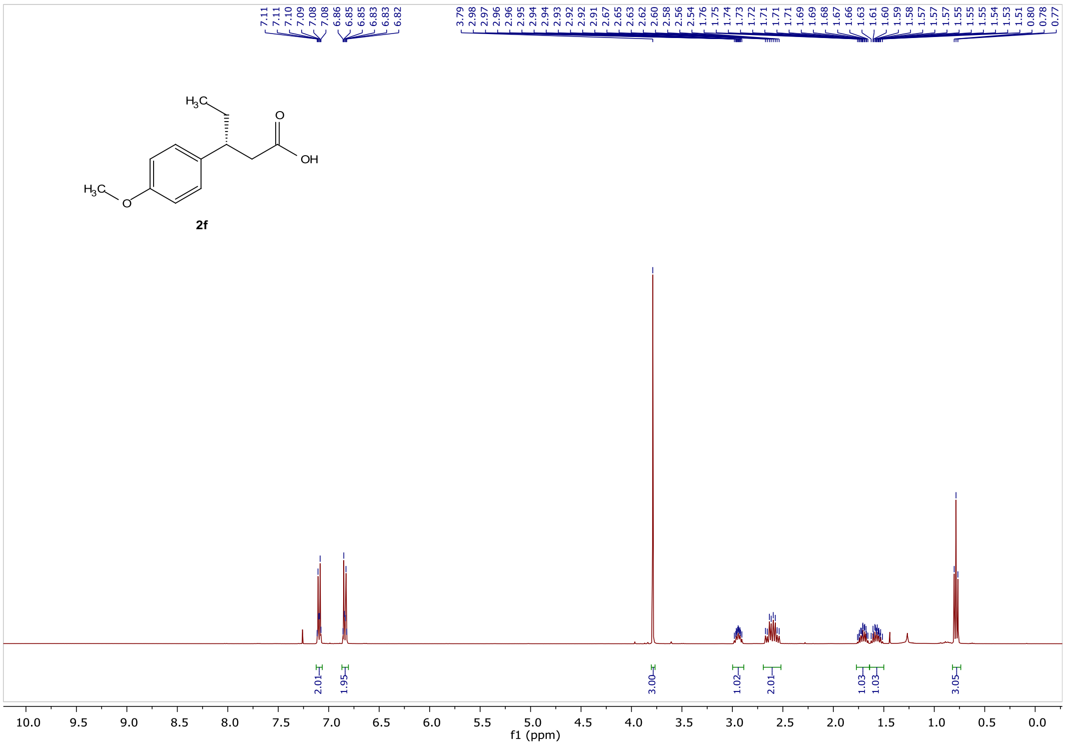

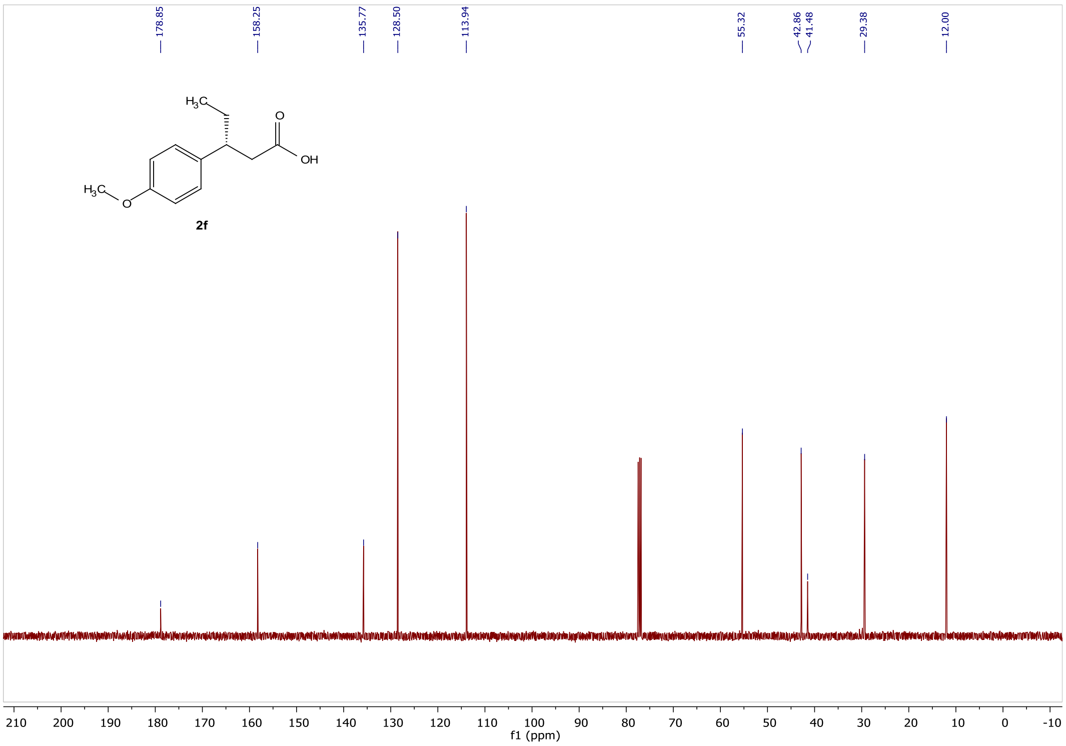

Supplementary Figure 14. NMR spectra of (*R*)-3-(4-methoxyphenyl)pentanoic acid (2f)

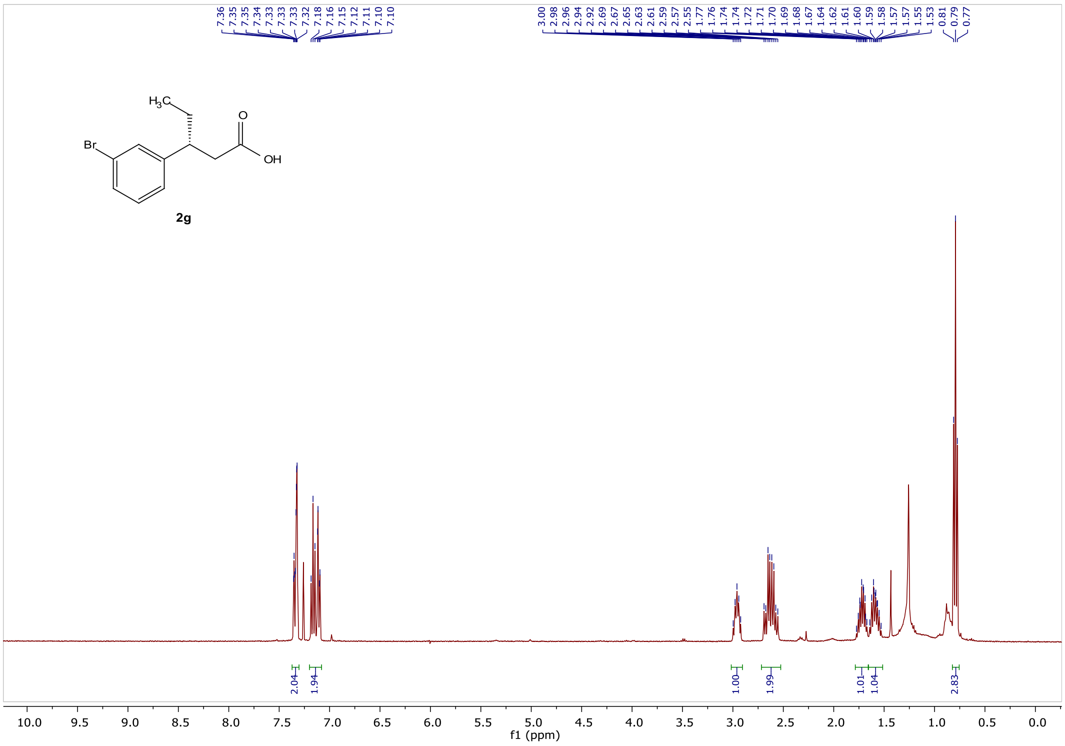

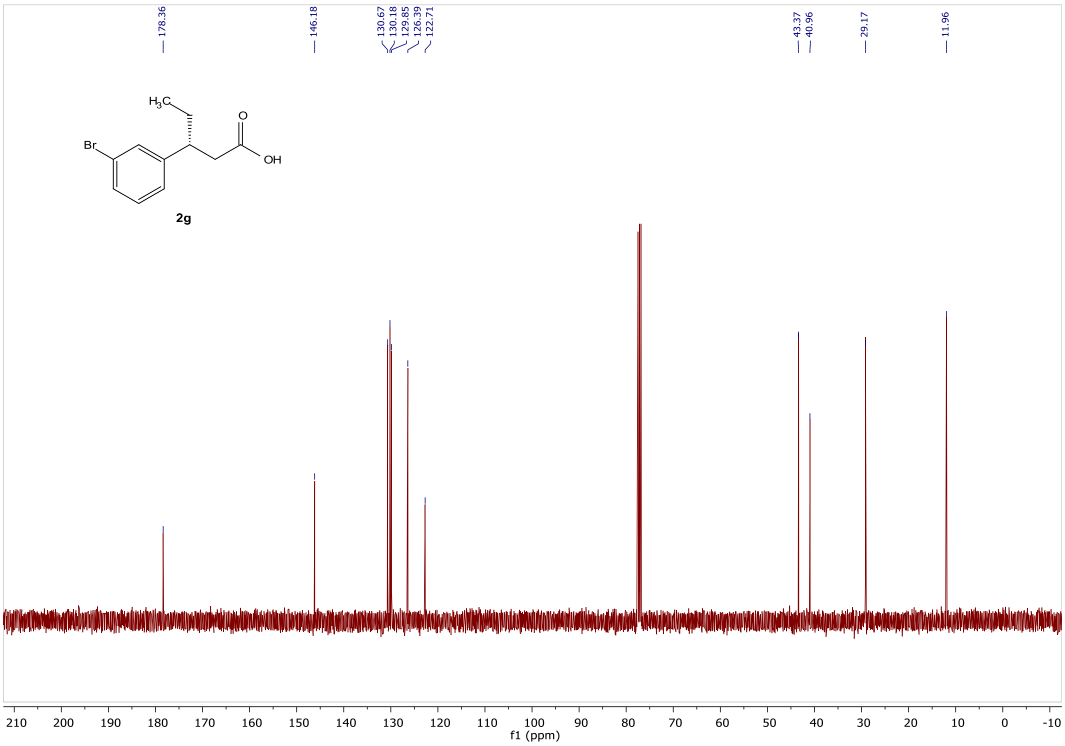

Supplementary Figure 15. NMR spectra of (*R*)-3-(3-bromophenyl)pentanoic acid (2g)

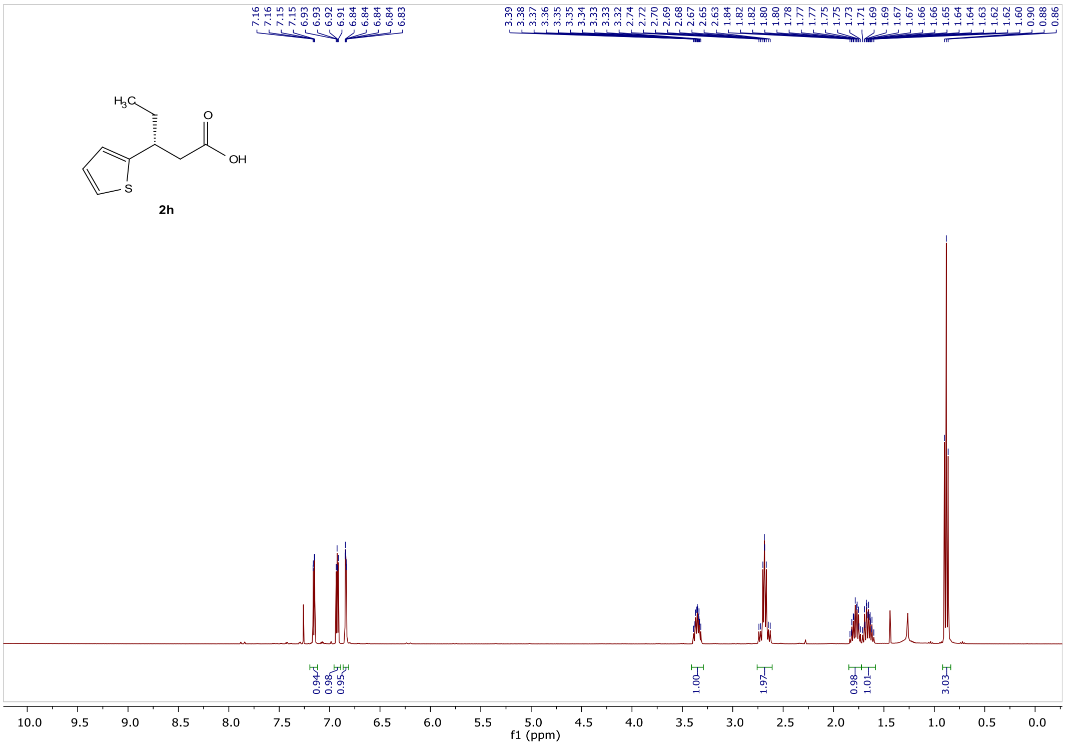

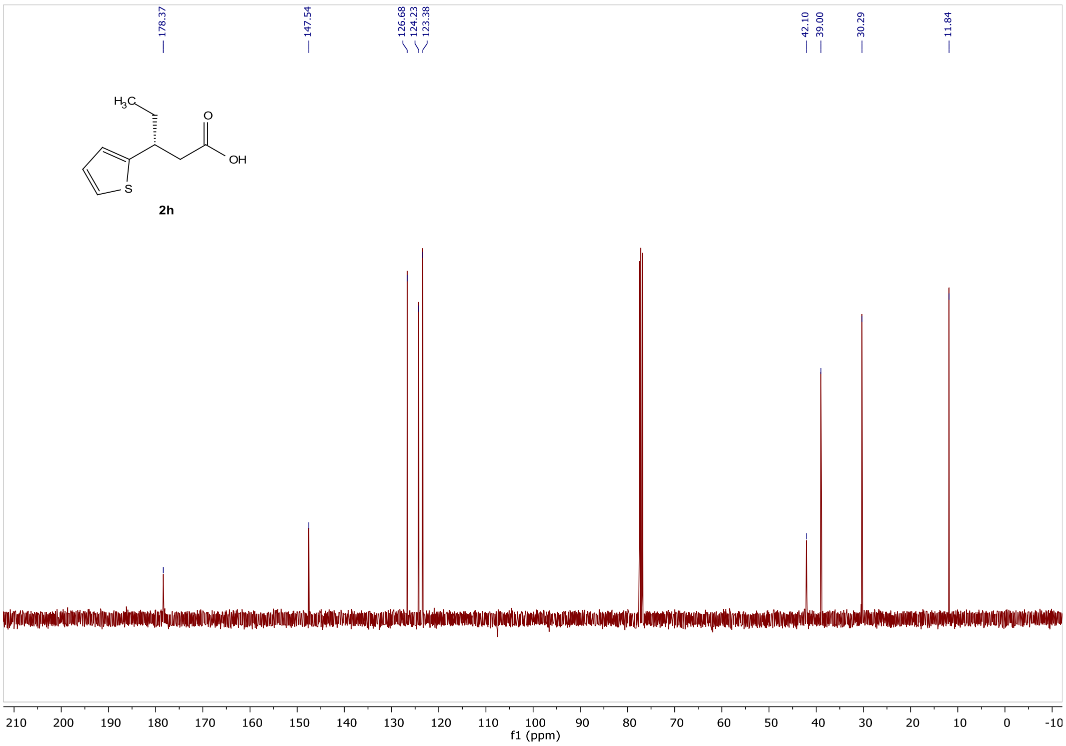

Supplementary Figure 16. NMR spectra of (*R*)-3-(thiophen-2-yl)pentanoic acid (2h)

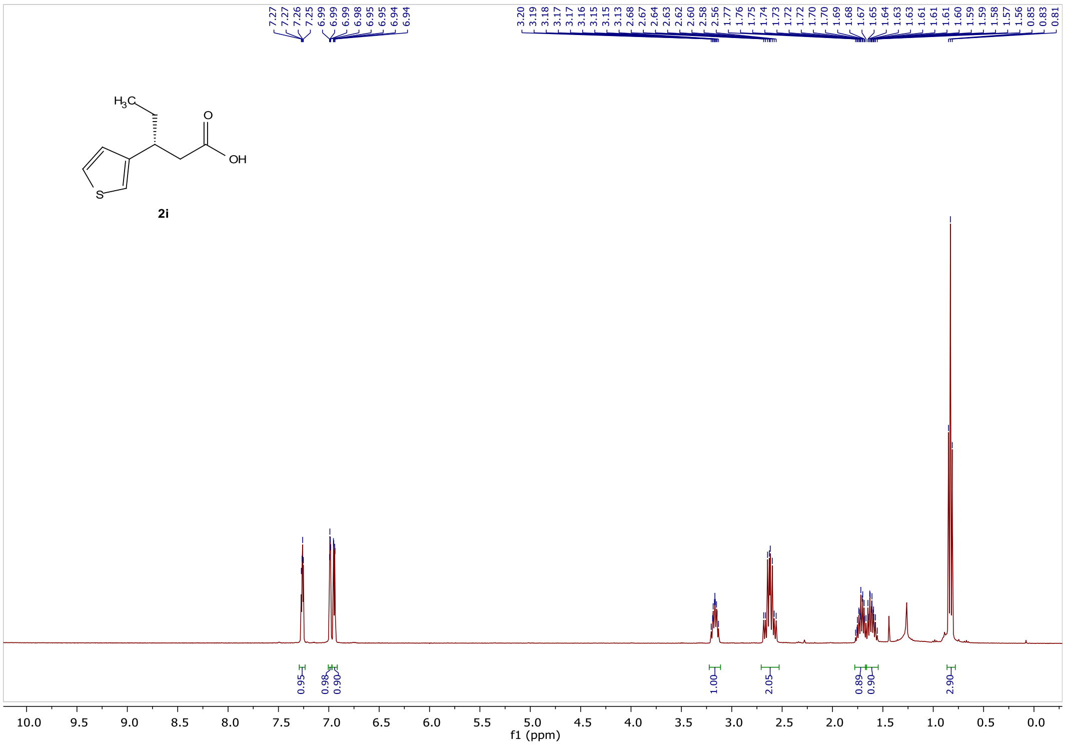

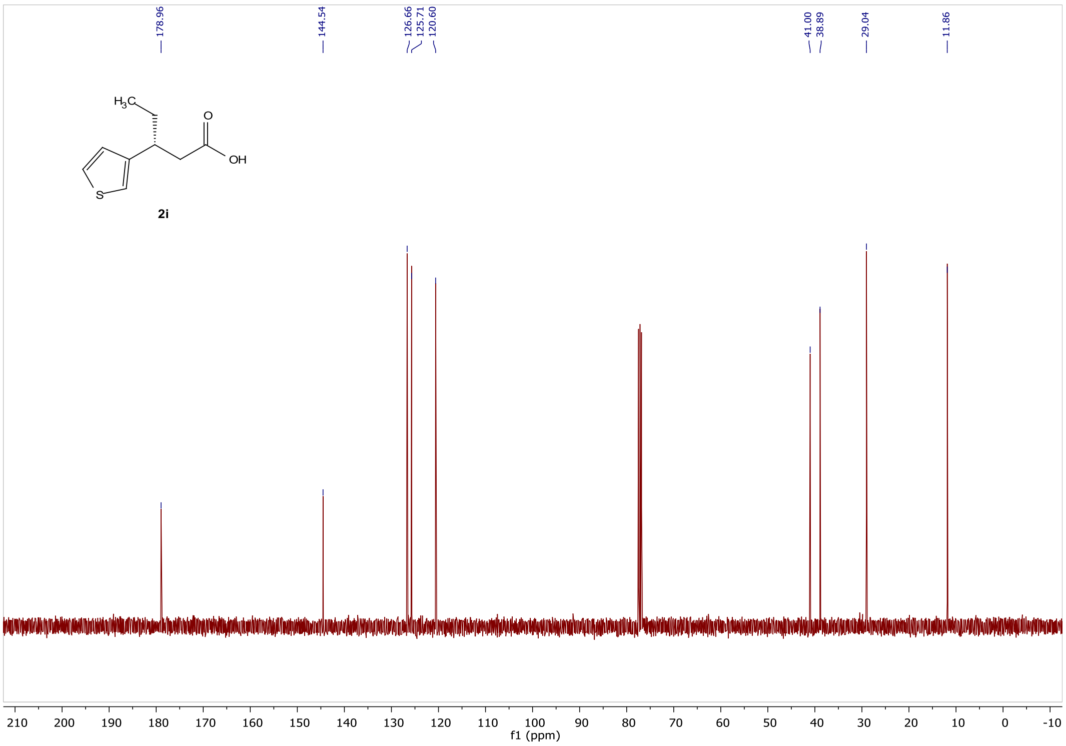

Supplementary Figure 17. NMR spectra of (*R*)-3-(thiophen-3-yl)pentanoic acid (2i)

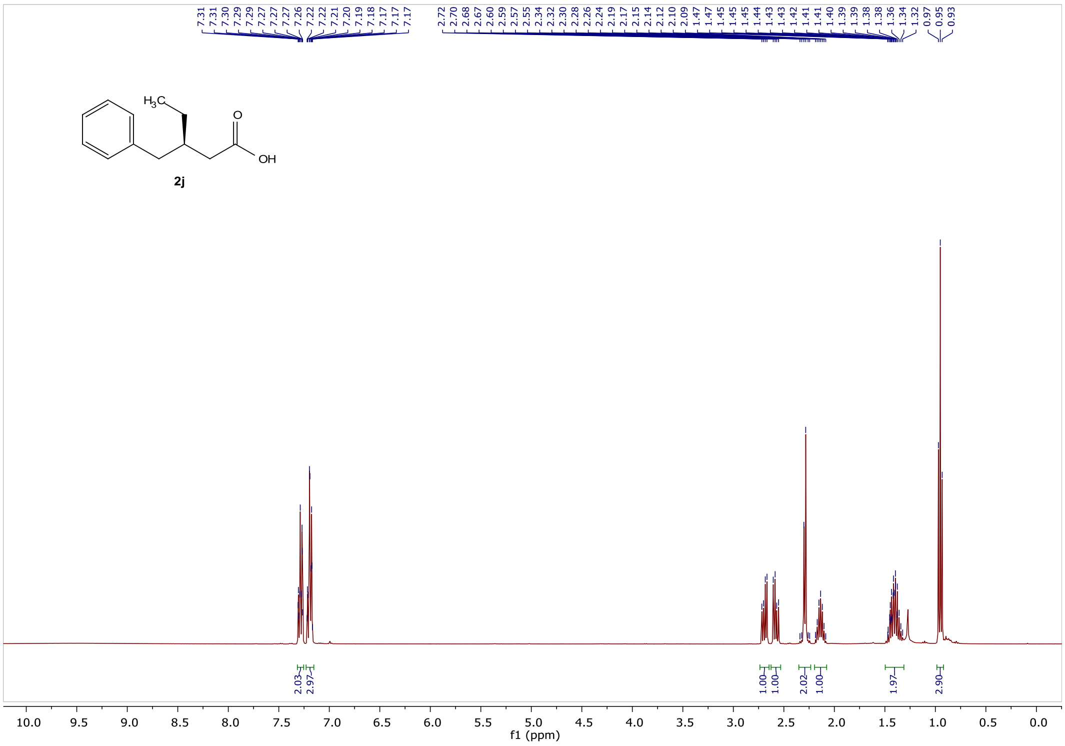

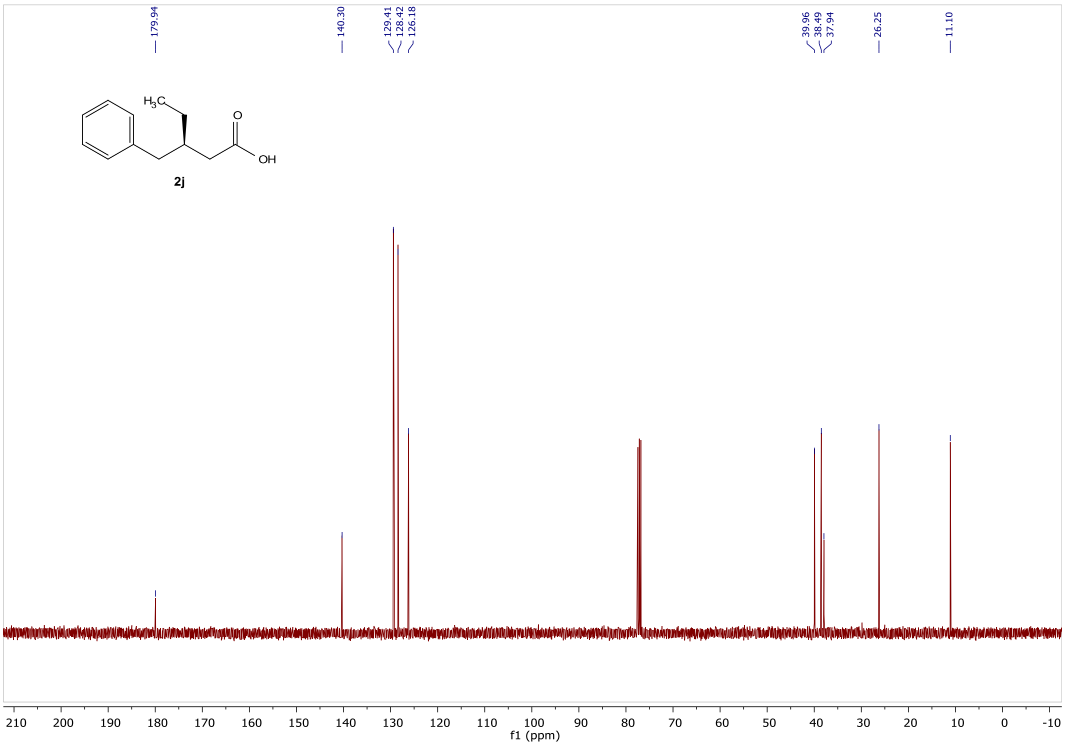

Supplementary Figure 18. NMR spectra of (*R*)-3-benzylpentanoic acid (2j)

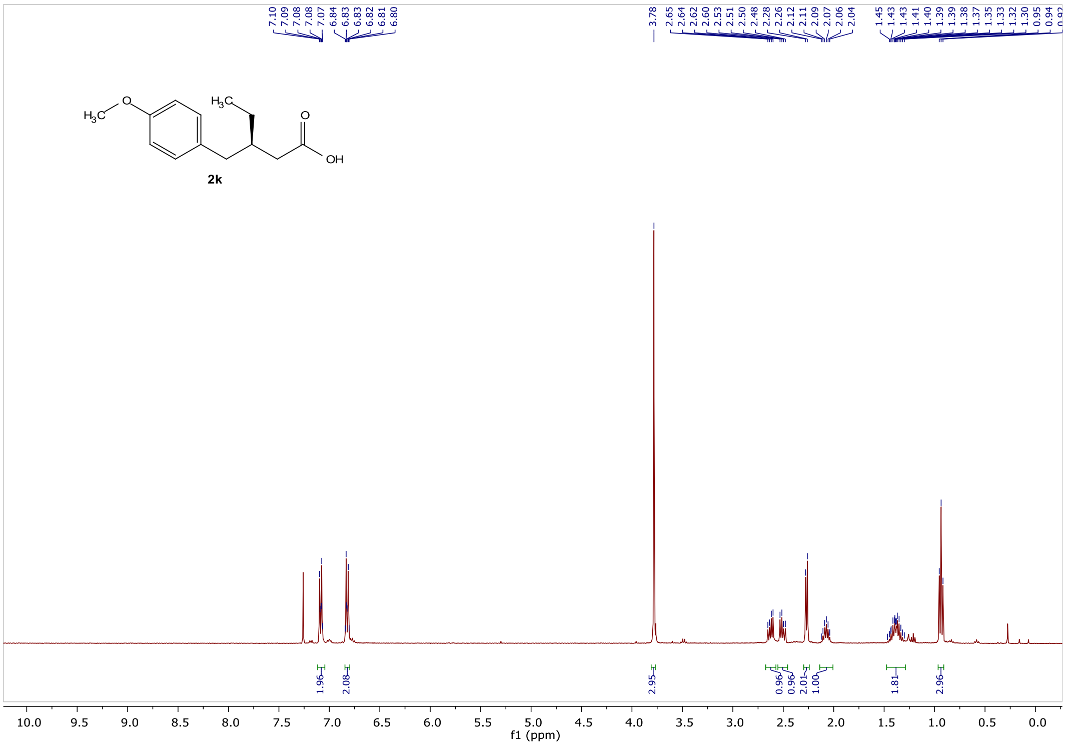

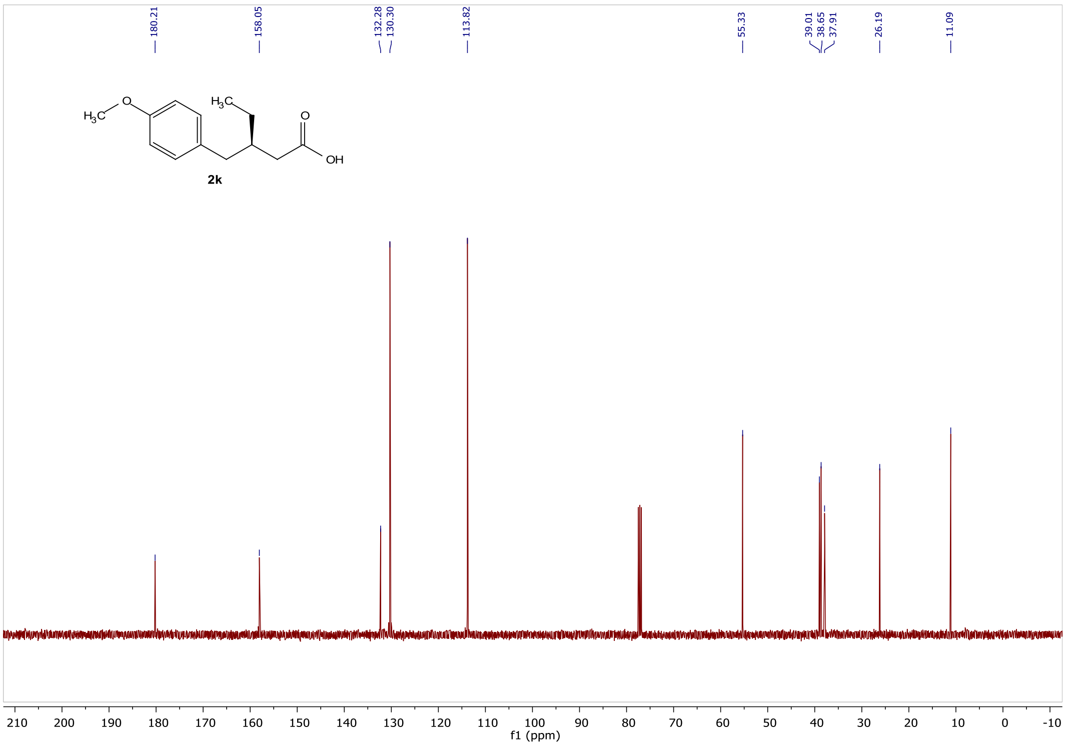

Supplementary Figure 19. NMR spectra of (*R*)-3-(4-methoxybenzyl)pentanoic acid (2k)

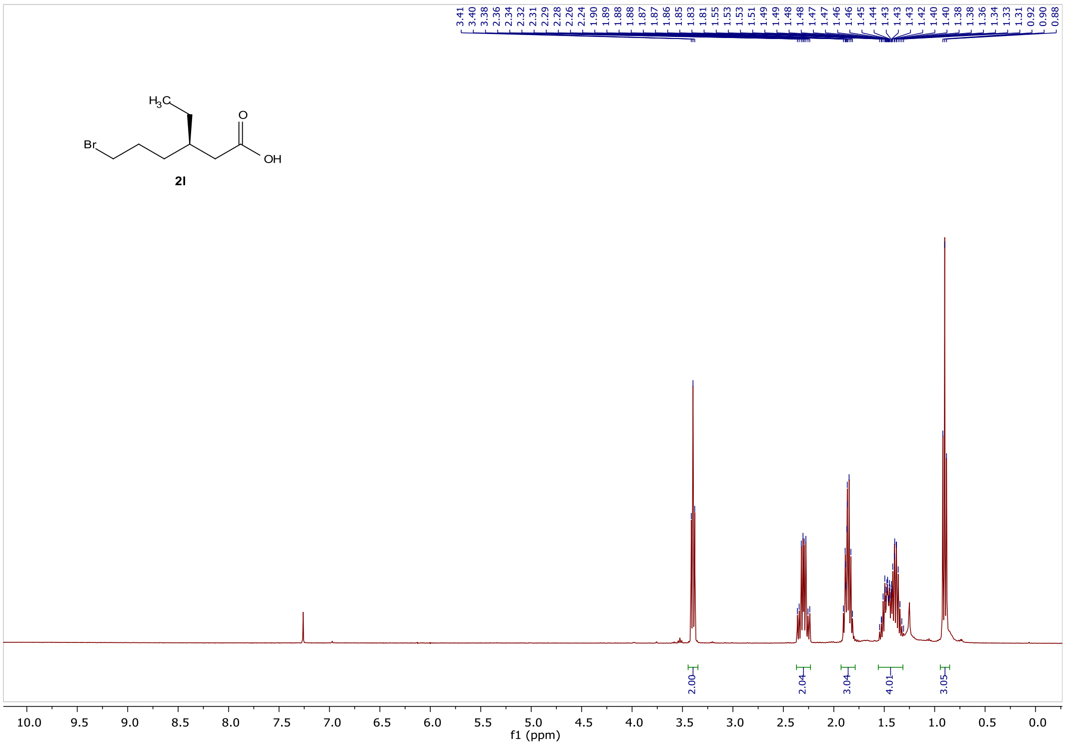

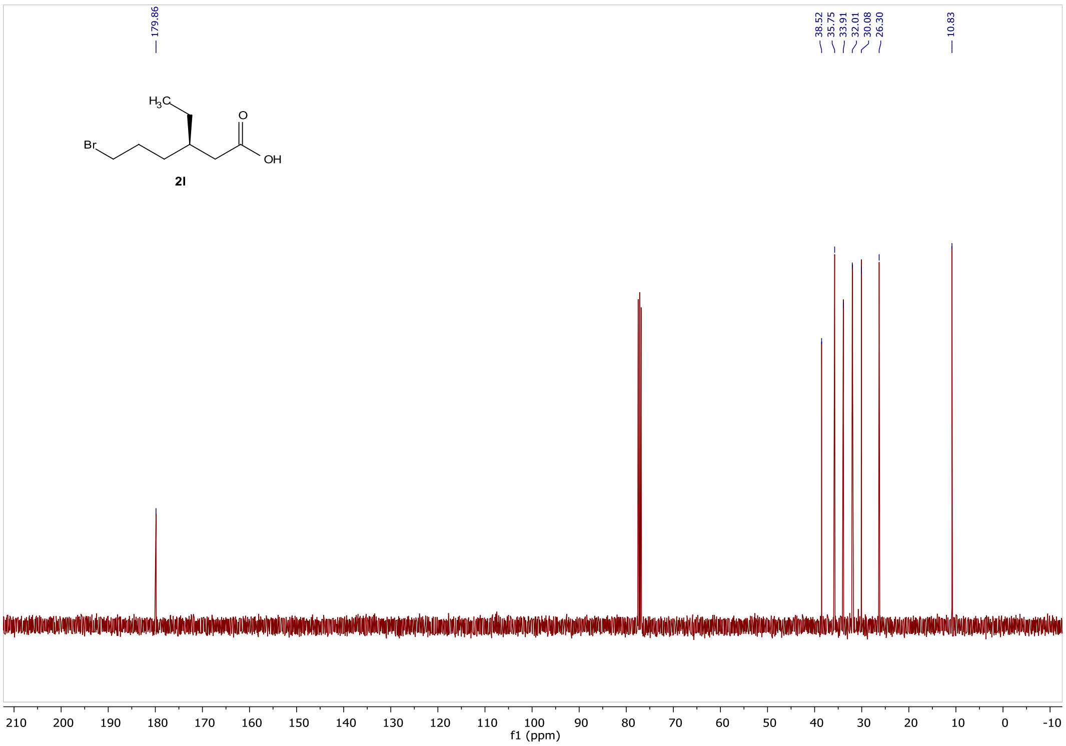

Supplementary Figure 20. NMR spectra of (*R*)-6-bromo-3-ethylhexanoic acid (2l)

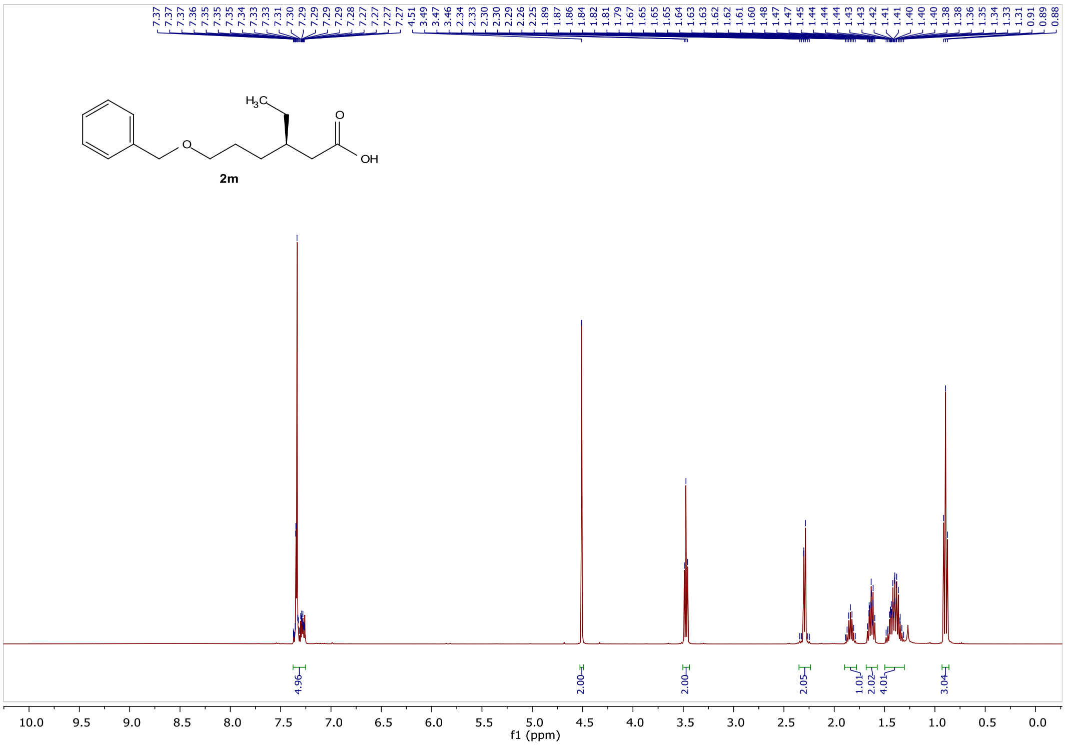

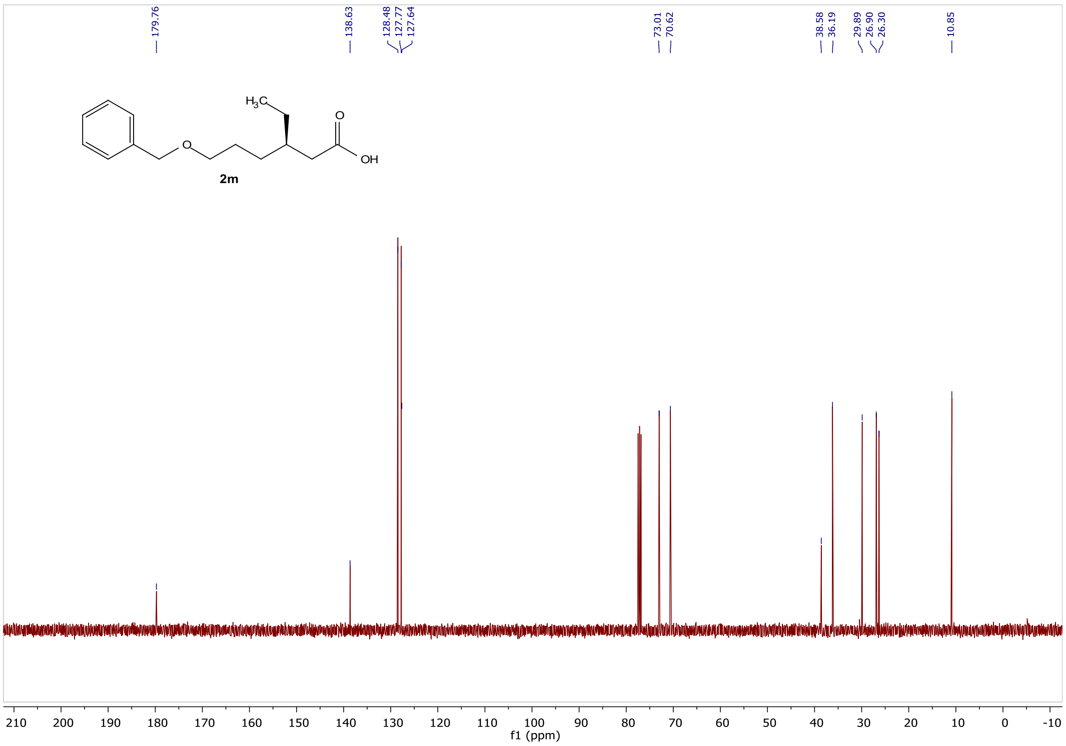

Supplementary Figure 21. NMR spectra of (*R*)-6-benzyloxy-3-ethylhexanoic acid (2m)

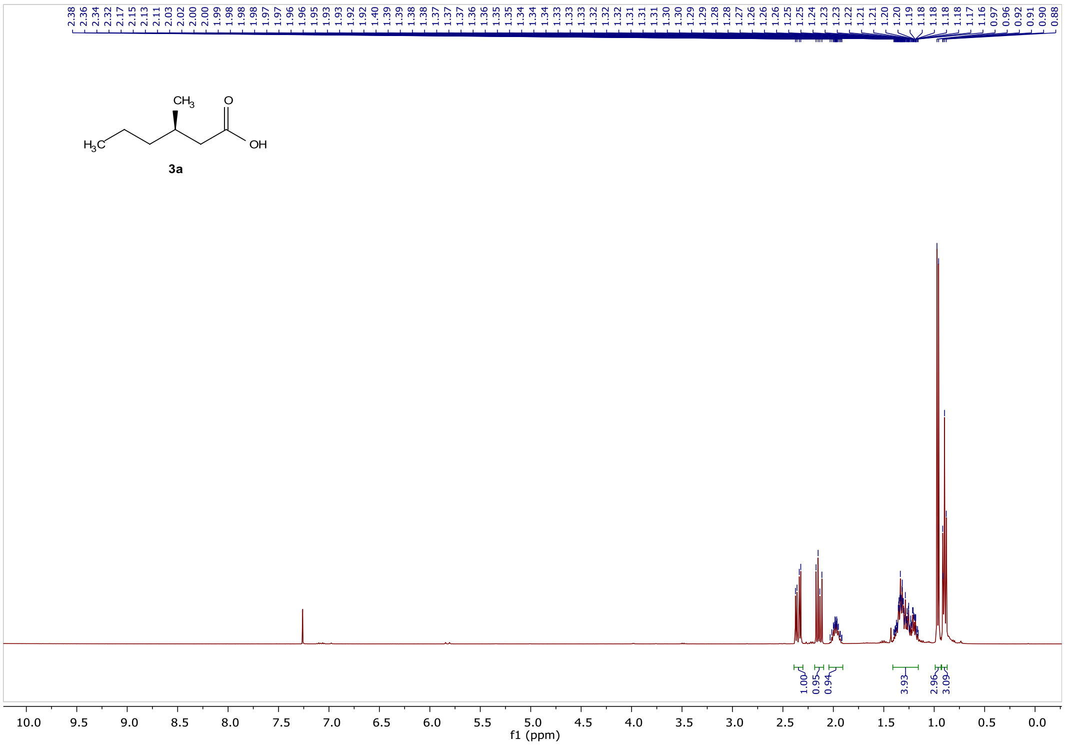

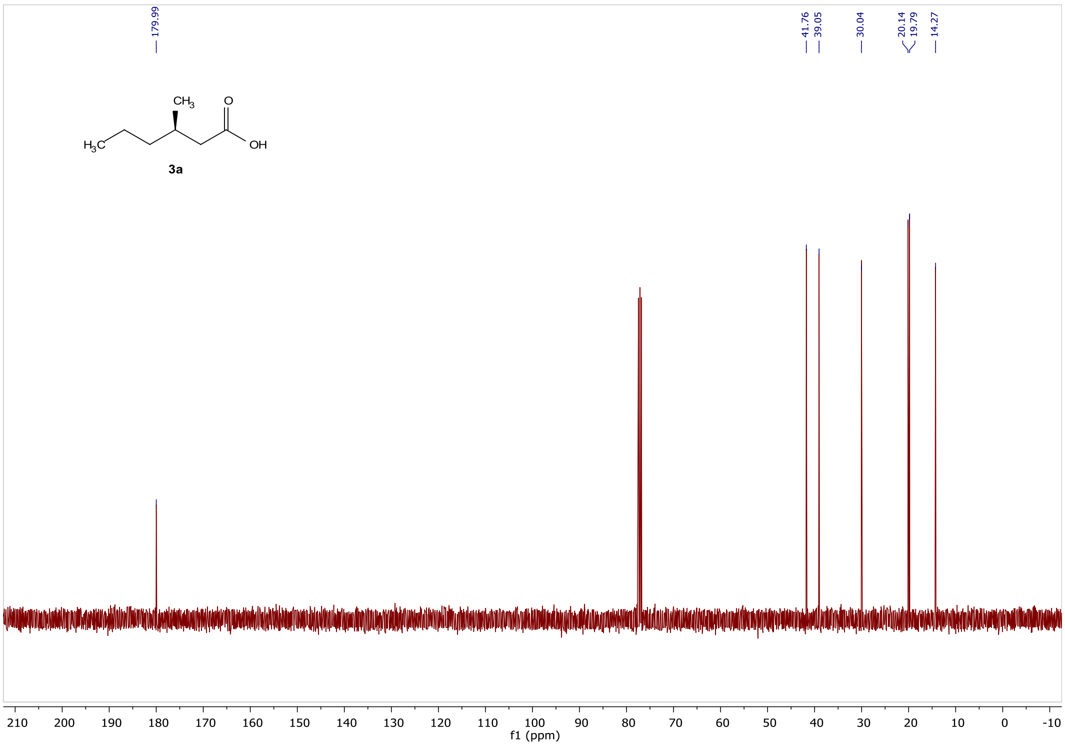

Supplementary Figure 22. NMR spectra of (*R*)-3-methylhexanoic acid (3a)

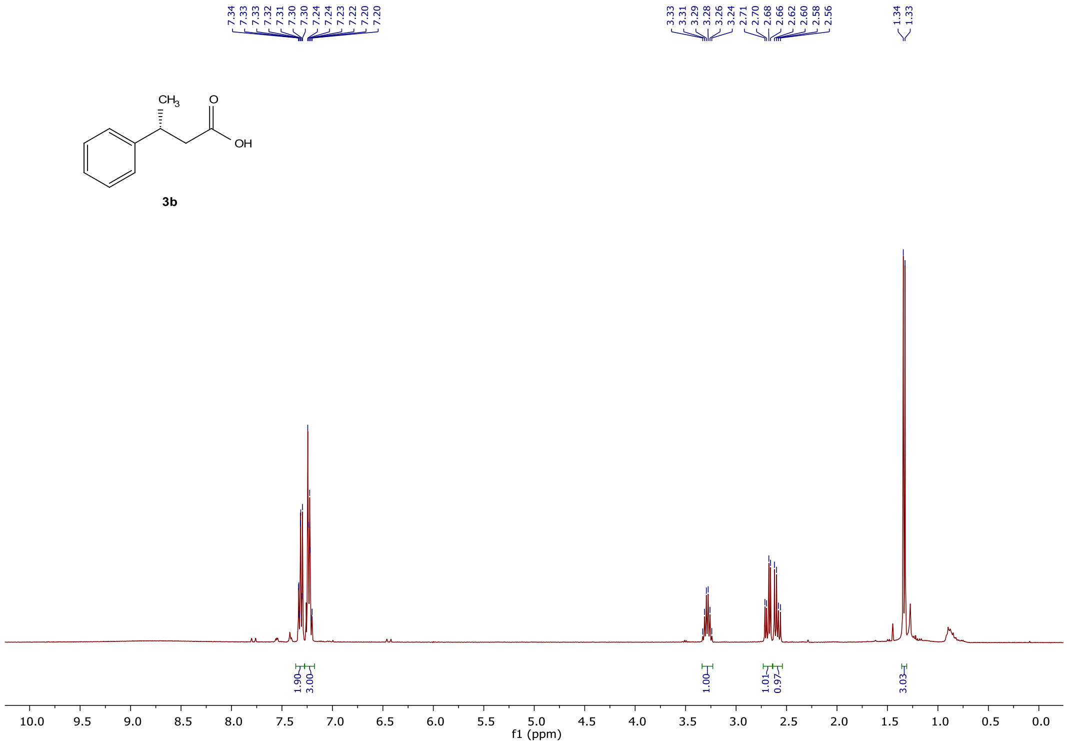

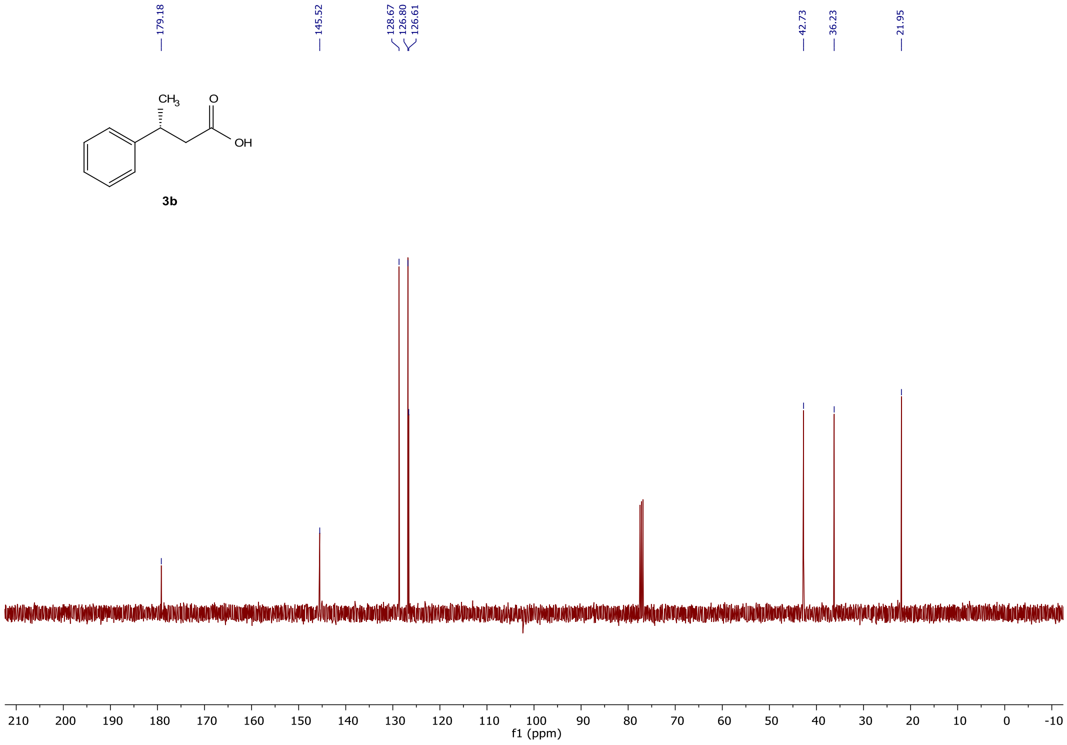

Supplementary Figure 23. NMR spectra of (*R*)-3-phenylbutanoic acid (3b)

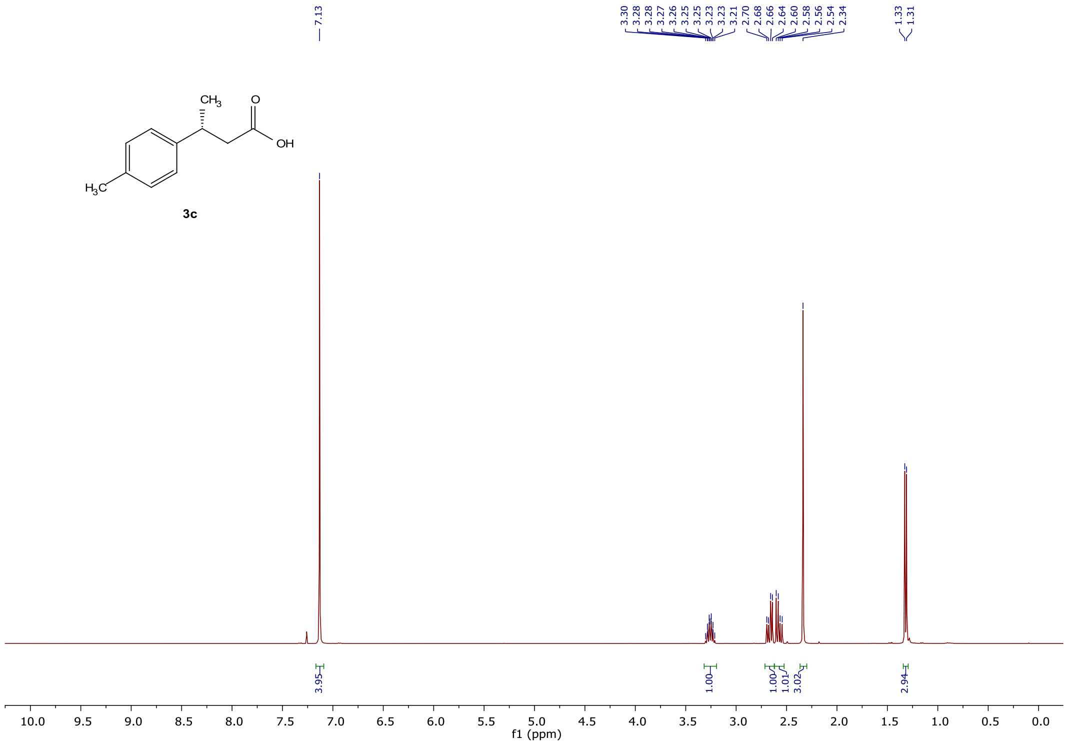

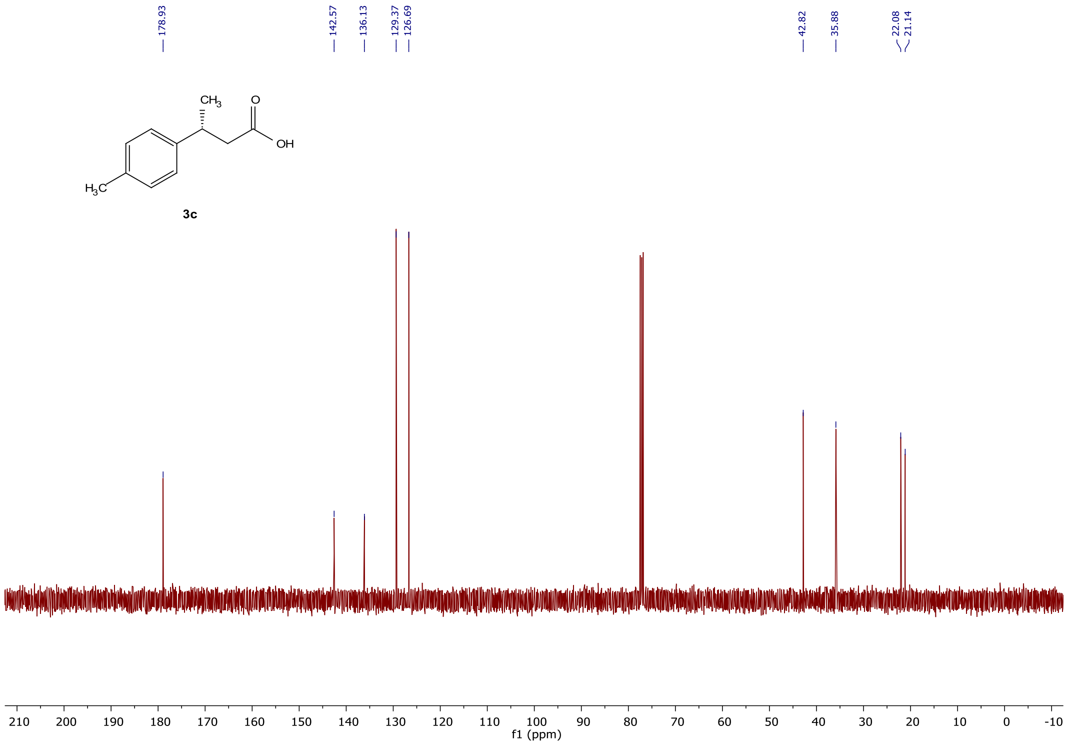

Supplementary Figure 24. NMR spectra of (*R*)-3-(4-methylphenyl)butanoic acid (3c)

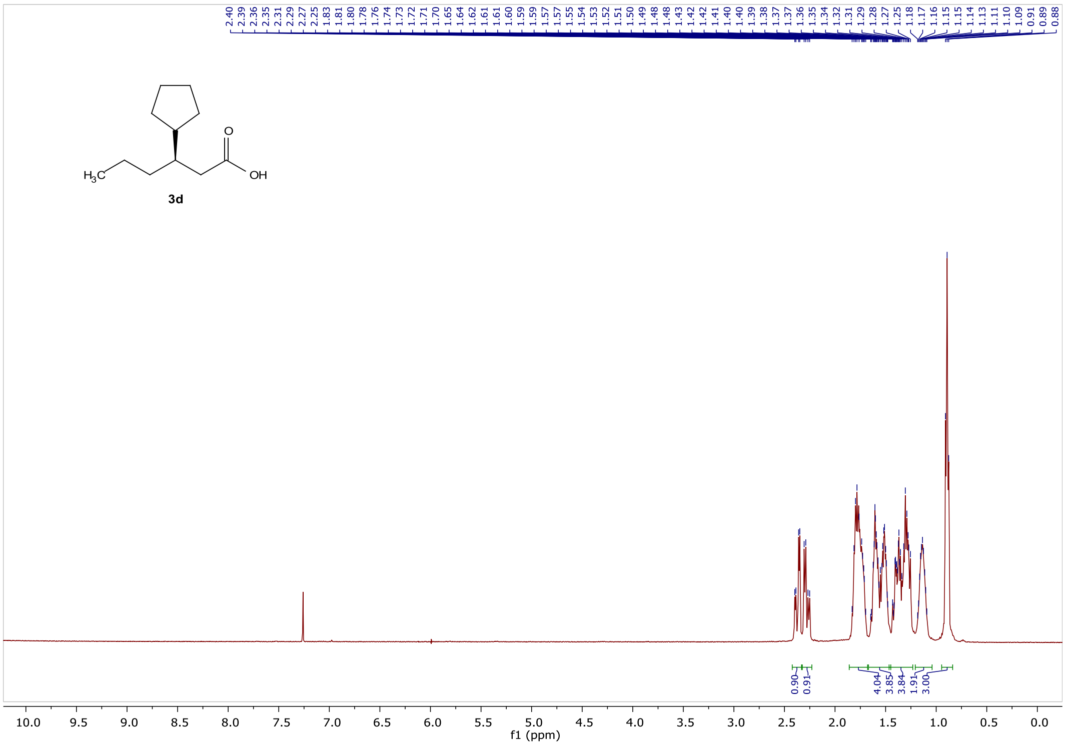

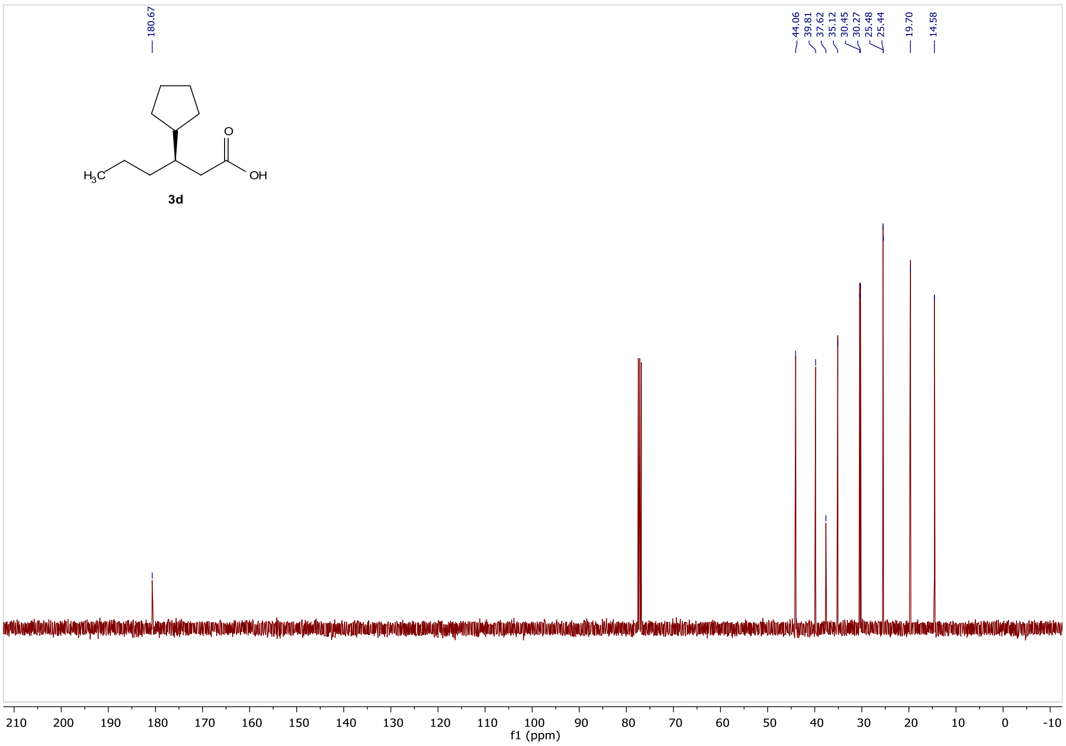

Supplementary Figure 25. NMR spectra of (*R*)-3-cyclopentylhexanoic acid (3d)

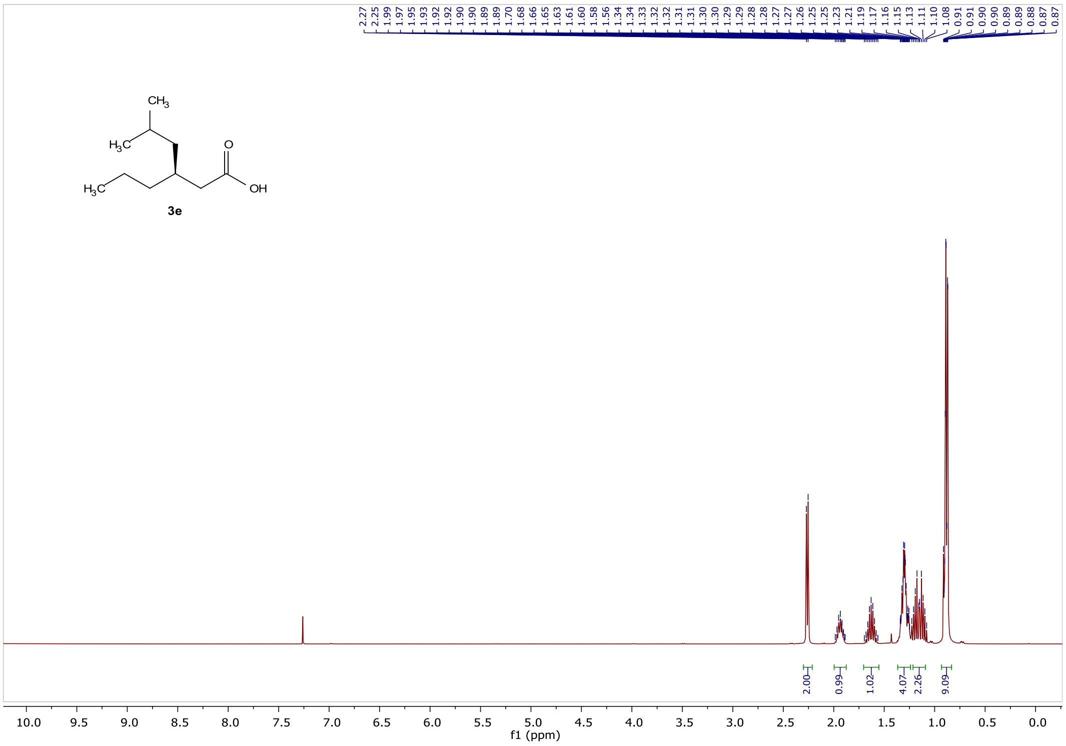

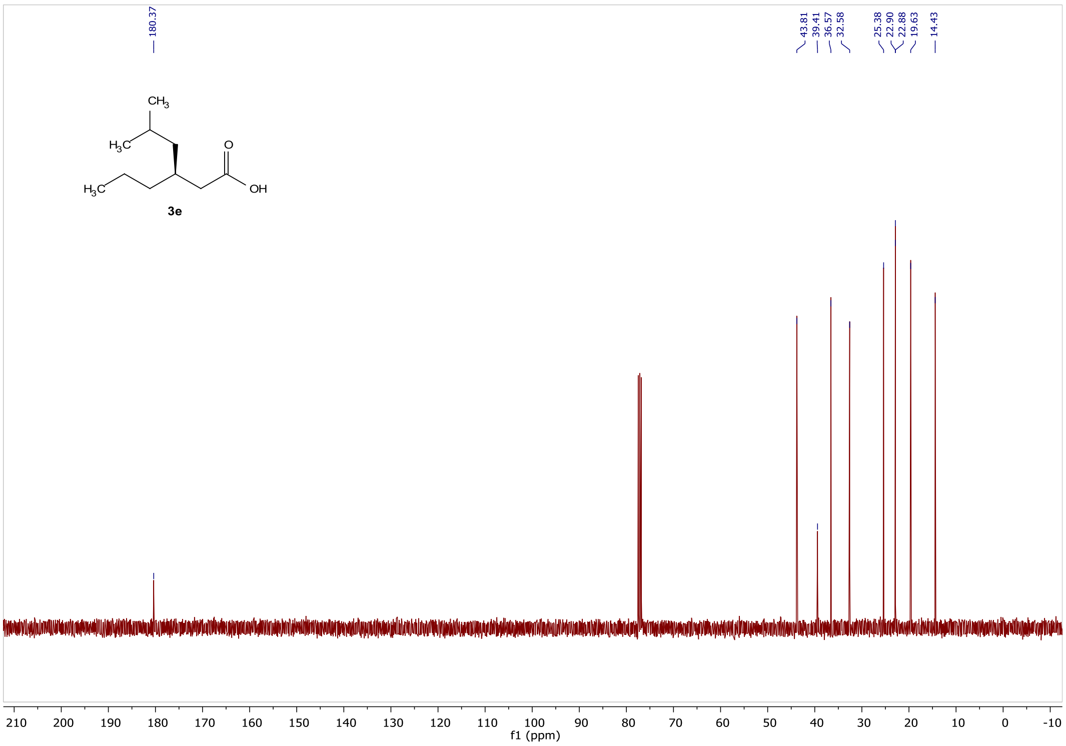

Supplementary Figure 26. NMR spectra of (*S*)-5-methyl-3-propylhexanoic acid (3e)

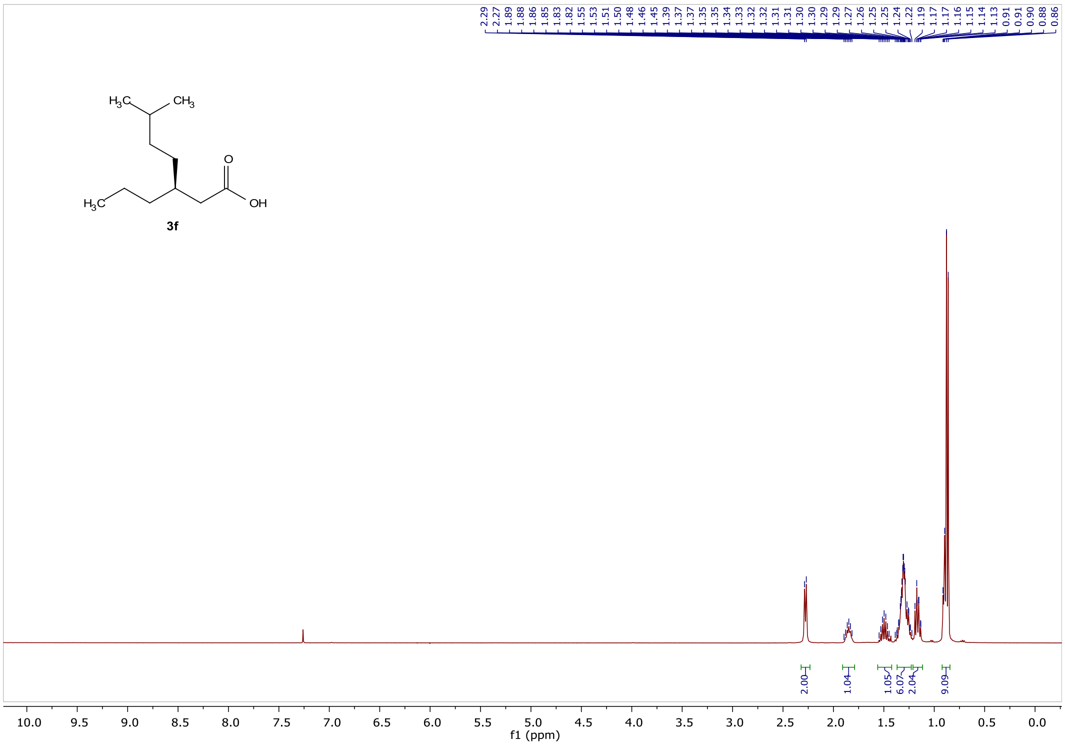

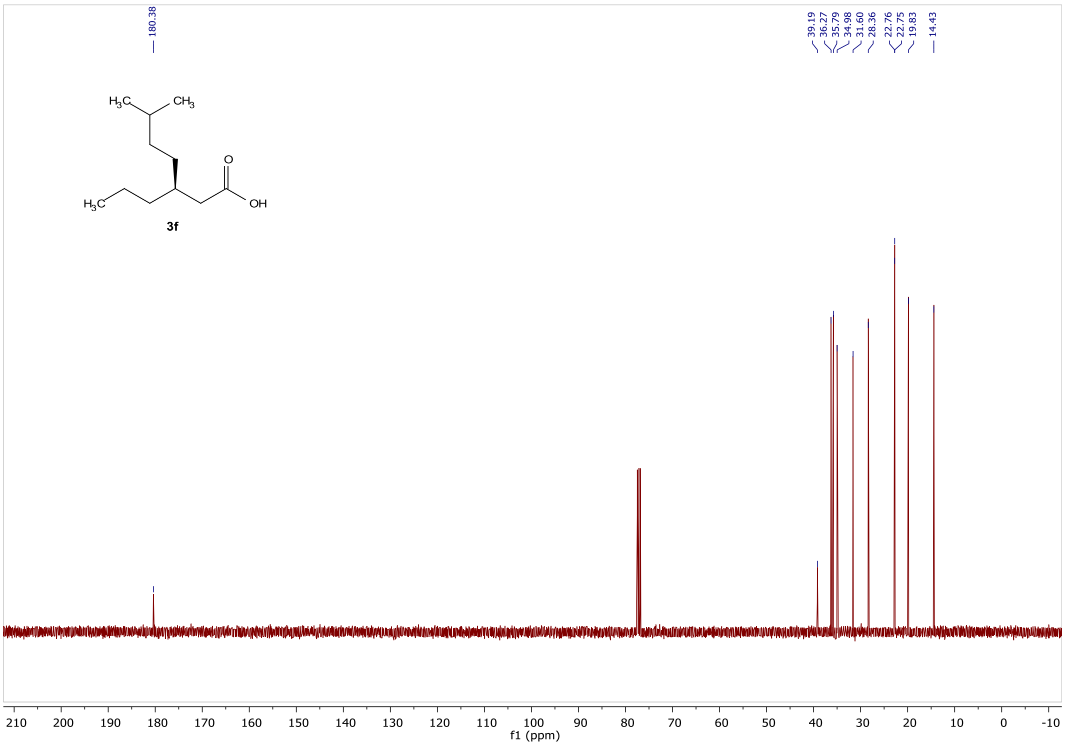

Supplementary Figure 27. NMR spectra of (*S*)-6-methyl-3-propylheptanoic acid (3f)

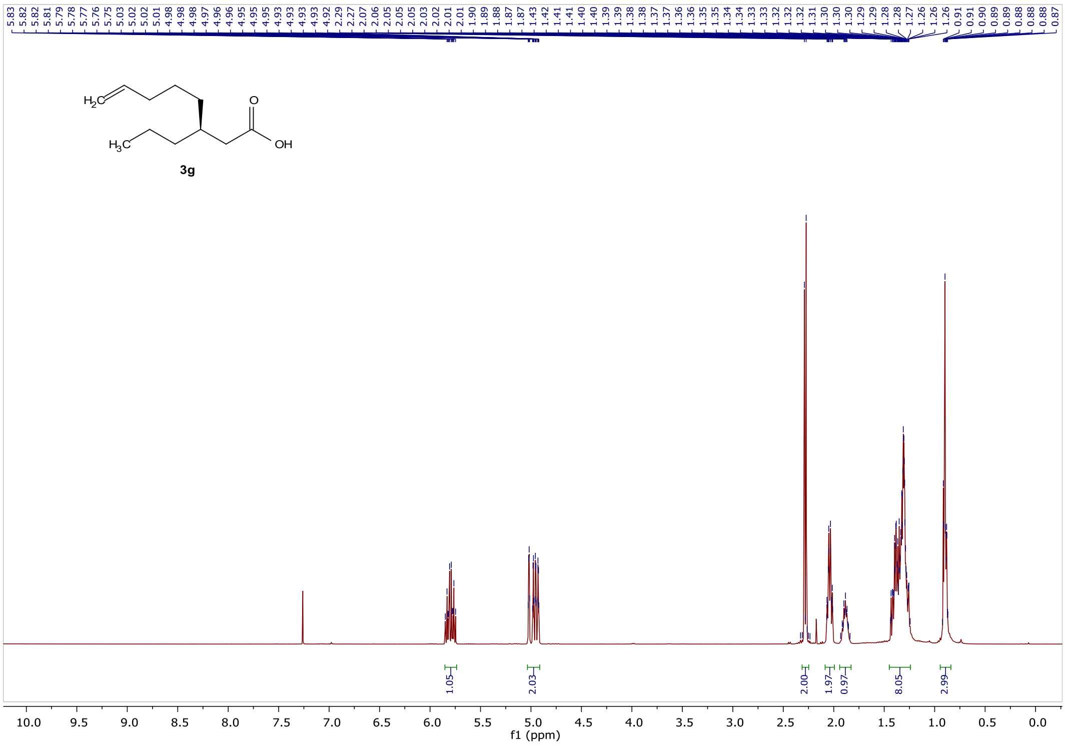

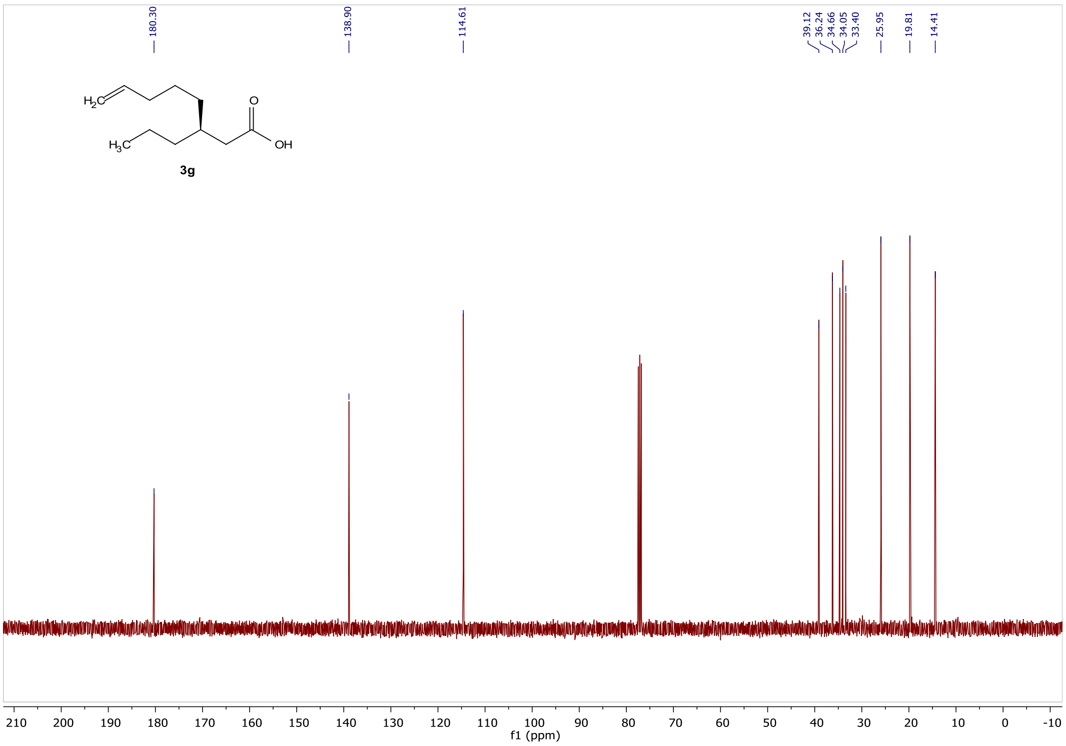

Supplementary Figure 28. NMR spectra of (*S*)-3-propyl-oct-7-enoic acid (3g)

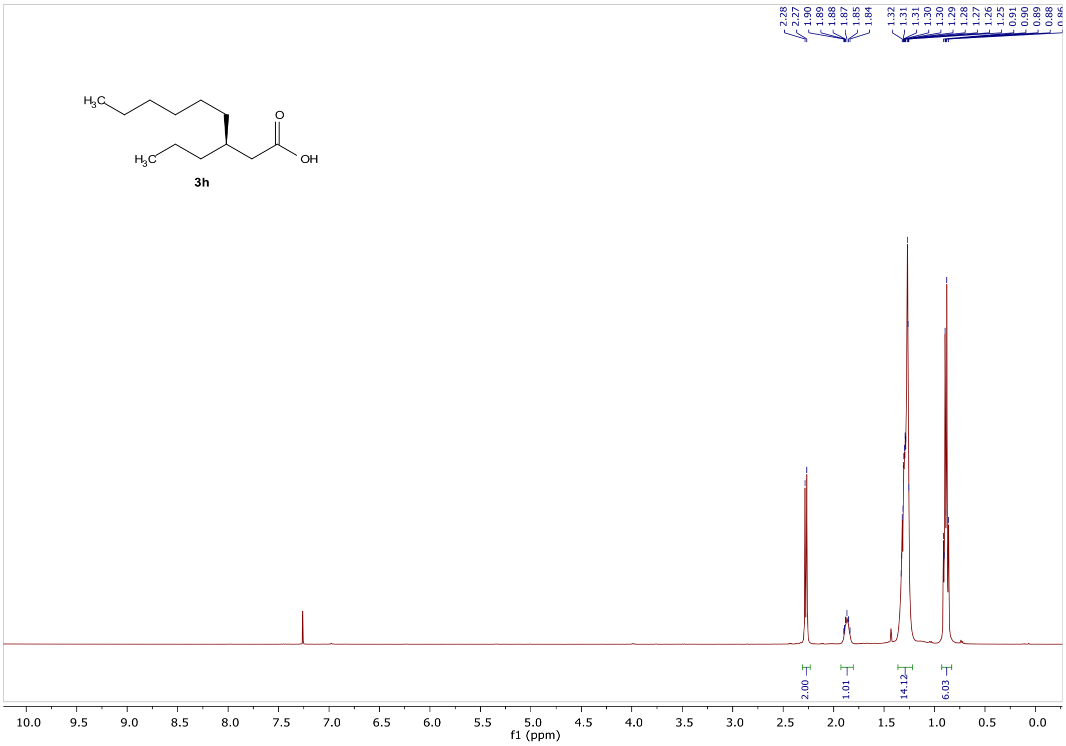

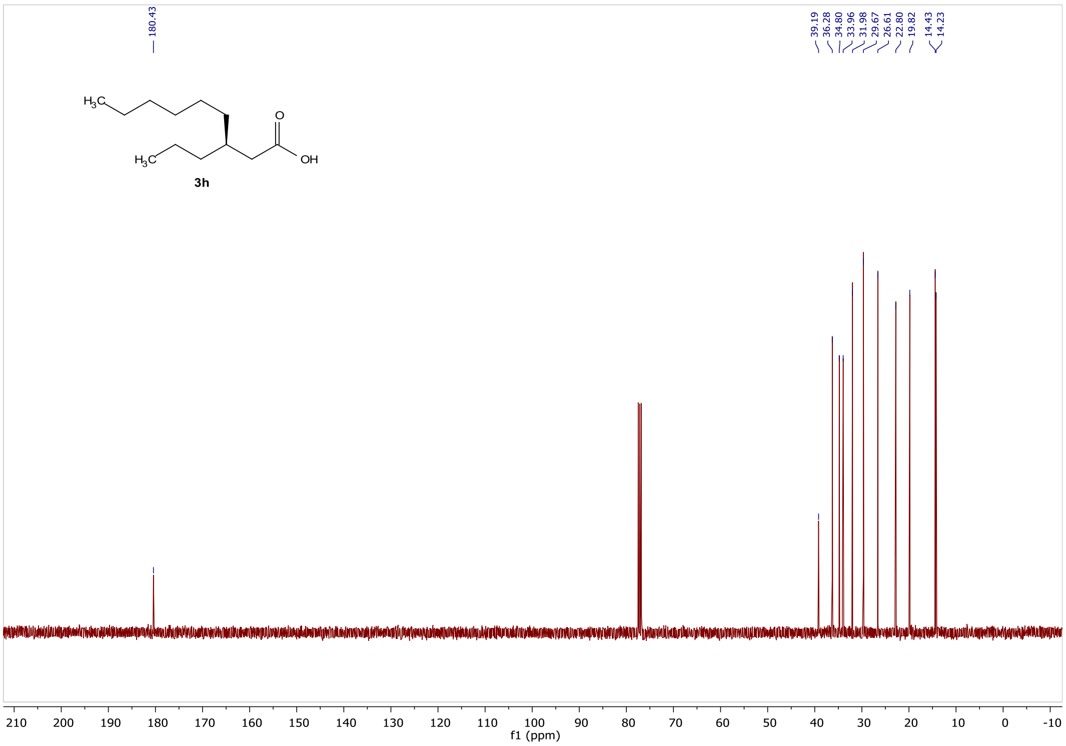

Supplementary Figure 29. NMR spectra of (*S*)-3-propylnonanoic acid (3h)

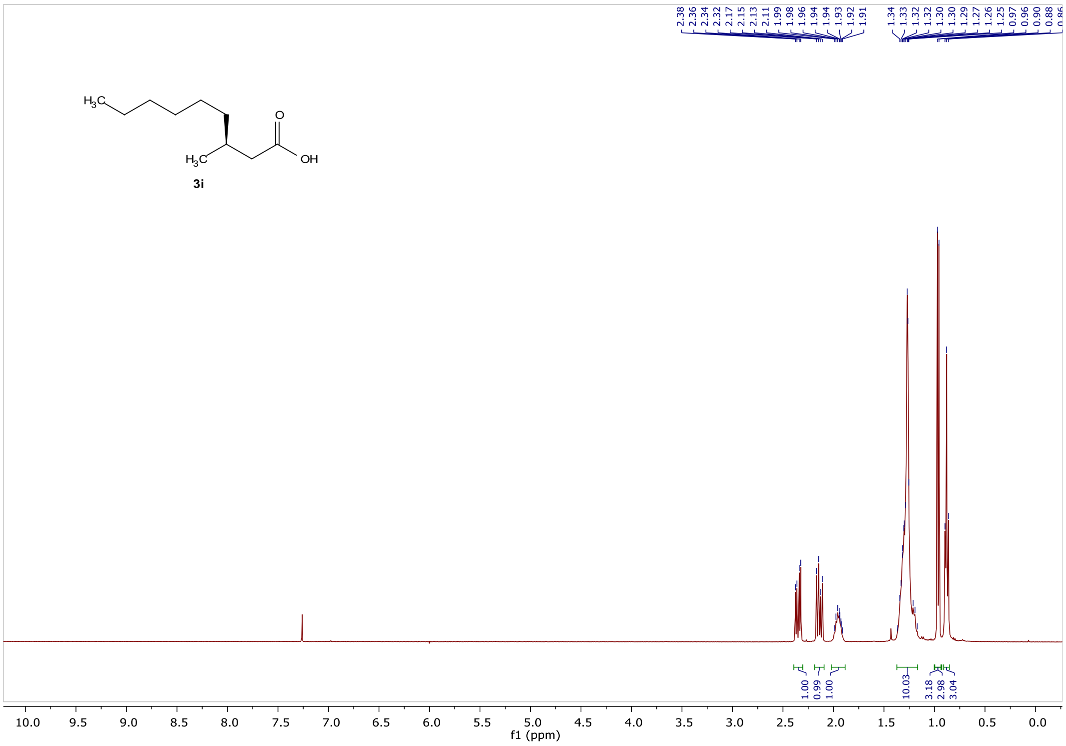

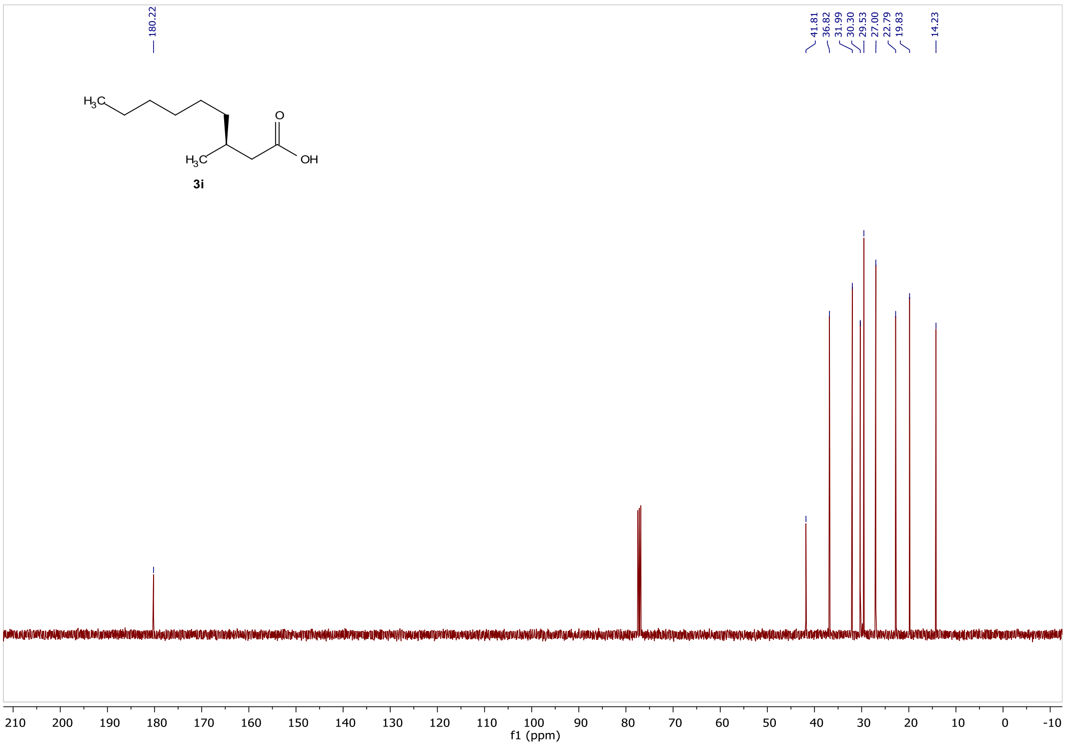

Supplementary Figure 30. NMR spectra of (*S*)-3-methylnonanoic acid (3i)

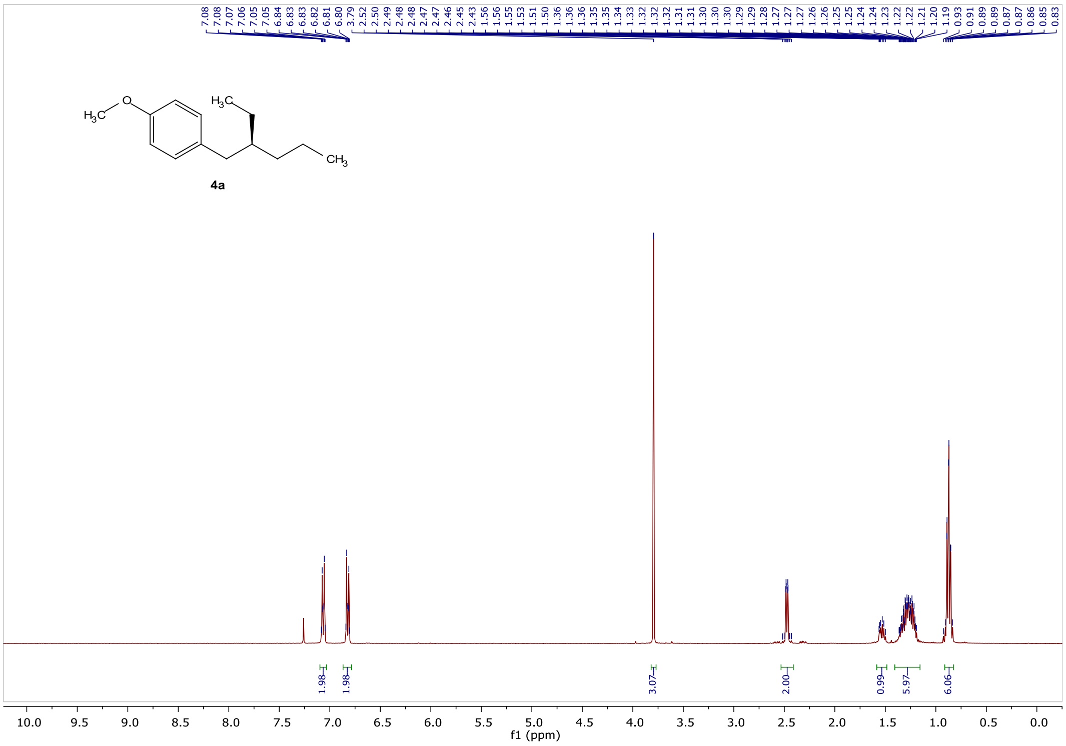

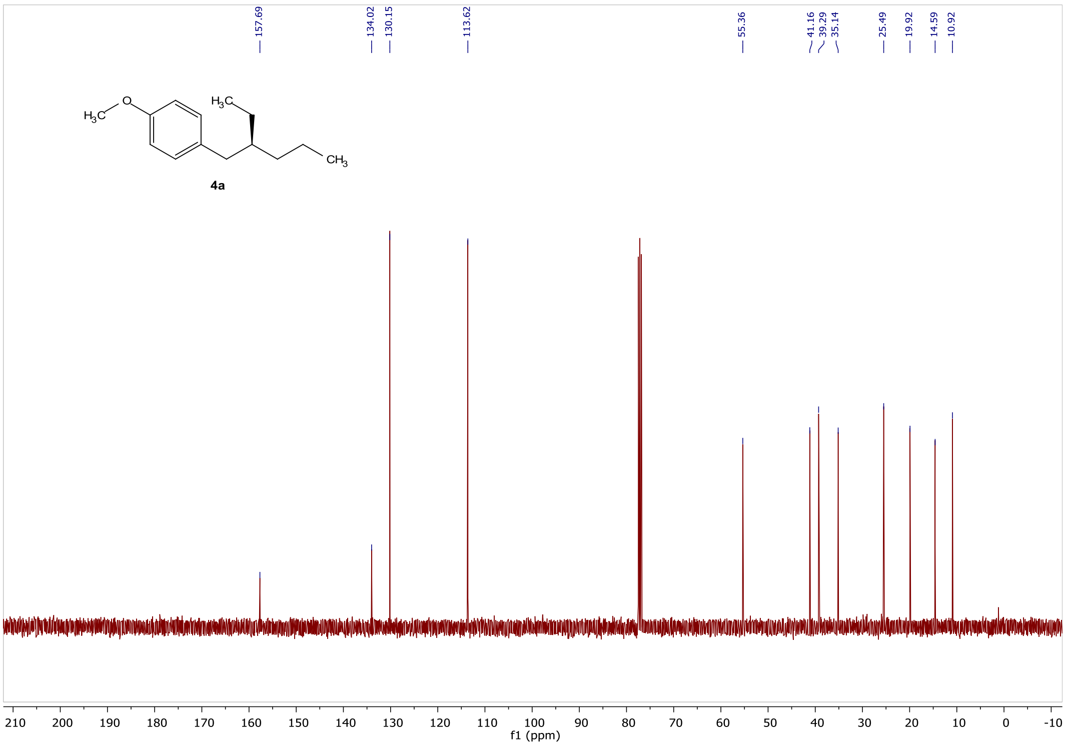

Supplementary Figure 31. NMR spectra of (*S*)-1-(2-ethylpentyl)-4-methoxy-benzene (4a)

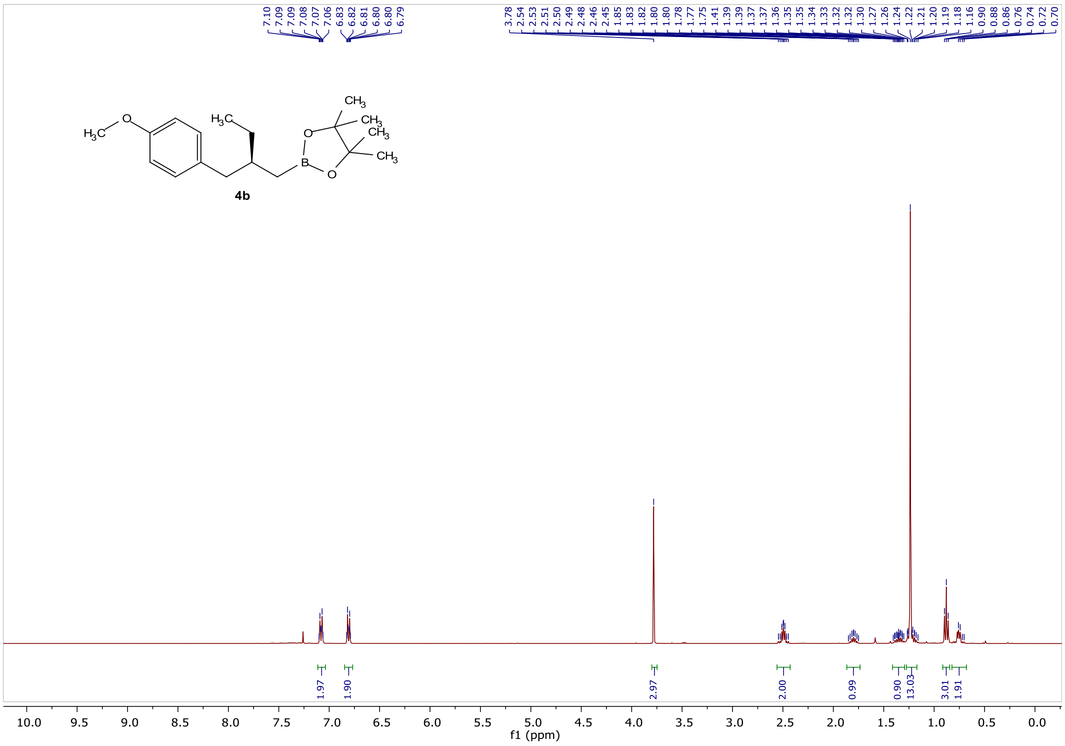

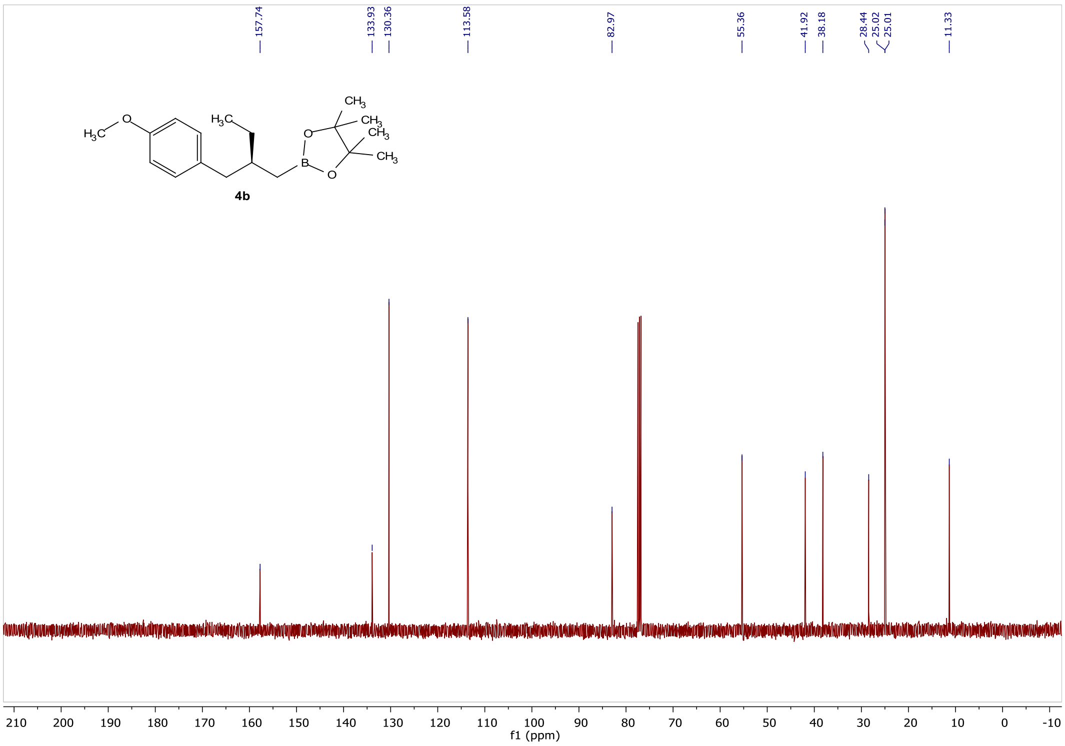

Supplementary Figure 32. NMR spectra of (*R*)-4,4,5,5-tetramethyl-2-(2-(4-methoxybenzyl)butyl)-1,3,2-dioxaborolane (4b)

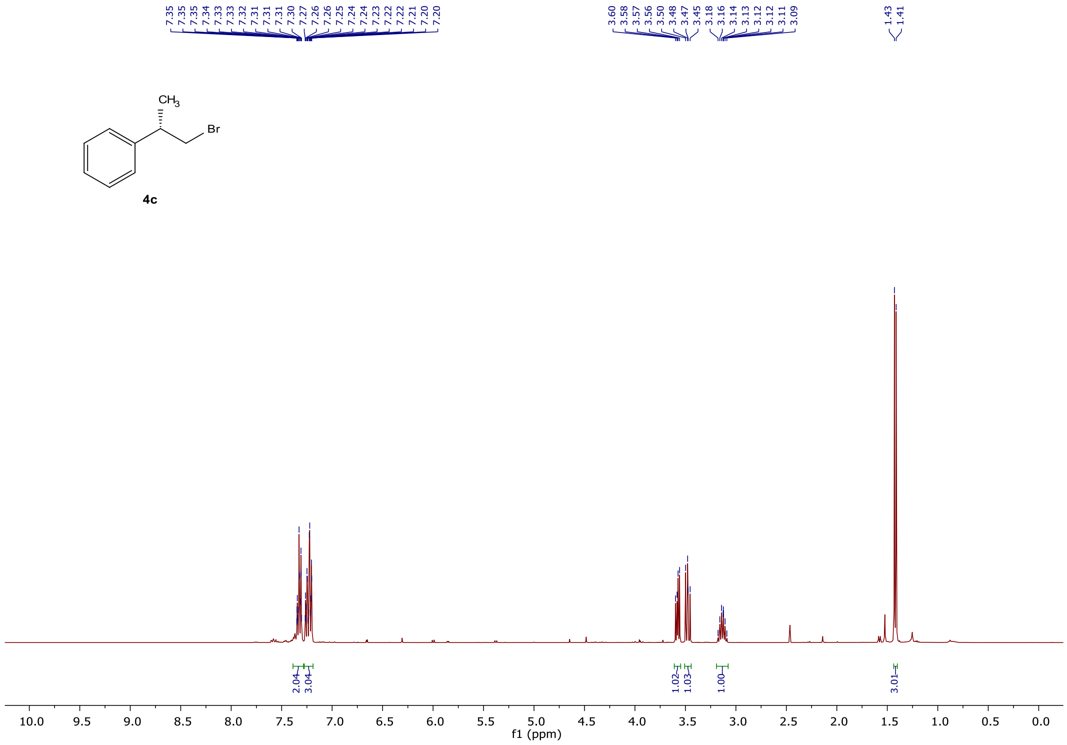

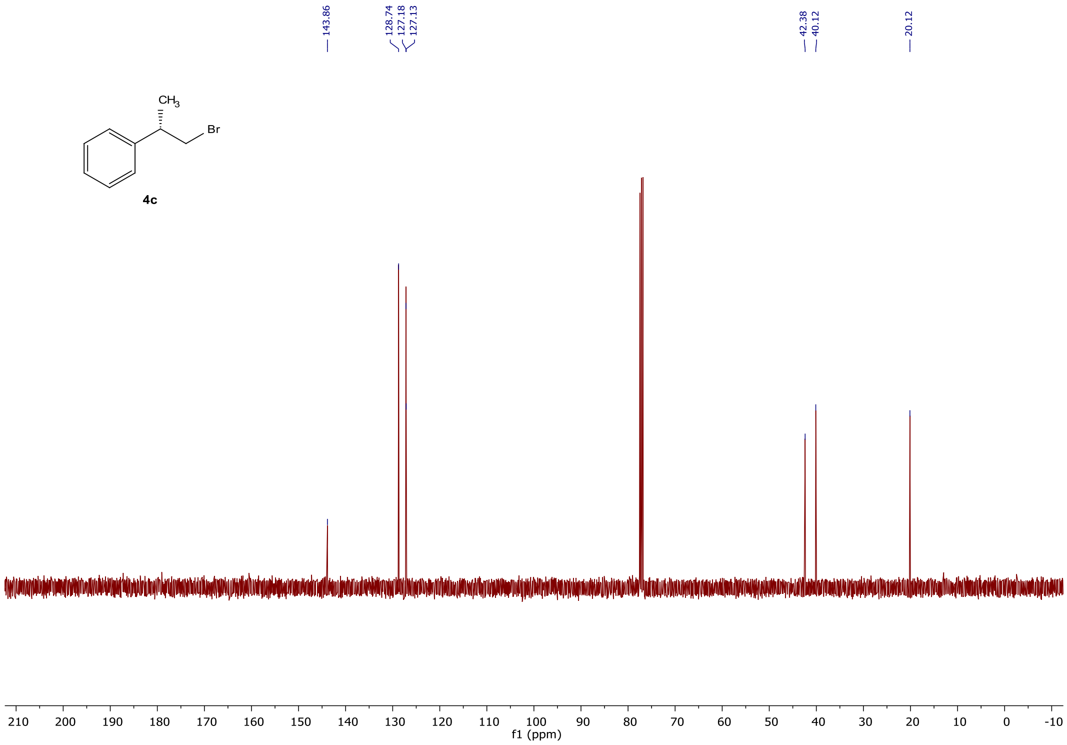

Supplementary Figure 33. NMR spectra of (*R*)-(2-bromo-1-methylethyl)benzene (4c)

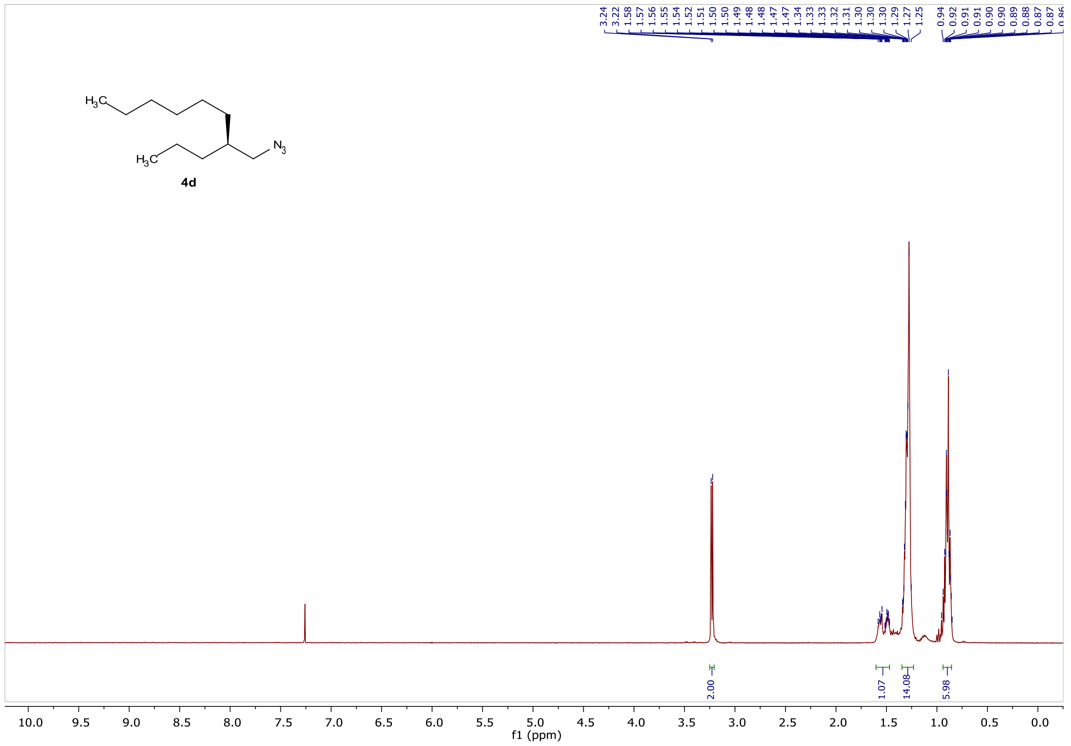

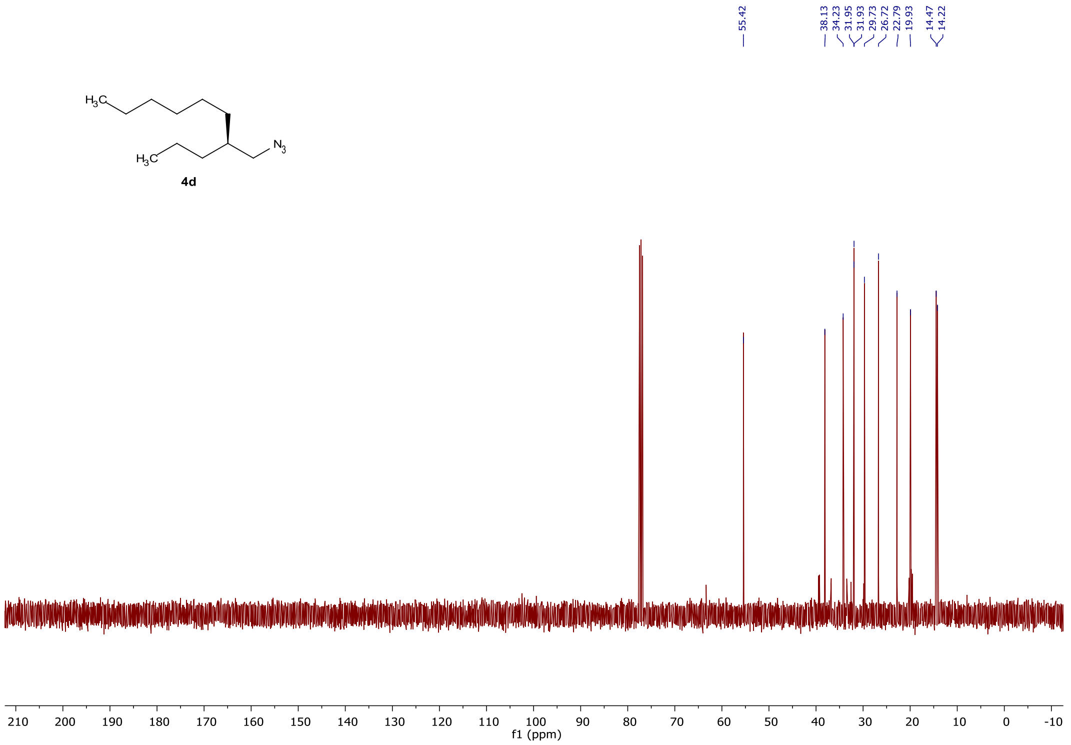

Supplementary Figure 34. NMR spectra of (*S*)-1-azido-2-propyloctane (4d)

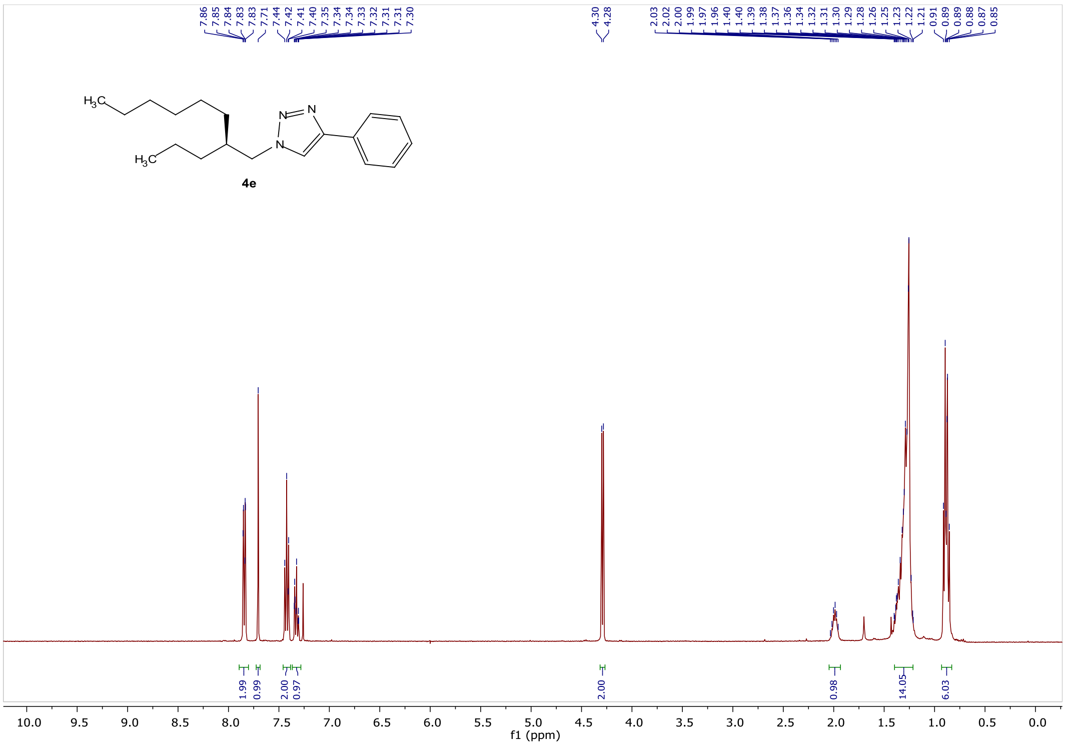

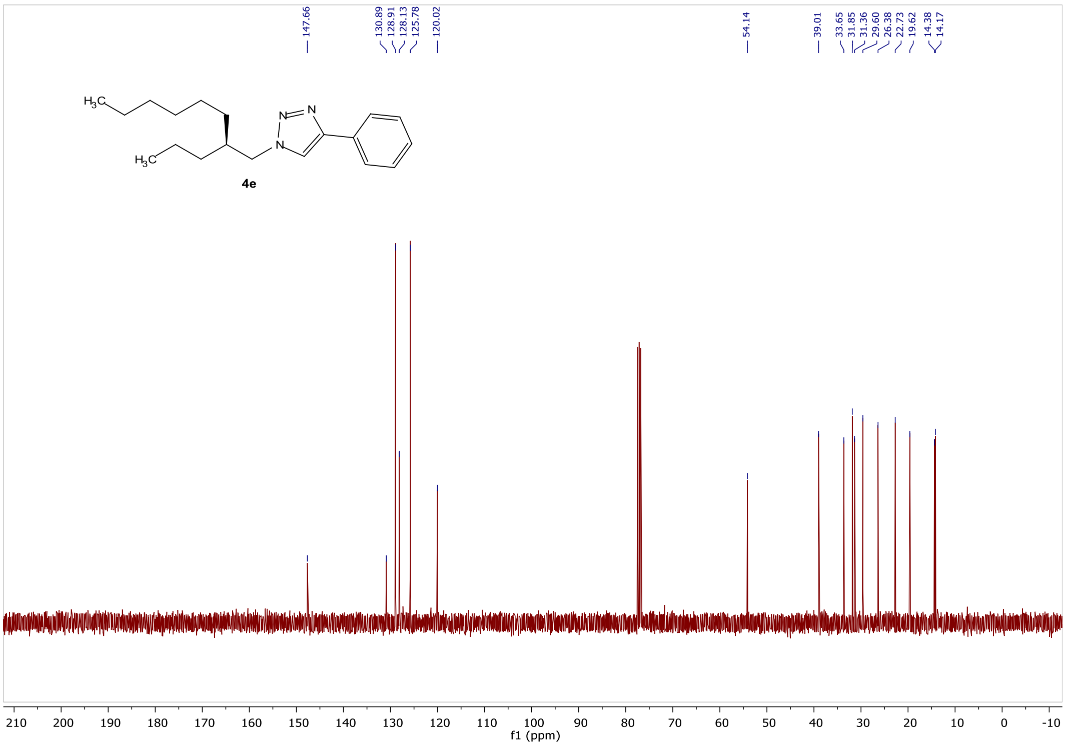

Supplementary Figure 35. NMR spectra of (*S*)-1-(2-propyl)octyl-4-phenyl-1*H*-1,2,3-triazole (4e)

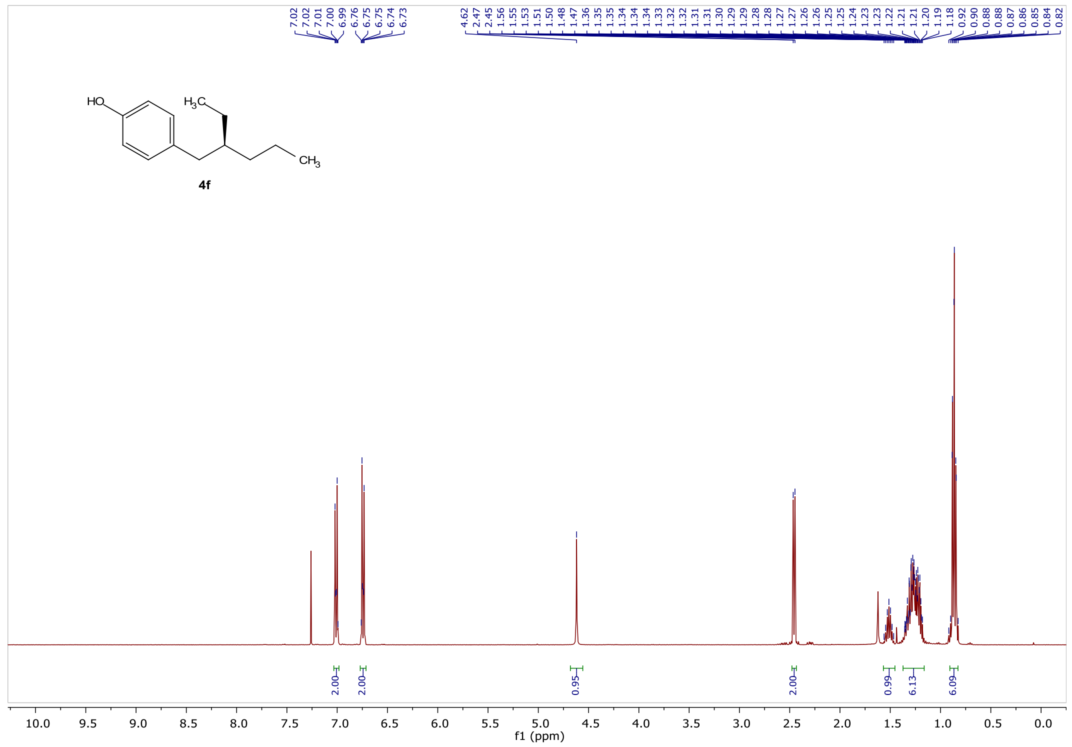

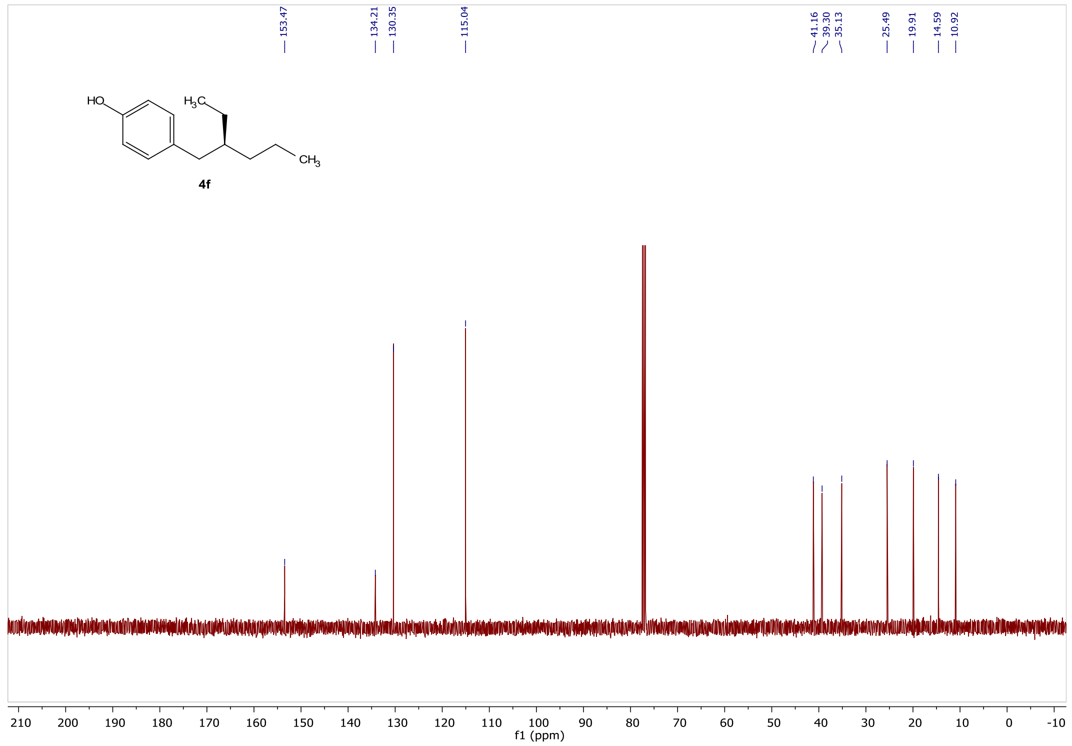

Supplementary Figure 36. NMR spectra of (*S*)-4-(2-ethylpentyl)phenol (4f)

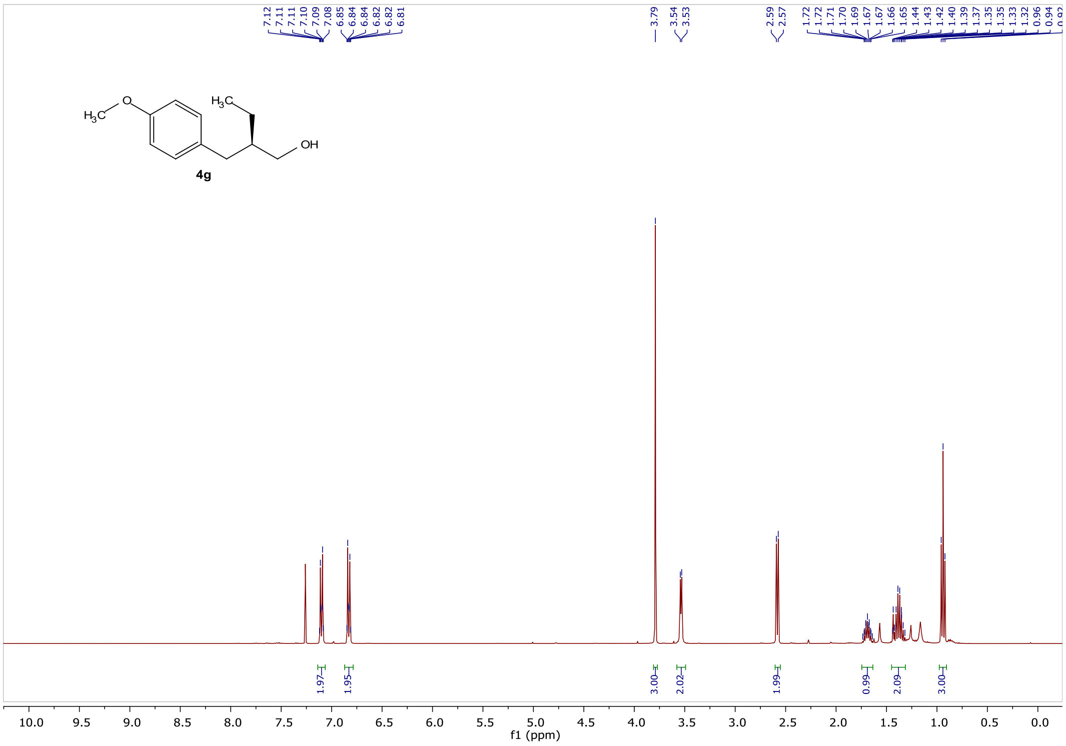

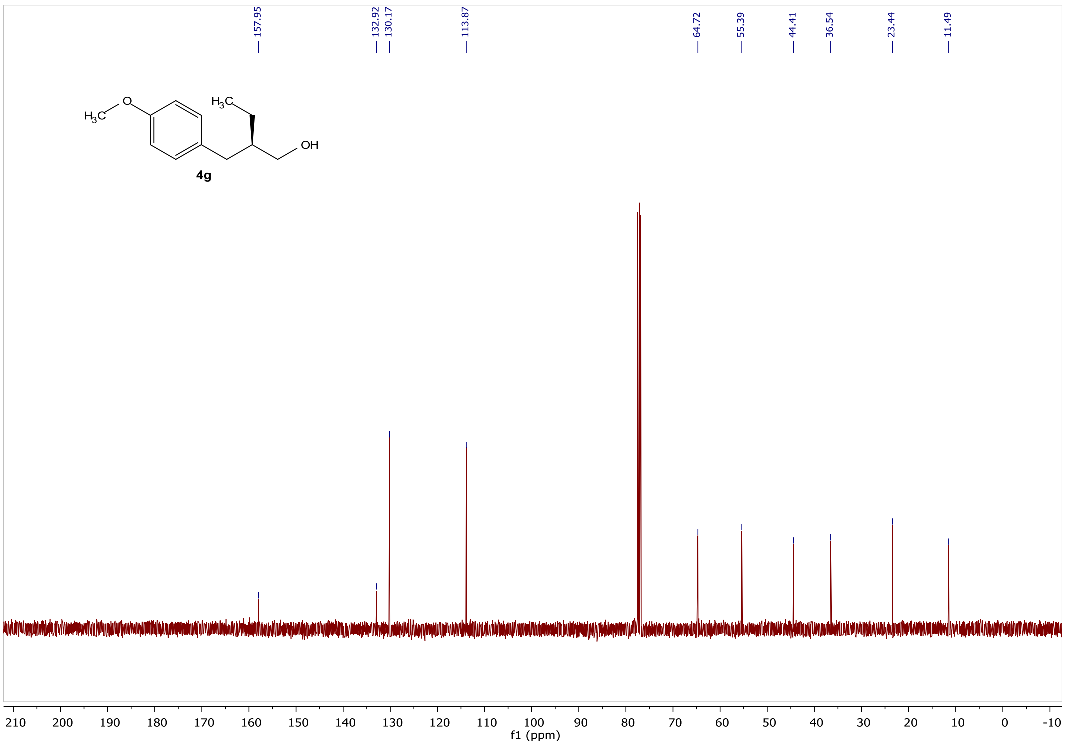

Supplementary Figure 37. NMR spectra of (*R*)-2-(4-methoxybenzyl)butan-1-ol (4g)

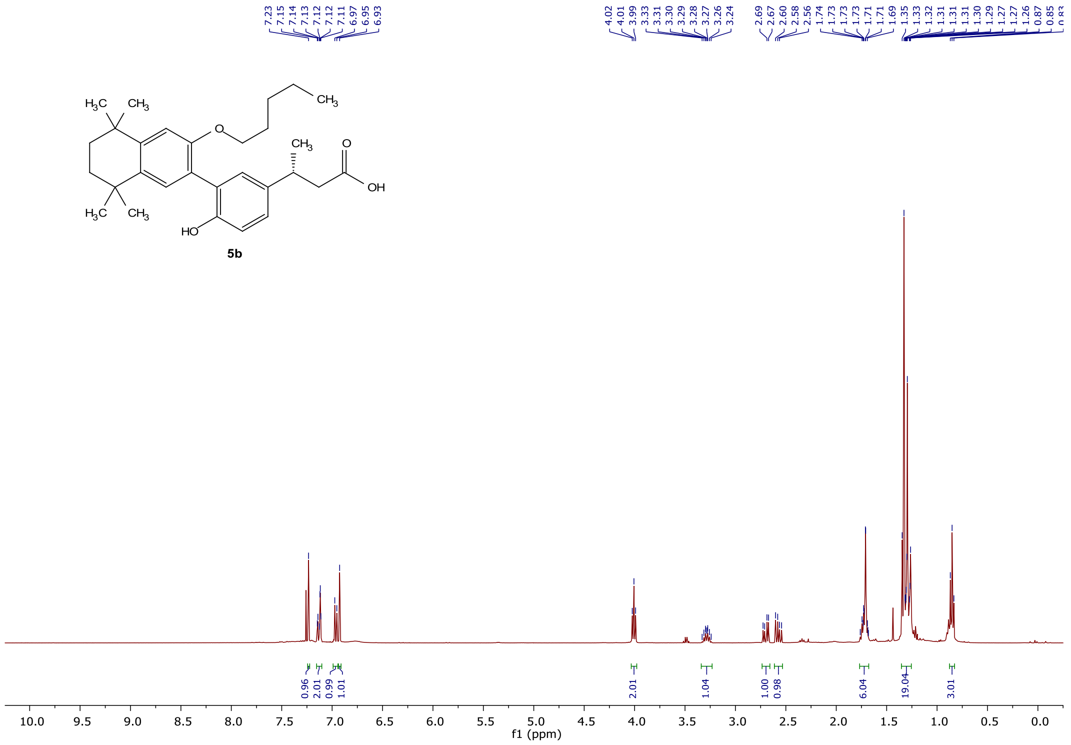

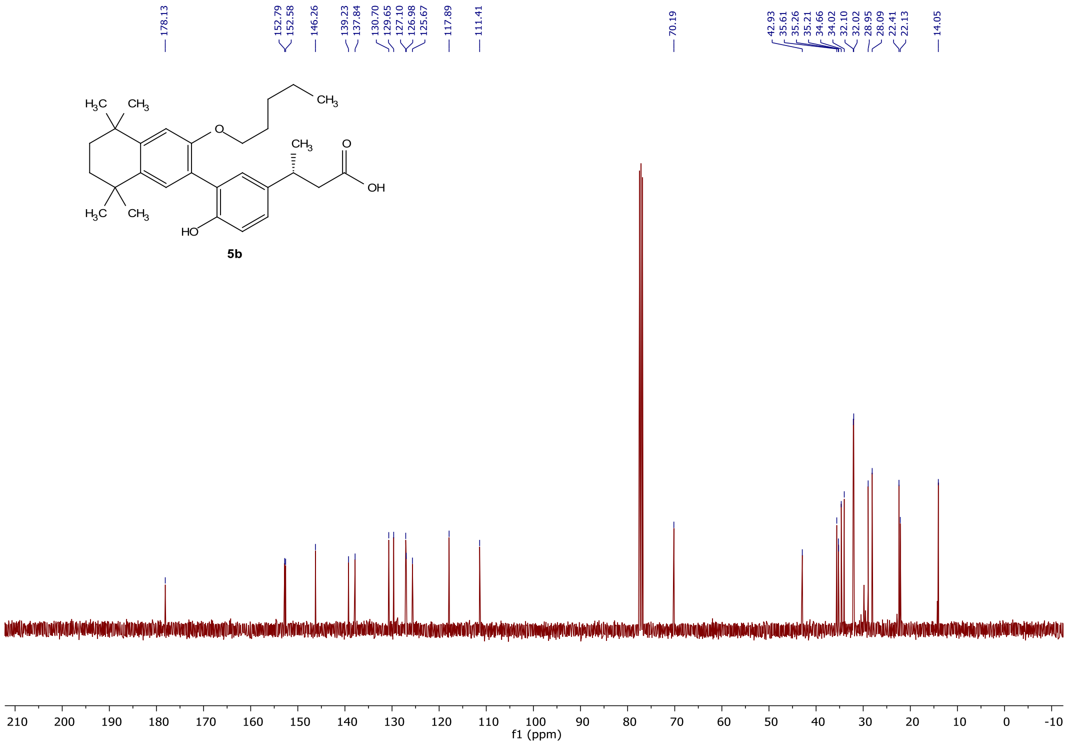

Supplementary Figure 38. NMR spectra of (R)-3-(4-hydroxy-3-(5,6,7,8-tetrahydro-5,5,8,8-tetramethyl-3-pentyloxy-2-naphthalenyl)phenyl) butanoic acid (5b)

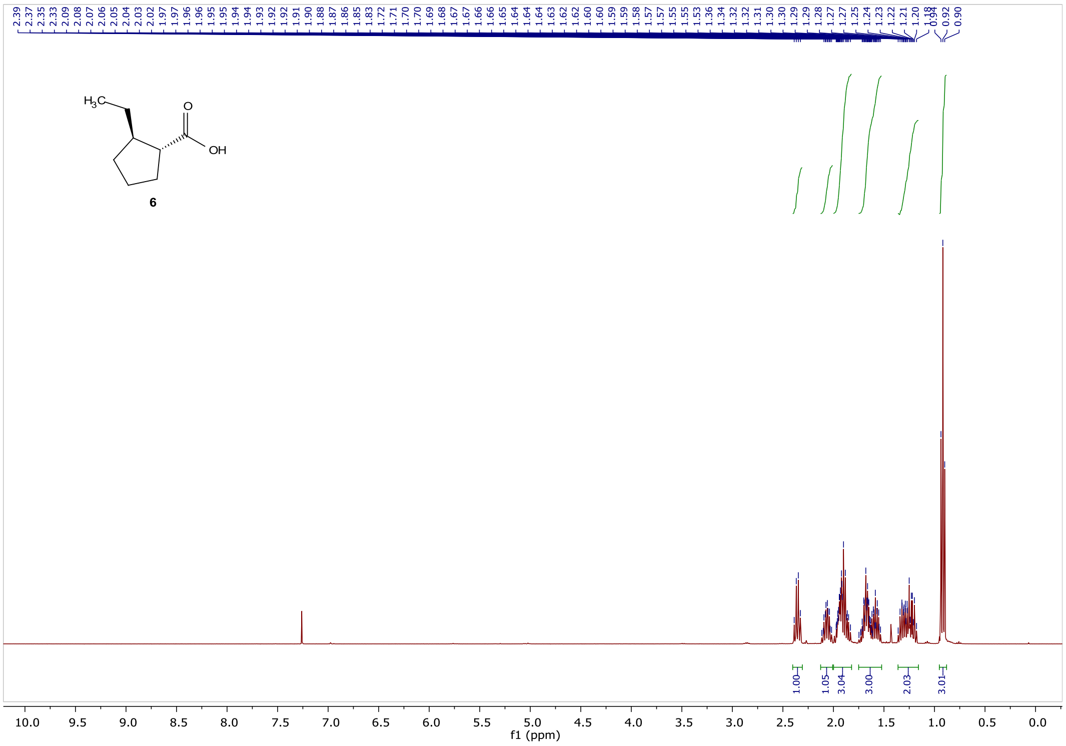

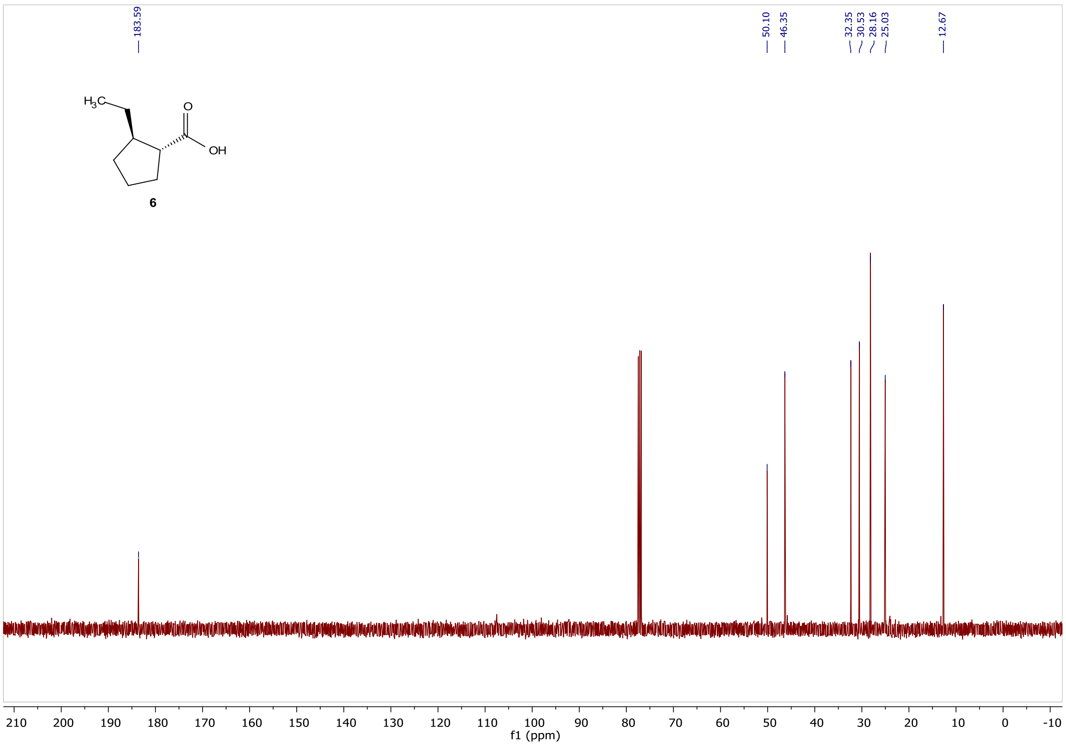

Supplementary Figure 39. NMR spectra of (1*R*,2*R*)-2-ethylcyclopentane-1-carboxylic acid (6)

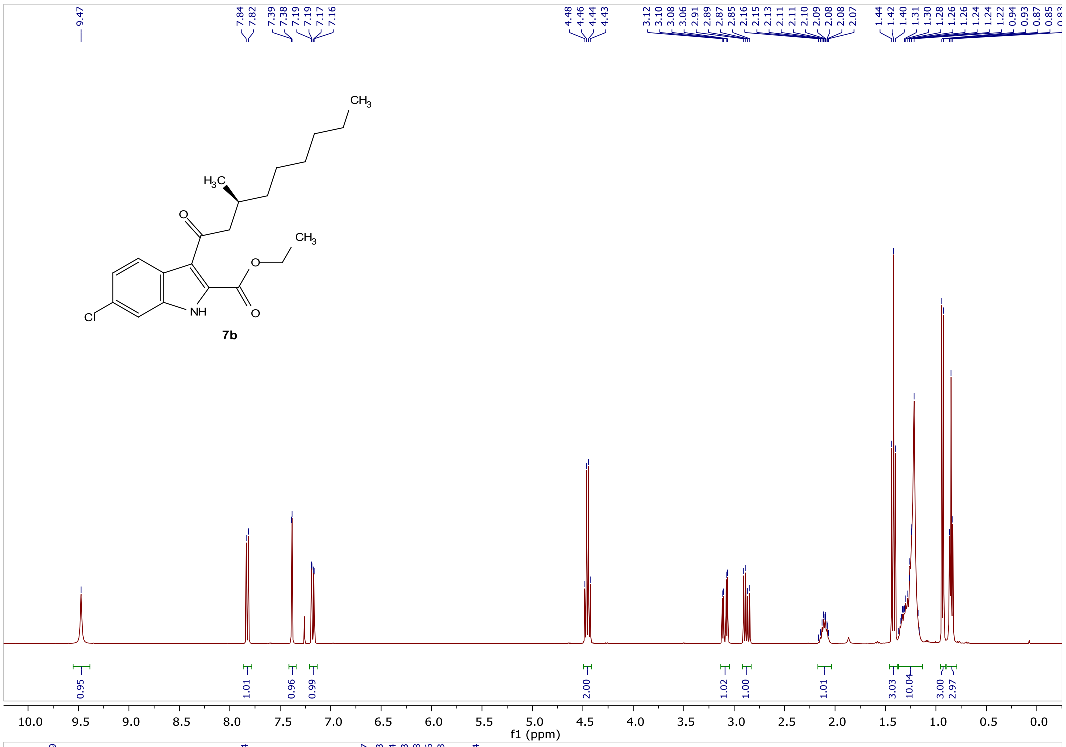

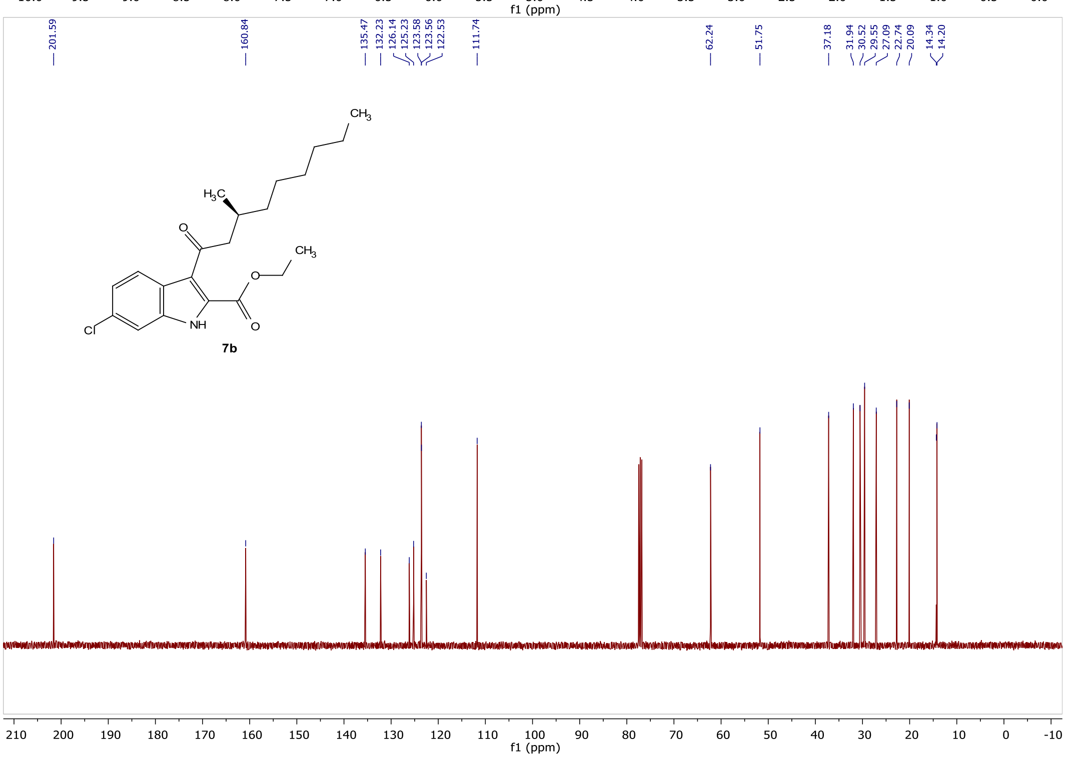

Supplementary Figure 40. NMR spectra of (*S*)-ethyl 6-chloro-3-(3-methylnonanoyl)-1*H*-indole-2-carboxylate (7b)

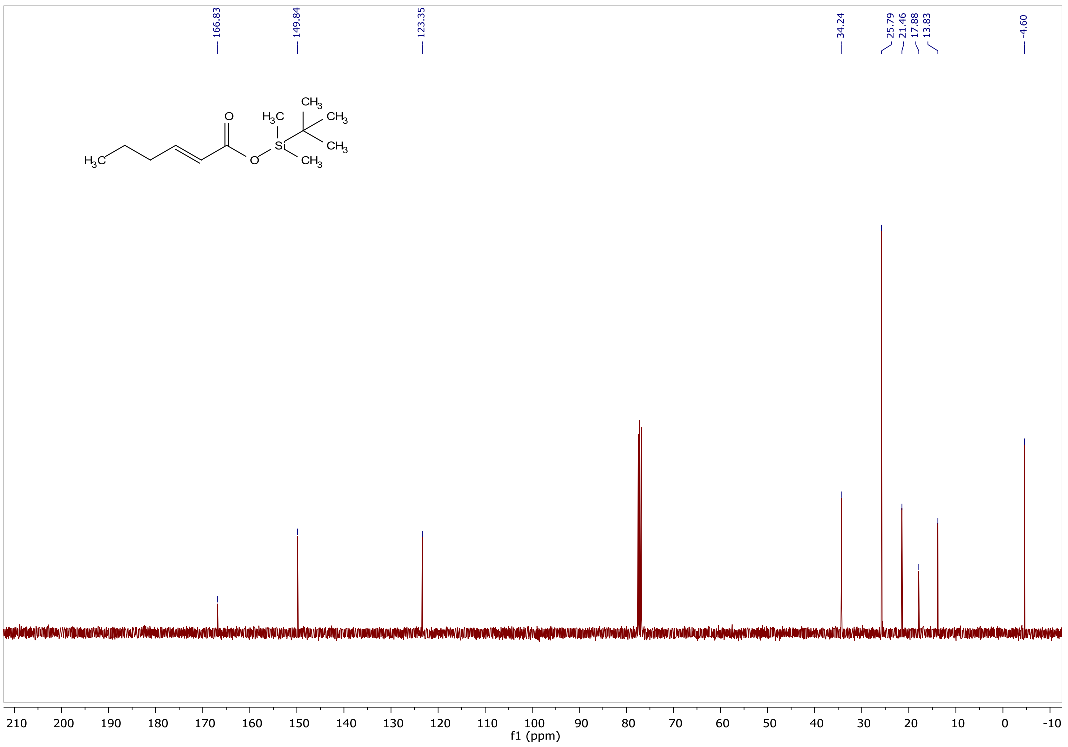

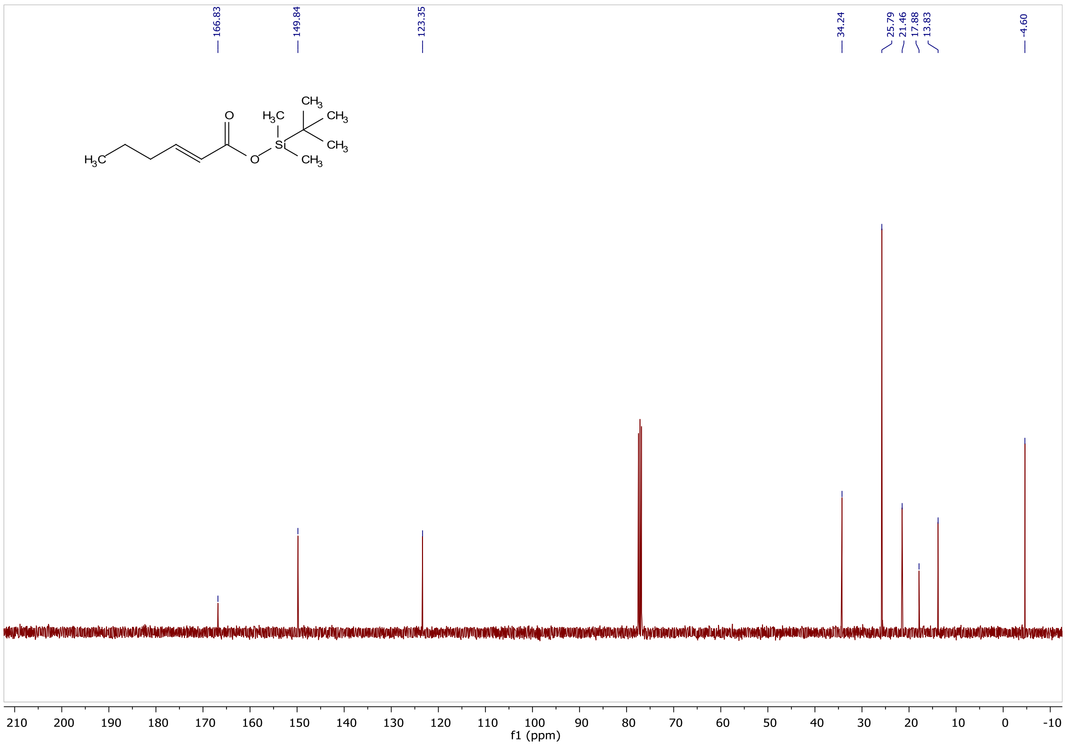

Supplementary Figure 41. NMR spectra of *t*-butyldimethylsilyl hex-2-enoate

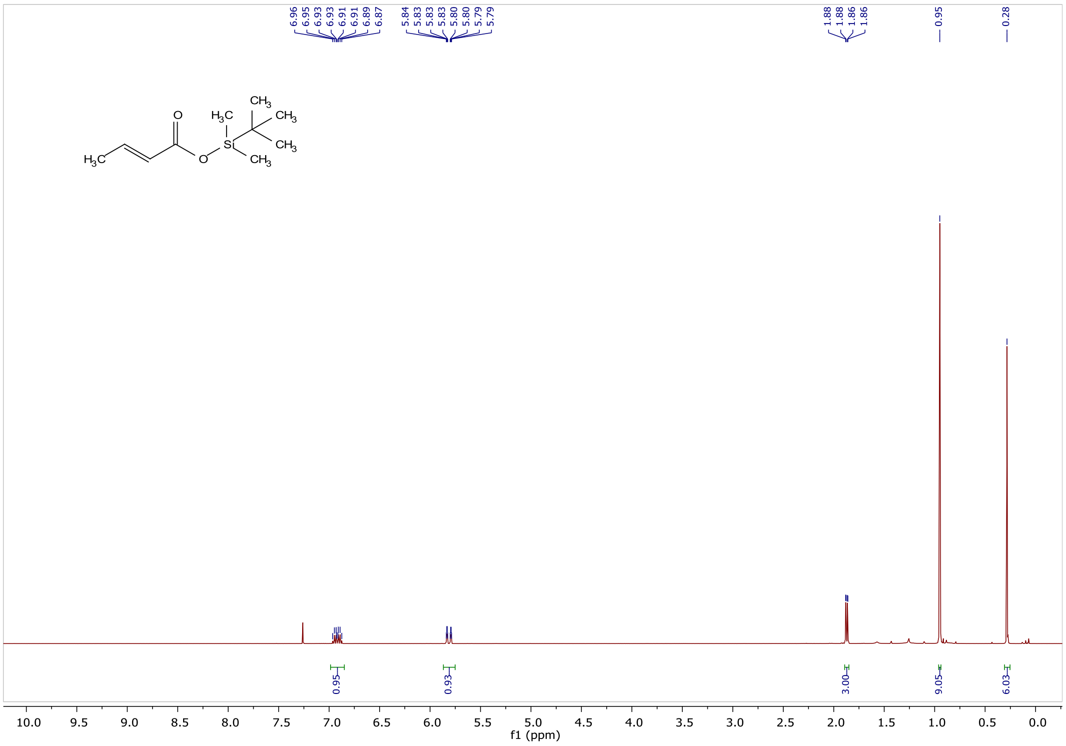

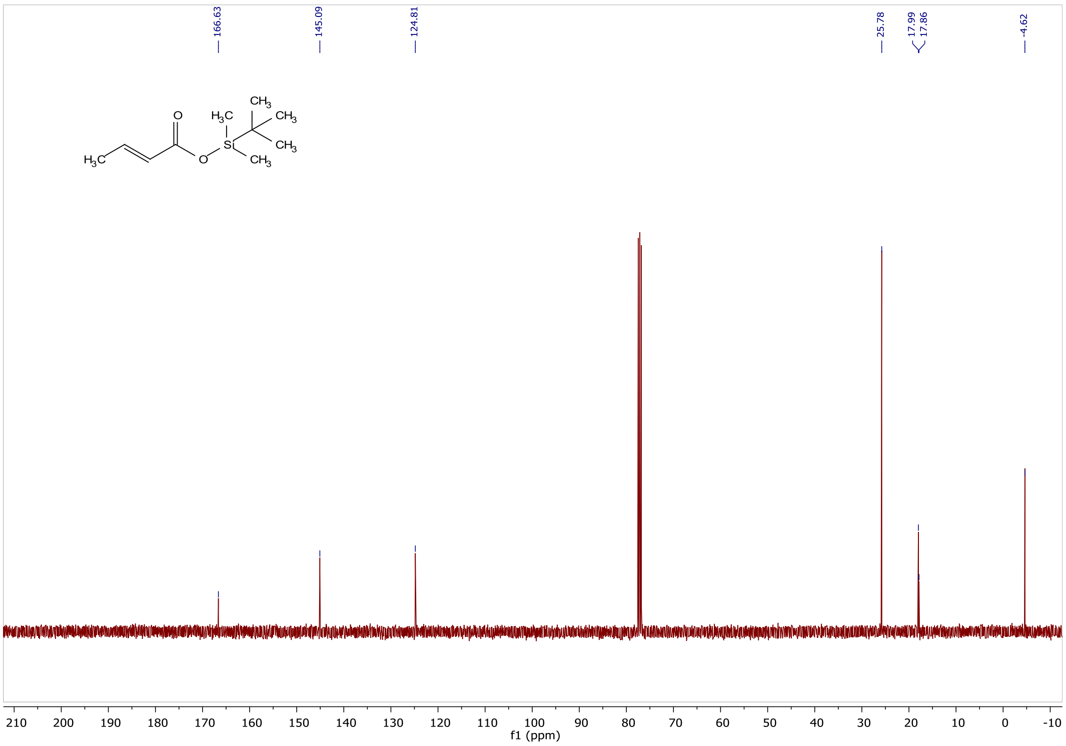

Supplementary Figure 42. NMR spectra of *t*-butyldimethylsilyl but-2-enoate

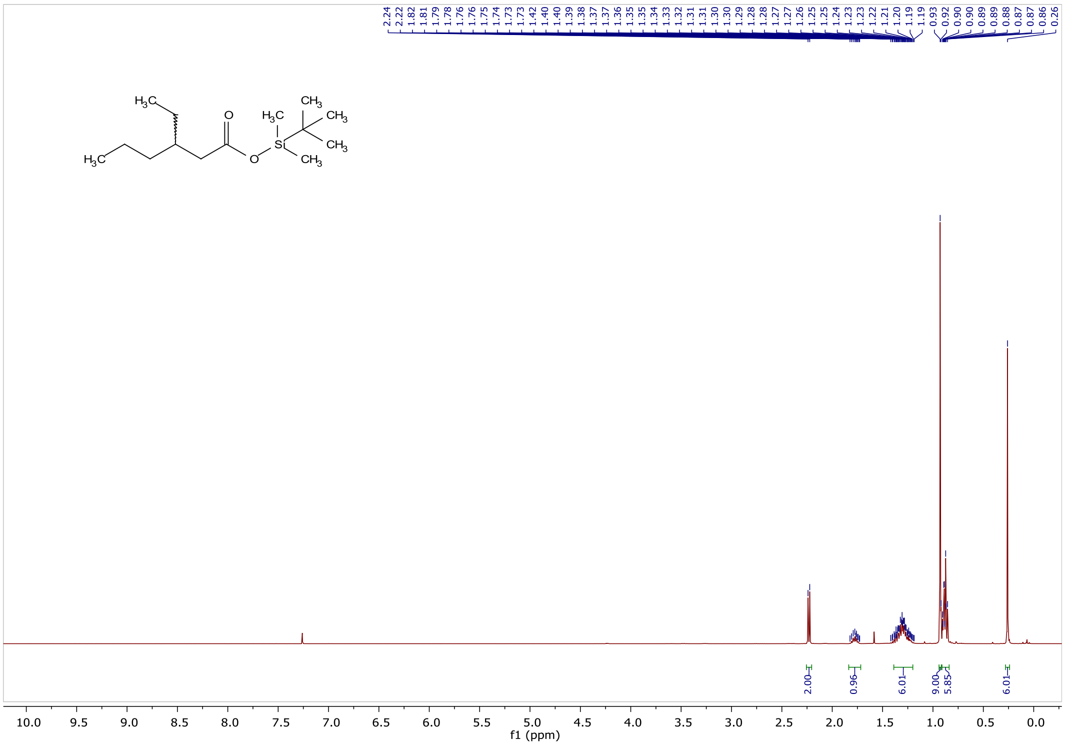

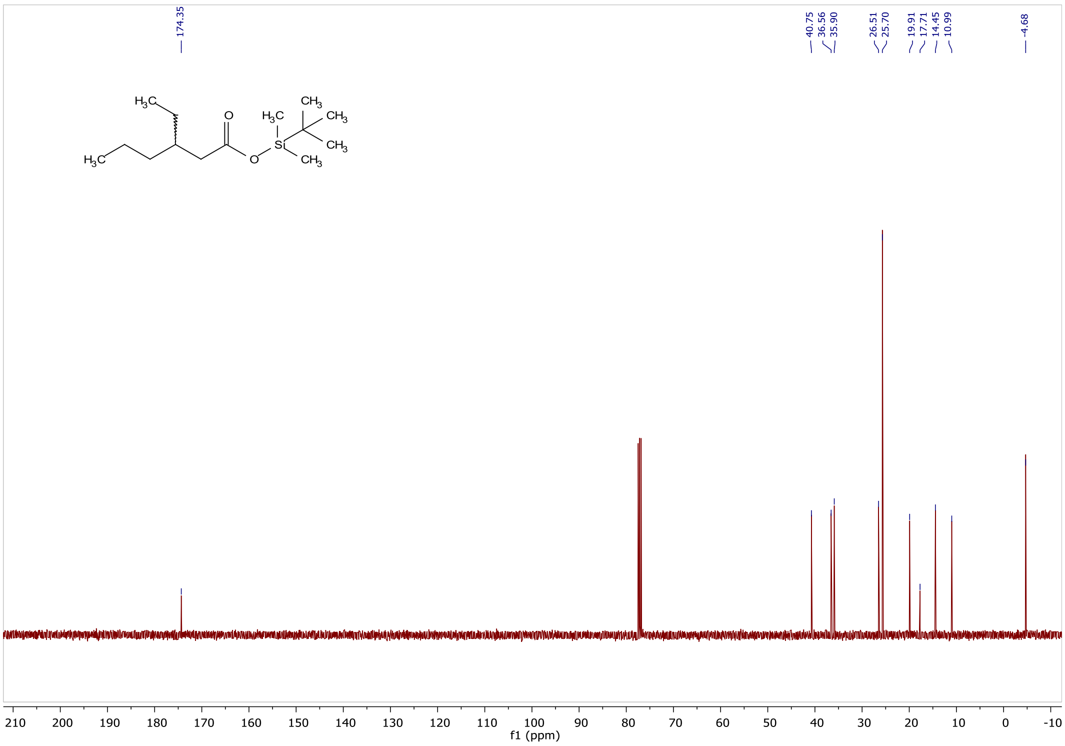

Supplementary Figure 43. NMR spectra of *t*-butyldimethylsilyl 3-ethylhexanoate

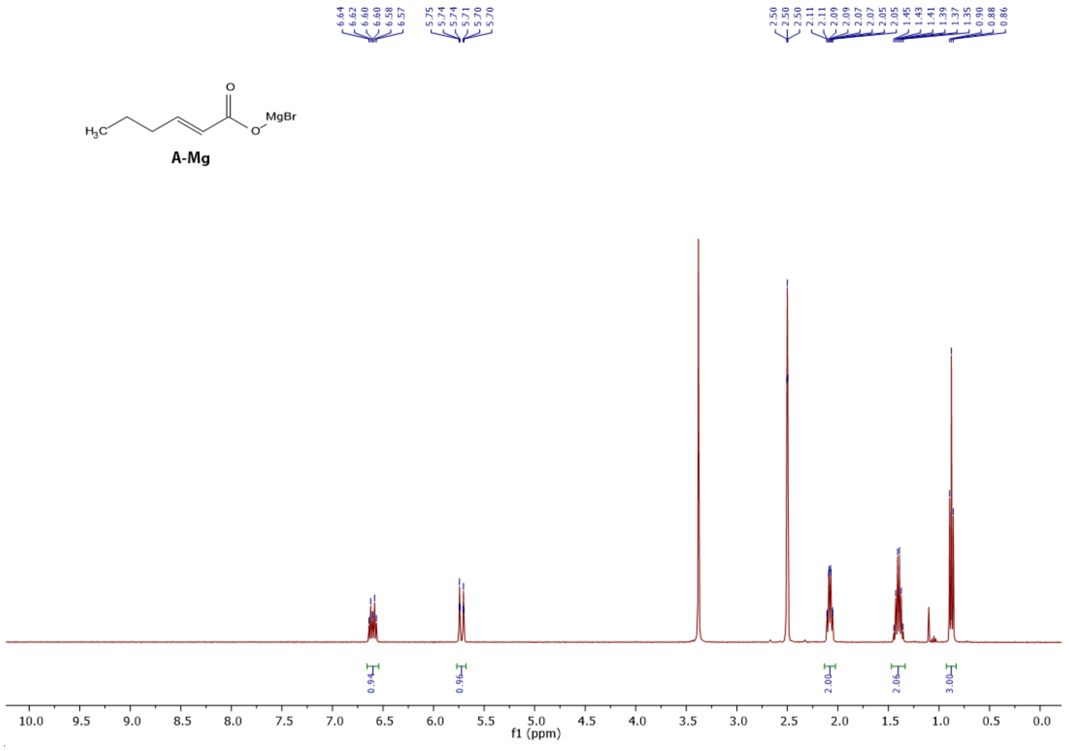

Supplementary Figure 44. ^{1}H NMR spectrum of magnesium bromide hex-2-enoate (A-Mg)

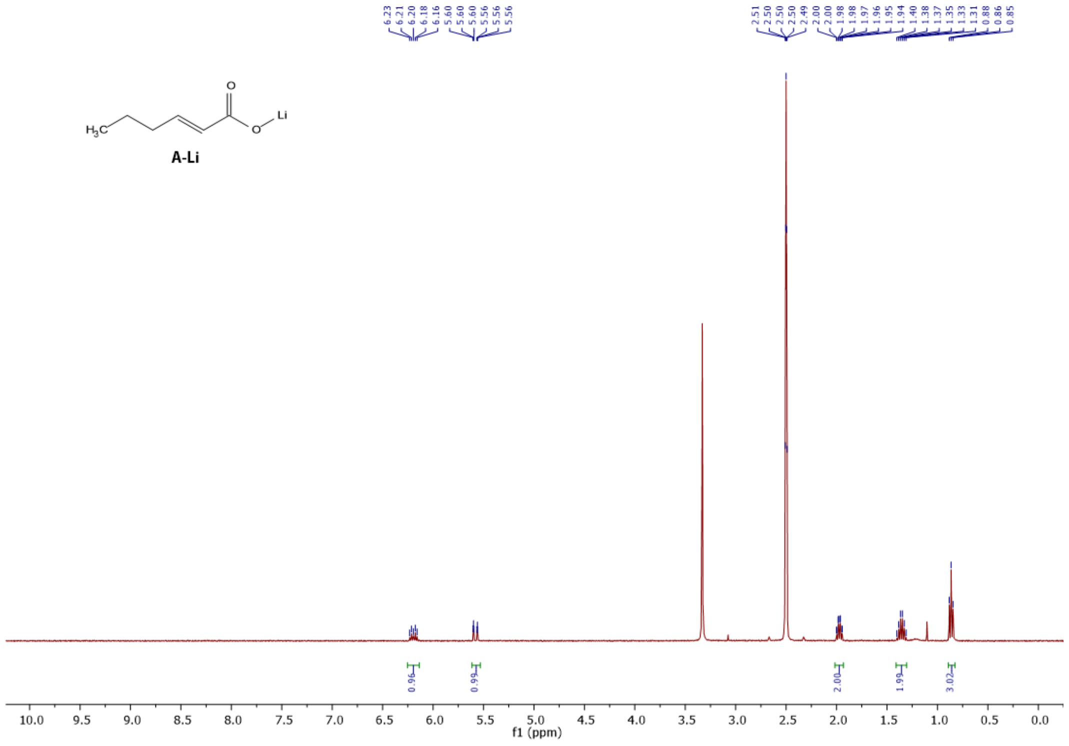

Supplementary Figure 45. ^{1}H NMR spectrum of lithium hex-2-enoate (A-Li)

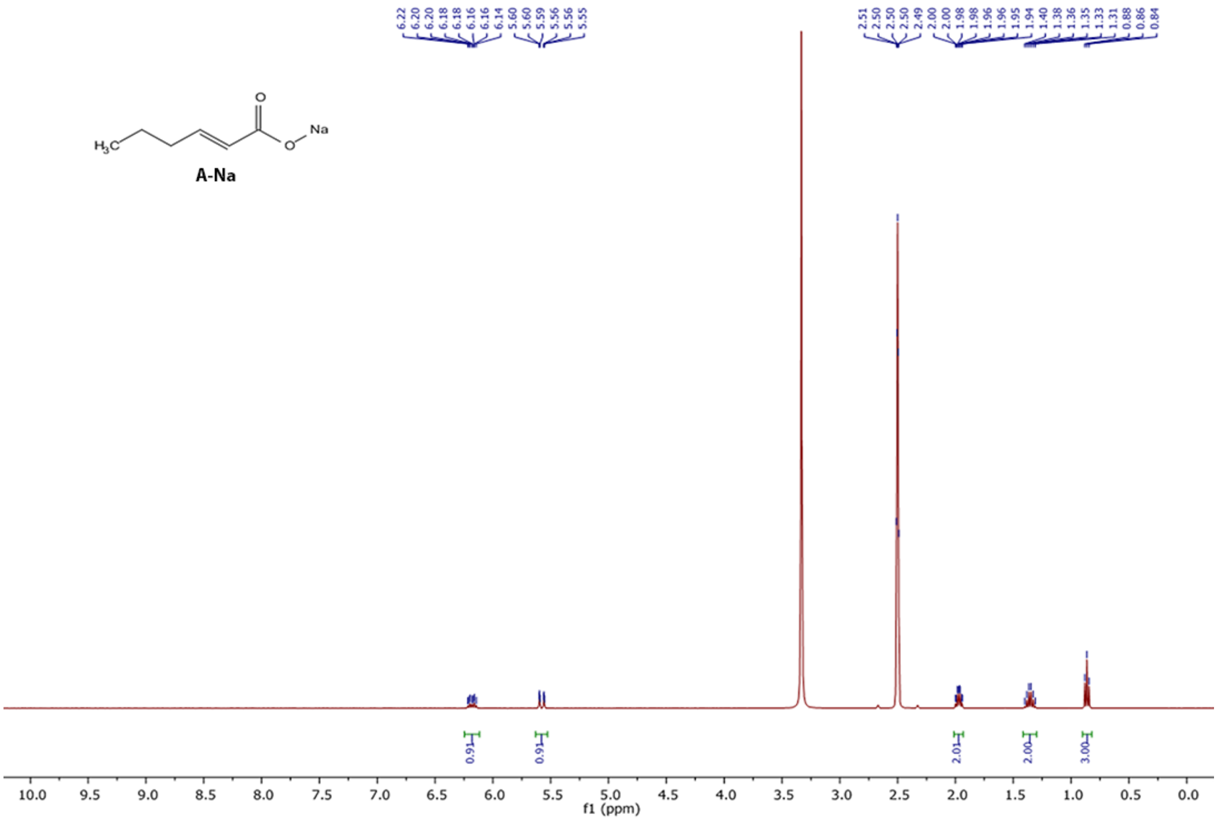

Supplementary Figure 46. ^{1}H NMR spectrum of sodium hex-2-enoate (A-Na)

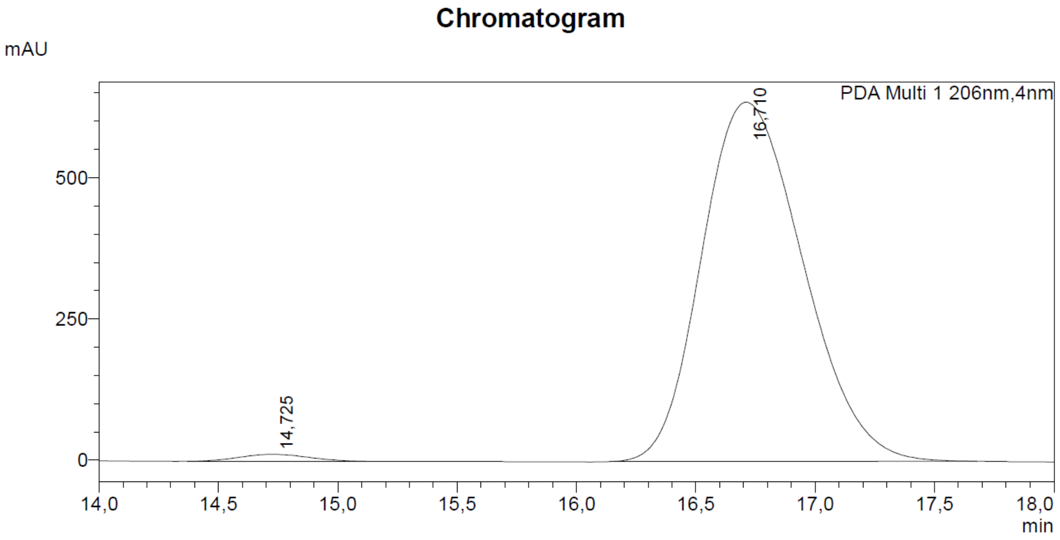

Peak Table

PDA Ch1 206nm

Peak#	Ret. Time	Area	Height	Conc.
1	14,725	252249	12453	1,326
2	16,710	18766796	635863	98,674
Total		19019046	648315	

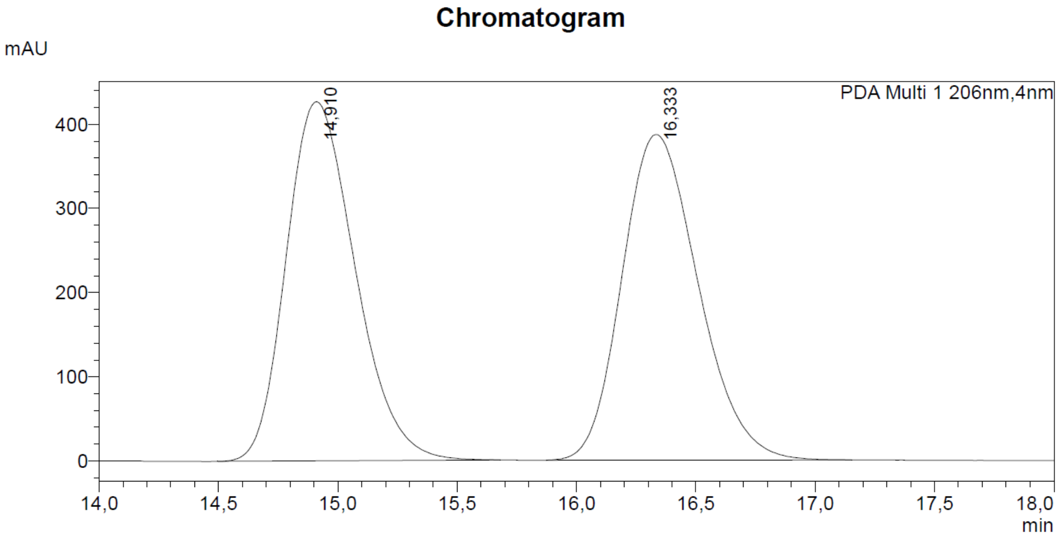

Peak Table

PDA Ch1 206nm

Peak#	Ret. Time	Area	Height	Conc.
1	14,910	8571570	426940	49,943
2	16,333	8591130	386997	50,057
Total		17162699	813937	

Supplementary Figure 47. HPLC spectra of (*R*)-3-Ethylhexanoic acid (2a)

Chromatogram

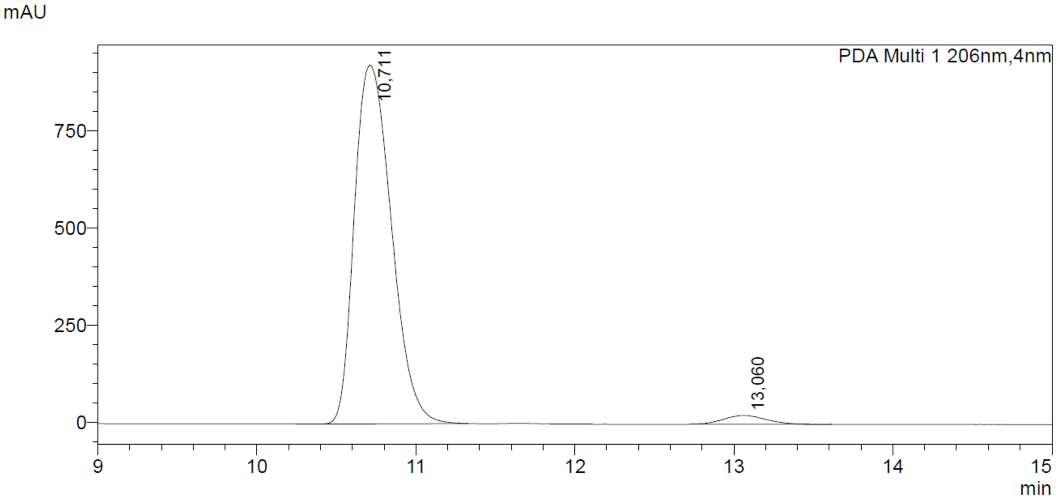

Peak Table

PDA Ch1 206nm

Peak#	Ret. Time	Area	Height	Conc.
1	10,711	14933807	922838	97,357
2	13,060	405348	22351	2,643
Total		15339155	945188	

Chromatogram

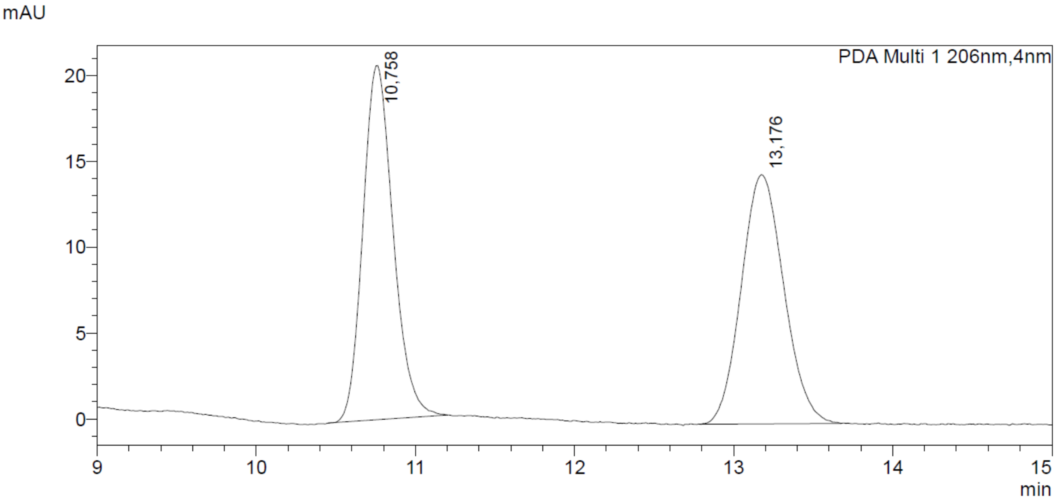

Peak Table

PDA Ch1 206nm

Peak#	Ret. Time	Area	Height	Conc.
1	10,758	272780	20621	50,278
2	13,176	269766	14512	49,722
Total		542546	35133	

Supplementary Figure 48. HPLC spectra of (*S*)-3-Methylpentanoic acid (2b)

Chromatogram

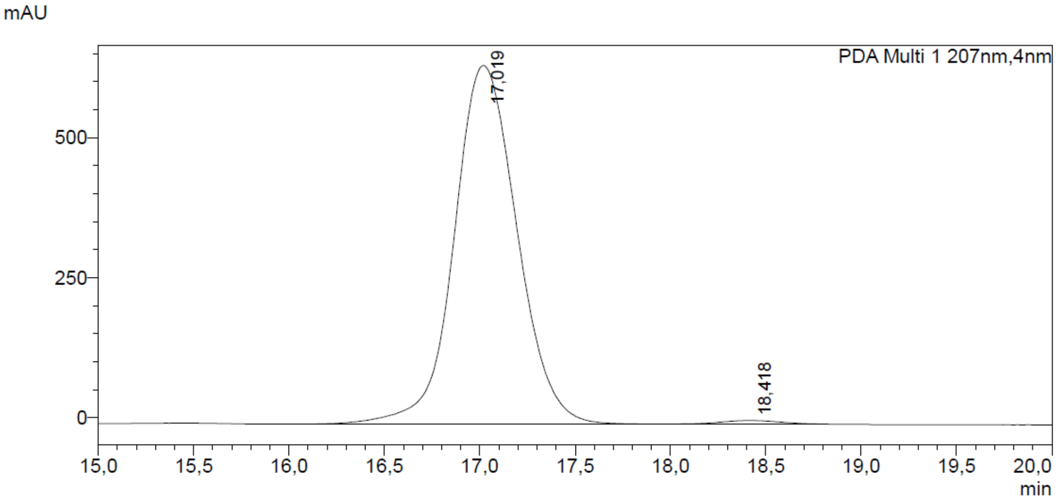

Peak Table

PDA Ch1 207nm

Peak#	Ret. Time	Area	Height	Conc.
1	17,019	14684549	639645	99,088
2	18,418	135167	6522	0,912
Total		14819716	646167	

Chromatogram

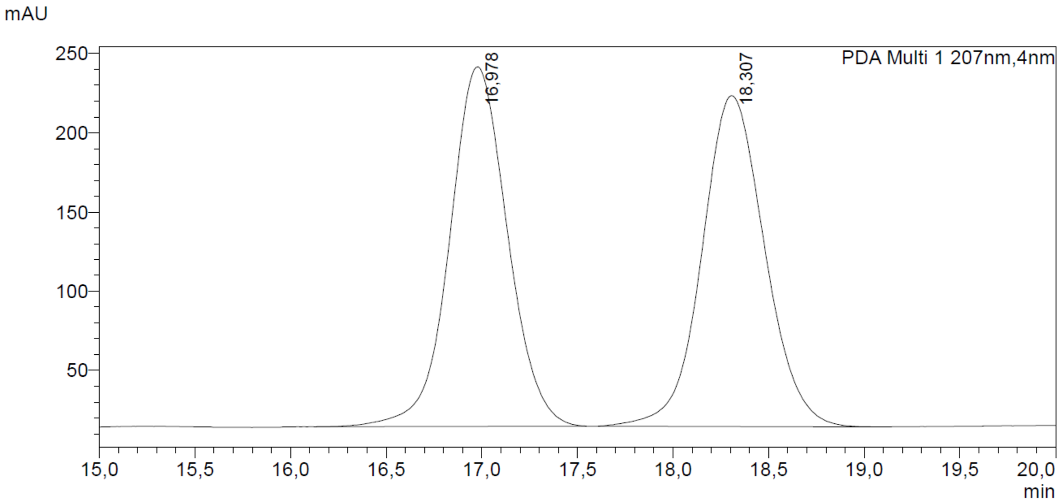

Peak Table

PDA Ch1 207nm

Peak#	Ret. Time	Area	Height	Conc.
1	16,978	4779732	227050	50,070
2	18,307	4766307	208865	49,930
Total		9546039	435915	

Supplementary Figure 49. HPLC spectra of (*S*)-3-Cyclohexylpentanoic acid (2c)

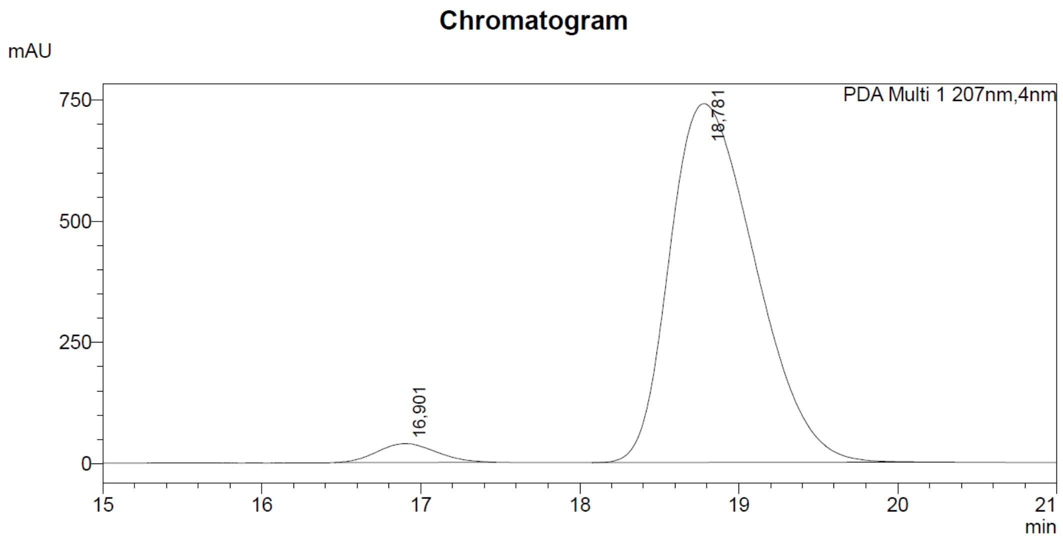

Peak Table

PDA Ch1 207nm

Peak#	Ret. Time	Area	Height	Conc.
1	16,901	998511	38749	3,480
2	18,781	27692170	739786	96,520
Total		28690682	778535	

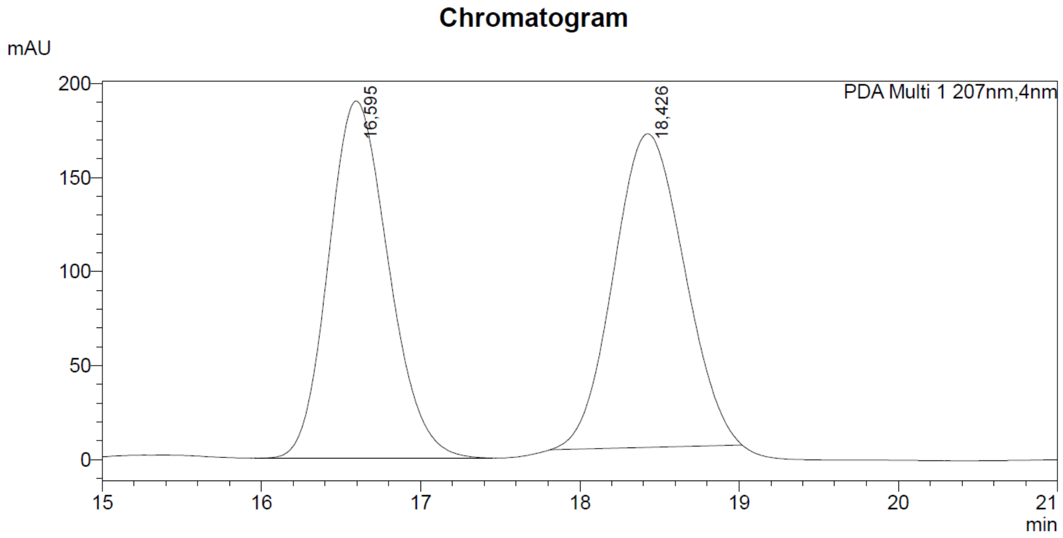

Peak Table

PDA Ch1 207nm

Peak#	Ret. Time	Area	Height	Conc.
1	16,595	4981287	190027	49,174
2	18,426	5148698	166796	50,826
Total		10129986	356823	

Supplementary Figure 50. HPLC spectra of (*S*)-3-Cyclopropylpentanoic acid (2d)

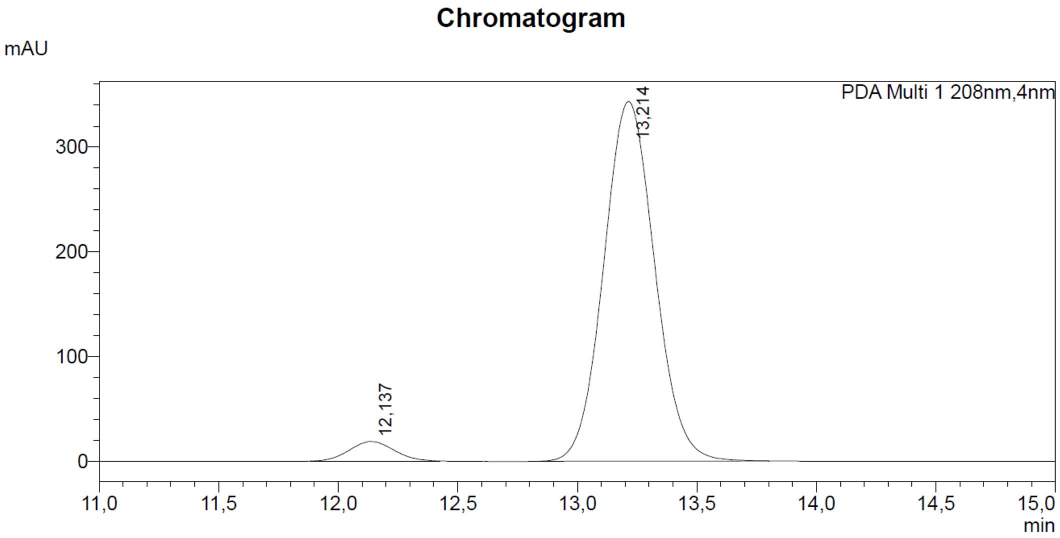

Peak Table

PDA Ch1 208nm

Peak#	Ret. Time	Area	Height	Conc.
1	12,137	244091	18750	4,568
2	13,214	5098947	343902	95,432
Total		5343038	362652	

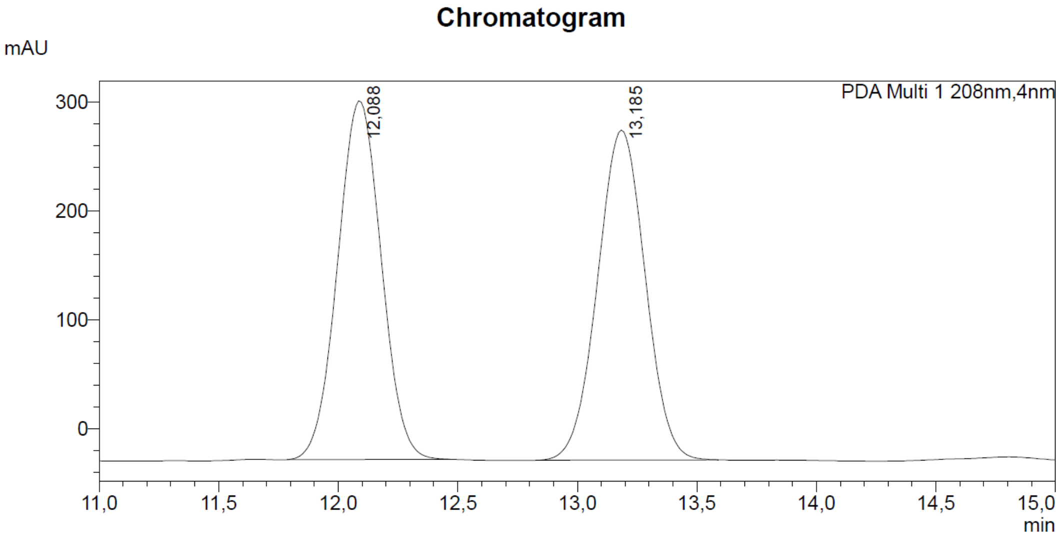

Peak Table

PDA Ch1 208nm

Peak#	Ret. Time	Area	Height	Conc.
1	12,088	4186813	329363	49,939
2	13,185	4197003	302801	50,061
Total		8383816	632165	

Supplementary Figure 51. HPLC spectra of (*R*)-3-Phenylpentanoic acid (2e)

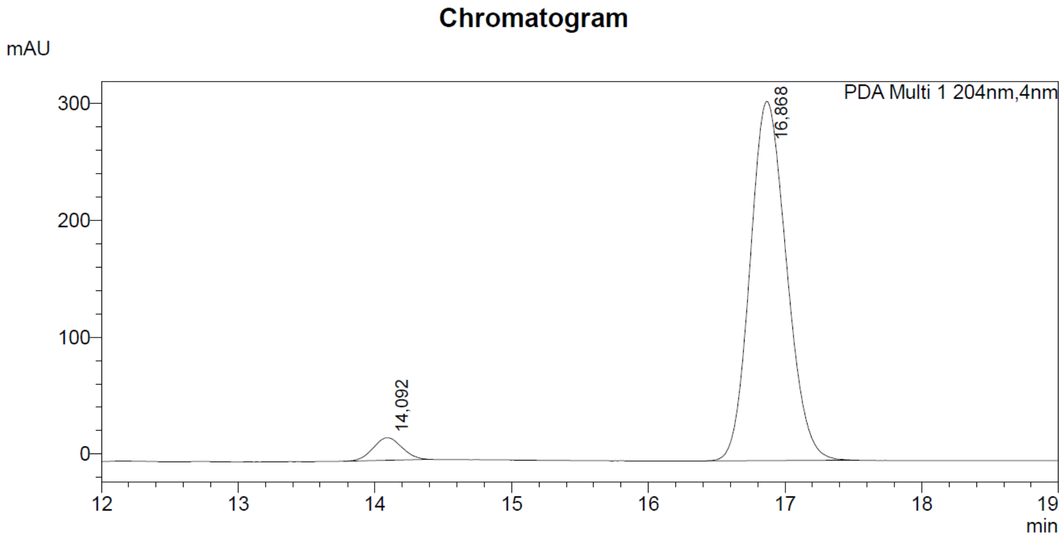

Peak Table

PDA Ch1 204nm

Peak#	Ret. Time	Area	Height	Conc.
1	14,092	281915	19290	4,719
2	16,868	5692649	307484	95,281
Total		5974564	326775	

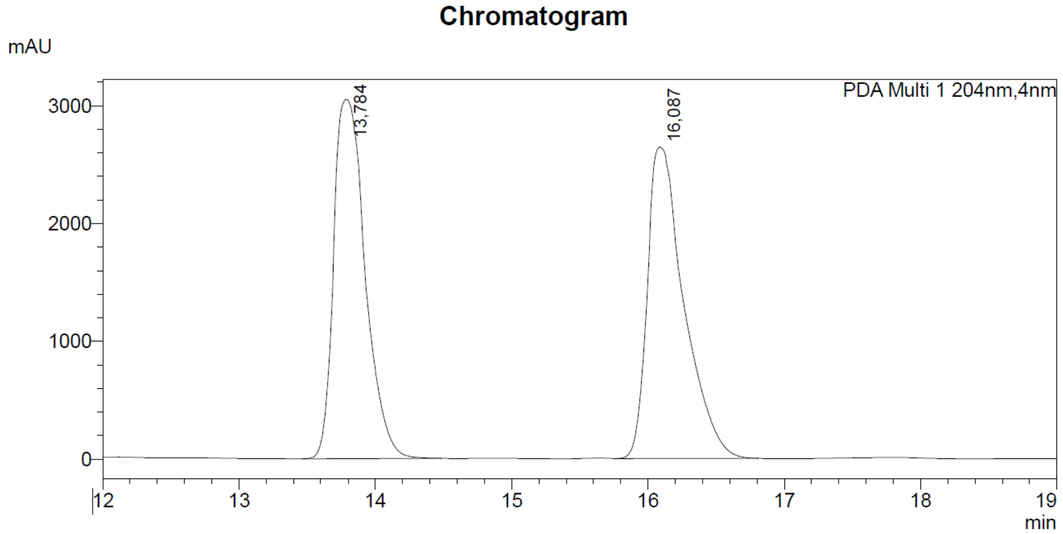

Peak Table

PDA Ch1 204nm

Peak#	Ret. Time	Area	Height	Conc.
1	13,784	49000761	3048491	49,854
2	16,087	49287705	2642449	50,146
Total		98288466	5690940	

Supplementary Figure 52. HPLC spectra of (*R*)-3-(4-Methoxyphenyl)pentanoic acid (2f)

Chromatogram

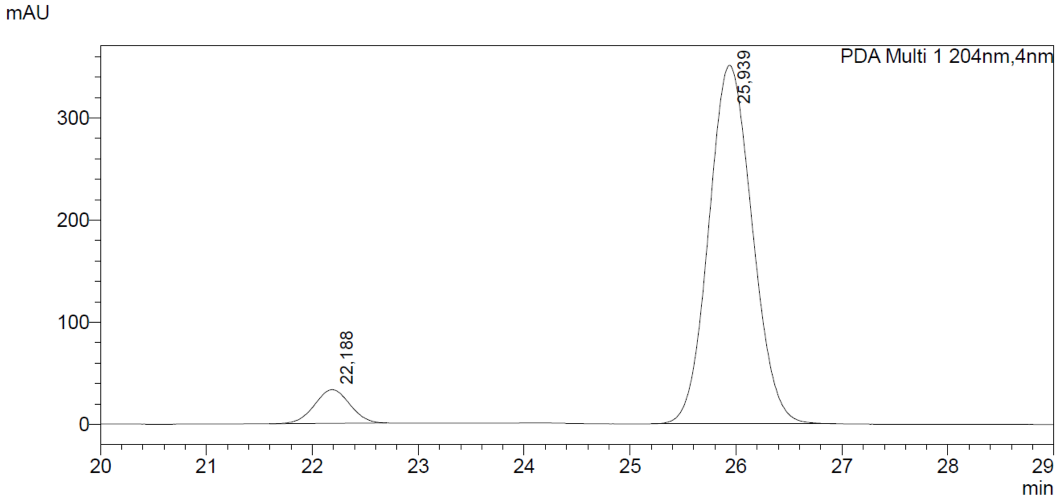

Peak Table

PDA Ch1 204nm

Peak#	Ret. Time	Area	Height	Conc.
1	22,188	766731	32984	7,117
2	25,939	10005850	350960	92,883
Total		10772582	383943	

Chromatogram

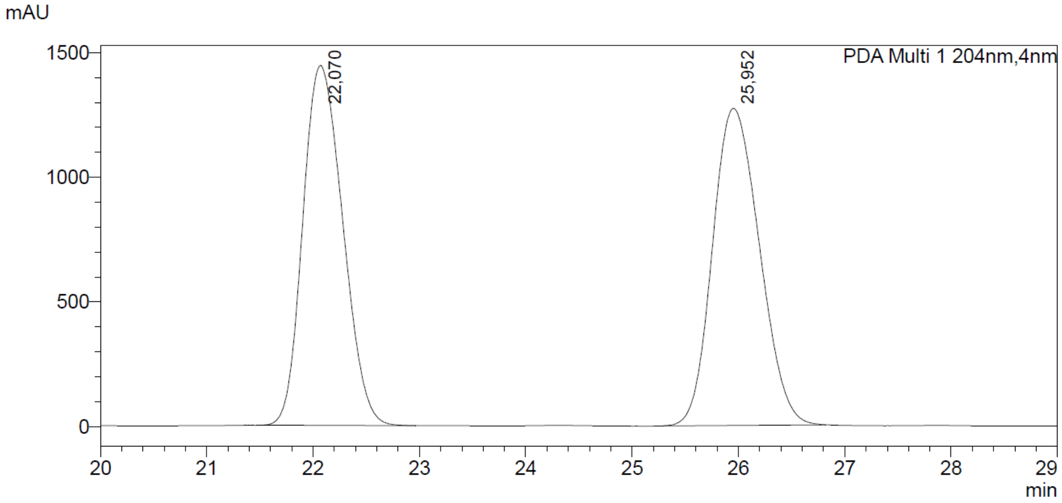

Peak Table

PDA Ch1 204nm

Peak#	Ret. Time	Area	Height	Conc.
1	22,070	37750765	1445439	49,138
2	25,952	39075786	1272701	50,862
Total		76826551	2718139	

Supplementary Figure 53. HPLC spectra of (*R*)-3-(3-Bromophenyl)pentanoic acid (2g)

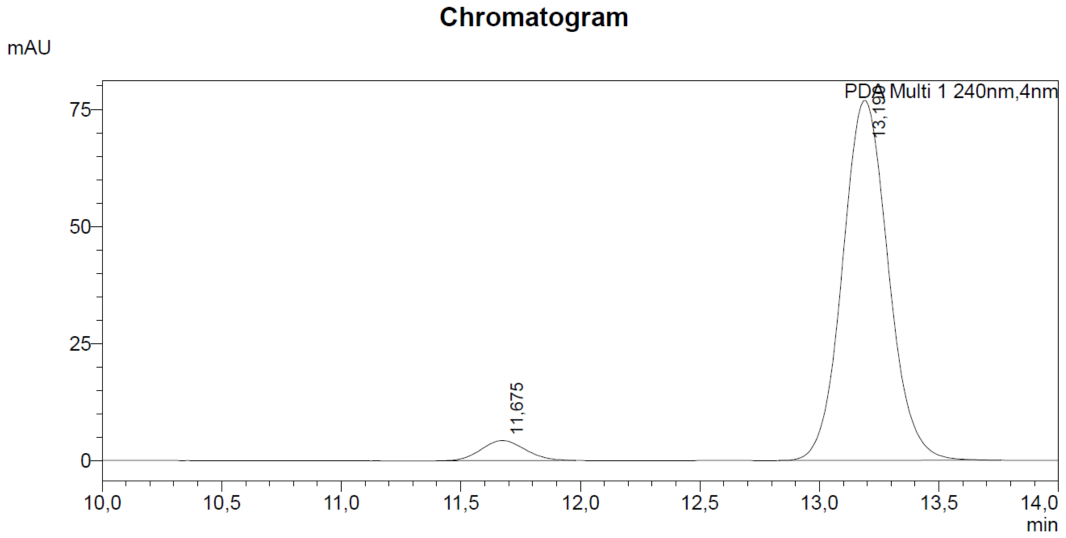

Peak Table

PDA Ch1 240nm

Peak#	Ret. Time	Area	Height	Conc.
1	11,675	54697	4274	5,078
2	13,190	1022339	76748	94,922
Total		1077035	81022	

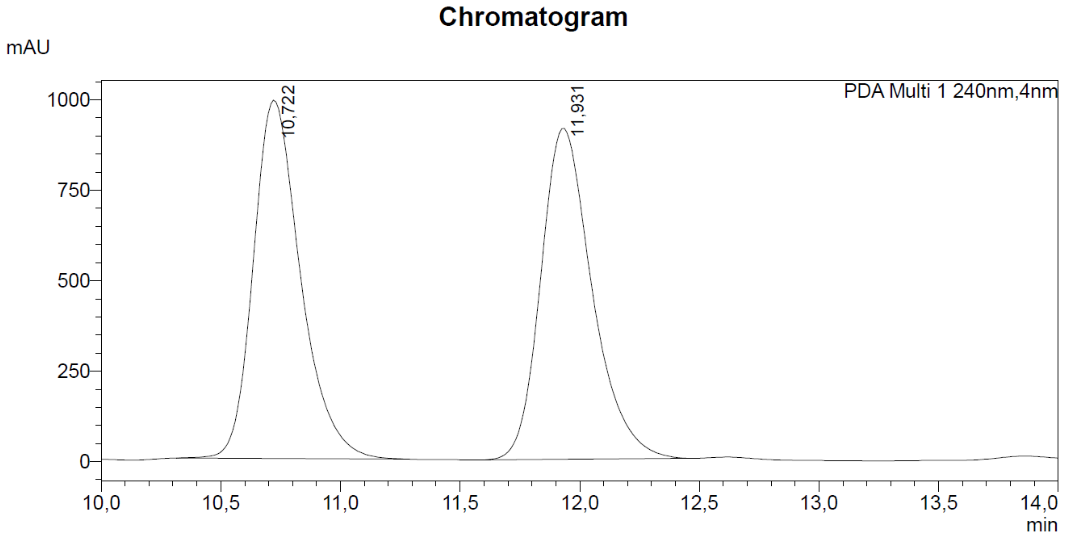

Peak Table

PDA Ch1 240nm

Peak#	Ret. Time	Area	Height	Conc.
1	10,722	13621413	990455	49,906
2	11,931	13672603	914292	50,094
Total		27294016	1904747	

Supplementary Figure 54. HPLC spectra of (*R*)-3-(Thiophen-2-yl)pentanoic acid (2h)

Chromatogram

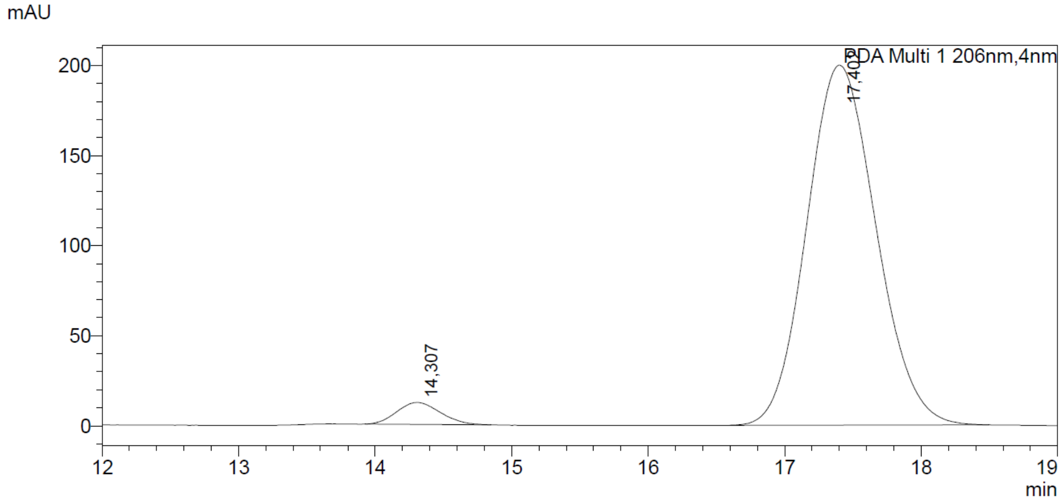

Peak Table

PDA Ch1 206nm

Peak#	Ret. Time	Area	Height	Conc.
1	14,307	269126	12175	3,672
2	17,402	7059714	199844	96,328
Total		7328841	212020	

Chromatogram

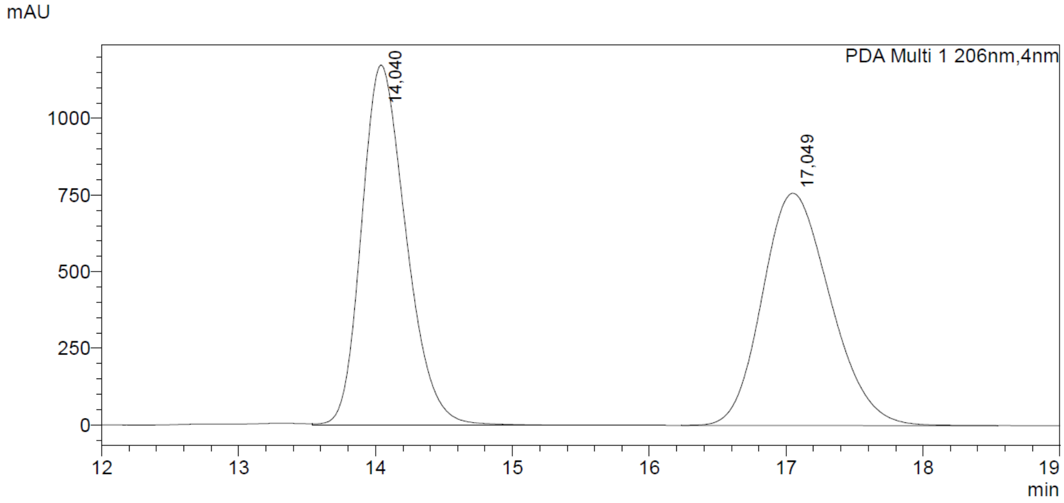

Peak Table

PDA Ch1 206nm

Peak#	Ret. Time	Area	Height	Conc.
1	14,040	26626496	1174817	50,433
2	17,049	26169539	757431	49,567
Total		52796035	1932248	

Supplementary Figure 55. HPLC spectra of (*R*)-3-(Thiophen-3-yl)pentanoic acid (2i)

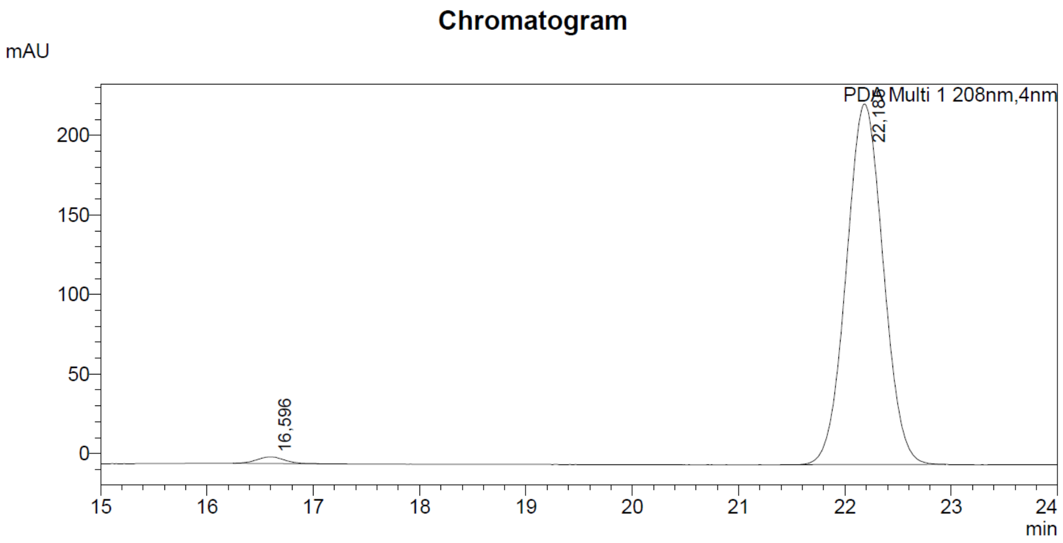

Peak Table

PDA Ch1 208nm

Peak#	Ret. Time	Area	Height	Conc.
1	16,596	73330	4133	1,320
2	22,185	5481356	226795	98,680
Total		5554686	230928	

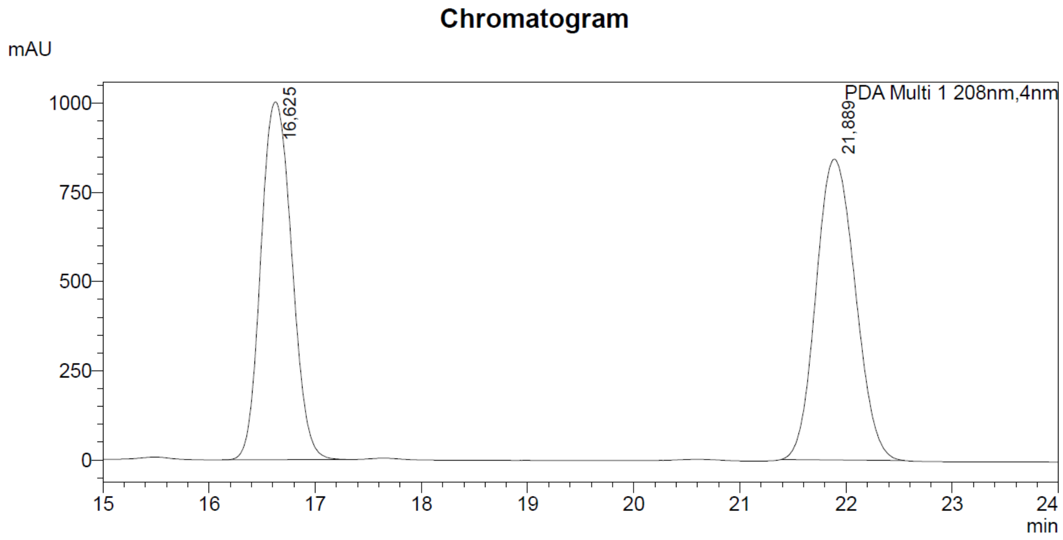

Peak Table

PDA Ch1 208nm

Peak#	Ret. Time	Area	Height	Conc.
1	16,625	20324527	1002704	48,374
2	21,889	21690595	843175	51,626
Total		42015123	1845879	

Supplementary Figure 56. HPLC spectra of (*R*)-3-Benzylpentanoic acid (2j)

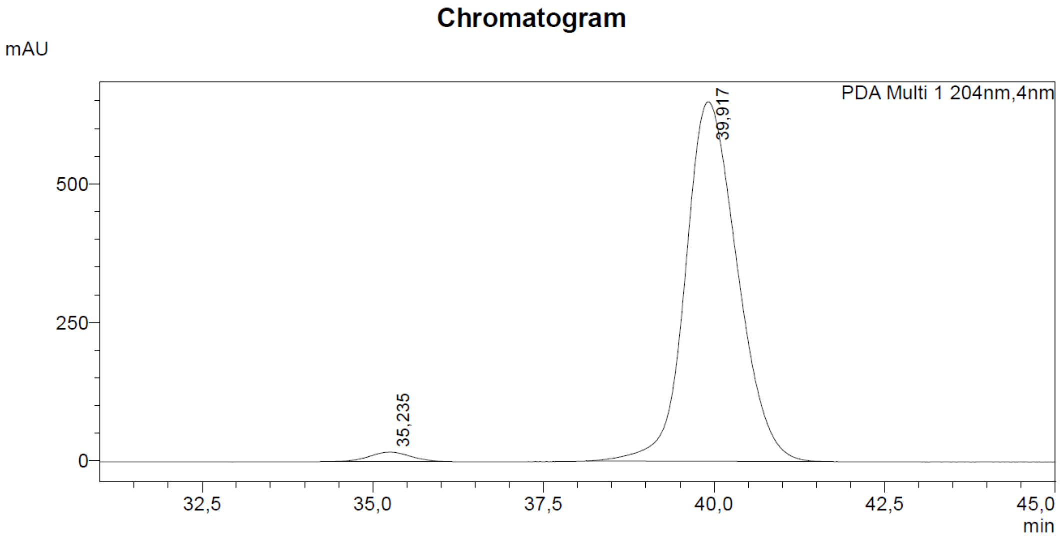

Peak Table

PDA Ch1 204nm

Peak#	Ret. Time	Area	Height	Conc.
1	35,235	698942	16981	2,000
2	39,917	34240886	648271	98,000
Total		34939828	665251	

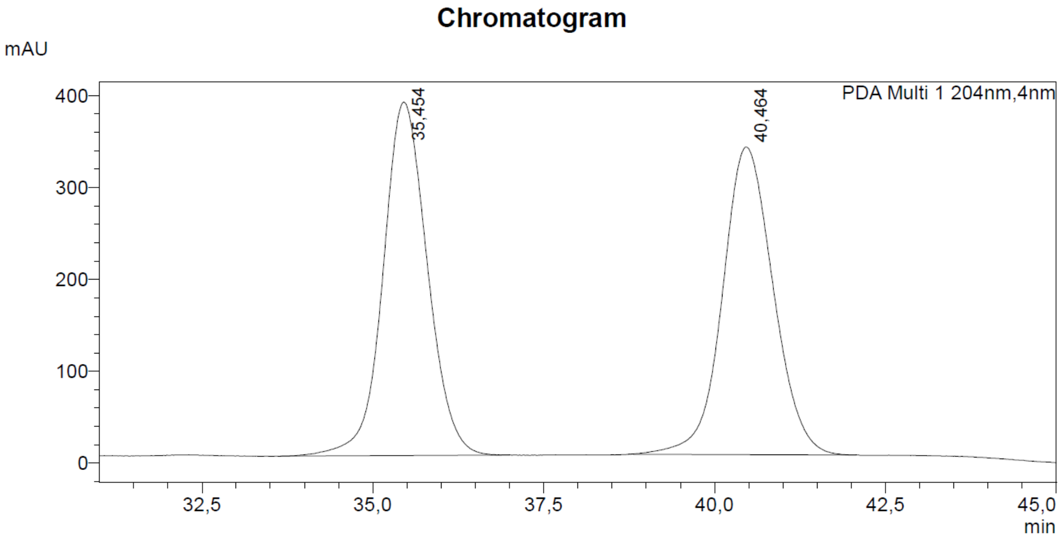

Peak Table

PDA Ch1 204nm

Peak#	Ret. Time	Area	Height	Conc.
1	35,454	17376687	384612	50,040
2	40,464	17348746	334750	49,960
Total		34725433	719362	

Supplementary Figure 57. HPLC spectra of (*R*)-3-(4-Methoxybenzyl)pentanoic acid (2k)

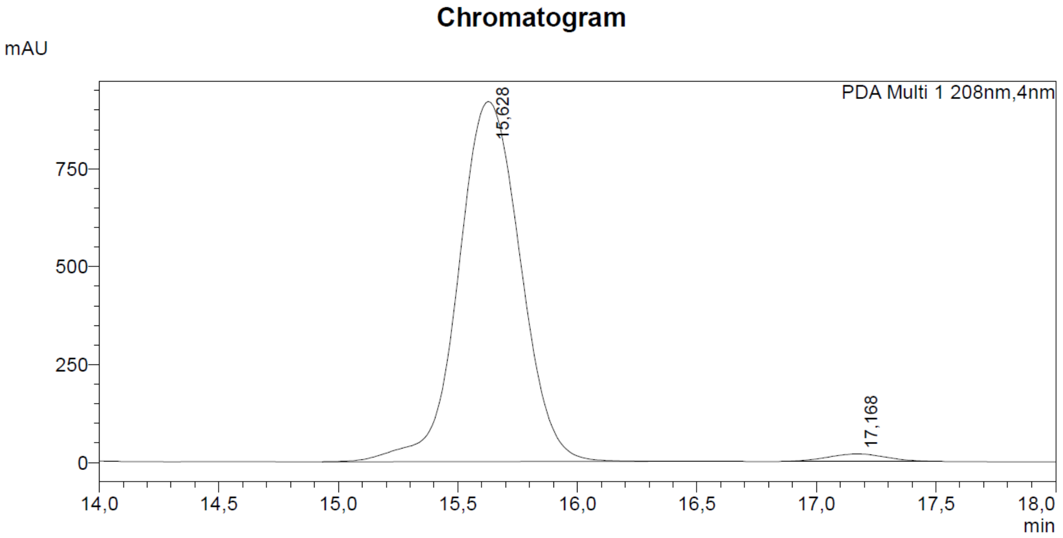

Peak Table

PDA Ch1 208nm

Peak#	Ret. Time	Area	Height	Conc.
1	15,628	16587899	919627	98,126
2	17,168	316867	18866	1,874
Total		16904766	938493	

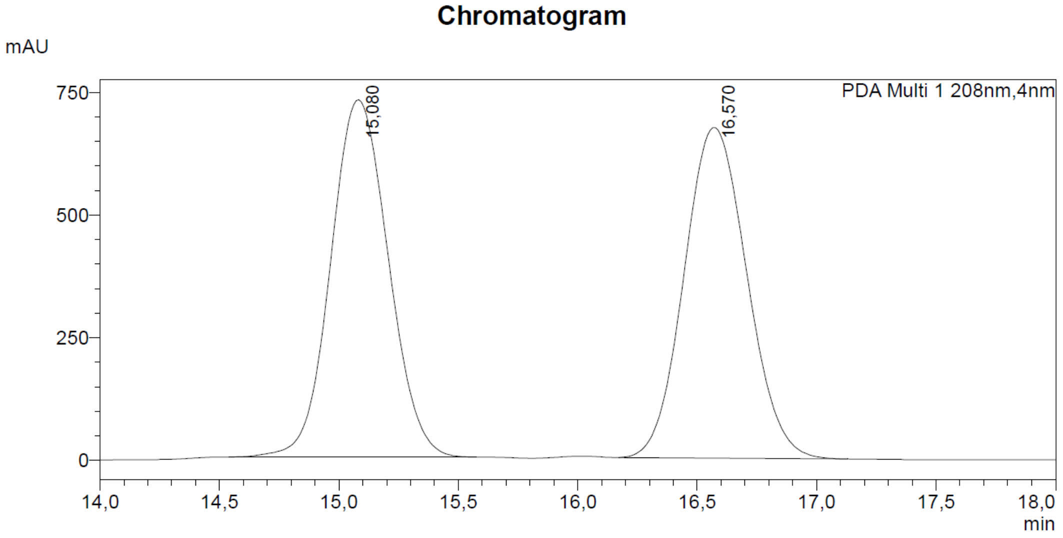

Peak Table

PDA Ch1 208nm

Peak#	Ret. Time	Area	Height	Conc.
1	15,080	12227202	728388	49,808
2	16,570	12321328	674015	50,192
Total		24548530	1402403	

Supplementary Figure 58. HPLC spectra of (*R*)-6-Bromo-3-ethylhexanoic acid (2l)

Chromatogram

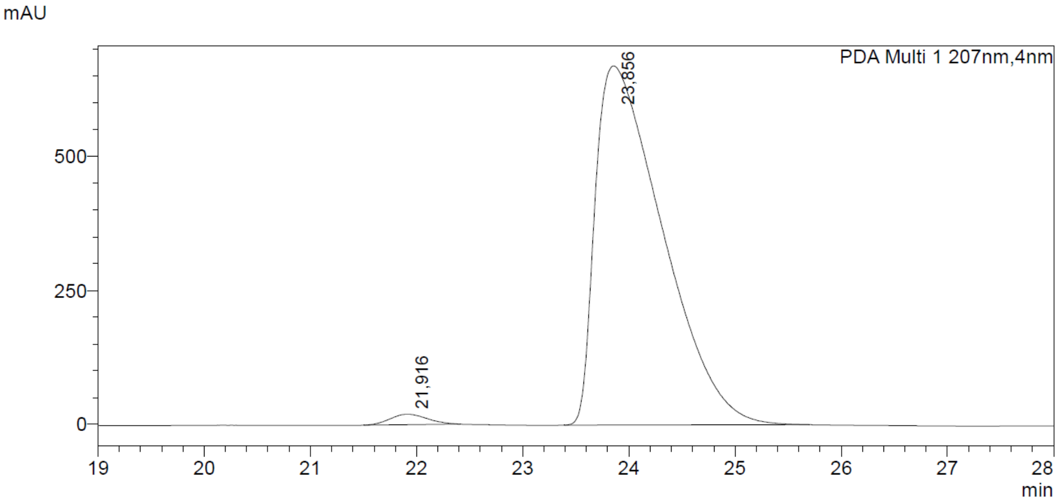

Peak Table

PDA Ch1 207nm

Peak#	Ret. Time	Area	Height	Conc.
1	21,916	475894	19523	1,583
2	23,856	29583722	669893	98,417
Total		30059615	689417	

Chromatogram

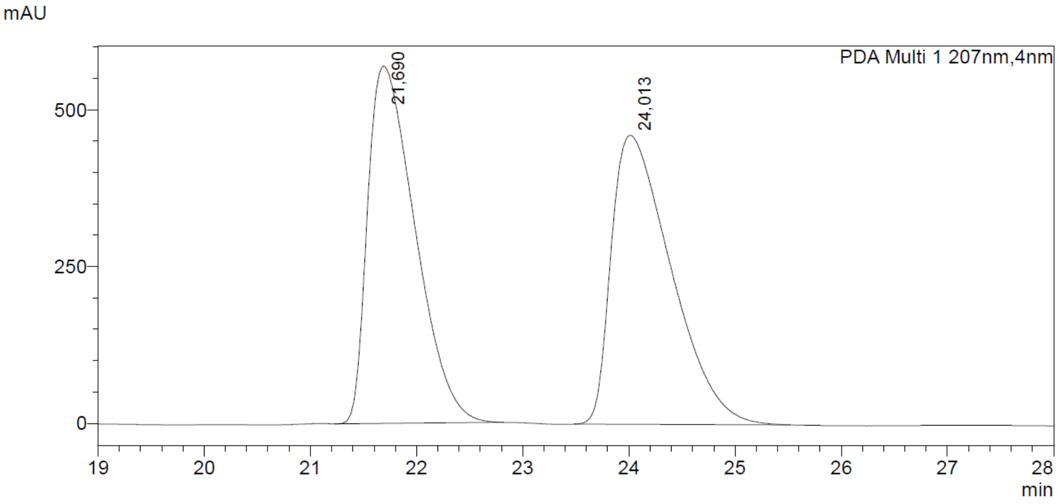

Peak Table

PDA Ch1 207nm

Peak#	Ret. Time	Area	Height	Conc.
1	21,690	17712426	569721	49,510
2	24,013	18062924	460836	50,490
Total		35775349	1030557	

Supplementary Figure 59. HPLC spectra of (*R*)-6-Benzyloxy-3-ethylhexanoic acid (2m)

Chromatogram

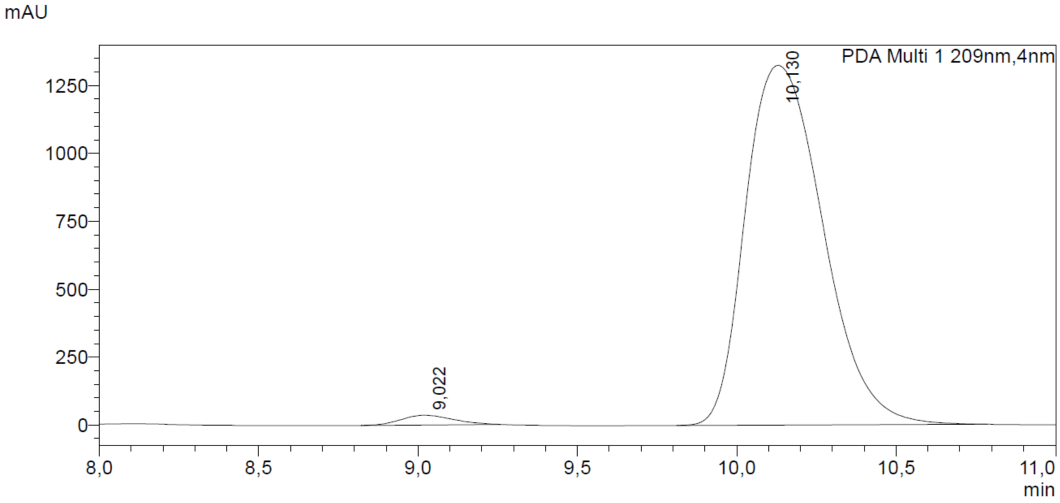

Peak Table

PDA Ch1 209nm

Peak#	Ret. Time	Area	Height	Conc.
1	9,022	407702	35896	1,785
2	10,130	22428761	1324754	98,215
Total		22836463	1360650	

Chromatogram

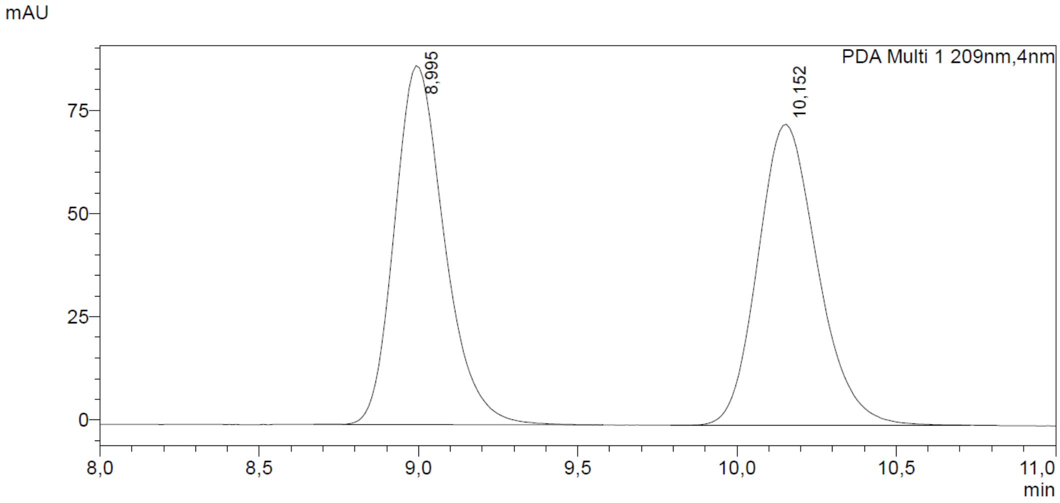

Peak Table

PDA Ch1 209nm

Peak#	Ret. Time	Area	Height	Conc.
1	8,995	950533	86909	49,955
2	10,152	952264	72869	50,045
Total		1902798	159778	

Supplementary Figure 60. HPLC spectra of (*R*)-3-Methylhexanoic acid (3a)

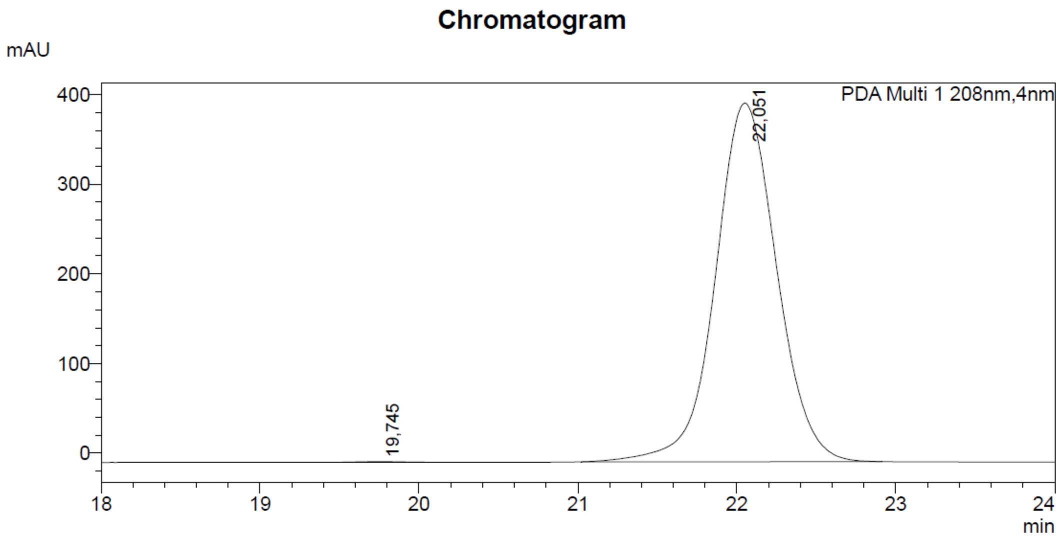

Peak Table

PDA Ch1 208nm

Peak#	Ret. Time	Area	Height	Conc.
1	19,745	18052	843	0,168
2	22,051	10745514	399869	99,832
Total		10763567	400712	

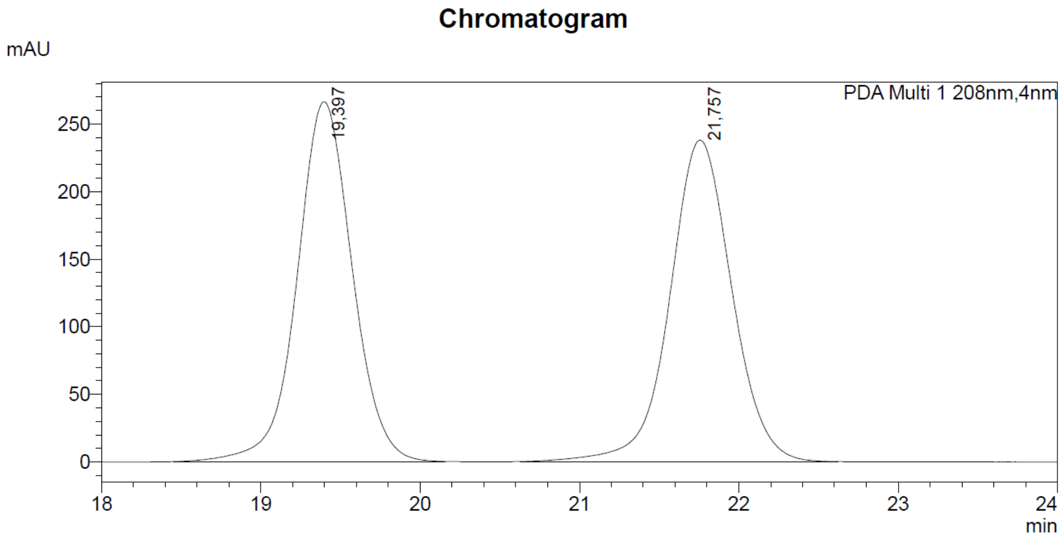

Peak Table

PDA Ch1 208nm

Peak#	Ret. Time	Area	Height	Conc.
1	19,397	6393816	265935	49,848
2	21,757	6432839	237805	50,152
Total		12826655	503739	

Supplementary Figure 61. HPLC spectra of (*R*)-3-Phenylbutanoic acid (3b)

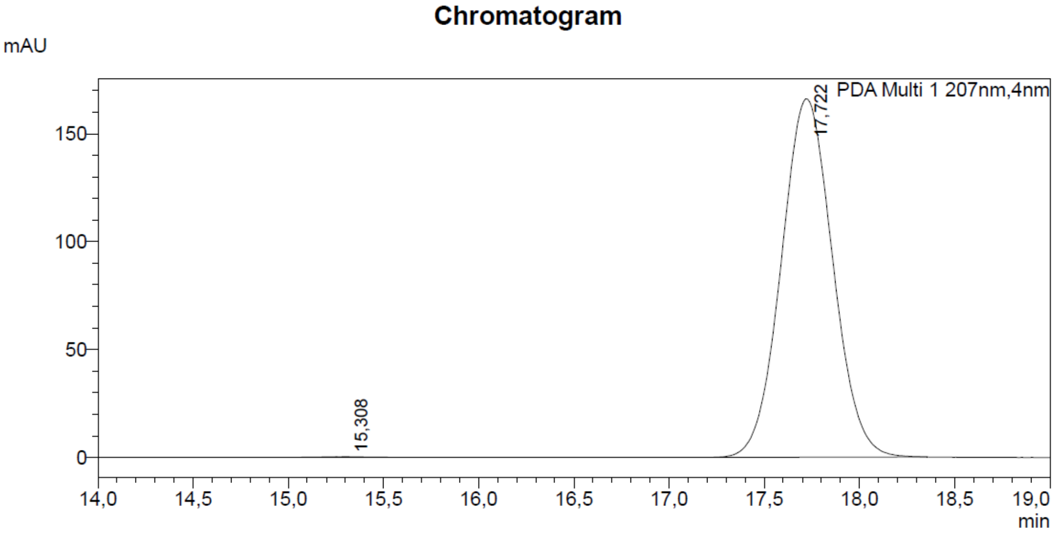

Peak Table

PDA Ch1 207nm

Peak#	Ret. Time	Area	Height	Conc.
1	15,308	4876	294	0,156
2	17,722	3122820	166071	99,844
Total		3127697	166365	

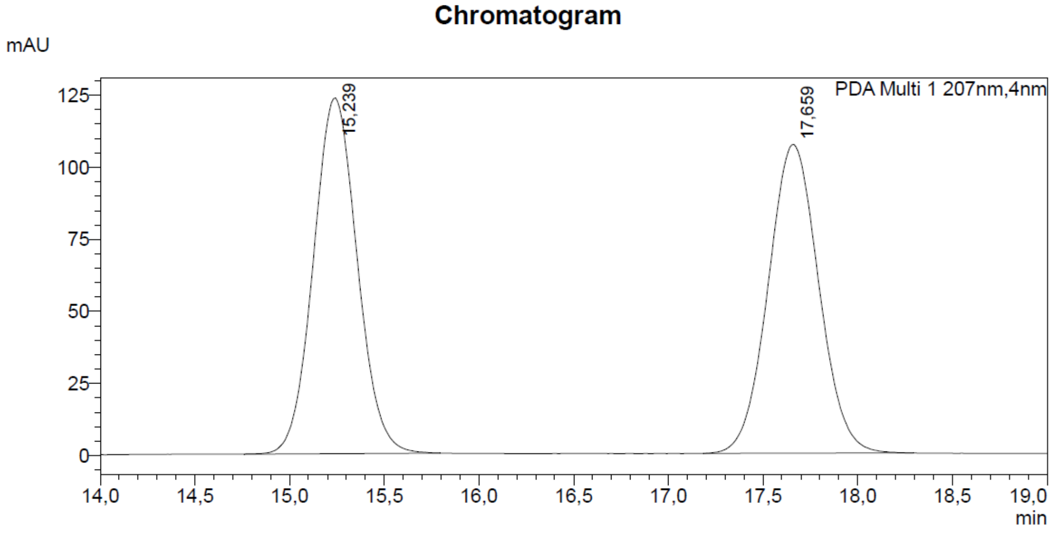

Peak Table

PDA Ch1 207nm

Peak#	Ret. Time	Area	Height	Conc.
1	15,239	1987280	123314	49,985
2	17,659	1988490	107025	50,015
Total		3975771	230340	

Supplementary Figure 62. HPLC spectra of (*R*)-3-(4-Methylphenyl)butanoic acid (3c)

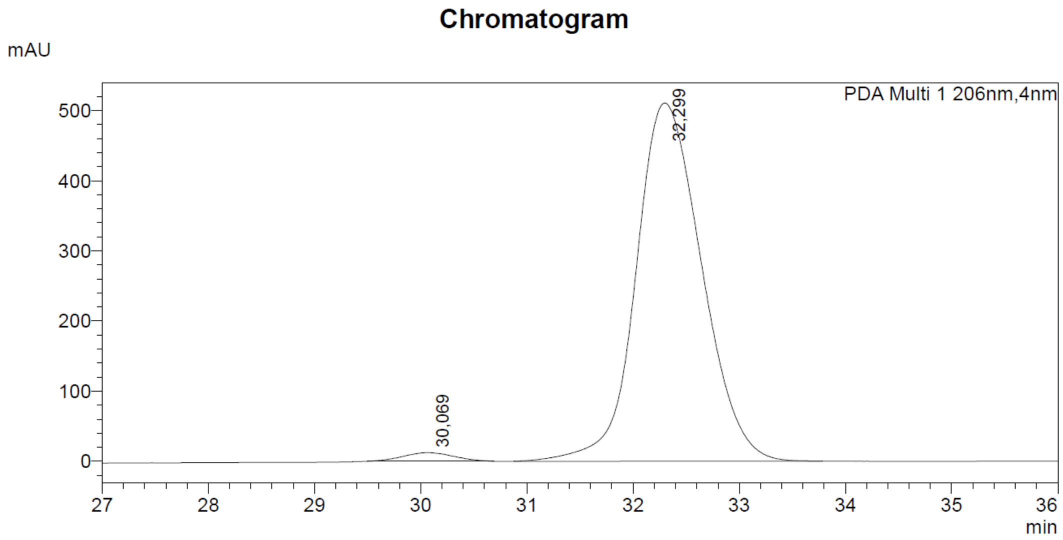

Peak Table

PDA Ch1 206nm

Peak#	Ret. Time	Area	Height	Conc.
1	30,069	406825	12247	1,821
2	32,299	21930234	510376	98,179
Total		22337059	522623	

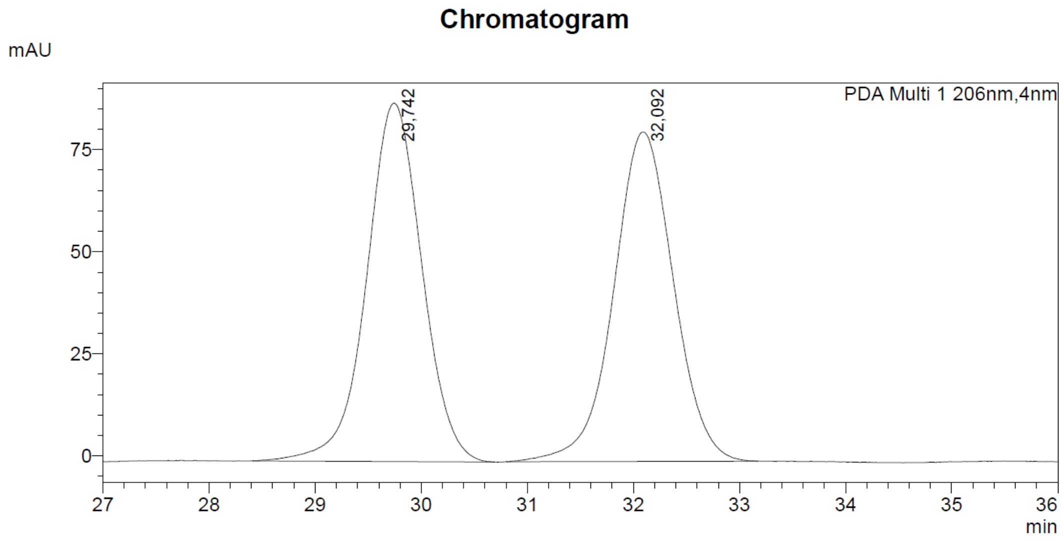

Peak Table

PDA Ch1 206nm

Peak#	Ret. Time	Area	Height	Conc.
1	29,742	3183843	87789	50,107
2	32,092	3170301	80666	49,893
Total		6354144	168455	

Supplementary Figure 63. HPLC spectra of (*R*)-3-Cyclopentylhexanoic acid (3d)

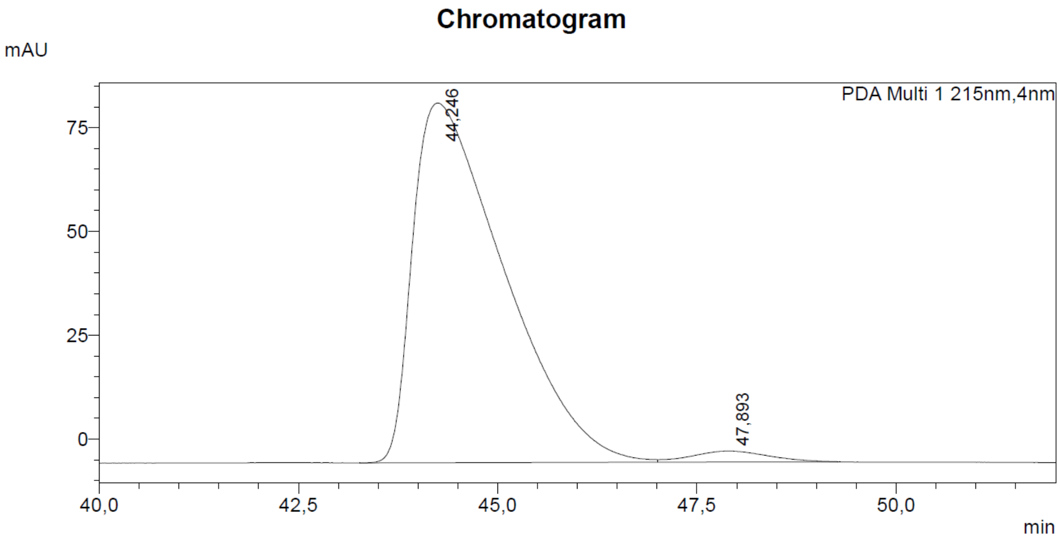

Peak Table

PDA Ch1 215nm

Peak#	Ret. Time	Area	Height	Conc.
1	44,246	6896487	86593	97,511
2	47,893	176012	2635	2,489
Total		7072499	89228	

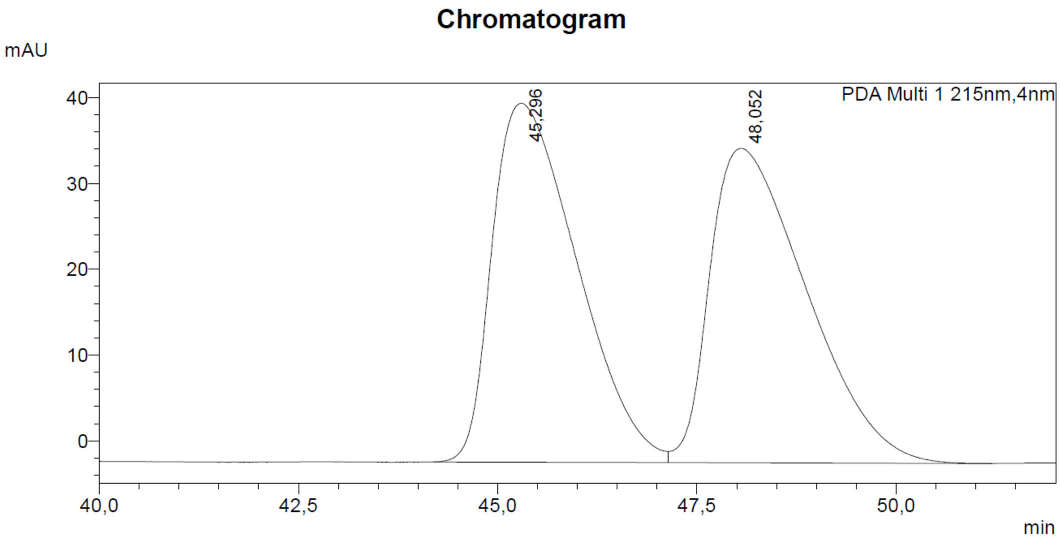

Peak Table

PDA Ch1 215nm

Peak#	Ret. Time	Area	Height	Conc.
1	45,296	3093985	41843	49,979
2	48,052	3096525	36664	50,021
Total		6190509	78507	

Supplementary Figure 64. HPLC spectra of (*S*)-5-Methyl-3-propylhexanoic acid (3e)

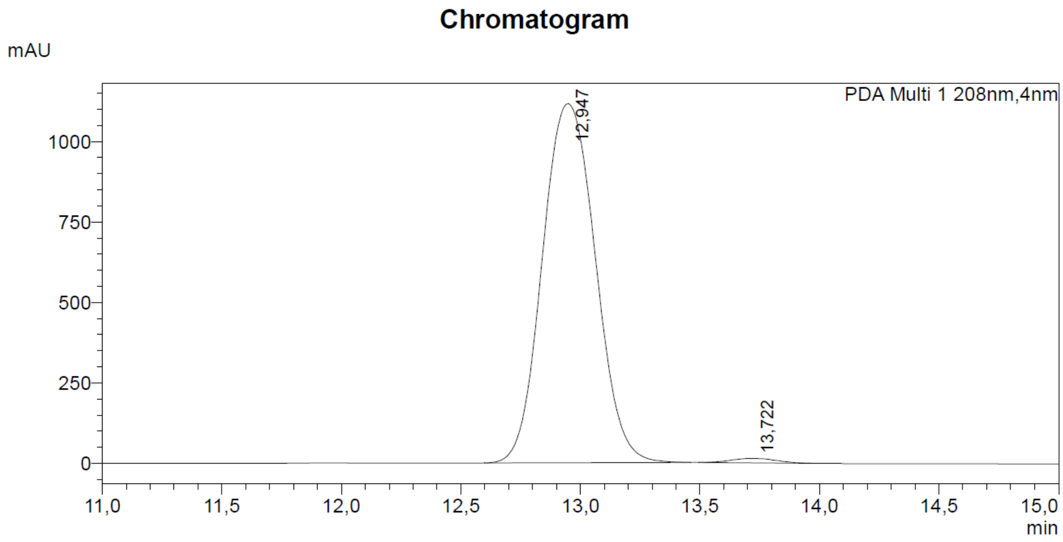

Peak Table

PDA Ch1 208nm

Peak#	Ret. Time	Area	Height	Conc.
1	12,947	17175110	1115729	98,932
2	13,722	185386	14069	1,068
Total		17360495	1129798	

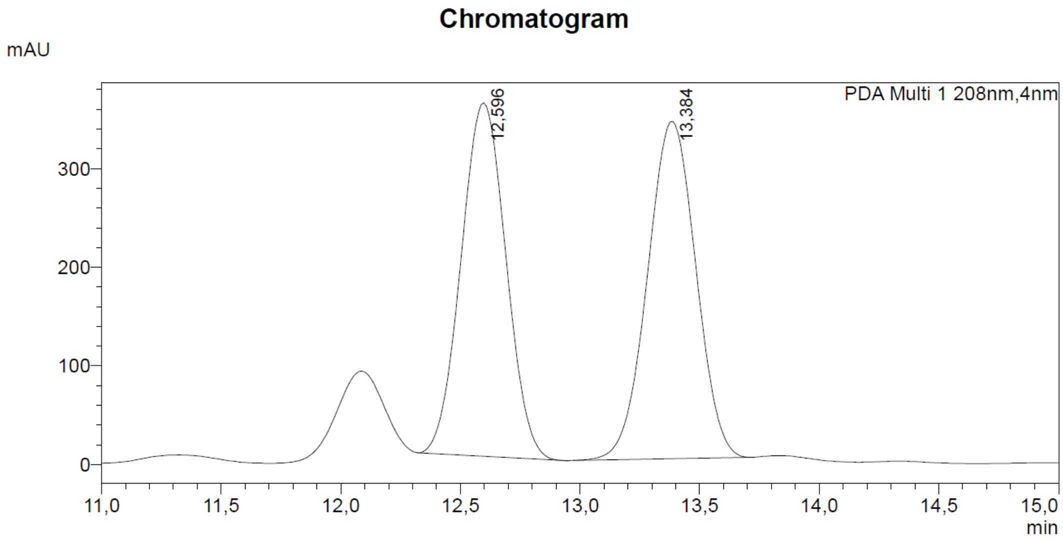

Peak Table

PDA Ch1 208nm

Peak#	Ret. Time	Area	Height	Conc.
1	12,596	4664613	358074	49,250
2	13,384	4806597	342072	50,750
Total		9471210	700146	

Supplementary Figure 65. HPLC spectra of (*S*)-6-Methyl-3-propylheptanoic acid (3f)

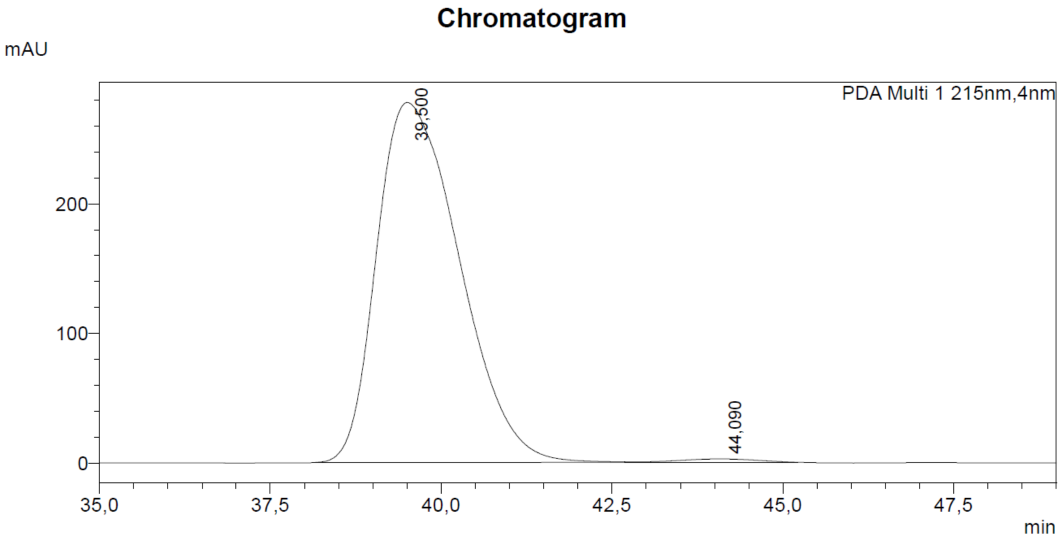

Peak Table

PDA Ch1 215nm

Peak#	Ret. Time	Area	Height	Conc.
1	39,500	23423708	277747	99,101
2	44,090	212381	2672	0,899
Total		23636089	280419	

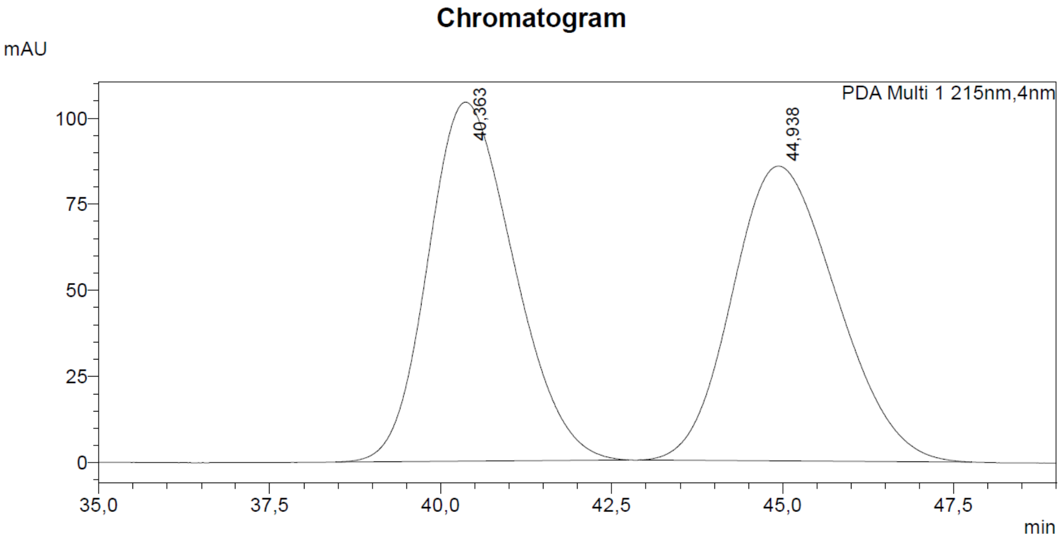

Peak Table

PDA Ch1 215nm

Peak#	Ret. Time	Area	Height	Conc.
1	40,363	9103386	104186	50,020
2	44,938	9096107	85552	49,980
Total		18199493	189738	

Supplementary Figure 66. HPLC spectra of (*S*)-3-Propyl-oct-7-enoic acid (3g)

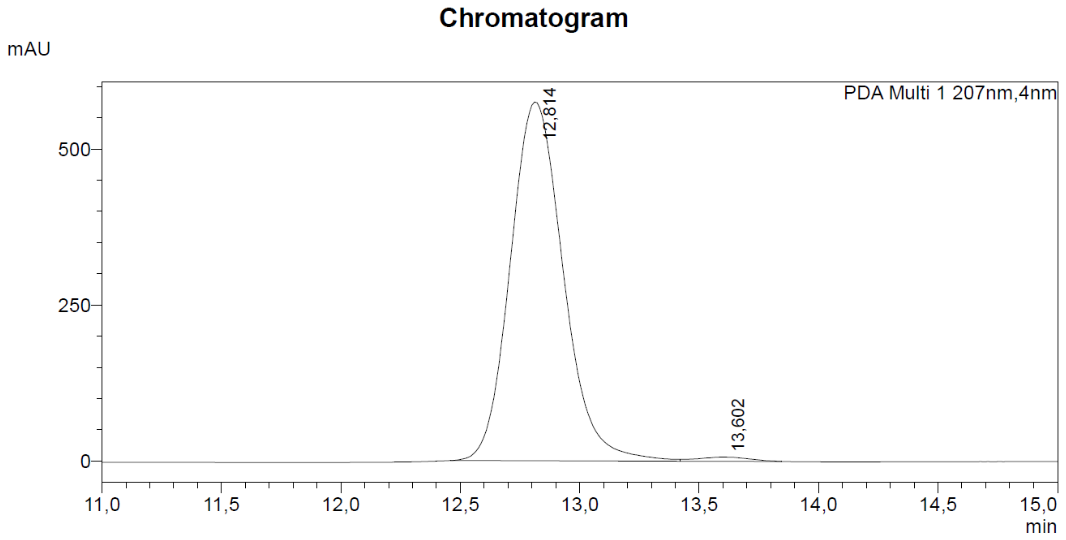

Peak Table

PDA Ch1 207nm

Peak#	Ret. Time	Area	Height	Conc.
1	12,814	8854419	574479	98,897
2	13,602	98719	6771	1,103
Total		8953138	581250	

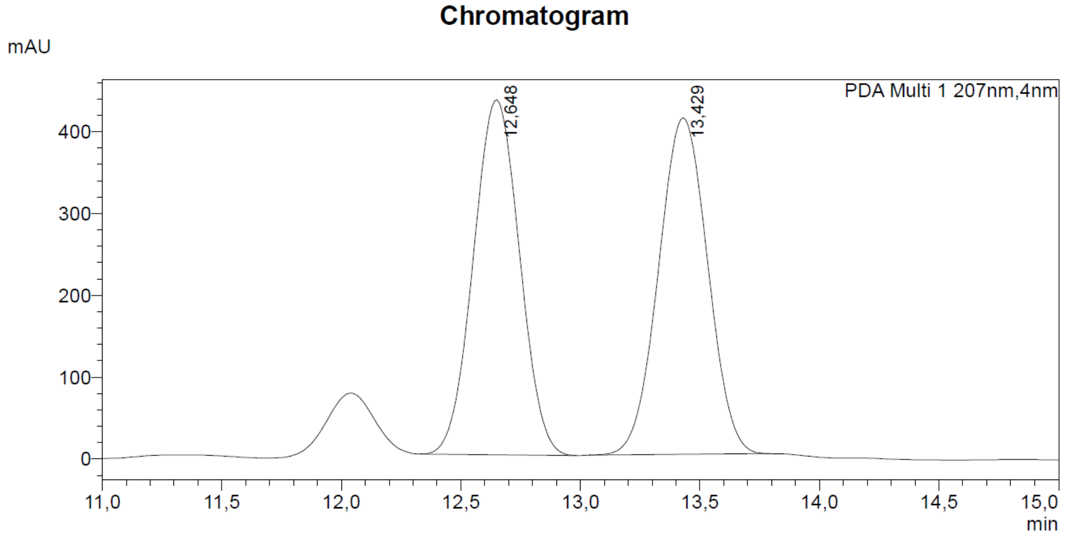

Peak Table

PDA Ch1 207nm

Peak#	Ret. Time	Area	Height	Conc.
1	12,648	5834788	433674	49,807
2	13,429	5880066	411237	50,193
Total		11714855	844911	

Supplementary Figure 67. HPLC spectra of (*S*)-3-Propylnonanoic acid (3h)

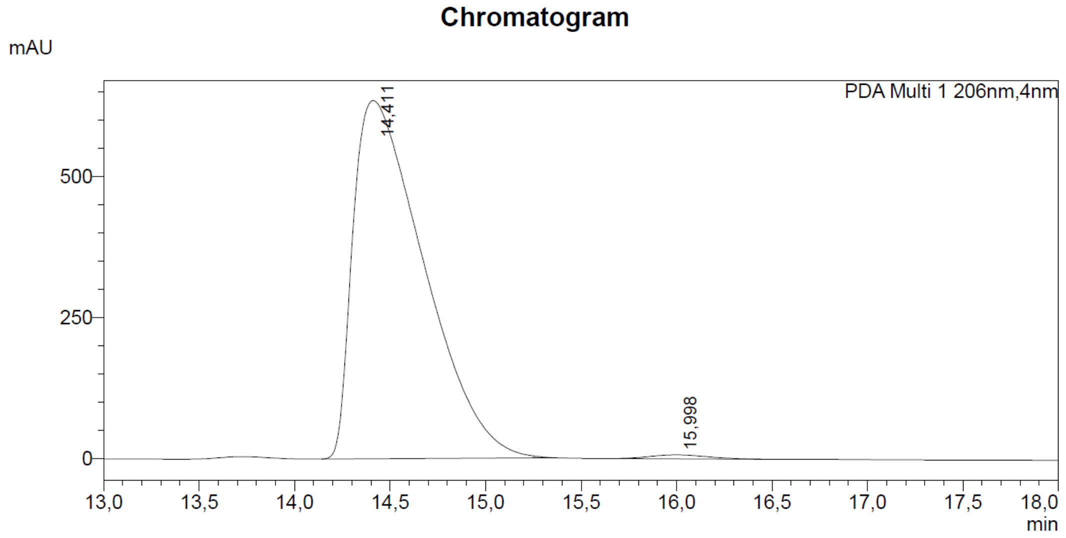

Peak Table

PDA Ch1 206nm

Peak#	Ret. Time	Area	Height	Conc.
1	14,411	16426336	635145	99,151
2	15,998	140733	6831	0,849
Total		16567068	641977	

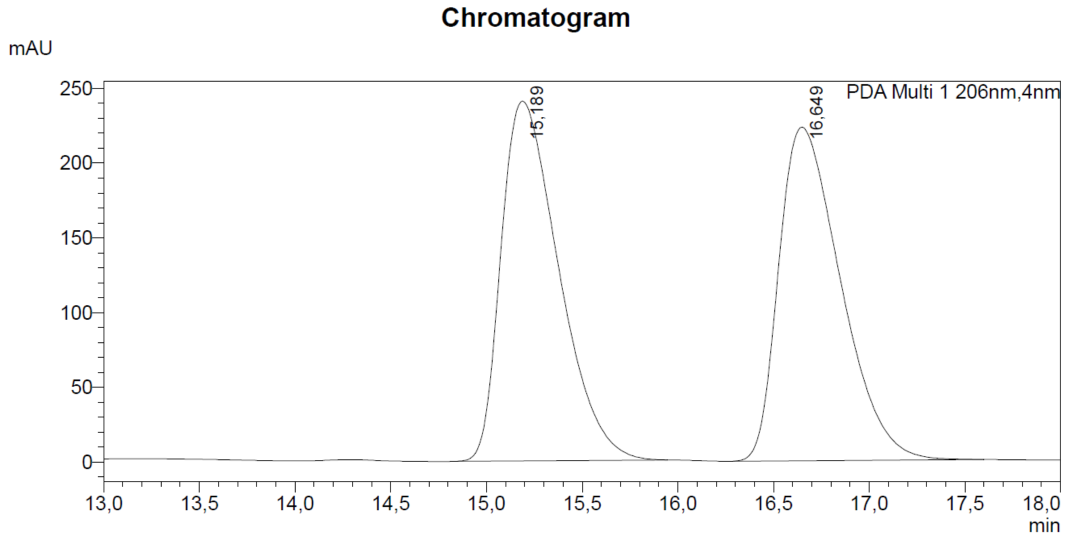

Peak Table

PDA Ch1 206nm

Peak#	Ret. Time	Area	Height	Conc.
1	15,189	5035670	240895	50,113
2	16,649	5012931	223358	49,887
Total		10048601	464253	

Supplementary Figure 68. HPLC spectra of (*S*)-3-Methylnonanoic acid (3i)

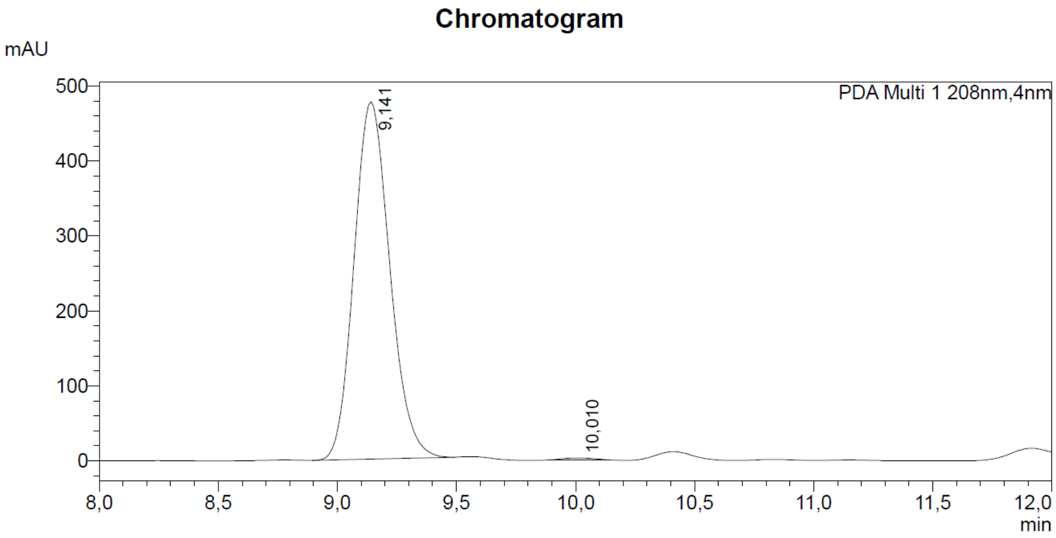

Peak Table

PDA Ch1 208nm

Peak#	Ret. Time	Area	Height	Conc.
1	9,141	4983529	476943	99,555
2	10,010	22280	2577	0,445
Total		5005809	479520	

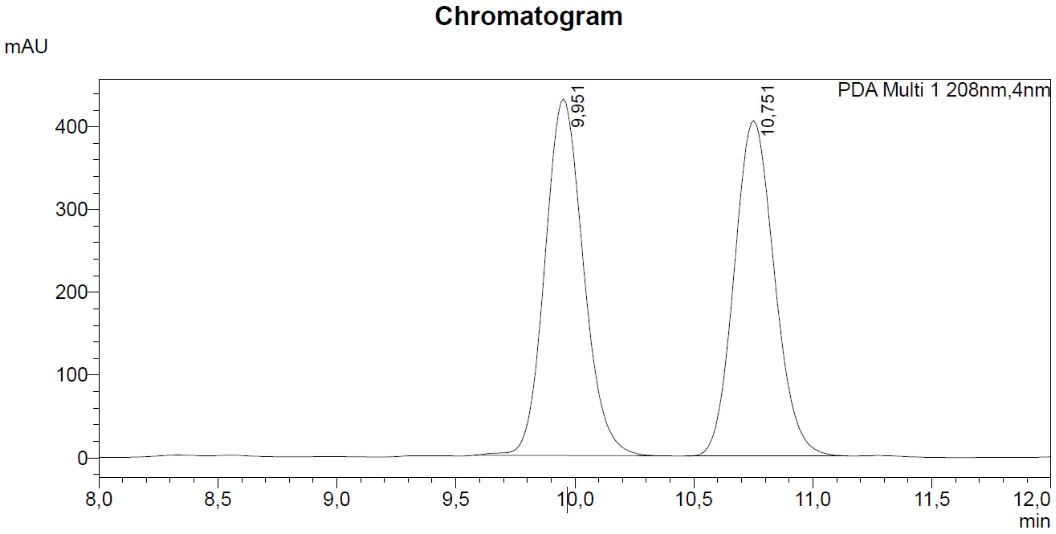

Peak Table

PDA Ch1 208nm

Peak#	Ret. Time	Area	Height	Conc.
1	9,951	4844582	430981	50,416
2	10,751	4764569	405583	49,584
Total		9609151	836565	

Supplementary Figure 69. HPLC spectra of (*R*)-(2-Bromo-1-methylethyl)benzene (4c)

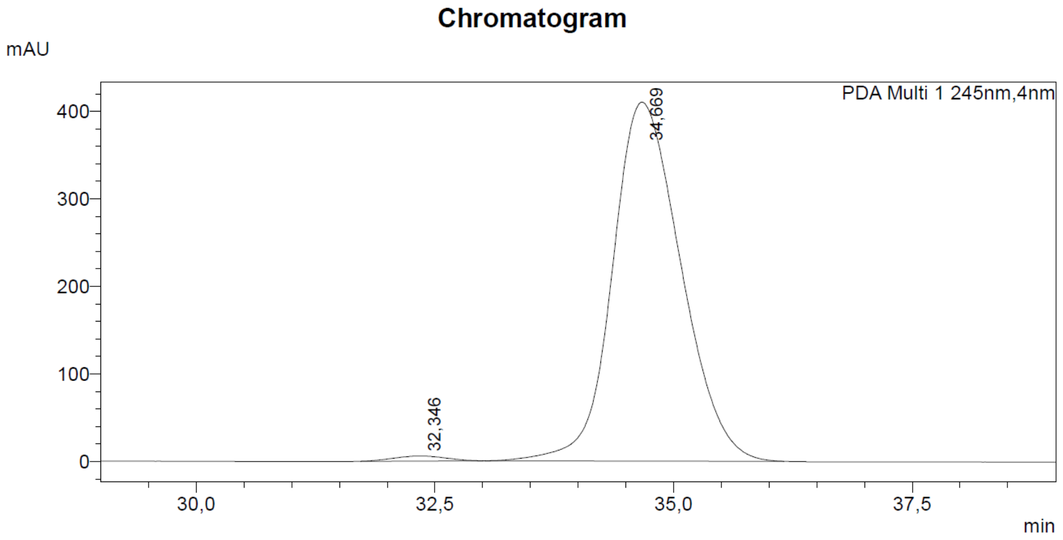

Peak Table

PDA Ch1 245nm

Peak#	Ret. Time	Area	Height	Conc.
1	32,346	214620	5735	1,037
2	34,669	20489544	409965	98,963
Total		20704164	415700	

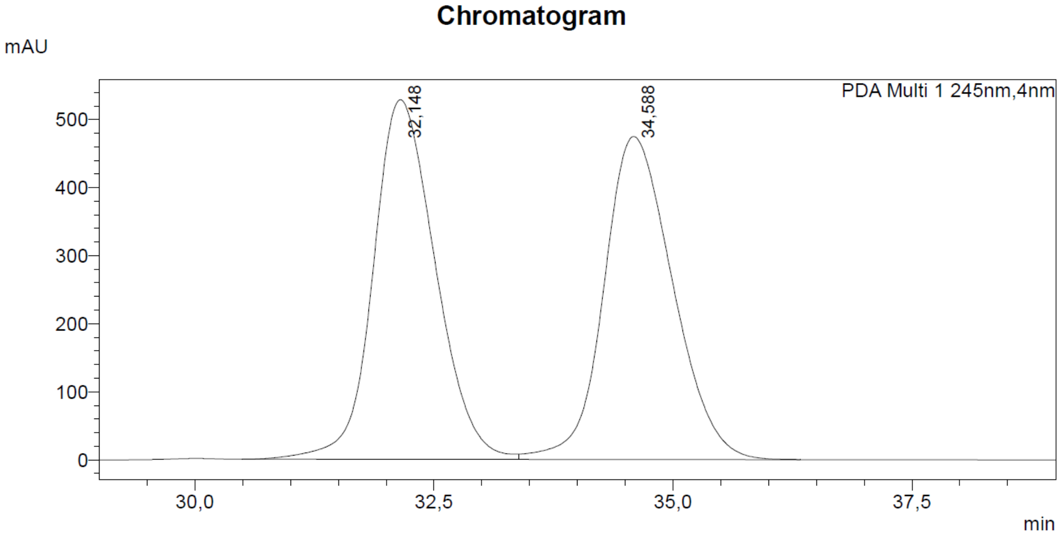

Peak Table

PDA Ch1 245nm

Peak#	Ret. Time	Area	Height	Conc.
1	32,148	23972280	528352	50,147
2	34,588	23831434	474482	49,853
Total		47803714	1002834	

Supplementary Figure 70. HPLC spectra of (*S*)-1-(2-Propyl)octyl-4-phenyl-1*H*-1,2,3-triazole (4e)

Chromatogram

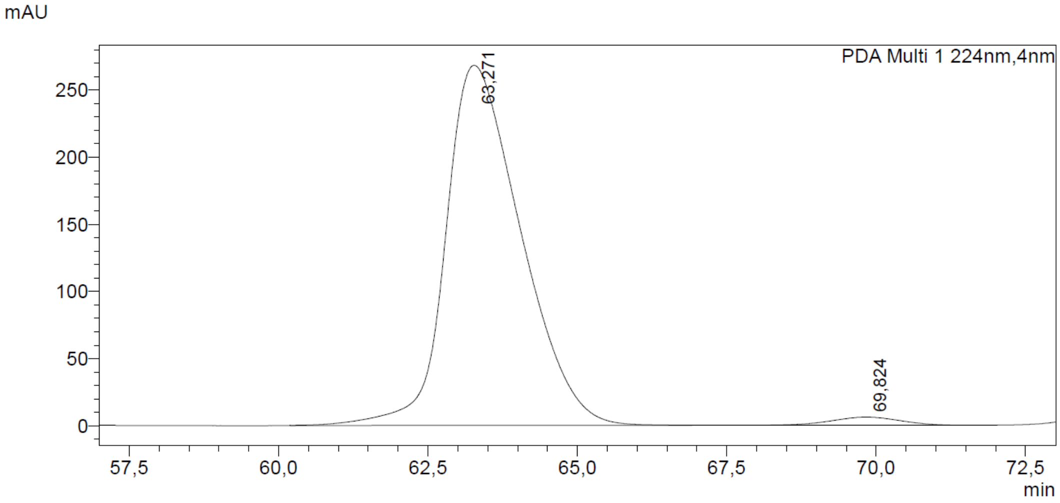

Peak Table

PDA Ch1 224nm

Peak#	Ret. Time	Area	Height	Conc.
1	63,271	23488782	268092	97,946
2	69,824	492472	6103	2,054
Total		23981253	274195	

Chromatogram

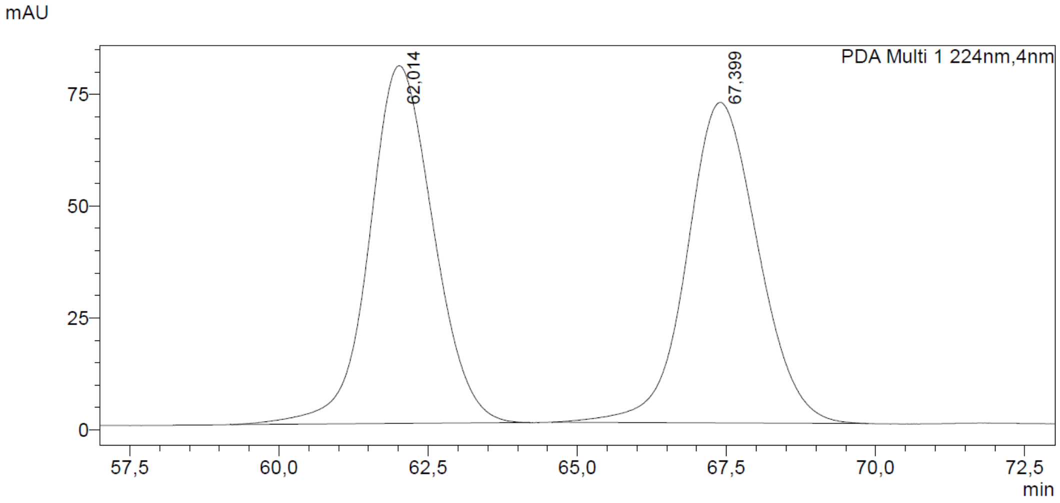

Peak Table

PDA Ch1 224nm

Peak#	Ret. Time	Area	Height	Conc.
1	62,014	5995266	79959	50,101
2	67,399	5970995	71600	49,899
Total		11966262	151559	

Supplementary Figure 71. HPLC spectra of (*S*)-4-(2-Ethylpentyl)phenol (4f)

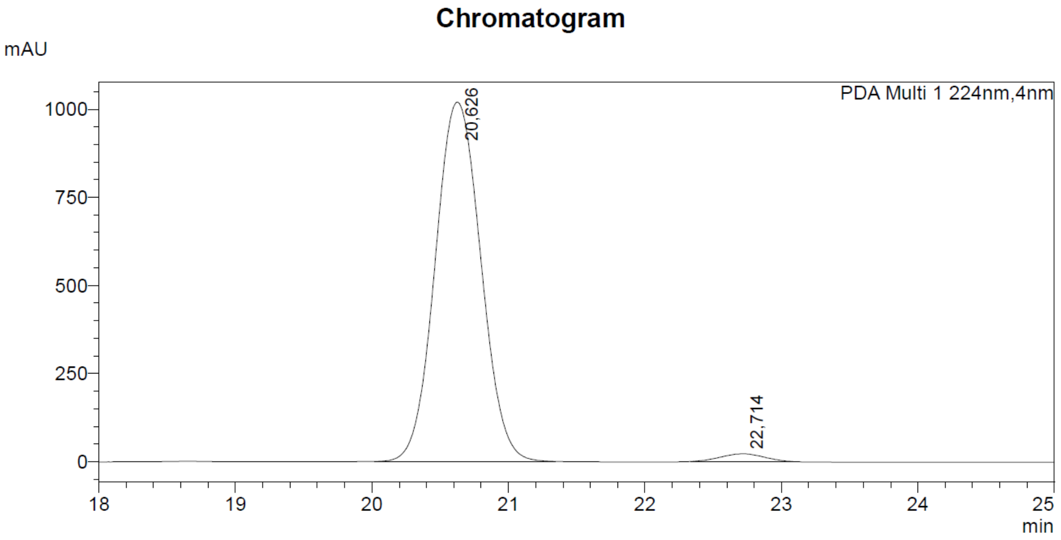

Peak Table

PDA Ch1 224nm

Peak#	Ret. Time	Area	Height	Conc.
1	20,626	23718719	1019381	98,035
2	22,714	475395	21733	1,965
Total		24194114	1041113	

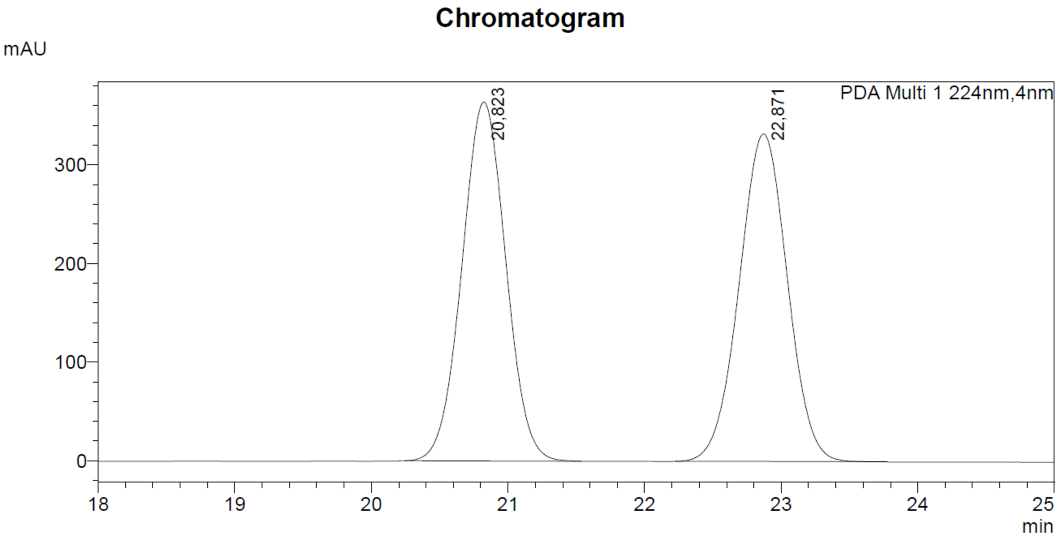

Peak Table

PDA Ch1 224nm

Peak#	Ret. Time	Area	Height	Conc.
1	20,823	8129329	363780	49,953
2	22,871	8144600	332038	50,047
Total		16273929	695818	

Supplementary Figure 72. HPLC spectra of (*R*)-2-(4-Methoxybenzyl)butan-1-ol (4g)

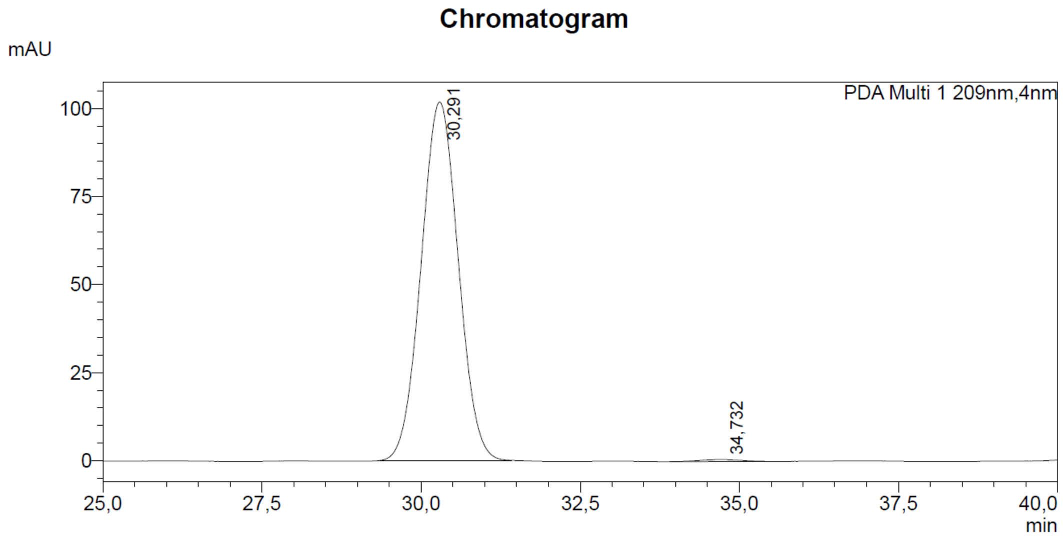

Peak Table

PDA Ch1 209nm

Peak#	Ret. Time	Area	Height	Conc.
1	30,291	4132695	101836	99,440
2	34,732	23264	537	0,560
Total		4155960	102374	

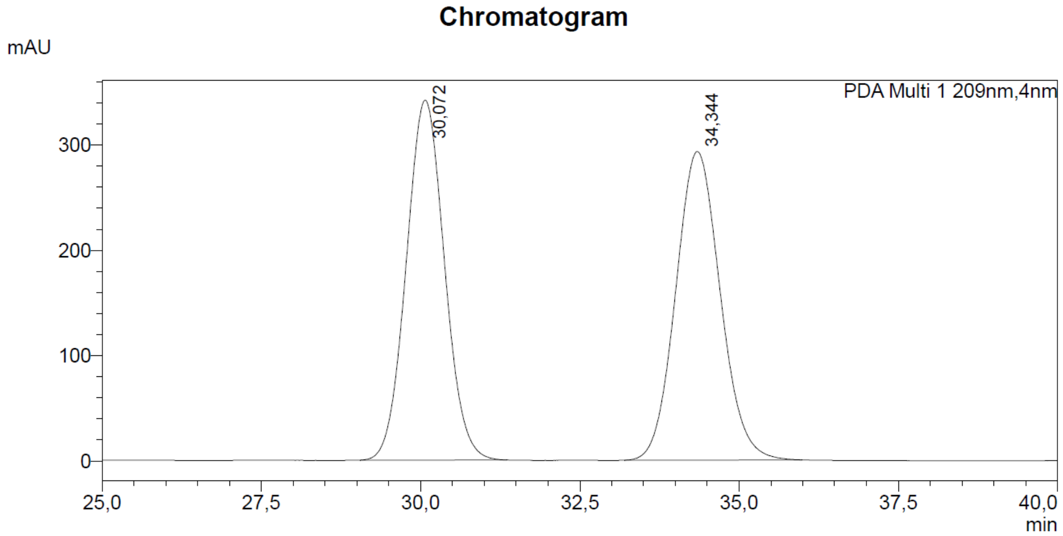

Peak Table

PDA Ch1 209nm

Peak#	Ret. Time	Area	Height	Conc.
1	30,072	14259050	341801	50,040
2	34,344	14236207	293063	49,960
Total		28495257	634863	

Supplementary Figure 73. HPLC spectra of (*R*)-3-(4-hydroxy-3-(5,6,7,8-tetrahydro-5,5,8,8-tetramethyl-3-pentyloxy-2-naphthalenyl)phenyl) butanoic acid (5b)

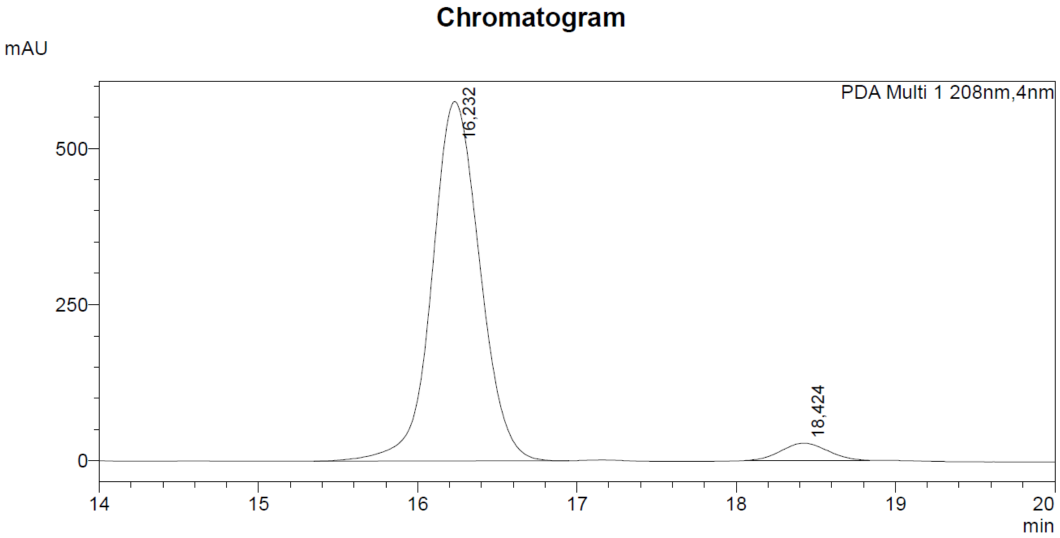

Peak Table

PDA Ch1 208nm

Peak#	Ret. Time	Area	Height	Conc.
1	16,232	12020503	575129	95,585
2	18,424	555208	27516	4,415
Total		12575711	602645	

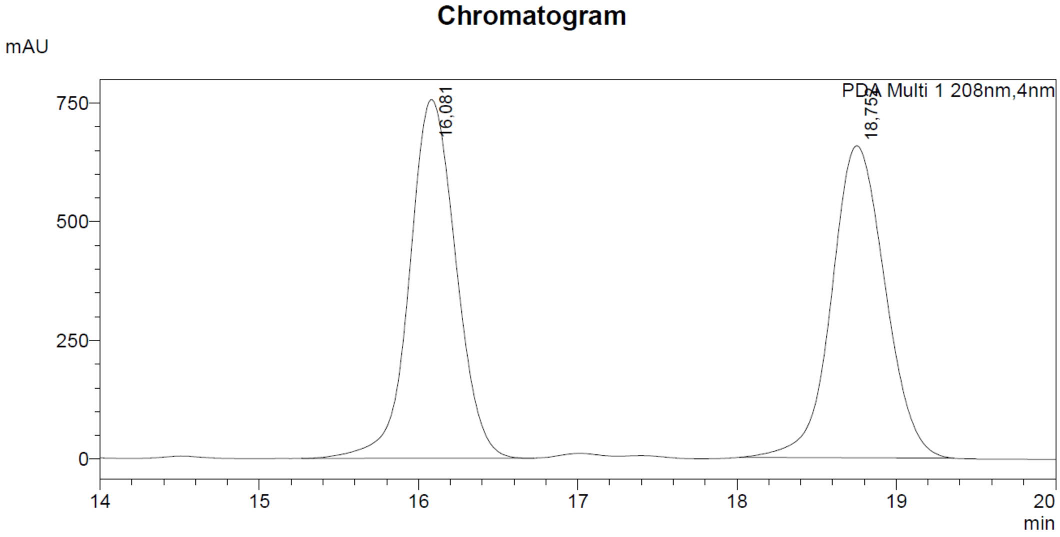

Peak Table

PDA Ch1 208nm

Peak#	Ret. Time	Area	Height	Conc.
1	16,081	15330706	754836	49,969
2	18,752	15349591	656953	50,031
Total		30680297	1411789	

Supplementary Figure 74. HPLC spectra of (1*R*,2*R*)-2-Ethylcyclopentane-1-carboxylic acid (6)

Chromatogram

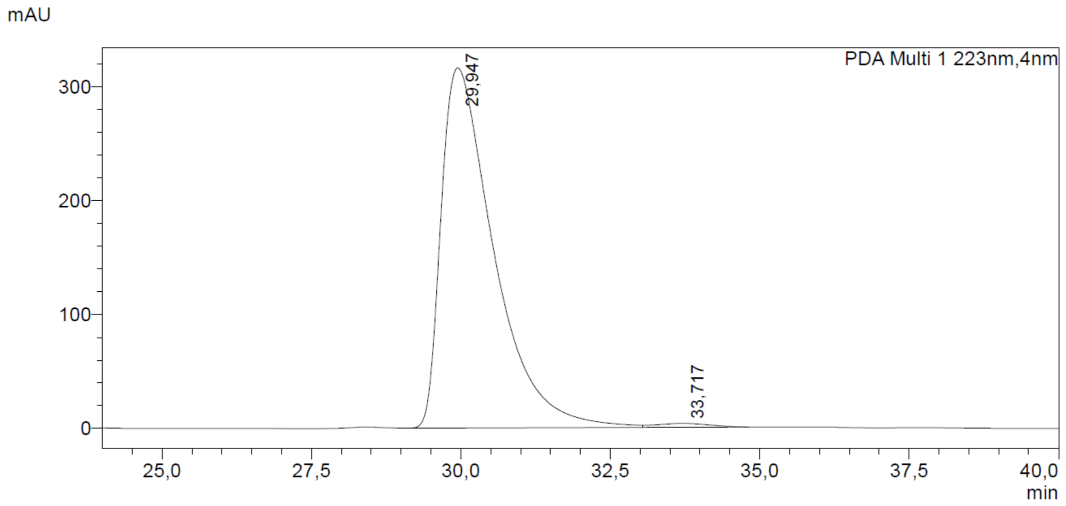

Peak Table

PDA Ch1 223nm

Peak#	Ret. Time	Area	Height	Conc.
1	29,947	19423784	316323	98,930
2	33,717	210142	3396	1,070
Total		19633926	319719	

Chromatogram

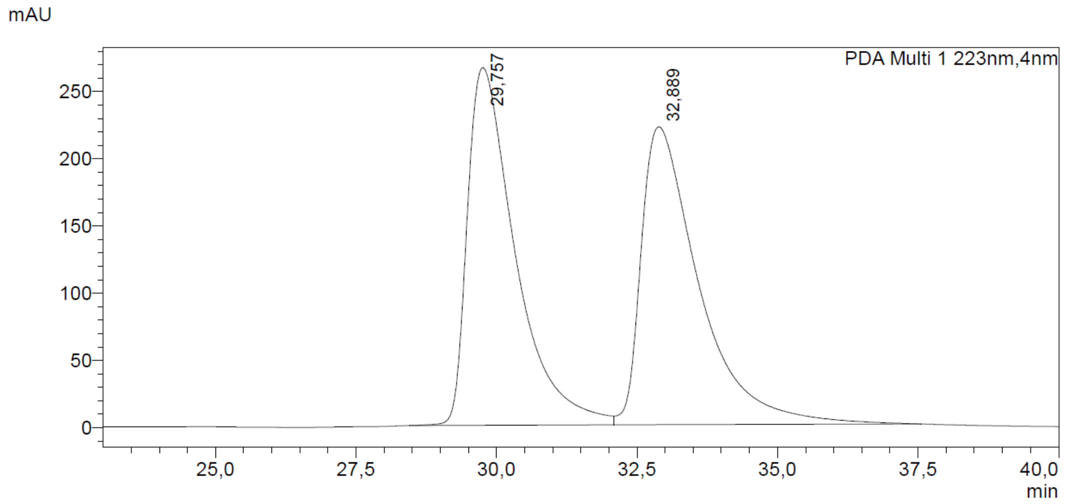

Peak Table

PDA Ch1 223nm

Peak#	Ret. Time	Area	Height	Conc.
1	29,757	15787721	266010	49,815
2	32,889	15905134	221652	50,185
Total		31692855	487662	

Supplementary Figure 75. HPLC spectra of (*S*)-Ethyl 6-chloro-3-(3-methylnonanoyl)-1*H*-indole-2-carboxylate (7b)

Supplementary References

1. Rodríguez-Fernández, M., Yan, X., Collados, J. F., White, P. B. & Harutyunyan, S. R. Lewis acid enabled copper-catalyzed asymmetric synthesis of chiral β-substituted amides. *J. Am. Chem. Soc.* **139**, 14224–14231 (2017).
2. Broggi, J., Urbina-Blanco, C. A., Clavier, H., Leitgeb, A., Slugovc, C., Slawin, A. M. Z. & Nolan, S. P. The influence of phosphane ligands on the versatility of Ruthenium-indenylidene complexes in metathesis. *Chem. Eur. J.* **16**, 9215–9225 (2010).
3. Concellón, J. M. & Concellón, C. Direct reaction of dibromoacetic acid with aldehydes promoted by Samarium diiodide: an easy, efficient, and rapid synthesis of (*E*)-α,β-unsaturated carboxylic acids with total stereoselectivity. *J. Org. Chem.* **71**, 1728–1731 (2006).
4. Hird, A. W. & Hoveyda, A. H. Cu-catalyzed enantioselective conjugate additions of alkyl zinc reagents to unsaturated N-acyloxazolidinones promoted by a chiral triamide phosphane. *Angew. Chem. Int. Ed.* **42**, 1276–1279 (2003).
5. García, J. M., González, A., Kardak, B. G., Odriozola, J. M., Oiarbide, M., Razkin, J. & Palomo, C. Copper-catalyzed enantioselective conjugate addition of dialkylzinc reagents to α'-oxy enones. *Chem. Eur. J.* **14**, 8768–8771 (2008).
6. Deasy, R. E., Brossat, M., Moody, T. S. & Maguire, A. R. Lipase catalysed kinetic resolutions of 3-aryl alkanoic acids. *Tetrahedron: Asymmetry* **22**, 47–61 (2011).
7. Derosa, J., Tran, V. T., Boulous, M. N., Chen, J. S. & Engle, K. M. Nickel-catalyzed β,γ-dicarbofunctionalization of alkenyl carbonyl compounds via conjunctive cross-coupling. *J. Am. Chem. Soc.* **139**, 10657–10660 (2017).
8. Norsikian, S., Marek, I., Klein, S., Poisson, J. F. & Normant, J. F. Enantioselective carbometalation of cinnamyl derivatives: new access to chiral disubstituted cyclopropanes-configurational stability of benzylic organozinc halides. *Chem. Eur. J.* **5**, 2055–2068 (1999).
9. Yuan, P., Chen, J., Zhao, J., & Huang, Y. Enantioselective hydroamidation of enals by trapping of a transient acyl species. *Angew. Chem. Int. Ed.* **57**, 8503–8507 (2018).
10. Chen, B., Cao, P., Liao, Y., Wang, M. & Liao, J. Enantioselective copper-catalyzed methylboration of alkenes. *Org. Lett.* **20**, 1346–1349 (2018).
11. Qin, T., Cornella, J., Li, C., Malins, L. R., Edwards, J. T., Kawamura, S., Maxwell, B. D., Eastgate, M. D. & Baran, P. S. A general alkyl-alkyl cross-coupling enabled by redox-active esters and alkylzinc reagents. *Science* **352**, 801–805 (2016).
12. Li, C., Wang, J., Barton, L. M., Yu, S., Tian, M., Peters, D. S., Kumar, M., Yu, A. W., Johnson, K. A., Chatterjee, A. K., Yan, M. & Baran, P. S. Decarboxylative borylation. *Science* **356**, 1045 (2017).
13. Tamao K., Yoshida, J., Akita, M., Sugihara, Y., Iwahara, T. & Kumada, M. Organofluorosilicates in organic synthesis. XVI. synthesis of organopentafluorosilicates via the Diels-Alder, ene, and Friedel-Crafts reaction. Their transformations to organic halides and alcohols. *Bull. Chem. Soc. Jpn.* **55**, 255–260 (1982).
14. Ozawa, J. & Kanai, M. Silver-catalyzed C(sp^3)–H chlorination. *Org. Lett.* **19**, 1430–1433 (2017).
15. Tan, X., Song, T., Wang, Z., Chen, H., Cui, L. & Li, C. Silver-catalyzed decarboxylative bromination of aliphatic carboxylic acids. *Org. Lett.* **19**, 1634–1637 (2017)

16. Raushel, J., Pitram, S. M. & Fokin, V. V. Efficient synthesis of sulfonyl azides from sulfonamides. *Org. Lett.* **10**, 3385–3388 (2008).
17. Xing, Y., Cheng, B., Wang, J., Lu, P. & Wang, Y. Copper-catalyzed three-component synthesis of 3-aminopyrazoles and 4-iminopyrimidines via β-alkynyl-N-sulfonyl ketenimine intermediates. *Org. Lett.* **16**, 4814–4817 (2014).
18. Zhu, Y., Li, X., Wang, X., Huang, X., Shen, T., Zhang, Y., Sun, X., Zou, M., Song, S. & Jiao, N. Silver-catalyzed decarboxylative azidation of aliphatic carboxylic acids. *Org. Lett.* **17**, 4702–4705 (2015).
19. Lu, X., Ge, L., Cheng, C., Chen, J., Cao, W. & Wu, X. Enantioselective cascade reaction for synthesis of quinolinones via synergistic catalysis using Cu-Pybox and chiral benzotetramisole as catalysts. *Chem. Eur. J.* **23**, 7689–7693 (2017).
20. Waghmode, S. B., Mahale, G., Patil, V. P., Renalson, K. & Singh, D. Efficient method for demethylation of aryl methyl ether using Aliquat-336. *Synth. Commun.* **43**, 3272–3280 (2013).
21. Cano, R., Yus, M. & Ramón, D. J. First practical cross-alkylation of primary alcohols with a new and recyclable impregnated iridium on magnetite catalyst. *Chem. Commun.* **48**, 7628–7630 (2012).
22. Jang, W. J., Song, S. M., Moon, J. H., Lee, J. Y. & Yun, J. Copper-catalyzed enantioselective hydroboration of unactivated 1,1-disubstituted alkenes. *J. Am. Chem. Soc.* **139**, 13660–13663 (2017).
23. Eleftheriadis, N., Neochoritis, C. G., Leus, N. G. J., van der Wouden, P. E., Dömling, A. & Dekker, F. J. Rational development of a potent 15-lipoxygenase-1 inhibitor with *in vitro* and *ex vivo* anti-inflammatory properties. *J. Med. Chem.* **58**, 7850–7862 (2015).
24. Sasaki, M., Ando, M., Kawahata, M., Yamaguchi, K. & Takeda, K. Spontaneous oxygenation of siloxy-N-silylketenimines to α-ketoamides. *Org. Lett.* **18**, 1598–1601 (2016).
